# New Syrphidae (Diptera) of North-eastern North America

**DOI:** 10.3897/BDJ.7.e36673

**Published:** 2019-09-03

**Authors:** Jeffrey H. Skevington, Andrew D. Young, Michelle M. Locke, Kevin M. Moran

**Affiliations:** 1 AAFC, Canadian National Collection of Insects, Arachnids and Nematodes, Ottawa, Canada AAFC, Canadian National Collection of Insects, Arachnids and Nematodes Ottawa Canada; 2 Carleton University, Ottawa, Canada Carleton University Ottawa Canada; 3 California Department of Food and Agriculture, Sacramento, United States of America California Department of Food and Agriculture Sacramento United States of America

**Keywords:** new species, taxonomy, flower flies, hover flies, Diptera, Syrphidae, Nearctic

## Abstract

**Background:**

This paper describes 11 of 18 new species recognised in the recent book, "Field Guide to the Flower Flies of Northeastern North America". Four species are omitted as they need to be described in the context of a revision (three *Cheilosia* and a *Palpada* species) and three other species (one *Neoascia* and two *Xylota*) will be described by F. Christian Thompson in a planned publication. Six of the new species have been recognised for decades and were treated by J. Richard Vockeroth in unpublished notes or by Thompson in his unpublished but widely distributed "A conspectus of the flower flies (Diptera: Syrphidae) of the Nearctic Region". Five of the 11 species were discovered during the preparation of the Field Guide. Eight of the 11 have DNA barcodes available that support the morphology.

**New information:**

New species treated in this paper include: *Anasimyiadiffusa* Locke, Skevington and Vockeroth (Smooth-legged Swamp Fly), *Anasimyiamatutina* Locke, Skevington and Vockeroth (Small-spotted Swamp Fly), *Brachyopacaesariata* Moran and Skevington (Plain-winged Sapeater), *Brachyopacummingi* Moran and Skevington (Somber Sapeater), *Hammerschmidtiasedmani* Vockeroth, Moran and Skevington (Pale-bristled Logsitter), Microdon (Microdon) scauros Skevington and Locke (Big-footed Ant Fly), *Mixogasterfattigi* Locke, Skevington and Greene (Fattig's Ant Fly), *Neoasciaguttata* Skevington and Moran (Spotted Fen Fly), *Orthonevrafeei* Moran and Skevington (Fee's Mucksucker), *Psilotaklymkoi* Locke, Young and Skevington (Black Haireye) and *Trichopsomyialitoralis* Vockeroth and Young (Coastal Psyllid-killer). Common names follow the "Field Guide to the Flower Flies of Northeastern North America" (Skevington et al. 2019).

## Introduction

Flower flies (Syrphidae) are important pollinators, perhaps even outperforming native bees in this critical agricultural ecosystem service (Rader et al. 2016, Skevington et al. 2019). In addition to this important role, most immature Syrphinae species are predators of soft-bodied insects such as aphids and scales, many of which are agricultural pests (Rojo et al. 2003, Tenhumberg and Poehling 1995). Many Eristalinae syrphid larvae, such as *Eristalis* Latreille 1804 and *Eristalinus* Rondani 1845, perform critical roles in recycling human and farm animal waste (Pérez-Bañón 2013). Other saprophagous species are found in old growth forests where they specialise in rotholes or under the bark of large trees and are important indicators of habitat health (e.g. *Blerafallax* Linnaeus 1758; Rotheray et al. 2012). Overall, syrphids have offered important insights into the health of our ecosystems and declines in insect numbers (Hallmann et al. 2017, Sánchez-Bayo and Wyckhuys 2019). A few syrphid larvae are phytophagous (with some *Cheilosia* Meigen 1822 used in biological control of plants (Grosskopf 2005)), while those of the subfamily Microdontinae are specialised ant predators and even parasitoids (Pérez-Lachaud et al. 2014). In fact, syrphid larvae are more varied and have more ecological roles than any other family of flies, with the possible exception of Phoridae (McAlpine et al. 1981, McAlpine et al. 1987). The family is species-rich, as well as ecologically diverse, with 828 valid species of Nearctic Syrphidae currently recognised (Skevington 2019) and over 6200 species known worldwide (Pape and Evenhuis 2019). Many syrphids are either perfect or imperfect mimics of bees and wasps, adding to both their interest scientifically (e.g. Edmunds and Reader 2014, Gilbert 2005, Penney et al. 2012, Penney et al. 2014) and to confusion by non-specialists who think that all floral visitors are bees. Many ‘bees’ portrayed in advertisements and even in the scientific literature are actually syrphids.

The popularity of Syrphidae, both as research subjects and as the subject of amateur natural history pursuits, has been increasing in recent years and field guides are emerging that capitalise on this interest. The first such guide was targeted on the United Kingdom fauna and, although not comprehensive, provided an excellent entry into the taxonomy and identification of the group (Ball and Morris 2015). The year 2019 has seen the publication of two comprehensive guides to syrphids. The first, by Bot and Van de Meutter (2019), treated all 384 species that are found, or are expected to be found, in The Netherlands and Belgium. The second, by Skevington et al. (2019) (hereafter, the “Field Guide”), treated 413 species found in north-eastern North America. This book includes 18 undescribed species of Syrphidae that are illustrated but not named. This paper names 11 of these 18 new taxa. Seven species are omitted (three *Cheilosia*, a *Neoascia* Williston 1887 species, a *Palpada* Macquart 1834 species and two *Xylota* Meigen 1822 species). *Cheilosia* is one of the most diverse Nearctic syrphid genera and includes 119 names (Pape and Evenhuis 2019). Moran reworked the eastern Nearctic species for inclusion in the Field Guide but did not study all related type material. Western Nearctic and Palaearctic species need to be included in a complete revision before we can be absolutely sure that morphospecies included in the field guide are not valid but are currently undiagnosable species. *Palpada* is not as complicated, but all types previously assigned as synonyms to *P.furcata* Wiedemann 1819 must be examined to ensure that one does not represent the putative new species. This is complicated by the fact that the putative new Nearctic species is supported largely by DNA data. Until morphological characters can be found to support this new taxon, there is no point in checking type specimens. The *Neoascia* and *Xylota* species are straightforward but will be described by Chris Thompson in a planned publication.

New species to be described herein are in the genera *Anasimyia* Schiner 1864, *Brachyopa* Meigen 1822, *Hammerschmidtia* Schummel 1834, *Microdon* Meigen 1803, *Mixogaster* Macquart 1842, *Neoascia*, *Orthonevra* Macquart 1829, *Psilota* Kieffer 1906 and *Trichopsomyia* Williston 1888. Thompson (1991) studied the Nearctic taxa as part of a plan to publish a Conspectus of Nearctic Syrphidae (hereafter, the “Conspectus”). Although never published, this work has been privately distributed and has served as the backbone of many taxonomic publications, including the Field Guide (Skevington et al. 2019). Thompson and Vockeroth studied most types in preparing the Conspectus and it thus provides a clear indication of taxonomic gaps for the family. Six of the species described herein were first recorded in the Conspectus. Five of the 11 new species were discovered during the preparation of the Field Guide.

## Materials and methods

Adult specimens were pinned directly after collecting or stored in ethanol and later critical-point dried in the case of Malaise trap samples. Clearing and removal of male genitalia were necessary in order to view internal characters, useful for classification and species delimitation. The genitalia capsule was removed by cutting between tergites 7 and 8 and then cleared by heating in 88% lactic acid overnight. A full materials examined list is provided after each species description. Specimens were borrowed from the following institutions and individuals (collection codes based on Evenhuis (2007) are used in the material examined sections):

ANSP - Academy of Natural Sciences of Drexel University, Philadelphia, Pennsylvania, USA

CAS - California Academy of Sciences, San Francisco, California, USA

CBG - Centre for Biodiversity Genomics, Guelph, Ontario, Canada

CNC - Canadian National Collection of Insects, Arachnics, and Nematodes, Ottawa, Ontario, Canada

DEBU - University of Guelph Insect Collection, Guelph, Ontario, Canada

MCZ - Museum of Comparative Zoology, Harvard University, Cambridge, Massachusetts

MEMU - Mississippi State University, Starkville, Mississippi, USA

NBMB - New Brunswick Museum, St. John's, New Brunswick

USNM - National Museum of Natural History, Washington D.C., USA

### Photography

Habitus and genitalia photographs for all specimens were taken using a Leica M205-C stereoscope (Leica Microsystems Inc., Concord, Ontario, Canada) using 0.6x (habitus) and 1.6x (genitalia) lenses. Raw images to be used in depth-of-field photomontages were captured using Leica Application Suite (Leica Microsystems 2019) and final images were created using Zerene Stacker (Littlefield 2018). Specimen measurements were taken using the Leica measurement module in Leica Application Suite. Figures are presented alphabetically.

### DNA Sequencing

The right hind leg was removed from all recently collected (post 2000) specimens and sent to the University of Guelph Biodiversity Institute of Ontario for sequencing of the 5' end of the Cyctochrome *c* Oxidase I mitochondrial gene (COI) or Barcoding region, following protocols published in Hajibabaei et al. (2005). DNA extraction and sequencing from older specimens (most) was performed by Scott Kelso at the CNC using a modified version of the same protocol, with custom primers developed for use in Diptera. These custom primers (Table 1) sequence the Barcoding region in three smaller segments to be assembled as a full barcode, allowing fragmented DNA from older specimens and specimens stored in suboptimal conditions to be successfully sequenced. All sequence data are stored on the BOLD website (www.boldsystems.org) in the 'New Species of Syrphidae from Field Guide' (DS-NSSFG) dataset, available at dx.doi.org/10.5883/DS-NSSFG. GenBank Numbers and unique identifiers ('Sample ID') of specimens sequenced are available in Table 2. With material sequenced at the CNC, raw sequence reads were scored using Sequencher 5.4.6 (Gene Codes Corporation 2019) and aligned using Mesquite (Maddison and Maddison 2010).

### Terminology

Syrphidae-specific terminology follows Thompson (1999), while all other morphological terminology follows Cumming and Wood (2017). A visual glossary of common terminology used is available in Skevington et al. (2019).

### Species Authorship

Authorship of each species is noted in the species description. As the species described below were discovered and described by different people, not all of the authors of this paper contributed equally to each species. In some cases, species discovered by now deceased entomologists are attributed in part to them (depending on how many notes they left on the taxon). In all cases, new data have been brought to bear on the species concepts, including but not limited to genitalic morphology and DNA. Additional authors are added to these species descriptions, based on their relative contributions.

### Notes on Identification

All species included here can be identified by using Skevington et al. (2019). Keys are thus not provided.

## Taxon treatments

### Anasimyia diffusa

Locke, Skevington and Vockeroth sp. n.

2E4A14B6EF15581A94AE0184CC877549

urn:lsid:zoobank.org:act:BF194AD1-F759-4EF0-BBB1-F8DD2EF53C7E

#### Materials

- **Type status:** Holotype. **Occurrence:** catalogNumber: CNC_Diptera102201; recordedBy: D.Blades; individualCount: 1; sex: male; lifeStage: adult; **Taxon:** scientificName: Anasimyiadiffusa Locke, Skevington and Vockeroth; kingdom: Animalia; phylum: Arthropoda; class: Hexapoda; order: Diptera; family: Syrphidae; genus: Anasimyia; specificEpithet: diffusa; taxonRank: species; scientificNameAuthorship: Locke, Skevington and Vockeroth; vernacularName: Smooth-legged Swamp Fly; nomenclaturalStatus: new species; **Location:** country: Canada; stateProvince: Ontario; locality: Oliver Bog, 3km, S Galt; decimalLatitude: 43.3168; decimalLongitude: -80.283; **Identification:** identifiedBy: Michelle M. Locke; dateIdentified: 2019; **Event:** eventDate: 1988-05-03/26; year: 1988; month: 5; day: 3; verbatimEventDate: 3-26.v.1988; **Record Level:** language: en; institutionCode: CNC; basisOfRecord: PreservedSpecimen
- **Type status:** Paratype. **Occurrence:** catalogNumber: CNC640314; recordedBy: D. Beresford; individualCount: 1; sex: female; lifeStage: adult; **Taxon:** scientificName: Anasimyiadiffusa Locke, Skevington and Vockeroth; kingdom: Animalia; phylum: Arthropoda; class: Hexapoda; order: Diptera; family: Syrphidae; genus: Anasimyia; specificEpithet: diffusa; taxonRank: species; scientificNameAuthorship: Locke, Skevington and Vockeroth; vernacularName: Smooth-legged Swamp Fly; nomenclaturalStatus: new species; **Location:** country: Canada; stateProvince: Nunavut; locality: Akimiski Island; decimalLatitude: 53.105; decimalLongitude: -80.957; **Identification:** identifiedBy: Michelle M. Locke; dateIdentified: 2019; **Event:** eventDate: 2013-07; year: 2013; month: 7; verbatimEventDate: vii.2013; **Record Level:** language: en; institutionCode: CNC; basisOfRecord: PreservedSpecimen
- **Type status:** Paratype. **Occurrence:** catalogNumber: CNC640356; recordedBy: D. Beresford; individualCount: 1; sex: female; lifeStage: adult; **Taxon:** scientificName: Anasimyiadiffusa Locke, Skevington and Vockeroth; kingdom: Animalia; phylum: Arthropoda; class: Hexapoda; order: Diptera; family: Syrphidae; genus: Anasimyia; specificEpithet: diffusa; taxonRank: species; scientificNameAuthorship: Locke, Skevington and Vockeroth; vernacularName: Smooth-legged Swamp Fly; nomenclaturalStatus: new species; **Location:** country: Canada; stateProvince: Nunavut; locality: Akimiski Island; decimalLatitude: 53.105; decimalLongitude: -80.957; **Identification:** identifiedBy: Michelle M. Locke; dateIdentified: 2019; **Event:** eventDate: 2013-07; year: 2013; month: 7; verbatimEventDate: vii.2013; **Record Level:** language: en; institutionCode: CNC; basisOfRecord: PreservedSpecimen
- **Type status:** Paratype. **Occurrence:** catalogNumber: CNC640369; recordedBy: Alex Howard & Stan Vasilauskas; individualCount: 1; sex: female; lifeStage: adult; otherCatalogNumbers: 1101721B-2012-088-00008; **Taxon:** scientificName: Anasimyiadiffusa Locke, Skevington and Vockeroth; kingdom: Animalia; phylum: Arthropoda; class: Hexapoda; order: Diptera; family: Syrphidae; genus: Anasimyia; specificEpithet: diffusa; taxonRank: species; scientificNameAuthorship: Locke, Skevington and Vockeroth; vernacularName: Smooth-legged Swamp Fly; nomenclaturalStatus: new species; **Location:** country: Canada; stateProvince: Ontario; locality: District: 2E-1 Albany River; decimalLatitude: 51.6492; decimalLongitude: -81.848522; **Identification:** identifiedBy: Michelle M. Locke; dateIdentified: 2019; **Event:** samplingProtocol: Malaise trap; eventDate: 2012-06-17; year: 2012; month: 6; day: 17; verbatimEventDate: 17.vi.2012; habitat: Forest, Closed Treed cover, Eco District 2E-1 Albany River; **Record Level:** language: en; institutionCode: CNC; basisOfRecord: PreservedSpecimen
- **Type status:** Paratype. **Occurrence:** catalogNumber: CNC640425; recordedBy: Ian Fife & Stan Phippen; individualCount: 1; sex: male; lifeStage: adult; otherCatalogNumbers: 1094706-2011-089-00002; **Taxon:** scientificName: Anasimyiadiffusa Locke, Skevington and Vockeroth; kingdom: Animalia; phylum: Arthropoda; class: Hexapoda; order: Diptera; family: Syrphidae; genus: Anasimyia; specificEpithet: diffusa; taxonRank: species; scientificNameAuthorship: Locke, Skevington and Vockeroth; vernacularName: Smooth-legged Swamp Fly; nomenclaturalStatus: new species; **Location:** country: Canada; stateProvince: Ontario; locality: District: 1E-3 Winisk River; decimalLatitude: 54.4712; decimalLongitude: -88.5649; **Identification:** identifiedBy: Michelle M. Locke; dateIdentified: 2019; **Event:** samplingProtocol: Malaise trap; eventDate: 2011-06-21; year: 2011; month: 6; day: 21; verbatimEventDate: 21.vi.2011; habitat: Forest, Bryophytes, Herbaceous plant cover, Eco District 1E-3 Winisk River; **Record Level:** language: en; institutionCode: CNC; basisOfRecord: PreservedSpecimen
- **Type status:** Paratype. **Occurrence:** catalogNumber: CNC641362; recordedBy: David Beresford; individualCount: 1; sex: male; lifeStage: adult; **Taxon:** scientificName: Anasimyiadiffusa Locke, Skevington and Vockeroth; kingdom: Animalia; phylum: Arthropoda; class: Hexapoda; order: Diptera; family: Syrphidae; genus: Anasimyia; specificEpithet: diffusa; taxonRank: species; scientificNameAuthorship: Locke, Skevington and Vockeroth; vernacularName: Smooth-legged Swamp Fly; nomenclaturalStatus: new species; **Location:** country: Canada; stateProvince: Nunavut; locality: Akimiski Island; decimalLatitude: 53.105; decimalLongitude: -80.957; **Identification:** identifiedBy: Michelle M. Locke; dateIdentified: 2019; **Event:** samplingProtocol: Malaise trap; eventDate: 2017-07-17; year: 2017; month: 7; day: 17; verbatimEventDate: 17.vii.2017; **Record Level:** language: en; institutionCode: CNC; basisOfRecord: PreservedSpecimen
- **Type status:** Paratype. **Occurrence:** catalogNumber: CNC641363; recordedBy: David Beresford; individualCount: 1; sex: male; lifeStage: adult; otherCatalogNumbers: 30/27; **Taxon:** scientificName: Anasimyiadiffusa Locke, Skevington and Vockeroth; kingdom: Animalia; phylum: Arthropoda; class: Hexapoda; order: Diptera; family: Syrphidae; genus: Anasimyia; specificEpithet: diffusa; taxonRank: species; scientificNameAuthorship: Locke, Skevington and Vockeroth; vernacularName: Smooth-legged Swamp Fly; nomenclaturalStatus: new species; **Location:** country: Canada; stateProvince: Nunavut; locality: Akimiski Island; decimalLatitude: 53.105; decimalLongitude: -80.957; **Identification:** identifiedBy: Michelle M. Locke; dateIdentified: 2019; **Event:** samplingProtocol: Malaise trap; eventDate: 2014-07-21; year: 2014; month: 7; day: 21; verbatimEventDate: 21.vii.2014; **Record Level:** language: en; institutionCode: CNC; basisOfRecord: PreservedSpecimen
- **Type status:** Paratype. **Occurrence:** catalogNumber: CNC641364; recordedBy: David Beresford; individualCount: 1; sex: male; lifeStage: adult; otherCatalogNumbers: 45/27; **Taxon:** scientificName: Anasimyiadiffusa Locke, Skevington and Vockeroth; kingdom: Animalia; phylum: Arthropoda; class: Hexapoda; order: Diptera; family: Syrphidae; genus: Anasimyia; specificEpithet: diffusa; taxonRank: species; scientificNameAuthorship: Locke, Skevington and Vockeroth; vernacularName: Smooth-legged Swamp Fly; nomenclaturalStatus: new species; **Location:** country: Canada; stateProvince: Nunavut; locality: Akimiski Island; decimalLatitude: 53.105; decimalLongitude: -80.957; **Identification:** identifiedBy: Michelle M. Locke; dateIdentified: 2019; **Event:** samplingProtocol: Malaise trap; eventDate: 2014-07-21; year: 2014; month: 7; day: 21; verbatimEventDate: 21.vii.2014; **Record Level:** language: en; institutionCode: CNC; basisOfRecord: PreservedSpecimen
- **Type status:** Paratype. **Occurrence:** catalogNumber: CNC_Diptera102200; recordedBy: S.J.Miller; individualCount: 1; sex: male; lifeStage: adult; **Taxon:** scientificName: Anasimyiadiffusa Locke, Skevington and Vockeroth; kingdom: Animalia; phylum: Arthropoda; class: Hexapoda; order: Diptera; family: Syrphidae; genus: Anasimyia; specificEpithet: diffusa; taxonRank: species; scientificNameAuthorship: Locke, Skevington and Vockeroth; vernacularName: Smooth-legged Swamp Fly; nomenclaturalStatus: new species; **Location:** country: Canada; stateProvince: New Brunswick; locality: Kouchibouguac National Park; decimalLatitude: 46.85; decimalLongitude: -64.97; **Identification:** identifiedBy: Michelle M. Locke; dateIdentified: 2019; **Event:** eventDate: 1978-06-15; year: 1978; month: 6; day: 15; verbatimEventDate: 15.vi.1978; **Record Level:** language: en; institutionCode: CNC; basisOfRecord: PreservedSpecimen
- **Type status:** Paratype. **Occurrence:** catalogNumber: CNC_Diptera245059; recordedBy: A. Borkent; individualCount: 1; sex: male; lifeStage: adult; **Taxon:** scientificName: Anasimyiadiffusa Locke, Skevington and Vockeroth; kingdom: Animalia; phylum: Arthropoda; class: Hexapoda; order: Diptera; family: Syrphidae; genus: Anasimyia; specificEpithet: diffusa; taxonRank: species; scientificNameAuthorship: Locke, Skevington and Vockeroth; vernacularName: Smooth-legged Swamp Fly; nomenclaturalStatus: new species; **Location:** country: Canada; stateProvince: British Columbia; locality: 6 km E[ast of] Salmon Arm; decimalLatitude: 50.7; decimalLongitude: -119.3; **Identification:** identifiedBy: Michelle M. Locke; dateIdentified: 2019; **Event:** eventDate: 1992-06-30; year: 1992; month: 4; day: 30; verbatimEventDate: 30.iv.1992; **Record Level:** language: en; institutionCode: CNC; basisOfRecord: PreservedSpecimen
- **Type status:** Paratype. **Occurrence:** catalogNumber: CNC_Diptera284501; recordedBy: CANPOLIN; individualCount: 1; sex: male; lifeStage: adult; otherCatalogNumbers: UG: SYRPH: 10763; **Taxon:** scientificName: Anasimyiadiffusa Locke, Skevington and Vockeroth; kingdom: Animalia; phylum: Arthropoda; class: Hexapoda; order: Diptera; family: Syrphidae; genus: Anasimyia; specificEpithet: diffusa; taxonRank: species; scientificNameAuthorship: Locke, Skevington and Vockeroth; vernacularName: Smooth-legged Swamp Fly; nomenclaturalStatus: new species; **Location:** country: Canada; stateProvince: Manitoba; locality: O'day; decimalLatitude: 57.582; decimalLongitude: -94.069; **Identification:** identifiedBy: Michelle M. Locke; dateIdentified: 2019; **Event:** samplingProtocol: Malaise trap; eventDate: 2011-07-07/19; year: 2011; month: 7; day: 7; verbatimEventDate: 7-19.vii.2011; **Record Level:** language: en; institutionCode: CNC; basisOfRecord: PreservedSpecimen
- **Type status:** Paratype. **Occurrence:** catalogNumber: CNC_Diptera284505; recordedBy: CANPOLIN; individualCount: 1; sex: male; lifeStage: adult; otherCatalogNumbers: UG: SYRPH: 01075; **Taxon:** scientificName: Anasimyiadiffusa Locke, Skevington and Vockeroth; kingdom: Animalia; phylum: Arthropoda; class: Hexapoda; order: Diptera; family: Syrphidae; genus: Anasimyia; specificEpithet: diffusa; taxonRank: species; scientificNameAuthorship: Locke, Skevington and Vockeroth; vernacularName: Smooth-legged Swamp Fly; nomenclaturalStatus: new species; **Location:** country: Canada; stateProvince: Manitoba; locality: Herchmer; decimalLatitude: 57.378; decimalLongitude: -94.195; **Identification:** identifiedBy: Michelle M. Locke; dateIdentified: 2019; **Event:** samplingProtocol: Malaise trap; eventDate: 2011-07-07/19; year: 2011; month: 7; day: 7; verbatimEventDate: 7-19.vii.2011; **Record Level:** language: en; institutionCode: CNC; basisOfRecord: PreservedSpecimen
- **Type status:** Paratype. **Occurrence:** catalogNumber: CNC_Diptera44602; recordedBy: H.J. Teskey; individualCount: 1; sex: male; lifeStage: adult; **Taxon:** scientificName: Anasimyiadiffusa Locke, Skevington and Vockeroth; kingdom: Animalia; phylum: Arthropoda; class: Hexapoda; order: Diptera; family: Syrphidae; genus: Anasimyia; specificEpithet: diffusa; taxonRank: species; scientificNameAuthorship: Locke, Skevington and Vockeroth; vernacularName: Smooth-legged Swamp Fly; nomenclaturalStatus: new species; **Location:** country: Canada; stateProvince: Alberta; locality: Waterton Lakes National Park; decimalLatitude: 49.083314; decimalLongitude: -113.916733; **Identification:** identifiedBy: Michelle M. Locke; dateIdentified: 2019; **Event:** eventDate: 1980-07-07/12; year: 1980; month: 7; day: 7; verbatimEventDate: 7-12.vii.1980; **Record Level:** language: en; institutionCode: CNC; basisOfRecord: PreservedSpecimen
- **Type status:** Paratype. **Occurrence:** catalogNumber: CNC_Diptera44629; recordedBy: A. Borkent; individualCount: 1; sex: male; lifeStage: adult; **Taxon:** scientificName: Anasimyiadiffusa Locke, Skevington and Vockeroth; kingdom: Animalia; phylum: Arthropoda; class: Hexapoda; order: Diptera; family: Syrphidae; genus: Anasimyia; specificEpithet: diffusa; taxonRank: species; scientificNameAuthorship: Locke, Skevington and Vockeroth; vernacularName: Smooth-legged Swamp Fly; nomenclaturalStatus: new species; **Location:** country: Canada; stateProvince: British Columbia; locality: 6 km East of Salmon Arm; decimalLatitude: 50.7; decimalLongitude: -119.2; **Identification:** identifiedBy: Michelle M. Locke; dateIdentified: 2019; **Event:** eventDate: 1990-05-16; year: 1990; month: 5; day: 16; verbatimEventDate: 16.v.1990; **Record Level:** language: en; institutionCode: CNC; basisOfRecord: PreservedSpecimen
- **Type status:** Paratype. **Occurrence:** catalogNumber: CNC_Diptera44630; recordedBy: A. Borkent; individualCount: 1; sex: male; lifeStage: adult; **Taxon:** scientificName: Anasimyiadiffusa Locke, Skevington and Vockeroth; kingdom: Animalia; phylum: Arthropoda; class: Hexapoda; order: Diptera; family: Syrphidae; genus: Anasimyia; specificEpithet: diffusa; taxonRank: species; scientificNameAuthorship: Locke, Skevington and Vockeroth; vernacularName: Smooth-legged Swamp Fly; nomenclaturalStatus: new species; **Location:** country: Canada; stateProvince: British Columbia; locality: 6 km East of Salmon Arm; decimalLatitude: 50.7; decimalLongitude: -119.2; **Identification:** identifiedBy: Michelle M. Locke; dateIdentified: 2019; **Event:** eventDate: 1990-05-16; year: 1990; month: 5; day: 16; verbatimEventDate: 16.v.1990; **Record Level:** language: en; institutionCode: CNC; basisOfRecord: PreservedSpecimen
- **Type status:** Paratype. **Occurrence:** catalogNumber: CNC_Diptera4972; recordedBy: Cooper, Wood; individualCount: 1; sex: female; lifeStage: adult; **Taxon:** scientificName: Anasimyiadiffusa Locke, Skevington and Vockeroth; kingdom: Animalia; phylum: Arthropoda; class: Hexapoda; order: Diptera; family: Syrphidae; genus: Anasimyia; specificEpithet: diffusa; taxonRank: species; scientificNameAuthorship: Locke, Skevington and Vockeroth; vernacularName: Smooth-legged Swamp Fly; nomenclaturalStatus: new species; **Location:** country: Canada; stateProvince: Ontario; locality: Carleton Co. 8 km S Richmond fen; decimalLatitude: 45.069536; decimalLongitude: -75.854558; **Identification:** identifiedBy: Michelle M. Locke; dateIdentified: 2019; **Event:** eventDate: 1983-08-02; year: 1983; month: 8; day: 2; verbatimEventDate: 2.viii.1983; **Record Level:** language: en; institutionCode: CNC; basisOfRecord: PreservedSpecimen
- **Type status:** Paratype. **Occurrence:** catalogNumber: CNC_Diptera4974; recordedBy: S.J. Miller; individualCount: 1; sex: female; lifeStage: adult; **Taxon:** scientificName: Anasimyiadiffusa Locke, Skevington and Vockeroth; kingdom: Animalia; phylum: Arthropoda; class: Hexapoda; order: Diptera; family: Syrphidae; genus: Anasimyia; specificEpithet: diffusa; taxonRank: species; scientificNameAuthorship: Locke, Skevington and Vockeroth; vernacularName: Smooth-legged Swamp Fly; nomenclaturalStatus: new species; **Location:** country: Canada; stateProvince: New Brunswick; locality: Kouchibouguac National Park; decimalLatitude: 46.85; decimalLongitude: -64.97; **Identification:** identifiedBy: Michelle M. Locke; dateIdentified: 2019; **Event:** eventDate: 1978-06-14; year: 1978; month: 6; day: 14; verbatimEventDate: 14.vi.1978; **Record Level:** language: en; institutionCode: CNC; basisOfRecord: PreservedSpecimen
- **Type status:** Paratype. **Occurrence:** catalogNumber: CNC_Diptera91228; recordedBy: J. Fletcher; individualCount: 1; sex: male; lifeStage: adult; **Taxon:** scientificName: Anasimyiadiffusa Locke, Skevington and Vockeroth; kingdom: Animalia; phylum: Arthropoda; class: Hexapoda; order: Diptera; family: Syrphidae; genus: Anasimyia; specificEpithet: diffusa; taxonRank: species; scientificNameAuthorship: Locke, Skevington and Vockeroth; vernacularName: Smooth-legged Swamp Fly; nomenclaturalStatus: new species; **Location:** country: Canada; stateProvince: Manitoba; locality: Aweme; decimalLatitude: 49.7; decimalLongitude: -99.6; **Identification:** identifiedBy: Michelle M. Locke; dateIdentified: 2019; **Event:** eventDate: 1907-08-25; year: 1907; month: 8; day: 25; verbatimEventDate: 25.viii.1907; **Record Level:** language: en; institutionCode: CNC; basisOfRecord: PreservedSpecimen
- **Type status:** Paratype. **Occurrence:** catalogNumber: Jeff_Skevington_Specimen17433; recordedBy: D. Blades; individualCount: 1; sex: male; lifeStage: adult; otherCatalogNumbers: accession#026W; **Taxon:** scientificName: Anasimyiadiffusa Locke, Skevington and Vockeroth; kingdom: Animalia; phylum: Arthropoda; class: Hexapoda; order: Diptera; family: Syrphidae; genus: Anasimyia; specificEpithet: diffusa; taxonRank: species; scientificNameAuthorship: Locke, Skevington and Vockeroth; vernacularName: Smooth-legged Swamp Fly; nomenclaturalStatus: new species; **Location:** country: Canada; stateProvince: Ontario; locality: Oliver Bog, 3km, S Galt; decimalLatitude: 43.3168; decimalLongitude: -80.283; **Identification:** identifiedBy: Michelle M. Locke; dateIdentified: 2019; **Event:** samplingProtocol: pan trap; eventDate: 1988-05-03/26; year: 1988; month: 5; day: 3; verbatimEventDate: 3-26.v.1988; habitat: pt2 expanse hollow; **Record Level:** language: en; institutionCode: DEBU; basisOfRecord: PreservedSpecimen
- **Type status:** Paratype. **Occurrence:** catalogNumber: Jeff_Skevington_Specimen17434; recordedBy: D. Blades; individualCount: 1; sex: male; lifeStage: adult; otherCatalogNumbers: accession#026W; **Taxon:** scientificName: Anasimyiadiffusa Locke, Skevington and Vockeroth; kingdom: Animalia; phylum: Arthropoda; class: Hexapoda; order: Diptera; family: Syrphidae; genus: Anasimyia; specificEpithet: diffusa; taxonRank: species; scientificNameAuthorship: Locke, Skevington and Vockeroth; vernacularName: Smooth-legged Swamp Fly; nomenclaturalStatus: new species; **Location:** country: Canada; stateProvince: Ontario; locality: Oliver Bog, 3km, S Galt; decimalLatitude: 43.3168; decimalLongitude: -80.283; **Identification:** identifiedBy: Michelle M. Locke; dateIdentified: 2019; **Event:** eventDate: 1988-05-03/26; year: 1988; month: 5; day: 3; verbatimEventDate: 3-26.v.1988; habitat: pt2 expanse hollow; **Record Level:** language: en; institutionCode: DEBU; basisOfRecord: PreservedSpecimen

#### Description

**Size**: Body length 7.6 to 10.7 mm; wing length 5.6 to 7.4 mm

**Male: Head**: Face yellow, covered in yellow pollen and fine yellow pile, sides of face black, shining and bare, small medial tubercle, lower face projecting forwards along the oral margin; gena black, covered in yellow pollen and fine yellow pile; frons black dorsally, yellow ventrally, sometimes narrowly so just along ventral edge and across dorsum of antennal socket, dense yellow pollen often obscures ground colour, narrow shining spot dorsal to the antennal socket, yellow pilose, sometimes with a few black pili; vertex black, mostly black pilose with some yellow pile; antenna wholly yellow to orange, scape and pedicel with short, black pile, pedicel slightly longer than scape, postpedicel round; eye bare.

**Thorax**: Scutum dull black with two medial, narrow, yellow pollinose stripes, lateral edges yellow pollinose from postpronotum to postalar callus, yellow pilose; postpronotum black, covered in yellow pollen and yellow pile; postalar callus black, covered in yellow pollen and yellow pile; scutellum yellow with black lateral edges and narrowly black along anterior edge, yellow pilose with some black pili admixed; pleuron black, covered in yellow pollen, anterior anepisternum, katepimeron and meron bare, posterior anepisternum, katepisternum and anepimeron yellow pilose; metasternum black, yellow pollinose, yellow pilose; pro- and mesolegs with coxae and trochanters black, yellow pilose, femora black on basal ½, yellow on apical ½, black extends almost to apex anteroventrally, sometimes more or less extensively black, anteroventral basal patch of black setulae, yellow pilose; protibia yellow, usually brown to black anteriorly on apical ¼-⅓, covered in short, yellow pile, protarsus brown anteriorly, yellow posteriorly, similar in colour to apex of protibia, covered in short yellow pile, few black pili anteriorly; mesotibia yellow, sometimes brown, yellow pilose; mesotarsus yellow basally, brown to black apically, tarsomeres 1 and 2 yellow, 3 yellow or brown, 4 and 5 brown, all yellow pilose with black setulae posteriorly; metaleg with coxa black, yellow pollinose, yellow pilose; metatrochanter without tubercle, black, shining, long yellow pile anteriorly, some short yellow pile posteriorly; metafemur enlarged, yellow, anteromedial spot ranging from obscure brown to black spot ~⅕ the length of the metafemur to large black spot on medial ½ of femur, posterior side ⅔-¾ black medially, dorsally extending to apex, apical end of metafemur narrowly black, metafemur yellow pilose, small patch of black setulae anterobasally, black setulae along ventral side; metatibia brown to black basally and apically, yellow on medial ¼-⅓, with ventral carinate ridge, yellow pilose, apical end projected forwards slightly beyond end of tibia not formed into noticeable spur; metatarsus brown to black, black pilose anteriorly, yellow pilose posteriorly; wing entirely densely microtrichose; halter yellow; calypter yellow.

**Abdomen**: Black, yellow pilose with few black pili admixed; tergite 1 pollinose anterolaterally; tergite 2 yellow laterally, widely on anterior ¾-⅘, usually narrowly on posterior ⅕-¼, yellow extending medially on to tergite and tapering to a point about ¼-⅓ from lateral edge, yellow cuticle covered in yellow pollen, sometimes extending slightly on to black cuticle medially where it tapers to a point; tergite 3 with anterolateral yellow spots ½ length of tergite, often narrowly yellow laterally on posterior half, pair of yellow to grey curved pollinose markings medially; tergite 4 similar to tergite 3 but lateral edges only narrowly yellow, no anterolateral yellow spots, narrowly yellow posteriorly (tergite 3 sometimes very narrowly yellow posteriorly as well); tergites 2-4 shining on posterior ⅕-½, typically more widely shining on tergites 3 and 4; sternite 1 black on anterior ¾, yellow on posterior ¼, black area convex; sternite 2 yellow, often with brown to black spot posteromedially, spot often obscure, sternite 3 often brown to black with posterior edge narrowly yellow, sometimes yellow on anterior ½; sternite 4 brown to black with posterior edge narrowly yellow; all sternites yellow to grey pollinose with yellow pile.

**Genitalia**: Epandrium longer than wide, narrows slightly dorsally, with apicodorsal ridge adjacent to cerci; cercus with laterally compressed, subquadrate, sclerotised outer portion covered in long pile and membranous inner portion; surstylus long and wide, bent, with small patch of setae on ventral inner surface at bend, apical end bilobed and curved inwards, dorsal and ventral lobes covered in setae; basal hypandrium convexly curved ventrally, with two low, parallel ridges ending with several posterior-facing, curved setae at apicoventral end (one pair of setae larger than other setae); apical hypandrium split into two complex, multi-lobed arms; phallapodeme long, rod-like structure, laterally compressed and curved dorsally at base; phallus is difficult to distinguish, hidden between the multi-lobed hypandrial arms, small, short, with two pairs of lobes, basal lobes broad and directed laterally, distal lobes narrow and directed apically, with separate dorsal conical structure, pointed at apex (possibly secondarily derived from surrounding membrane).

**Female**: Differs from male in the following ways: frons black with narrow yellow ventral edge including dorsum of antennal socket, black pilose; scutellum often entirely yellow pilose, sometimes with few black pili admixed; pro- and mesofemora black on basal ⅓-½, yellow on apical ½-⅔, black extends almost to apex ventrally, sometimes less extensively black basally with black not extending entirely around femur; metafemur with brown anterior spot smaller than in male, sometimes completely absent; metatibia similar to male but often more yellow with brown areas reduced especially basally; abdomen with short subappressed yellow and black pile; tergite 1 entirely pollinose, with short, yellow subappressed pile; tergite 2 yellow anterolaterally, sometimes with yellow extending narrowly along lateral margin, with pair of curved pollinose markings beginning anterolaterally and curving inwards medially, pile subappressed with some long pile on anterolateral edge, yellow pilose on anterior ¾ or more, black pilose on posterior ¼ or less, posterior edge shining; tergites 3 and 4 similar to tergite 2, sometimes with posterior edge narrowly yellow and with small medial pollinose marking posteromedially, often more extensively black pilose on posterior ½ or less, pile uniformly short, no long pile anterolaterally, tergites 3 and 4 not as long as tergite 2; tergite 5 smaller than other tergites, black anteriorly, yellow posteriorly, tergite almost entirely pollinose except along anterior edge and narrow medial stripe, yellow pilose with few black pili posteriorly; sternites pollinose and yellow pilose; sternite one black; sternite 2 mostly yellow with black medial triangular marking; sternites 3 and 4 mostly black with narrow yellow lateral and sometimes posterior edges.

See Fig. 1 & Fig. 2.

#### Diagnosis

Male metatrochanter without tubercle, with fine, yellow pile anteriorly (Fig. 1e); metatibia without spur (Fig. 1d) and tergite 4 with large pair of curved pollinose markings.

#### Etymology

The word *diffusa* is Latin, meaning spread out, extended, dispersed. This is the name given to this species by J.R. Vockeroth, presumably with reference to the wide distribution.

#### Distribution

This species is known from 20 specimens from New Brunswick to British Columbia.

#### Ecology

*Anasimyiadiffusa* is often collected in the same location as the much more common *A.anausis* (Walker 1849). They appear to both have a broad distribution and a long flight season, with *A.diffusa* collected from mid-April to late August. Some specimens have been collected in bogs but it is unclear if it is a bog specialist or not.

#### Taxon discussion

Differences in the metatrochanter are the easiest way to distinguish *A.diffusa* from *A.anausis*. The metatrochanter of *A.anausis* has short black setuale anteroventrally. In some specimens these setulae appear to be broken off but their insertions into the cuticle give the metatrochanter a rough texture. *Anasimyiabilinearis* (Williston 1887) can be differentiated from *A.diffusa* by its mostly dark abdomen with reduced pollinose markings. There is no tubercle on the metatrochanter of *A.diffusa* as there is in *Anasimyia* Group 2 males (see Skevington et al. 2019). Females are similar to other Group 1 females (see Skevington et al. 2019) and cannot be distinguished from similar species. Five females of *A.diffusa* were identified using the barcoding region of COI. These specimens in the paratype series clustered in a neighbour-joining tree with four males that were morphologically identified to *A.diffusa*. DNA sequences of this species are available in the public BOLD dataset "New Species of Syrphidae from Field Guide - DS-NSSFG" and in GenBank (Table 2). They are genetically most similar to *A.anausis* but consistently separable with DNA barcodes.

#### Common Name

The common name given to the species by Skevington et al. (2019) is Smooth-legged Swamp Fly.

### Anasimyia matutina

Locke, Skevington and Vockeroth sp. n.

C90980C8E6295B1CB0853A15AD99B8E1

urn:lsid:zoobank.org:act:2FCCCB1B-FBA1-4E33-BAE6-F5D722A5ECF8

#### Materials

- **Type status:** Holotype. **Occurrence:** catalogNumber: CNC_Diptera91232; recordedBy: N. Banks; individualCount: 1; sex: male; lifeStage: adult; **Taxon:** scientificName: Anasimyiamatutina Locke, Skevington and Vockeroth; kingdom: Animalia; phylum: Arthropoda; class: Hexapoda; order: Diptera; family: Syrphidae; genus: Anasimyia; specificEpithet: matutina; taxonRank: species; scientificNameAuthorship: Locke, Skevington and Vockeroth; vernacularName: Small-spotted Swamp Fly; nomenclaturalStatus: new species; **Location:** country: U.S.A.; stateProvince: Virginia; locality: Great Falls; decimalLatitude: 39; decimalLongitude: -77.283333; **Identification:** identifiedBy: J.R. Vockeroth; **Event:** samplingProtocol: hand collected; eventDate: 06/16/1952; year: 1952; month: 6; day: 16; verbatimEventDate: 16.vi.1952; **Record Level:** language: en; institutionCode: CNC; basisOfRecord: PreservedSpecimen
- **Type status:** Paratype. **Occurrence:** catalogNumber: CNC_Diptera91233; individualCount: 1; sex: male; lifeStage: adult; **Taxon:** scientificName: Anasimyiamatutina Locke, Skevington and Vockeroth; kingdom: Animalia; phylum: Arthropoda; class: Hexapoda; order: Diptera; family: Syrphidae; genus: Anasimyia; specificEpithet: matutina; taxonRank: species; scientificNameAuthorship: Locke, Skevington and Vockeroth; vernacularName: Small-spotted Swamp Fly; nomenclaturalStatus: new species; **Location:** country: U.S.A.; stateProvince: Virginia; locality: Petersburg, Chesterford County; decimalLatitude: 37.227858; decimalLongitude: -77.40195; **Identification:** identifiedBy: J.R. Vockeroth; **Event:** samplingProtocol: hand collected; eventDate: 06/01/1917; year: 1917; month: 6; day: 1; verbatimEventDate: 1.vi.1917; **Record Level:** language: en; institutionCode: CNC; basisOfRecord: PreservedSpecimen
- **Type status:** Paratype. **Occurrence:** catalogNumber: CNC_Diptera91234; recordedBy: Fred K. Knab; individualCount: 1; sex: male; lifeStage: adult; **Taxon:** scientificName: Anasimyiamatutina Locke, Skevington and Vockeroth; kingdom: Animalia; phylum: Arthropoda; class: Hexapoda; order: Diptera; family: Syrphidae; genus: Anasimyia; specificEpithet: matutina; taxonRank: species; scientificNameAuthorship: Locke, Skevington and Vockeroth; vernacularName: Small-spotted Swamp Fly; nomenclaturalStatus: new species; **Location:** country: U.S.A.; stateProvince: Washington, D.C.; locality: Washington; decimalLatitude: 38.9; decimalLongitude: -77.033333; **Identification:** identifiedBy: J.R. Vockeroth; **Event:** samplingProtocol: hand collected; eventDate: 05/17/1912; year: 1912; month: 5; day: 17; verbatimEventDate: 17.v.1912; **Record Level:** language: en; institutionCode: CNC; basisOfRecord: PreservedSpecimen
- **Type status:** Paratype. **Occurrence:** catalogNumber: Jeff_Skevington_Specimen17432; recordedBy: N. Banks; individualCount: 1; sex: male; lifeStage: adult; **Taxon:** scientificName: Anasimyiamatutina Locke, Skevington and Vockeroth; kingdom: Animalia; phylum: Arthropoda; class: Hexapoda; order: Diptera; family: Syrphidae; genus: Anasimyia; specificEpithet: matutina; taxonRank: species; scientificNameAuthorship: Locke, Skevington and Vockeroth; vernacularName: Small-spotted Swamp Fly; nomenclaturalStatus: new species; **Location:** country: U.S.A.; stateProvince: Virginia; locality: Chain Bridge; decimalLatitude: 38.929911; decimalLongitude: -77.114411; **Identification:** identifiedBy: J.R. Vockeroth; **Event:** samplingProtocol: hand collected; eventDate: 06-14; month: 6; day: 14; verbatimEventDate: 14.vi; **Record Level:** language: en; institutionCode: CNC; basisOfRecord: PreservedSpecimen

#### Description

**Size**: Body length 9.8 to 10.2 mm; wing length 6.1 to 6.4 mm

**Male: Head**: Face yellow, covered in yellow pollen and fine yellow pile, sides of face black, shining and bare, small medial tubercle, lower face projecting forwards along the oral margin; gena black, covered in yellow pollen and fine yellow pile; frons black dorsally, yellow ventrally, dense yellow pollen often obscures ground colour, narrow shining spot dorsal to the antennal socket, yellow pilose; vertex black, mostly black pilose with some yellow pile; antenna wholly yellow to orange, scape and pedicel with short, black pile, pedicel slightly longer than scape, postpedicel round; eye bare.

**Thorax**: Scutum dull black with two medial, yellow pollinose stripes that are more than ½ as wide as adjacent black area, lateral edges yellow pollinose from postpronotum to postalar callus, yellow pilose; postpronotum black, covered in yellow pollen and yellow pile; postalar callus black, covered in yellow pollen and yellow pile; scutellum black on basal ½, yellow on apical ½, yellow pilose; pleuron black, covered in yellow pollen, anterior anepisternum, katepimeron and meron bare, posterior anepisternum, katepisternum and anepimeron yellow pilose; metasternum black, yellow pollinose, yellow pilose; procoxa black, yellow pollinose, yellow pilose; protrochanter black, yellow pollinose, yellow pilose with a few black setulae apically; profemur black on basal ½-⅔, yellow on apical ⅓-½, yellow pollinose, yellow pilose, anteroventral basal patch of black setulae with cuticle shining below; protibia yellow, yellow pilose; protarsus yellow, yellow pilose, apical two tarsomeres sometimes darkened brown; mesocoxa black, yellow pollinose, yellow pilose, few black setulae apically; mesotrochanter black, yellow pollinose, yellow pilose, few black setulae apically; mesofemur black on basal ½, yellow on apical ½, yellow pollinose, yellow pilose, anteroventral basal patch of black setulae; mesotibia yellow, yellow pilose with few black setulae ventrally on apical end; mesotarsus yellow, yellow pilose with black setulae ventrally, apical one or two tarsomeres sometimes slightly darkened brown; metacoxa black, yellow pollinose, yellow pilose anteriorly; metatrochanter black, yellow pilose with small ventral tubercle; metafemur enlarged, black basal ⅔, yellow apical ⅓ with apex narrowly black, yellow pilose, black setulae ventrally on apical ⅔; metatibia light brown, sometimes yellow, on basal ⅓, yellow on medial ⅓, dark brown (darker than base) on apical ⅓ (sometimes >⅓), yellow pilose, with ventral carinate ridge ending in long apical spur, spur ¼-⅓ as long as basotarsomere; metatarsus brown, black pilose anteriorly, yellow pilose posteriorly; wing entirely densely microtrichose; halter yellow; calypter yellow.

**Abdomen**: Tergite 1 black, small yellow spots on lateral edges, covered in yellow to grey pollen, yellow pilose; tergite 2 elongate, longer than wide and longer than tergite 3, slightly narrowing posteriorly, yellow laterally, black medially forming a ‘v’ shape that is widest anteriorly, narrowest medially, posterior ¼ brown to black, pair of faint yellow pollinose markings on yellow cuticle that extend from anterolateral edge and curve inwards medially, yellow pilose, posterior edge shining; tergite 3 brown to reddish to black, sometimes more yellow to orange, usually not uniform in colour and without a distinct pattern, anterolateral corners yellow with yellow pollen, yellow pilose, posterior edge shining; tergite 4 similar to tergite 3 but usually with anterior medial pair of small pollinose spots, faint pollinose spot between dull anterior portion and shining posterior margin; sternite 1 black, yellow to grey pollinose, yellow pilose; sternite 2 yellow anteriorly, black posteriorly, black extends to or almost to anterior edge in a narrow, medial point; sternite 3 brown to black anteriorly, yellow to brown posteriorly, amount varies from ¼ black to almost entirely black; sternite 4 yellow to brown; sternites 2-4 covered in yellow to grey pollen, pollen is more dense, often forming bands, along the posterior edge, yellow pilose.

**Genitalia**: Epandrium subquadrate, longer than wide, narrows slightly dorsally; cercus with laterally compressed, subquadrate sclerotised outer portion covered in long pile and membranous inner portion; surstylus long, bent at a 90° angle, with setae on ventral inner surface, apical end triangular, curved inwards, covered in setae on dorsal and ventral edges; basal hypandrium with central, basoventral pointed process, directed apically, narrows medially, deeply concave ventrally, with several posteroventral-facing curved setae at apicoventral end (one pair of setae longer than other setae); apical hypandrium split into two complex, multi-lobed arms; phallapodeme long, rod-like, laterally compressed and curved dorsally at base; phallus situated between the multi-lobed hypandrial arms, small and difficult to distinguish, with separate small, dorsal conical structure, pointed at apex (possibly secondarily derived from surrounding membrane).

**Female**: Unknown.

See Fig. 3 & Fig. 4.

#### Diagnosis

Male metatrochanter with modest tubercle and long, acute apical metatibial spur (Fig. 3d).

#### Etymology

The word *matutina* is Latin, meaning of the morning, early. This is the name given to this species by J.R. Vockeroth.

#### Distribution

Only four specimens are known from Virginia (Chain Bridge, Great Falls and Petersburg) and Washington DC.

#### Ecology

Rarely found, therefore nothing is known about its habitat association. It is known to fly from mid-May to mid-June.

#### Taxon discussion

Size and shape of spur on metatrochanter and of metatibial spur separate this from other *Anasimyia* Group 2 males (see Skevington et al. 2019). Females are unknown, but are likely to be identifiable as *Anasimyia* Group 2 females (Skevington et al. 2019).

#### Common Name

The common name given to the species by Skevington et al. (2019) is Small-spotted Swamp Fly.

### Brachyopa caesariata

Moran and Skevington sp. n.

78F4002E31AB5B4ABF8F3A22C74B52FD

urn:lsid:zoobank.org:act:A39E0E80-49A9-41F9-877A-D9B3AF5A2347

#### Materials

- **Type status:** Holotype. **Occurrence:** catalogNumber: CNC1042805; recordedBy: J.H.Skevington, M.M. Locke; individualCount: 1; sex: male; lifeStage: adult; otherCatalogNumbers: JSM11211; **Taxon:** scientificName: Brachyopacaesariata Moran and Skevington; kingdom: Animalia; phylum: Arthropoda; class: Hexapoda; order: Diptera; family: Syrphidae; genus: Brachyopa; specificEpithet: caesariata; taxonRank: species; scientificNameAuthorship: Moran and Skevington; vernacularName: Plain-winged Sapeater; nomenclaturalStatus: new species; **Location:** country: Canada; stateProvince: Ontario; locality: Algonquin Provincial Park, Opeongo Road; decimalLatitude: 45.625911; decimalLongitude: -78.350753; **Identification:** identifiedBy: K. Moran; **Event:** eventDate: 06/03/2018; year: 2018; month: 6; day: 3; verbatimEventDate: 3.vi.2018; habitat: on flowers of Prunusvirginiana; **Record Level:** language: en; institutionCode: CNC; basisOfRecord: PreservedSpecimen
- **Type status:** Paratype. **Occurrence:** catalogNumber: CNC1042806; recordedBy: J.H.Skevington, M.M. Locke; individualCount: 1; sex: male; lifeStage: adult; otherCatalogNumbers: JSM11212; **Taxon:** scientificName: Brachyopacaesariata Moran and Skevington; kingdom: Animalia; phylum: Arthropoda; class: Hexapoda; order: Diptera; family: Syrphidae; genus: Brachyopa; specificEpithet: caesariata; taxonRank: species; scientificNameAuthorship: Moran and Skevington; vernacularName: Plain-winged Sapeater; nomenclaturalStatus: new species; **Location:** country: Canada; stateProvince: Ontario; locality: Algonquin Provincial Park, Opeongo Road; decimalLatitude: 45.625911; decimalLongitude: -78.350753; **Identification:** identifiedBy: K. Moran; **Event:** eventDate: 06/03/2018; year: 2018; month: 6; day: 3; verbatimEventDate: 3.vi.2018; habitat: on flowers of Prunusvirginiana; **Record Level:** language: en; institutionCode: CNC; basisOfRecord: PreservedSpecimen
- **Type status:** Paratype. **Occurrence:** catalogNumber: CNC1185344; recordedBy: A.L. Melander; individualCount: 1; sex: male; lifeStage: adult; **Taxon:** scientificName: Brachyopacaesariata Moran and Skevington; kingdom: Animalia; phylum: Arthropoda; class: Hexapoda; order: Diptera; family: Syrphidae; genus: Brachyopa; specificEpithet: caesariata; taxonRank: species; scientificNameAuthorship: Moran and Skevington; vernacularName: Plain-winged Sapeater; nomenclaturalStatus: new species; **Location:** country: U.S.A.; stateProvince: Washington; locality: ValleyFord; decimalLatitude: 47.535775; decimalLongitude: -117.238994; **Identification:** identifiedBy: F.C. Thompson; **Event:** eventDate: 05/17/1924; year: 1924; month: 5; day: 17; verbatimEventDate: 17.v.1924; **Record Level:** language: en; institutionCode: USNM; basisOfRecord: PreservedSpecimen
- **Type status:** Paratype. **Occurrence:** catalogNumber: CNC1185345; recordedBy: O. Bryant; individualCount: 1; sex: male; lifeStage: adult; **Taxon:** scientificName: Brachyopacaesariata Moran and Skevington; kingdom: Animalia; phylum: Arthropoda; class: Hexapoda; order: Diptera; family: Syrphidae; genus: Brachyopa; specificEpithet: caesariata; taxonRank: species; scientificNameAuthorship: Moran and Skevington; vernacularName: Plain-winged Sapeater; nomenclaturalStatus: new species; **Location:** country: Canada; stateProvince: British Columbia; locality: Kamloops; decimalLatitude: 50.674234; decimalLongitude: -120.299615; **Identification:** identifiedBy: F.C. Thompson; **Event:** eventDate: 05/01/1932; year: 1932; month: 5; day: 1; verbatimEventDate: 1.v.1932; **Record Level:** language: en; institutionCode: CAS; basisOfRecord: PreservedSpecimen
- **Type status:** Paratype. **Occurrence:** catalogNumber: CNC1185346; recordedBy: V. Argo; individualCount: 1; sex: male; lifeStage: adult; **Taxon:** scientificName: Brachyopacaesariata Moran and Skevington; kingdom: Animalia; phylum: Arthropoda; class: Hexapoda; order: Diptera; family: Syrphidae; genus: Brachyopa; specificEpithet: caesariata; taxonRank: species; scientificNameAuthorship: Moran and Skevington; vernacularName: Plain-winged Sapeater; nomenclaturalStatus: new species; **Location:** country: U.S.A.; stateProvince: Washington; locality: Walla Walla; decimalLatitude: 46.069657; decimalLongitude: -118.356384; **Identification:** identifiedBy: F.C. Thompson; **Event:** eventDate: 04/12/1924; year: 1924; month: 4; day: 12; verbatimEventDate: 12.iv.1924; **Record Level:** language: en; institutionCode: USNM; basisOfRecord: PreservedSpecimen
- **Type status:** Paratype. **Occurrence:** catalogNumber: CNC1185347; recordedBy: V. Argo; individualCount: 1; sex: male; lifeStage: adult; otherCatalogNumbers: USNMENT01518169; **Taxon:** scientificName: Brachyopacaesariata Moran and Skevington; kingdom: Animalia; phylum: Arthropoda; class: Hexapoda; order: Diptera; family: Syrphidae; genus: Brachyopa; specificEpithet: caesariata; taxonRank: species; scientificNameAuthorship: Moran and Skevington; vernacularName: Plain-winged Sapeater; nomenclaturalStatus: new species; **Location:** country: U.S.A.; stateProvince: Washington; locality: Asotin; decimalLatitude: 46.341789; decimalLongitude: -117.055822; **Identification:** identifiedBy: F.C. Thompson; **Event:** eventDate: 04/06/1924; year: 1924; month: 4; day: 6; verbatimEventDate: 6.iv.1924; **Record Level:** language: en; institutionCode: USNM; basisOfRecord: PreservedSpecimen
- **Type status:** Paratype. **Occurrence:** catalogNumber: CNC1185348; individualCount: 1; sex: male; lifeStage: adult; **Taxon:** scientificName: Brachyopacaesariata Moran and Skevington; kingdom: Animalia; phylum: Arthropoda; class: Hexapoda; order: Diptera; family: Syrphidae; genus: Brachyopa; specificEpithet: caesariata; taxonRank: species; scientificNameAuthorship: Moran and Skevington; vernacularName: Plain-winged Sapeater; nomenclaturalStatus: new species; **Location:** country: U.S.A.; stateProvince: Utah; locality: Cache Co., Logan Canyon, Twin Creek; decimalLatitude: 41.867666; decimalLongitude: -111.594781; **Identification:** identifiedBy: F.C. Thompson; **Event:** eventDate: 1979-06-12/21; startDayOfYear: 163; endDayOfYear: 172; year: 1979; month: 6; day: 12; verbatimEventDate: 12-21.vi.1979; **Record Level:** language: en; institutionCode: USNM; basisOfRecord: PreservedSpecimen
- **Type status:** Paratype. **Occurrence:** catalogNumber: CNC_Diptera101644; recordedBy: Dbell,M.Wood; individualCount: 1; sex: male; lifeStage: adult; **Taxon:** scientificName: Brachyopacaesariata Moran and Skevington; kingdom: Animalia; phylum: Arthropoda; class: Hexapoda; order: Diptera; family: Syrphidae; genus: Brachyopa; specificEpithet: caesariata; taxonRank: species; scientificNameAuthorship: Moran and Skevington; vernacularName: Plain-winged Sapeater; nomenclaturalStatus: new species; **Location:** country: Canada; stateProvince: Ontario; locality: Maynooth, Hastings County; decimalLatitude: 45.230639; decimalLongitude: -77.938375; **Identification:** identifiedBy: K. Moran; **Event:** eventDate: 05/10/1987; year: 1987; month: 5; day: 10; verbatimEventDate: 10.v.1987; **Record Level:** language: en; institutionCode: CNC; basisOfRecord: PreservedSpecimen
- **Type status:** Paratype. **Occurrence:** catalogNumber: CNC_Diptera106863; recordedBy: D. Bell, M. Wood; individualCount: 1; sex: female; lifeStage: adult; **Taxon:** scientificName: Brachyopacaesariata Moran and Skevington; kingdom: Animalia; phylum: Arthropoda; class: Hexapoda; order: Diptera; family: Syrphidae; genus: Brachyopa; specificEpithet: caesariata; taxonRank: species; scientificNameAuthorship: Moran and Skevington; vernacularName: Plain-winged Sapeater; nomenclaturalStatus: new species; **Location:** country: Canada; stateProvince: Ontario; locality: Maynooth, Hastings County; decimalLatitude: 45.230639; decimalLongitude: -77.938375; **Identification:** identifiedBy: K. Moran; **Event:** eventDate: 05/10/1987; year: 1987; month: 5; day: 10; verbatimEventDate: 10.v.1987; **Record Level:** language: en; institutionCode: CNC; basisOfRecord: PreservedSpecimen
- **Type status:** Paratype. **Occurrence:** catalogNumber: CNC_Diptera107450; recordedBy: S. & J. Peck; individualCount: 1; sex: male; lifeStage: adult; **Taxon:** scientificName: Brachyopacaesariata Moran and Skevington; kingdom: Animalia; phylum: Arthropoda; class: Hexapoda; order: Diptera; family: Syrphidae; genus: Brachyopa; specificEpithet: caesariata; taxonRank: species; scientificNameAuthorship: Moran and Skevington; vernacularName: Plain-winged Sapeater; nomenclaturalStatus: new species; **Location:** country: U.S.A.; stateProvince: Alaska; locality: 11 Miles South Anderson Junction, Mile 270 Rte 3; decimalLatitude: 64.165328; decimalLongitude: -149.287278; **Identification:** identifiedBy: K. Moran; **Event:** samplingProtocol: Malaise trap; eventDate: 1984-06/08-23/11; startDayOfYear: 174; endDayOfYear: 224; year: 1984; month: 6; day: 23; verbatimEventDate: 23.vi.-11.viii.1984; habitat: Alnus Populus-Picea; **Record Level:** language: en; institutionCode: CNC; basisOfRecord: PreservedSpecimen
- **Type status:** Paratype. **Occurrence:** catalogNumber: CNC_Diptera107451; recordedBy: S. & J. Peck; individualCount: 1; sex: male; lifeStage: adult; **Taxon:** scientificName: Brachyopacaesariata Moran and Skevington; kingdom: Animalia; phylum: Arthropoda; class: Hexapoda; order: Diptera; family: Syrphidae; genus: Brachyopa; specificEpithet: caesariata; taxonRank: species; scientificNameAuthorship: Moran and Skevington; vernacularName: Plain-winged Sapeater; nomenclaturalStatus: new species; **Location:** country: U.S.A.; stateProvince: Alaska; locality: 11 Miles South Anderson Junction, Mile 270 Rte 3; decimalLatitude: 64.165328; decimalLongitude: -149.287278; **Identification:** identifiedBy: K. Moran; **Event:** samplingProtocol: Malaise trap; eventDate: 1984-06/08-23/11; startDayOfYear: 174; endDayOfYear: 224; year: 1984; month: 6; day: 23; verbatimEventDate: 23.vi.-11.viii.1984; habitat: Alnus Populus-Picea; **Record Level:** language: en; institutionCode: CNC; basisOfRecord: PreservedSpecimen
- **Type status:** Paratype. **Occurrence:** catalogNumber: CNC_Diptera107454; recordedBy: S. & J. Peck; individualCount: 1; sex: male; lifeStage: adult; **Taxon:** scientificName: Brachyopacaesariata Moran and Skevington; kingdom: Animalia; phylum: Arthropoda; class: Hexapoda; order: Diptera; family: Syrphidae; genus: Brachyopa; specificEpithet: caesariata; taxonRank: species; scientificNameAuthorship: Moran and Skevington; vernacularName: Plain-winged Sapeater; nomenclaturalStatus: new species; **Location:** country: U.S.A.; stateProvince: Alaska; locality: 11 Miles South Anderson Junction, Mile 270 Rte 3; decimalLatitude: 64.165328; decimalLongitude: -149.287278; **Identification:** identifiedBy: K. Moran; **Event:** samplingProtocol: Malaise trap; eventDate: 1984-06/08-23/11; startDayOfYear: 174; endDayOfYear: 224; year: 1984; month: 6; day: 23; verbatimEventDate: 23.vi.-11.viii.1984; habitat: Alnus Populus-Picea; **Record Level:** language: en; institutionCode: CNC; basisOfRecord: PreservedSpecimen
- **Type status:** Paratype. **Occurrence:** catalogNumber: CNC_Diptera107456; recordedBy: S. & J. Peck; individualCount: 1; sex: female; lifeStage: adult; **Taxon:** scientificName: Brachyopacaesariata Moran and Skevington; kingdom: Animalia; phylum: Arthropoda; class: Hexapoda; order: Diptera; family: Syrphidae; genus: Brachyopa; specificEpithet: caesariata; taxonRank: species; scientificNameAuthorship: Moran and Skevington; vernacularName: Plain-winged Sapeater; nomenclaturalStatus: new species; **Location:** country: U.S.A.; stateProvince: Alaska; locality: 11 Miles South Anderson Junction, Mile 270 Rte 3; decimalLatitude: 64.165328; decimalLongitude: -149.287278; **Identification:** identifiedBy: K. Moran; **Event:** samplingProtocol: Malaise trap; eventDate: 1984-06/08-23/11; startDayOfYear: 174; endDayOfYear: 224; year: 1984; month: 6; day: 23; verbatimEventDate: 23.vi.-11.viii.1984; habitat: Alnus Populus-Picea; **Record Level:** language: en; institutionCode: CNC; basisOfRecord: PreservedSpecimen
- **Type status:** Paratype. **Occurrence:** catalogNumber: CNC_Diptera243273; recordedBy: B.J. Sinclair; individualCount: 1; sex: male; lifeStage: adult; **Taxon:** scientificName: Brachyopacaesariata Moran and Skevington; kingdom: Animalia; phylum: Arthropoda; class: Hexapoda; order: Diptera; family: Syrphidae; genus: Brachyopa; specificEpithet: caesariata; taxonRank: species; scientificNameAuthorship: Moran and Skevington; vernacularName: Plain-winged Sapeater; nomenclaturalStatus: new species; **Location:** country: Canada; stateProvince: Ontario; locality: Ottawa, Marlborough Forest Rideau Trail; decimalLatitude: 45.05; decimalLongitude: -75.816667; **Identification:** identifiedBy: K. Moran; **Event:** eventDate: 05/25/2011; year: 2011; month: 5; day: 25; verbatimEventDate: 25.v.2011; habitat: near Rogers Pond; **Record Level:** language: en; institutionCode: CNC; basisOfRecord: PreservedSpecimen
- **Type status:** Paratype. **Occurrence:** catalogNumber: CNC_Diptera37403; recordedBy: M.B. Dunn; individualCount: 1; sex: male; lifeStage: adult; **Taxon:** scientificName: Brachyopacaesariata Moran and Skevington; kingdom: Animalia; phylum: Arthropoda; class: Hexapoda; order: Diptera; family: Syrphidae; genus: Brachyopa; specificEpithet: caesariata; taxonRank: species; scientificNameAuthorship: Moran and Skevington; vernacularName: Plain-winged Sapeater; nomenclaturalStatus: new species; **Location:** country: Canada; stateProvince: Quebec; locality: Laniel; decimalLatitude: 47.033333; decimalLongitude: -79.266667; **Identification:** identifiedBy: K. Moran; **Event:** eventDate: 06/10/1931; year: 1931; month: 6; day: 10; verbatimEventDate: 10.vi.1931; **Record Level:** language: en; institutionCode: CNC; basisOfRecord: PreservedSpecimen
- **Type status:** Paratype. **Occurrence:** catalogNumber: CNC_Diptera37420; recordedBy: J.F. McAlpine; individualCount: 1; sex: female; lifeStage: adult; **Taxon:** scientificName: Brachyopacaesariata Moran and Skevington; kingdom: Animalia; phylum: Arthropoda; class: Hexapoda; order: Diptera; family: Syrphidae; genus: Brachyopa; specificEpithet: caesariata; taxonRank: species; scientificNameAuthorship: Moran and Skevington; vernacularName: Plain-winged Sapeater; nomenclaturalStatus: new species; **Location:** country: Canada; stateProvince: Ontario; locality: Maynooth; decimalLatitude: 45.233333; decimalLongitude: -77.95; **Identification:** identifiedBy: K. Moran; **Event:** eventDate: 06/18/1953; year: 1953; month: 6; day: 18; verbatimEventDate: 18.vi.1953; **Record Level:** language: en; institutionCode: CNC; basisOfRecord: PreservedSpecimen
- **Type status:** Paratype. **Occurrence:** catalogNumber: CNC_Diptera37461; recordedBy: J.F. McAlpine; individualCount: 1; sex: female; lifeStage: adult; **Taxon:** scientificName: Brachyopacaesariata Moran and Skevington; kingdom: Animalia; phylum: Arthropoda; class: Hexapoda; order: Diptera; family: Syrphidae; genus: Brachyopa; specificEpithet: caesariata; taxonRank: species; scientificNameAuthorship: Moran and Skevington; vernacularName: Plain-winged Sapeater; nomenclaturalStatus: new species; **Location:** country: Canada; stateProvince: Quebec; locality: Duncan Lake, near Rupert; decimalLatitude: 45.681389; decimalLongitude: -76.050278; **Identification:** identifiedBy: K. Moran; **Event:** eventDate: 06/14/1969; year: 1969; month: 6; day: 14; verbatimEventDate: 14.vi.1969; **Record Level:** language: en; institutionCode: CNC; basisOfRecord: PreservedSpecimen
- **Type status:** Paratype. **Occurrence:** catalogNumber: CNC_Diptera37465; recordedBy: R.E. Leech; individualCount: 1; sex: female; lifeStage: adult; **Taxon:** scientificName: Brachyopacaesariata Moran and Skevington; kingdom: Animalia; phylum: Arthropoda; class: Hexapoda; order: Diptera; family: Syrphidae; genus: Brachyopa; specificEpithet: caesariata; taxonRank: species; scientificNameAuthorship: Moran and Skevington; vernacularName: Plain-winged Sapeater; nomenclaturalStatus: new species; **Location:** country: Canada; stateProvince: British Columbia; locality: Liard Hot Springs, Mi. 496 Alaska Hwy; minimumElevationInMeters: 457; decimalLatitude: 59.425992; decimalLongitude: -126.0964; **Identification:** identifiedBy: K. Moran; **Event:** eventDate: 07/09/1959; year: 1959; month: 7; day: 9; verbatimEventDate: 9.vii.1959; **Record Level:** language: en; institutionCode: CNC; basisOfRecord: PreservedSpecimen
- **Type status:** Paratype. **Occurrence:** catalogNumber: CNC_Diptera37547; recordedBy: R.E. Leech; individualCount: 1; sex: male; lifeStage: adult; **Taxon:** scientificName: Brachyopacaesariata Moran and Skevington; kingdom: Animalia; phylum: Arthropoda; class: Hexapoda; order: Diptera; family: Syrphidae; genus: Brachyopa; specificEpithet: caesariata; taxonRank: species; scientificNameAuthorship: Moran and Skevington; vernacularName: Plain-winged Sapeater; nomenclaturalStatus: new species; **Location:** country: Canada; stateProvince: British Columbia; locality: Liard Hot Springs, Mi. 496 Alaska Hwy; minimumElevationInMeters: 457; decimalLatitude: 59.425992; decimalLongitude: -126.0964; **Identification:** identifiedBy: K. Moran; **Event:** eventDate: 07/09/1959; year: 1959; month: 7; day: 9; verbatimEventDate: 9.vii.1959; **Record Level:** language: en; institutionCode: CNC; basisOfRecord: PreservedSpecimen
- **Type status:** Paratype. **Occurrence:** catalogNumber: CNC_Diptera37548; recordedBy: J.R. Vockeroth; individualCount: 1; sex: male; lifeStage: adult; **Taxon:** scientificName: Brachyopacaesariata Moran and Skevington; kingdom: Animalia; phylum: Arthropoda; class: Hexapoda; order: Diptera; family: Syrphidae; genus: Brachyopa; specificEpithet: caesariata; taxonRank: species; scientificNameAuthorship: Moran and Skevington; vernacularName: Plain-winged Sapeater; nomenclaturalStatus: new species; **Location:** country: Canada; stateProvince: Alberta; locality: Edmonton; decimalLatitude: 53.540942; decimalLongitude: -113.4937; **Identification:** identifiedBy: K. Moran; **Event:** eventDate: 06/15/1948; year: 1948; month: 6; day: 15; verbatimEventDate: 15.vi.1948; **Record Level:** language: en; institutionCode: CNC; basisOfRecord: PreservedSpecimen
- **Type status:** Paratype. **Occurrence:** catalogNumber: CNC_Diptera37549; recordedBy: C.H. Curran; individualCount: 1; sex: male; lifeStage: adult; **Taxon:** scientificName: Brachyopacaesariata Moran and Skevington; kingdom: Animalia; phylum: Arthropoda; class: Hexapoda; order: Diptera; family: Syrphidae; genus: Brachyopa; specificEpithet: caesariata; taxonRank: species; scientificNameAuthorship: Moran and Skevington; vernacularName: Plain-winged Sapeater; nomenclaturalStatus: new species; **Location:** country: Canada; stateProvince: Ontario; locality: Mer Bleue Ottawa; decimalLatitude: 45.4; decimalLongitude: -75.5; **Identification:** identifiedBy: K. Moran; **Event:** eventDate: 05/31/1923; year: 1923; month: 5; day: 31; verbatimEventDate: 31.v.1923; **Record Level:** language: en; institutionCode: CNC; basisOfRecord: PreservedSpecimen
- **Type status:** Paratype. **Occurrence:** catalogNumber: CNC_Diptera37550; recordedBy: C.H. Curran; individualCount: 1; sex: male; lifeStage: adult; **Taxon:** scientificName: Brachyopacaesariata Moran and Skevington; kingdom: Animalia; phylum: Arthropoda; class: Hexapoda; order: Diptera; family: Syrphidae; genus: Brachyopa; specificEpithet: caesariata; taxonRank: species; scientificNameAuthorship: Moran and Skevington; vernacularName: Plain-winged Sapeater; nomenclaturalStatus: new species; **Location:** country: Canada; stateProvince: Ontario; locality: Ottawa; decimalLatitude: 45.423; decimalLongitude: -75.698; **Identification:** identifiedBy: K. Moran; **Event:** eventDate: 06/02/1927; year: 1927; month: 6; day: 2; verbatimEventDate: 2.vi.1927; **Record Level:** language: en; institutionCode: CNC; basisOfRecord: PreservedSpecimen
- **Type status:** Paratype. **Occurrence:** catalogNumber: CNC_Diptera37551; recordedBy: J.M. Cumming; individualCount: 1; sex: male; lifeStage: adult; **Taxon:** scientificName: Brachyopacaesariata Moran and Skevington; kingdom: Animalia; phylum: Arthropoda; class: Hexapoda; order: Diptera; family: Syrphidae; genus: Brachyopa; specificEpithet: caesariata; taxonRank: species; scientificNameAuthorship: Moran and Skevington; vernacularName: Plain-winged Sapeater; nomenclaturalStatus: new species; **Location:** country: Canada; stateProvince: Ontario; locality: Ottawa nr. Uplands Airport; decimalLatitude: 45.333333; decimalLongitude: -75.583333; **Identification:** identifiedBy: K. Moran; **Event:** eventDate: 05/29/1990; year: 1990; month: 5; day: 29; verbatimEventDate: 29.v.1990; **Record Level:** language: en; institutionCode: CNC; basisOfRecord: PreservedSpecimen
- **Type status:** Paratype. **Occurrence:** catalogNumber: CNC_Diptera37555; recordedBy: R.D. Bird; individualCount: 1; sex: male; lifeStage: adult; **Taxon:** scientificName: Brachyopacaesariata Moran and Skevington; kingdom: Animalia; phylum: Arthropoda; class: Hexapoda; order: Diptera; family: Syrphidae; genus: Brachyopa; specificEpithet: caesariata; taxonRank: species; scientificNameAuthorship: Moran and Skevington; vernacularName: Plain-winged Sapeater; nomenclaturalStatus: new species; **Location:** country: Canada; stateProvince: Manitoba; locality: Aweme; decimalLatitude: 49.708531; decimalLongitude: -99.602758; **Identification:** identifiedBy: K. Moran; **Event:** eventDate: 06/09/1925; year: 1925; month: 6; day: 9; verbatimEventDate: 9.vi.1925; **Record Level:** language: en; institutionCode: CNC; basisOfRecord: PreservedSpecimen
- **Type status:** Paratype. **Occurrence:** catalogNumber: CNC_Diptera37556; individualCount: 1; sex: male; lifeStage: adult; **Taxon:** scientificName: Brachyopacaesariata Moran and Skevington; kingdom: Animalia; phylum: Arthropoda; class: Hexapoda; order: Diptera; family: Syrphidae; genus: Brachyopa; specificEpithet: caesariata; taxonRank: species; scientificNameAuthorship: Moran and Skevington; vernacularName: Plain-winged Sapeater; nomenclaturalStatus: new species; **Location:** country: Canada; stateProvince: Nova Scotia; locality: Kentville; decimalLatitude: 45.066667; decimalLongitude: -64.483333; **Identification:** identifiedBy: K. Moran; **Event:** eventDate: 06/09/1915; year: 1915; month: 6; day: 9; verbatimEventDate: 9.vi.1915; **Record Level:** language: en; institutionCode: CNC; basisOfRecord: PreservedSpecimen
- **Type status:** Paratype. **Occurrence:** catalogNumber: CNC_Diptera37559; recordedBy: E.D.A. Dyer; individualCount: 1; sex: male; lifeStage: adult; **Taxon:** scientificName: Brachyopacaesariata Moran and Skevington; kingdom: Animalia; phylum: Arthropoda; class: Hexapoda; order: Diptera; family: Syrphidae; genus: Brachyopa; specificEpithet: caesariata; taxonRank: species; scientificNameAuthorship: Moran and Skevington; vernacularName: Plain-winged Sapeater; nomenclaturalStatus: new species; **Location:** country: Canada; stateProvince: British Columbia; locality: Hixon; decimalLatitude: 53.420261; decimalLongitude: -122.585961; **Identification:** identifiedBy: K. Moran; **Event:** eventDate: 06/07/1966; year: 1966; month: 6; day: 7; verbatimEventDate: 7.vi.1966; **Record Level:** language: en; institutionCode: CNC; basisOfRecord: PreservedSpecimen
- **Type status:** Paratype. **Occurrence:** catalogNumber: CNC_Diptera37560; recordedBy: E.D.A. Dyer; individualCount: 1; sex: male; lifeStage: adult; **Taxon:** scientificName: Brachyopacaesariata Moran and Skevington; kingdom: Animalia; phylum: Arthropoda; class: Hexapoda; order: Diptera; family: Syrphidae; genus: Brachyopa; specificEpithet: caesariata; taxonRank: species; scientificNameAuthorship: Moran and Skevington; vernacularName: Plain-winged Sapeater; nomenclaturalStatus: new species; **Location:** country: Canada; stateProvince: British Columbia; locality: Hixon; decimalLatitude: 53.420261; decimalLongitude: -122.585961; **Identification:** identifiedBy: K. Moran; **Event:** eventDate: 06/23/1966; year: 1966; month: 6; day: 23; verbatimEventDate: 23.vi.1966; **Record Level:** language: en; institutionCode: CNC; basisOfRecord: PreservedSpecimen
- **Type status:** Paratype. **Occurrence:** catalogNumber: CNC_Diptera37561; recordedBy: S. & J. Peck; individualCount: 1; sex: male; lifeStage: adult; **Taxon:** scientificName: Brachyopacaesariata Moran and Skevington; kingdom: Animalia; phylum: Arthropoda; class: Hexapoda; order: Diptera; family: Syrphidae; genus: Brachyopa; specificEpithet: caesariata; taxonRank: species; scientificNameAuthorship: Moran and Skevington; vernacularName: Plain-winged Sapeater; nomenclaturalStatus: new species; **Location:** country: U.S.A.; stateProvince: Alaska; locality: Rte. 3, mi. 270 11 mi. S Anderson; decimalLatitude: 64.165328; decimalLongitude: -149.287278; **Identification:** identifiedBy: K. Moran; **Event:** samplingProtocol: Malaise trap; eventDate: 1984-06/08-22/11; startDayOfYear: 173; endDayOfYear: 224; year: 1984; month: 6; day: 22; verbatimEventDate: 22.vi.-11.viii.1984; habitat: Alder-Poplar-Spruce; **Record Level:** language: en; institutionCode: CNC; basisOfRecord: PreservedSpecimen
- **Type status:** Paratype. **Occurrence:** catalogNumber: CNC_Diptera37566; recordedBy: D.M. Wood; individualCount: 1; sex: male; lifeStage: adult; **Taxon:** scientificName: Brachyopacaesariata Moran and Skevington; kingdom: Animalia; phylum: Arthropoda; class: Hexapoda; order: Diptera; family: Syrphidae; genus: Brachyopa; specificEpithet: caesariata; taxonRank: species; scientificNameAuthorship: Moran and Skevington; vernacularName: Plain-winged Sapeater; nomenclaturalStatus: new species; **Location:** country: Canada; stateProvince: Ontario; locality: St. Williams; Norfolk; decimalLatitude: 42.666667; decimalLongitude: -80.4; **Identification:** identifiedBy: K. Moran; **Event:** eventDate: 05/19/1970; year: 1970; month: 5; day: 19; verbatimEventDate: 19.v.1970; **Record Level:** language: en; institutionCode: CNC; basisOfRecord: PreservedSpecimen
- **Type status:** Paratype. **Occurrence:** catalogNumber: CNC_Diptera37580; recordedBy: A.R. Brooks; individualCount: 1; sex: female; lifeStage: adult; **Taxon:** scientificName: Brachyopacaesariata Moran and Skevington; kingdom: Animalia; phylum: Arthropoda; class: Hexapoda; order: Diptera; family: Syrphidae; genus: Brachyopa; specificEpithet: caesariata; taxonRank: species; scientificNameAuthorship: Moran and Skevington; vernacularName: Plain-winged Sapeater; nomenclaturalStatus: new species; **Location:** country: Canada; stateProvince: Alberta; locality: Vallyview; decimalLatitude: 55.068678; decimalLongitude: -117.269575; **Identification:** identifiedBy: K. Moran; **Event:** eventDate: 06/04/1961; year: 1961; month: 6; day: 4; verbatimEventDate: 4.vi.1961; **Record Level:** language: en; institutionCode: CNC; basisOfRecord: PreservedSpecimen
- **Type status:** Paratype. **Occurrence:** catalogNumber: CNC_Diptera37581; recordedBy: J.R. Vockeroth; individualCount: 1; sex: female; lifeStage: adult; **Taxon:** scientificName: Brachyopacaesariata Moran and Skevington; kingdom: Animalia; phylum: Arthropoda; class: Hexapoda; order: Diptera; family: Syrphidae; genus: Brachyopa; specificEpithet: caesariata; taxonRank: species; scientificNameAuthorship: Moran and Skevington; vernacularName: Plain-winged Sapeater; nomenclaturalStatus: new species; **Location:** country: Canada; stateProvince: Ontario; locality: Constance L. South March; decimalLatitude: 45.401397; decimalLongitude: -75.979717; **Identification:** identifiedBy: K. Moran; **Event:** eventDate: 06/16/1965; year: 1965; month: 6; day: 16; verbatimEventDate: 16.vi.1965; **Record Level:** language: en; institutionCode: CNC; basisOfRecord: PreservedSpecimen
- **Type status:** Paratype. **Occurrence:** catalogNumber: CNC_Diptera37582; recordedBy: S.M. Clark; individualCount: 1; sex: female; lifeStage: adult; **Taxon:** scientificName: Brachyopacaesariata Moran and Skevington; kingdom: Animalia; phylum: Arthropoda; class: Hexapoda; order: Diptera; family: Syrphidae; genus: Brachyopa; specificEpithet: caesariata; taxonRank: species; scientificNameAuthorship: Moran and Skevington; vernacularName: Plain-winged Sapeater; nomenclaturalStatus: new species; **Location:** country: Canada; stateProvince: Ontario; locality: One Sided Lake; decimalLatitude: 49.05; decimalLongitude: -93.883333; **Identification:** identifiedBy: K. Moran; **Event:** eventDate: 06/20/1960; year: 1960; month: 6; day: 20; verbatimEventDate: 20.vi.1960; **Record Level:** language: en; institutionCode: CNC; basisOfRecord: PreservedSpecimen
- **Type status:** Paratype. **Occurrence:** catalogNumber: CNC_Diptera37583; recordedBy: J.G. Chillcott; individualCount: 1; sex: female; lifeStage: adult; **Taxon:** scientificName: Brachyopacaesariata Moran and Skevington; kingdom: Animalia; phylum: Arthropoda; class: Hexapoda; order: Diptera; family: Syrphidae; genus: Brachyopa; specificEpithet: caesariata; taxonRank: species; scientificNameAuthorship: Moran and Skevington; vernacularName: Plain-winged Sapeater; nomenclaturalStatus: new species; **Location:** country: Canada; stateProvince: Ontario; locality: Port Severn, 3 mi[les] N.; decimalLatitude: 44.8; decimalLongitude: -79.716667; **Identification:** identifiedBy: K. Moran; **Event:** eventDate: 05/27/1959; year: 1959; month: 5; day: 27; verbatimEventDate: 27.v.1959; habitat: black spruce bog; **Record Level:** language: en; institutionCode: CNC; basisOfRecord: PreservedSpecimen
- **Type status:** Paratype. **Occurrence:** catalogNumber: CNC_Diptera37585; individualCount: 1; sex: female; lifeStage: adult; **Taxon:** scientificName: Brachyopacaesariata Moran and Skevington; kingdom: Animalia; phylum: Arthropoda; class: Hexapoda; order: Diptera; family: Syrphidae; genus: Brachyopa; specificEpithet: caesariata; taxonRank: species; scientificNameAuthorship: Moran and Skevington; vernacularName: Plain-winged Sapeater; nomenclaturalStatus: new species; **Location:** country: U.S.A.; stateProvince: Maryland; locality: Laurel; decimalLatitude: 39.083333; decimalLongitude: -76.833333; **Identification:** identifiedBy: K. Moran; **Event:** samplingProtocol: Malaise trap; eventDate: 05/25/1965; year: 1965; month: 5; day: 25; verbatimEventDate: 25.v.1965; **Record Level:** language: en; institutionCode: CNC; basisOfRecord: PreservedSpecimen
- **Type status:** Paratype. **Occurrence:** catalogNumber: CNC_Diptera60558; recordedBy: S. & J. Peck; individualCount: 1; sex: male; lifeStage: adult; **Taxon:** scientificName: Brachyopacaesariata Moran and Skevington; kingdom: Animalia; phylum: Arthropoda; class: Hexapoda; order: Diptera; family: Syrphidae; genus: Brachyopa; specificEpithet: caesariata; taxonRank: species; scientificNameAuthorship: Moran and Skevington; vernacularName: Plain-winged Sapeater; nomenclaturalStatus: new species; **Location:** country: Canada; stateProvince: British Columbia; locality: 14 km East of Coal River; decimalLatitude: 59.649875; decimalLongitude: -126.683892; **Identification:** identifiedBy: K. Moran; **Event:** eventDate: 1984-06/09-14/03; startDayOfYear: 166; endDayOfYear: 247; year: 1984; month: 6; day: 14; verbatimEventDate: 14.vi.-3.ix.1984; habitat: Picea-Alnus forest; **Record Level:** language: en; institutionCode: CNC; basisOfRecord: PreservedSpecimen
- **Type status:** Paratype. **Occurrence:** catalogNumber: CNC_Diptera60559; recordedBy: S. & J. Peck; individualCount: 1; sex: female; lifeStage: adult; **Taxon:** scientificName: Brachyopacaesariata Moran and Skevington; kingdom: Animalia; phylum: Arthropoda; class: Hexapoda; order: Diptera; family: Syrphidae; genus: Brachyopa; specificEpithet: caesariata; taxonRank: species; scientificNameAuthorship: Moran and Skevington; vernacularName: Plain-winged Sapeater; nomenclaturalStatus: new species; **Location:** country: Canada; stateProvince: British Columbia; locality: 14 km East of Coal River; decimalLatitude: 59.649875; decimalLongitude: -126.683892; **Identification:** identifiedBy: K. Moran; **Event:** eventDate: 1984-06/09-14/03; startDayOfYear: 166; endDayOfYear: 247; year: 1984; month: 6; day: 14; verbatimEventDate: 14.vi.-3.ix.1984; habitat: Picea-Alnus forest; **Record Level:** language: en; institutionCode: CNC; basisOfRecord: PreservedSpecimen
- **Type status:** Paratype. **Occurrence:** catalogNumber: CNC_Diptera94649; recordedBy: J.R. Vockeroth; individualCount: 1; sex: female; lifeStage: adult; **Taxon:** scientificName: Brachyopacaesariata Moran and Skevington; kingdom: Animalia; phylum: Arthropoda; class: Hexapoda; order: Diptera; family: Syrphidae; genus: Brachyopa; specificEpithet: caesariata; taxonRank: species; scientificNameAuthorship: Moran and Skevington; vernacularName: Plain-winged Sapeater; nomenclaturalStatus: new species; **Location:** country: U.S.A.; stateProvince: New Hampshire; locality: Lancaster, Mount Prospect; minimumElevationInMeters: 625; decimalLatitude: 44.449317; decimalLongitude: -71.570889; **Identification:** identifiedBy: K. Moran; **Event:** eventDate: 06/19/1982; year: 1982; month: 6; day: 19; verbatimEventDate: 19.vi.1982; **Record Level:** language: en; institutionCode: CNC; basisOfRecord: PreservedSpecimen
- **Type status:** Paratype. **Occurrence:** catalogNumber: CNC_Diptera95516; recordedBy: B. Gallaway; individualCount: 1; sex: female; lifeStage: adult; **Taxon:** scientificName: Brachyopacaesariata Moran and Skevington; kingdom: Animalia; phylum: Arthropoda; class: Hexapoda; order: Diptera; family: Syrphidae; genus: Brachyopa; specificEpithet: caesariata; taxonRank: species; scientificNameAuthorship: Moran and Skevington; vernacularName: Plain-winged Sapeater; nomenclaturalStatus: new species; **Location:** country: Canada; stateProvince: Manitoba; locality: 2 miles North East of Treebank along Souris River; decimalLatitude: 49.666667; decimalLongitude: -99.633333; **Identification:** identifiedBy: K. Moran; **Event:** eventDate: 06/09/1993; year: 1993; month: 6; day: 9; verbatimEventDate: 9.vi.1993; **Record Level:** language: en; institutionCode: CNC; basisOfRecord: PreservedSpecimen
- **Type status:** Paratype. **Occurrence:** catalogNumber: CNC_Diptera95517; recordedBy: B. Gallaway; individualCount: 1; sex: female; lifeStage: adult; **Taxon:** scientificName: Brachyopacaesariata Moran and Skevington; kingdom: Animalia; phylum: Arthropoda; class: Hexapoda; order: Diptera; family: Syrphidae; genus: Brachyopa; specificEpithet: caesariata; taxonRank: species; scientificNameAuthorship: Moran and Skevington; vernacularName: Plain-winged Sapeater; nomenclaturalStatus: new species; **Location:** country: Canada; stateProvince: Manitoba; locality: 2 miles North East of Treebank along Souris River; decimalLatitude: 49.666667; decimalLongitude: -99.633333; **Identification:** identifiedBy: K. Moran; **Event:** eventDate: 06/09/1993; year: 1993; month: 6; day: 9; verbatimEventDate: 9.vi.1993; **Record Level:** language: en; institutionCode: CNC; basisOfRecord: PreservedSpecimen
- **Type status:** Paratype. **Occurrence:** catalogNumber: CNC_Diptera96849; recordedBy: J.R. Vockeroth; individualCount: 1; sex: male; lifeStage: adult; **Taxon:** scientificName: Brachyopacaesariata Moran and Skevington; kingdom: Animalia; phylum: Arthropoda; class: Hexapoda; order: Diptera; family: Syrphidae; genus: Brachyopa; specificEpithet: caesariata; taxonRank: species; scientificNameAuthorship: Moran and Skevington; vernacularName: Plain-winged Sapeater; nomenclaturalStatus: new species; **Location:** country: Canada; stateProvince: Ontario; locality: Orleans, Chapel Hill; decimalLatitude: 45.466667; decimalLongitude: -75.516667; **Identification:** identifiedBy: K. Moran; **Event:** samplingProtocol: sweeping; eventDate: 06/21/1995; year: 1995; month: 6; day: 21; verbatimEventDate: 21.vi.1995; **Record Level:** language: en; institutionCode: CNC; basisOfRecord: PreservedSpecimen

#### Description

**Size**: Body length 5.2 to 7.9 mm; wing length 4.7 to 6.2 mm

**Male: Head**: Yellow to orange; face concave, covered in white pollen and bare, lower face projecting forwards along the oral margin; gena pale pilose, shiny anteriorly and gold pollinose posteriorly; frontal triangle shiny, bare except for gold pollinosity narrowly along eye margin; vertex yellow, bare and gold pollinose, except ocellar triangle pale pilose; occiput gold pollinose, pale pilose with some short black setae on dorsal ¼; antenna wholly yellow to orange, scape and pedicel with short, pale pile, pedicel about as long as scape, postpedicel oval, about as long as wide, with distinct sensory pit on inner surface, arista pubescent but not plumose; eye bare, broadly holoptic.

**Thorax**: Mesonotum yellow to orange, pale, black or mixed pale and black pilose and gold pollinose except for two medial, bare stripes starting at anterior edge and running about three-fourths the length of mesonotum and four bare vittae, two before transverse suture and two after; scutellum yellow to orange, pale, black or mixed pale and black pilose, with macrosetae; subscutellar fringe absent; postpronotum yellow to orange, pale pilose and white pollinose; postalar callus yellow, pale pilose and shiny; pleuron yellow, sparsely white pollinose; anterior anepisternum, katepimeron and meron bare; posterior anepisternum and anepimeron pale pilose; katepisternum bare on dorsal half; metasternum yellow, sparsely white pollinose, bare; legs yellow, except 4th and 5th pro- and mesotarsomeres, as well as metatibia and tarsomeres black; meso- and metacoxae sparsely white pollinose; femora pale pilose except meso- and metafemur with black setae ventrally; tibia and tarsi mixed pale and black pilose; wing entirely densely microtrichose; halter yellow; calypter white.

**Abdomen**: Oval, yellow to brown, pale pilose except tergites 2-4 black pilose medially, shiny.

**Genitalia**: Epandrium subquadrate, longer than wide, narrows slightly dorsally; cercus with laterally compressed, oval sclerotised outer portion covered in long pile and membranous inner portion; surstylus short, with well separated dorsal and ventral lobes, dorsal lobe short, ventrally compressed, ventral lobe large, laterally compressed, rounded and elongate, dorsal lobe densely setose on posterior half of inner surface and the posterior rim of the outer surface, ventral lobe with sparse row of pile on posterior rim; subepandrial sclerite divided with one arm off of each surstylus, joined only where it articulates with hypandrium; ejaculatory apodeme tiny, asymmetrical with distal end slightly broadened; sperm pump inside the proximal end of the hyprandrium; hypandrium narrow and elongated, rounded basally, ventrally with grooves mediobasally; postgonite rectangular; phallus stubby and short.

**Female**: Similar to male except completely golden pollinose on frons between eyes in addition to normal sexual dimorphism.

See Fig. 5.

#### Diagnosis

Katepisternum usually bare on dorsal half, rarely with 1-2 hairs. Mesonotum yellow in colour. Never with wing spots. Arista pubescent. Abdomen and legs extensively yellow.

#### Etymology

The name is from the Latin *caesariatus*, meaning covered with hair, with reference to the pilose arista.

#### Distribution

This species is found from Nova Scotia to British Columbia, north to Alaska and south to Utah in the west and Maryland in the east.

#### Ecology

This uncommon species can be found flying from mid-May to late July but may be found as early as the beginning of March in the southwest. Specimens have been collected from *Heracleum* and *Rubus* plants and from black spruce bog and Alder-Poplar-Spruce forest habitats.

#### Taxon discussion

This species is part of the *B.punctipennis* Curran 1925 complex of five species. Skevington et al. (2019) incorrectly states that the name for this group is the *B.perplexa* Curran 1922 complex. *Brachyopapunctipennis* *s.s.* is restricted to the Western US as are three additional undescribed species in the complex. Like other species in this complex, *Brachyopacaesariata* is found in the Western US; however it is the only species whose range also extends to the Eastern US. Data for three of the five undescribed species in this complex are available in BOLD. *Brachyopapunctipennis* *s.s.* has not yet been sequenced. DNA sequences available for *B.caesariata* are listed in (Table 2).

#### Common Name

The common name given to the species by Skevington et al. (2019) is Plain-winged Sapeater.

### Brachyopa cummingi

Moran and Skevington sp. n.

2D8349D5F70C5F3BACD523A94BFB2670

urn:lsid:zoobank.org:act:FE5C29DE-53A5-4CAD-B31A-039112D8DE28

#### Materials

- **Type status:** Holotype. **Occurrence:** catalogNumber: CNC_Diptera106864; recordedBy: J.M. Cumming; individualCount: 1; sex: male; lifeStage: adult; **Taxon:** scientificName: Brachyopacummingi Moran and Skevington; kingdom: Animalia; phylum: Arthropoda; class: Hexapoda; order: Diptera; family: Syrphidae; genus: Brachyopa; specificEpithet: cummingi; taxonRank: species; scientificNameAuthorship: Moran and Skevington 2019; vernacularName: Somber Sapeater; nomenclaturalStatus: new species; **Location:** country: Canada; stateProvince: Ontario; locality: Ottawa; decimalLatitude: 45.266667; decimalLongitude: -75.75; **Identification:** identifiedBy: K. Moran; **Event:** samplingProtocol: hand collected; eventDate: 1986-05-14; year: 1986; month: 5; day: 14; verbatimEventDate: 14.v.1986; **Record Level:** language: en; institutionCode: CNC; basisOfRecord: PreservedSpecimen

#### Description

**Size**: Body length 5.8 mm; wing length 5.4 mm

**Male: Head**: Yellow to orange; face concave, covered in white pollen and bare, lower face projecting forwards along the oral margin; gena pale pilose, shiny anteriorly and white pollinose posteriorly; frontal triangle shiny, bare except for white pollinosity and short pale pile narrowly along eye margin; vertex yellow, except ocellar triangle black, white pollinose and pale pilose; occiput white pollinose, pale pilose with some short black setae on dorsal ¼; antenna wholly yellow to orange, scape and pedicel with short, pale pile, pedicel about as long as scape, postpedicel oval, about twice as long as wide, with minute sensory pit on inner surface, arista minutely pubescent; eye bare, very narrowly dichoptic.

**Thorax**: Mesonotum dull brown, pale pilose, white pollinose except for two medial, bare stripes starting at anterior edge and running about three-fourths the length of mesonotum and four bare vittae, two before transverse suture and two after; scutellum yellow, pale pilose, sparsely white pollinose and without macrosetae; subscutellar fringe absent; postpronotum yellow, pale pilose and white pollinose; postalar callus yellow, pale pilose and white pollinose; pleuron yellow, covered in white pollen; anterior anepisternum, katepimeron and meron bare; posterior anepisternum and anepimeron pale pilose; katepisternum discontinuously pale pilose; metasternum yellow, white pollinose, bare; legs yellow, pale pilose; meso- and metacoxa sparsely white pollinose; metafemur with black setulae along ventral side; wing entirely densely microtrichose; halter yellow; calypter white.

**Abdomen**: Oval, unicolorous light brown, pale pilose, sparsely white pollinose.

**Genitalia**: Epandrium subquadrate, longer than wide, narrows slightly dorsally; cercus with laterally compressed, subtriangular sclerotised outer portion covered in long pile and membranous inner portion; surstylus short, with broadly fused, for ⅓ the length of the ventral lobe, dorsal and ventral lobes, dorsal lobe short, rounded apically, ventral lobe large and elongate rectangularly, dorsal lobe densely setose on apex and with dense cluster of long pile on outer surface just prior to apex, ventral lobe with sparse smattering of pile on outer surface; subepandrial sclerite divided with one arm off of each surstylus, joined only where it articulates with hypandrium; ejaculatory apodeme narrow, straight; phallapodeme long and narrow; postgonite hooked ventrally, adjacent to hypandrium; dorsal hypandrium with paired, pointed, long arms projecting from distal end, with triangular tooth near tip; phallus simple tube within hypandrium.

**Female**: Unknown.

See Fig. 6.

#### Diagnosis

Arista pubescent but not plumose. Male postpedicel with distinct sensory pit. White pollinose on scutum. Postpronotum and scutellum yellow in colour. Wing hyaline. Katepisternum pilose on dorsal half. Scutellum pale pilose; completely pollinose. Abdomen unicolorous light brown; completely pollinose.

#### Etymology

This species is named for the collector of the only known specimen. Jeff Cumming works at the Canadian National Collection of Insects, Arachnids and Nematodes where he specialises on Empidoidea systematics.

#### Distribution

The only known specimen of this species was collected in Ottawa, Ontario, Canada.

#### Ecology

This extremely rare fly has been collected once in mid-May (14 May 1986).

#### Taxon discussion

We were unable to sequence this specimen. Morphologically, it appears to be closely related to *B.daeckei* Johnson 1917. *Brachyopadaeckei* lacks the distinct sensory pit on the postpedicel that is present in *B.cummingi*. Additionally, *B.daeckei* has dark brown instead of pale yellow postpronotum and scutelllum, gold instead of white pollinosity on scutum and a pitch black instead of pale brown abdomen. Genitalia of *B.cummingi* and *B.daeckei* are similar, except surstylar lobes of *B.daeckei* are fused over ½ the width of ventral lobe in comparison to *B.cummingi* in which they are fused for ⅓ the width.

#### Common Name

The common name given to the species by Skevington et al. (2019) is Somber Sapeater.

### Hammerschmidtia sedmani

Vockeroth, Moran and Skevington sp. n.

0CC1C98EC5C85D6DBB00CE7B0D39A952

urn:lsid:zoobank.org:act:64C74904-E2A0-4EC1-8E42-0A18942BBA7C

#### Materials

- **Type status:** Holotype. **Occurrence:** catalogNumber: CNC298236; recordedBy: J.H. Skevington; individualCount: 1; sex: male; lifeStage: adult; **Taxon:** scientificName: Hammerschmidtiasedmani Vockeroth, Moran and Skevington; kingdom: Animalia; phylum: Arthropoda; class: Hexapoda; order: Diptera; family: Syrphidae; genus: Hammerschmidtia; specificEpithet: sedmani; taxonRank: species; scientificNameAuthorship: Vockeroth, Moran and Skevington 2019; vernacularName: Pale-bristled Logsitter; nomenclaturalStatus: new species; **Location:** country: Canada; stateProvince: Quebec; locality: Base of Mount Rigaud; decimalLatitude: 45.466261; decimalLongitude: -74.322978; **Identification:** identifiedBy: J.H. Skevington; **Event:** eventDate: 2014-06-08; year: 2014; month: 6; day: 8; verbatimEventDate: 8.vi.2014; habitat: on large fallen aspen log (on ground for about one year); **Record Level:** institutionCode: CNC; basisOfRecord: PreservedSpecimen
- **Type status:** Paratype. **Occurrence:** catalogNumber: CNC1038609; recordedBy: J.H.Skevington, M.M. Locke; individualCount: 1; lifeStage: adult; **Taxon:** scientificName: Hammerschmidtiasedmani Vockeroth, Moran and Skevington; kingdom: Animalia; phylum: Arthropoda; class: Hexapoda; order: Diptera; family: Syrphidae; genus: Hammerschmidtia; specificEpithet: sedmani; taxonRank: species; scientificNameAuthorship: Vockeroth, Moran and Skevington 2019; vernacularName: Pale-bristled Logsitter; nomenclaturalStatus: new species; **Location:** country: Canada; stateProvince: Ontario; locality: Algonquin Provincial Park, Opeongo Road; decimalLatitude: 45.625911; decimalLongitude: -78.350753; **Identification:** identifiedBy: J.H. Skevington; **Event:** eventDate: 2019-06-03; year: 2018; month: 6; day: 3; verbatimEventDate: 3.vi.2018; habitat: on flowers of Prunusvirginiana; **Record Level:** language: en; institutionCode: CNC; basisOfRecord: PreservedSpecimen
- **Type status:** Paratype. **Occurrence:** catalogNumber: CNC298237; recordedBy: J.H. Skevington; individualCount: 1; sex: male; lifeStage: adult; **Taxon:** scientificName: Hammerschmidtiasedmani Vockeroth, Moran and Skevington; kingdom: Animalia; phylum: Arthropoda; class: Hexapoda; order: Diptera; family: Syrphidae; genus: Hammerschmidtia; specificEpithet: sedmani; taxonRank: species; scientificNameAuthorship: Vockeroth, Moran and Skevington 2019; vernacularName: Pale-bristled Logsitter; nomenclaturalStatus: new species; **Location:** country: Canada; stateProvince: Quebec; locality: Base of Mount Rigaud; decimalLatitude: 45.466261; decimalLongitude: -74.322976; **Identification:** identifiedBy: J.H. Skevington; **Event:** eventDate: 2014-06-08; year: 2014; month: 6; day: 8; verbatimEventDate: 8.vi.2014; habitat: on large fallen aspen log (on ground for about one year); **Record Level:** institutionCode: CNC; basisOfRecord: PreservedSpecimen
- **Type status:** Paratype. **Occurrence:** catalogNumber: CNC298238; recordedBy: J.H. Skevington; individualCount: 1; sex: male; lifeStage: adult; **Taxon:** scientificName: Hammerschmidtiasedmani Vockeroth, Moran and Skevington; kingdom: Animalia; phylum: Arthropoda; class: Hexapoda; order: Diptera; family: Syrphidae; genus: Hammerschmidtia; specificEpithet: sedmani; taxonRank: species; scientificNameAuthorship: Vockeroth, Moran and Skevington 2019; vernacularName: Pale-bristled Logsitter; nomenclaturalStatus: new species; **Location:** country: Canada; stateProvince: Quebec; locality: Base of Mount Rigaud; decimalLatitude: 45.466261; decimalLongitude: -74.322976; **Identification:** identifiedBy: J.H. Skevington; **Event:** eventDate: 2014-06-08; year: 2014; month: 6; day: 8; verbatimEventDate: 8.vi.2014; habitat: on large fallen aspen log (on ground for about one year); **Record Level:** institutionCode: CNC; basisOfRecord: PreservedSpecimen
- **Type status:** Paratype. **Occurrence:** catalogNumber: CNC298239; recordedBy: J.H. Skevington; individualCount: 1; sex: male; lifeStage: adult; **Taxon:** scientificName: Hammerschmidtiasedmani Vockeroth, Moran and Skevington; kingdom: Animalia; phylum: Arthropoda; class: Hexapoda; order: Diptera; family: Syrphidae; genus: Hammerschmidtia; specificEpithet: sedmani; taxonRank: species; scientificNameAuthorship: Vockeroth, Moran and Skevington 2019; vernacularName: Pale-bristled Logsitter; nomenclaturalStatus: new species; **Location:** country: Canada; stateProvince: Quebec; locality: Base of Mount Rigaud; decimalLatitude: 45.466261; decimalLongitude: -74.322976; **Identification:** identifiedBy: J.H. Skevington; **Event:** eventDate: 2014-06-08; year: 2014; month: 6; day: 8; verbatimEventDate: 8.vi.2014; habitat: on large fallen aspen log (on ground for about one year); **Record Level:** institutionCode: CNC; basisOfRecord: PreservedSpecimen
- **Type status:** Paratype. **Occurrence:** catalogNumber: CNC298240; recordedBy: J.H. Skevington; individualCount: 1; sex: male; lifeStage: adult; **Taxon:** scientificName: Hammerschmidtiasedmani Vockeroth, Moran and Skevington; kingdom: Animalia; phylum: Arthropoda; class: Hexapoda; order: Diptera; family: Syrphidae; genus: Hammerschmidtia; specificEpithet: sedmani; taxonRank: species; scientificNameAuthorship: Vockeroth, Moran and Skevington 2019; vernacularName: Pale-bristled Logsitter; nomenclaturalStatus: new species; **Location:** country: Canada; stateProvince: Quebec; locality: Base of Mount Rigaud; decimalLatitude: 45.466261; decimalLongitude: -74.322976; **Identification:** identifiedBy: J.H. Skevington; **Event:** eventDate: 2014-06-08; year: 2014; month: 6; day: 8; verbatimEventDate: 8.vi.2014; habitat: on large fallen aspen log (on ground for about one year); **Record Level:** institutionCode: CNC; basisOfRecord: PreservedSpecimen
- **Type status:** Paratype. **Occurrence:** catalogNumber: CNC298241; recordedBy: J.H. Skevington; individualCount: 1; sex: male; lifeStage: adult; **Taxon:** scientificName: Hammerschmidtiasedmani Vockeroth, Moran and Skevington; kingdom: Animalia; phylum: Arthropoda; class: Hexapoda; order: Diptera; family: Syrphidae; genus: Hammerschmidtia; specificEpithet: sedmani; taxonRank: species; scientificNameAuthorship: Vockeroth, Moran and Skevington 2019; vernacularName: Pale-bristled Logsitter; nomenclaturalStatus: new species; **Location:** country: Canada; stateProvince: Quebec; locality: Base of Mount Rigaud; decimalLatitude: 45.466261; decimalLongitude: -74.322976; **Identification:** identifiedBy: J.H. Skevington; **Event:** eventDate: 2014-06-08; year: 2014; month: 6; day: 8; verbatimEventDate: 8.vi.2014; habitat: on large fallen aspen log (on ground for about one year); **Record Level:** institutionCode: CNC; basisOfRecord: PreservedSpecimen
- **Type status:** Paratype. **Occurrence:** catalogNumber: CNC298242; recordedBy: J.H. Skevington; individualCount: 1; sex: male; lifeStage: adult; **Taxon:** scientificName: Hammerschmidtiasedmani Vockeroth, Moran and Skevington; kingdom: Animalia; phylum: Arthropoda; class: Hexapoda; order: Diptera; family: Syrphidae; genus: Hammerschmidtia; specificEpithet: sedmani; taxonRank: species; scientificNameAuthorship: Vockeroth, Moran and Skevington 2019; vernacularName: Pale-bristled Logsitter; nomenclaturalStatus: new species; **Location:** country: Canada; stateProvince: Quebec; locality: Base of Mount Rigaud; decimalLatitude: 45.466261; decimalLongitude: -74.322976; **Identification:** identifiedBy: J.H. Skevington; **Event:** eventDate: 2014-06-08; year: 2014; month: 6; day: 8; verbatimEventDate: 8.vi.2014; habitat: on large fallen aspen log (on ground for about one year); **Record Level:** institutionCode: CNC; basisOfRecord: PreservedSpecimen
- **Type status:** Paratype. **Occurrence:** catalogNumber: CNC298243; recordedBy: J.H. Skevington; individualCount: 1; sex: male; lifeStage: adult; **Taxon:** scientificName: Hammerschmidtiasedmani Vockeroth, Moran and Skevington; kingdom: Animalia; phylum: Arthropoda; class: Hexapoda; order: Diptera; family: Syrphidae; genus: Hammerschmidtia; specificEpithet: sedmani; taxonRank: species; scientificNameAuthorship: Vockeroth, Moran and Skevington 2019; vernacularName: Pale-bristled Logsitter; nomenclaturalStatus: new species; **Location:** country: Canada; stateProvince: Quebec; locality: Base of Mount Rigaud; decimalLatitude: 45.466261; decimalLongitude: -74.322978; **Identification:** identifiedBy: J.H. Skevington; **Event:** eventDate: 2014-06-08; year: 2014; month: 6; day: 8; verbatimEventDate: 8.vi.2014; habitat: on large fallen aspen log (on ground for about one year); **Record Level:** institutionCode: CNC; basisOfRecord: PreservedSpecimen
- **Type status:** Paratype. **Occurrence:** catalogNumber: CNC298244; recordedBy: J.H. Skevington; individualCount: 1; sex: male; lifeStage: adult; **Taxon:** scientificName: Hammerschmidtiasedmani Vockeroth, Moran and Skevington; kingdom: Animalia; phylum: Arthropoda; class: Hexapoda; order: Diptera; family: Syrphidae; genus: Hammerschmidtia; specificEpithet: sedmani; taxonRank: species; scientificNameAuthorship: Vockeroth, Moran and Skevington 2019; vernacularName: Pale-bristled Logsitter; nomenclaturalStatus: new species; **Location:** country: Canada; stateProvince: Quebec; locality: Base of Mount Rigaud; decimalLatitude: 45.466261; decimalLongitude: -74.322978; **Identification:** identifiedBy: J.H. Skevington; **Event:** eventDate: 2014-06-08; year: 2014; month: 6; day: 8; verbatimEventDate: 8.vi.2014; habitat: on large fallen aspen log (on ground for about one year); **Record Level:** institutionCode: CNC; basisOfRecord: PreservedSpecimen
- **Type status:** Paratype. **Occurrence:** catalogNumber: CNC_Diptera111405; recordedBy: S. & J. Peck; individualCount: 1; sex: female; lifeStage: adult; **Taxon:** scientificName: Hammerschmidtiasedmani Vockeroth, Moran and Skevington; kingdom: Animalia; phylum: Arthropoda; class: Hexapoda; order: Diptera; family: Syrphidae; genus: Hammerschmidtia; specificEpithet: sedmani; taxonRank: species; scientificNameAuthorship: Vockeroth, Moran and Skevington 2019; vernacularName: Pale-bristled Logsitter; nomenclaturalStatus: new species; **Location:** country: Canada; stateProvince: Ontario; locality: Smooth Rock Falls, 56 km Northwest of Cochrane; decimalLatitude: 49.275469; decimalLongitude: -81.629928; **Identification:** identifiedBy: J.R. Vockeroth; **Event:** eventDate: 1984-06-03; year: 1984; month: 6; day: 3; verbatimEventDate: 3.vi.1984; habitat: Picea-betula forest; **Record Level:** institutionCode: CNC; basisOfRecord: PreservedSpecimen
- **Type status:** Paratype. **Occurrence:** catalogNumber: CNC_Diptera243287; recordedBy: B.J. Sinclair; individualCount: 1; sex: female; lifeStage: adult; **Taxon:** scientificName: Hammerschmidtiasedmani Vockeroth, Moran and Skevington; kingdom: Animalia; phylum: Arthropoda; class: Hexapoda; order: Diptera; family: Syrphidae; genus: Hammerschmidtia; specificEpithet: sedmani; taxonRank: species; scientificNameAuthorship: Vockeroth, Moran and Skevington 2019; vernacularName: Pale-bristled Logsitter; nomenclaturalStatus: new species; **Location:** country: Canada; stateProvince: Ontario; locality: Ottawa, Marlborough Forest Rideau Trail; decimalLatitude: 45.05; decimalLongitude: -75.816667; **Identification:** identifiedBy: J.R. Vockeroth; **Event:** eventDate: 2011-05-26; year: 2011; month: 5; day: 26; verbatimEventDate: 26.v.2011; habitat: near Rogers Pond; **Record Level:** institutionCode: CNC; basisOfRecord: PreservedSpecimen
- **Type status:** Paratype. **Occurrence:** catalogNumber: CNC_Diptera35319; recordedBy: E.D.A. Dyer; individualCount: 1; sex: male; lifeStage: adult; **Taxon:** scientificName: Hammerschmidtiasedmani Vockeroth, Moran and Skevington; kingdom: Animalia; phylum: Arthropoda; class: Hexapoda; order: Diptera; family: Syrphidae; genus: Hammerschmidtia; specificEpithet: sedmani; taxonRank: species; scientificNameAuthorship: Vockeroth, Moran and Skevington 2019; vernacularName: Pale-bristled Logsitter; nomenclaturalStatus: new species; **Location:** country: Canada; stateProvince: British Columbia; locality: Hixon; decimalLatitude: 53.4; decimalLongitude: -122.566667; **Identification:** identifiedBy: J.R. Vockeroth; **Event:** eventDate: 1966-06-07; year: 1966; month: 6; day: 7; verbatimEventDate: 7.vi.1966; **Record Level:** institutionCode: CNC; basisOfRecord: PreservedSpecimen
- **Type status:** Paratype. **Occurrence:** catalogNumber: CNC_Diptera49254; recordedBy: H.J. Teskey; individualCount: 1; sex: male; lifeStage: adult; **Taxon:** scientificName: Hammerschmidtiasedmani Vockeroth, Moran and Skevington; kingdom: Animalia; phylum: Arthropoda; class: Hexapoda; order: Diptera; family: Syrphidae; genus: Hammerschmidtia; specificEpithet: sedmani; taxonRank: species; scientificNameAuthorship: Vockeroth, Moran and Skevington 2019; vernacularName: Pale-bristled Logsitter; nomenclaturalStatus: new species; **Location:** country: Canada; stateProvince: Alberta; locality: Kananaskis, Forest Experimental Station Seebe; decimalLatitude: 51.100879; decimalLongitude: -115.087692; **Identification:** identifiedBy: J.R. Vockeroth; **Event:** eventDate: 1968-07-03; year: 1968; month: 7; day: 3; verbatimEventDate: 3.vii.1968; **Record Level:** institutionCode: CNC; basisOfRecord: PreservedSpecimen
- **Type status:** Paratype. **Occurrence:** catalogNumber: CNC_Diptera49255; recordedBy: J.B. Wallis; individualCount: 1; sex: male; lifeStage: adult; **Taxon:** scientificName: Hammerschmidtiasedmani Vockeroth, Moran and Skevington; kingdom: Animalia; phylum: Arthropoda; class: Hexapoda; order: Diptera; family: Syrphidae; genus: Hammerschmidtia; specificEpithet: sedmani; taxonRank: species; scientificNameAuthorship: Vockeroth, Moran and Skevington 2019; vernacularName: Pale-bristled Logsitter; nomenclaturalStatus: new species; **Location:** country: Canada; stateProvince: Ontario; locality: Ogoki; decimalLatitude: 51.633716; decimalLongitude: -85.933333; **Identification:** identifiedBy: J.R. Vockeroth; **Event:** eventDate: 1952-07-05; year: 1952; month: 7; day: 5; verbatimEventDate: 5.vii.1952; **Record Level:** institutionCode: CNC; basisOfRecord: PreservedSpecimen
- **Type status:** Paratype. **Occurrence:** catalogNumber: CNC_Diptera49256; recordedBy: H.S. Fleming; individualCount: 1; sex: male; lifeStage: adult; **Taxon:** scientificName: Hammerschmidtiasedmani Vockeroth, Moran and Skevington; kingdom: Animalia; phylum: Arthropoda; class: Hexapoda; order: Diptera; family: Syrphidae; genus: Hammerschmidtia; specificEpithet: sedmani; taxonRank: species; scientificNameAuthorship: Vockeroth, Moran and Skevington 2019; vernacularName: Pale-bristled Logsitter; nomenclaturalStatus: new species; **Location:** country: Canada; stateProvince: Quebec; locality: Laniel; decimalLatitude: 47.033333; decimalLongitude: -79.266667; **Identification:** identifiedBy: J.R. Vockeroth; **Event:** eventDate: 1931-06-10; year: 1931; month: 6; day: 10; verbatimEventDate: 10.vi.1931; **Record Level:** institutionCode: CNC; basisOfRecord: PreservedSpecimen
- **Type status:** Paratype. **Occurrence:** catalogNumber: CNC_Diptera49257; recordedBy: G.E. Shewell; individualCount: 1; sex: male; lifeStage: adult; **Taxon:** scientificName: Hammerschmidtiasedmani Vockeroth, Moran and Skevington; kingdom: Animalia; phylum: Arthropoda; class: Hexapoda; order: Diptera; family: Syrphidae; genus: Hammerschmidtia; specificEpithet: sedmani; taxonRank: species; scientificNameAuthorship: Vockeroth, Moran and Skevington 2019; vernacularName: Pale-bristled Logsitter; nomenclaturalStatus: new species; **Location:** country: Canada; stateProvince: Quebec; locality: Parke Reserve, Kamouraska County; decimalLatitude: 47.523387; decimalLongitude: -69.624739; **Identification:** identifiedBy: J.R. Vockeroth; **Event:** eventDate: 1957-07-10; year: 1957; month: 7; day: 10; verbatimEventDate: 1957-07-10; **Record Level:** institutionCode: CNC; basisOfRecord: PreservedSpecimen
- **Type status:** Paratype. **Occurrence:** catalogNumber: CNC_Diptera49258; recordedBy: B. Heming; individualCount: 1; sex: male; lifeStage: adult; **Taxon:** scientificName: Hammerschmidtiasedmani Vockeroth, Moran and Skevington; kingdom: Animalia; phylum: Arthropoda; class: Hexapoda; order: Diptera; family: Syrphidae; genus: Hammerschmidtia; specificEpithet: sedmani; taxonRank: species; scientificNameAuthorship: Vockeroth, Moran and Skevington 2019; vernacularName: Pale-bristled Logsitter; nomenclaturalStatus: new species; **Location:** country: Canada; stateProvince: British Columbia; locality: Shames, 18 Miles South West of Terrace; minimumElevationInMeters: 32; decimalLatitude: 54.409648; decimalLongitude: -128.935301; **Identification:** identifiedBy: J.R. Vockeroth; **Event:** eventDate: 1960-07-17; year: 1960; month: 7; day: 17; verbatimEventDate: 17.vii.1960; **Record Level:** institutionCode: CNC; basisOfRecord: PreservedSpecimen
- **Type status:** Paratype. **Occurrence:** catalogNumber: CNC_Diptera49259; recordedBy: C.H. Curran; individualCount: 1; sex: male; lifeStage: adult; **Taxon:** scientificName: Hammerschmidtiasedmani Vockeroth, Moran and Skevington; kingdom: Animalia; phylum: Arthropoda; class: Hexapoda; order: Diptera; family: Syrphidae; genus: Hammerschmidtia; specificEpithet: sedmani; taxonRank: species; scientificNameAuthorship: Vockeroth, Moran and Skevington 2019; vernacularName: Pale-bristled Logsitter; nomenclaturalStatus: new species; **Location:** country: Canada; stateProvince: Quebec; locality: Mégantic; decimalLatitude: 45.583333; decimalLongitude: -70.883333; **Identification:** identifiedBy: J.R. Vockeroth; **Event:** eventDate: 1923-06-21; year: 1923; month: 6; day: 21; verbatimEventDate: 21.vi.1923; **Record Level:** institutionCode: CNC; basisOfRecord: PreservedSpecimen
- **Type status:** Paratype. **Occurrence:** catalogNumber: CNC_Diptera49260; recordedBy: H.J. Teskey; individualCount: 1; sex: male; lifeStage: adult; **Taxon:** scientificName: Hammerschmidtiasedmani Vockeroth, Moran and Skevington; kingdom: Animalia; phylum: Arthropoda; class: Hexapoda; order: Diptera; family: Syrphidae; genus: Hammerschmidtia; specificEpithet: sedmani; taxonRank: species; scientificNameAuthorship: Vockeroth, Moran and Skevington 2019; vernacularName: Pale-bristled Logsitter; nomenclaturalStatus: new species; **Location:** country: Canada; stateProvince: Alberta; locality: Kananaskis, Forest Experimental Station Seebe; decimalLatitude: 51.100883; decimalLongitude: -115.087692; **Identification:** identifiedBy: J.R. Vockeroth; **Event:** eventDate: 1968-07-03; year: 1968; month: 7; day: 3; verbatimEventDate: 3.vii.1968; **Record Level:** institutionCode: CNC; basisOfRecord: PreservedSpecimen
- **Type status:** Paratype. **Occurrence:** catalogNumber: CNC_Diptera49261; recordedBy: W.R. Richards; individualCount: 1; sex: female; lifeStage: adult; **Taxon:** scientificName: Hammerschmidtiasedmani Vockeroth, Moran and Skevington; kingdom: Animalia; phylum: Arthropoda; class: Hexapoda; order: Diptera; family: Syrphidae; genus: Hammerschmidtia; specificEpithet: sedmani; taxonRank: species; scientificNameAuthorship: Vockeroth, Moran and Skevington 2019; vernacularName: Pale-bristled Logsitter; nomenclaturalStatus: new species; **Location:** country: Canada; stateProvince: Quebec; locality: Harrington Lake, Gatineau Park [Lac Mousseau]; decimalLatitude: 45.568; decimalLongitude: -75.954; **Identification:** identifiedBy: J.R. Vockeroth; **Event:** eventDate: 1954-06-09; year: 1954; month: 6; day: 9; verbatimEventDate: 9.vi.1954; **Record Level:** institutionCode: CNC; basisOfRecord: PreservedSpecimen
- **Type status:** Paratype. **Occurrence:** catalogNumber: CNC_Diptera49262; recordedBy: D.M. Wood; individualCount: 1; sex: female; lifeStage: adult; **Taxon:** scientificName: Hammerschmidtiasedmani Vockeroth, Moran and Skevington; kingdom: Animalia; phylum: Arthropoda; class: Hexapoda; order: Diptera; family: Syrphidae; genus: Hammerschmidtia; specificEpithet: sedmani; taxonRank: species; scientificNameAuthorship: Vockeroth, Moran and Skevington 2019; vernacularName: Pale-bristled Logsitter; nomenclaturalStatus: new species; **Location:** country: Canada; stateProvince: Quebec; locality: Masham Township; Gatineau; decimalLatitude: 45.683333; decimalLongitude: -76.05; **Identification:** identifiedBy: J.R. Vockeroth; **Event:** eventDate: 1974-06-25; year: 1974; month: 6; day: 25; verbatimEventDate: 25.vi.1974; habitat: sphagnum bog; **Record Level:** institutionCode: CNC; basisOfRecord: PreservedSpecimen
- **Type status:** Paratype. **Occurrence:** catalogNumber: CNC_Diptera49263; recordedBy: D.M. Wood; individualCount: 1; sex: female; lifeStage: adult; **Taxon:** scientificName: Hammerschmidtiasedmani Vockeroth, Moran and Skevington; kingdom: Animalia; phylum: Arthropoda; class: Hexapoda; order: Diptera; family: Syrphidae; genus: Hammerschmidtia; specificEpithet: sedmani; taxonRank: species; scientificNameAuthorship: Vockeroth, Moran and Skevington 2019; vernacularName: Pale-bristled Logsitter; nomenclaturalStatus: new species; **Location:** country: Canada; stateProvince: Quebec; locality: Masham Township; Gatineau; decimalLatitude: 45.683333; decimalLongitude: -76.05; **Identification:** identifiedBy: J.R. Vockeroth; **Event:** eventDate: 1974-06-25; year: 1974; month: 6; day: 25; verbatimEventDate: 25.vi.1974; habitat: sphagnum bog; **Record Level:** institutionCode: CNC; basisOfRecord: PreservedSpecimen
- **Type status:** Paratype. **Occurrence:** catalogNumber: CNC_Diptera49264; recordedBy: E.R. Buckell; individualCount: 1; sex: female; lifeStage: adult; **Taxon:** scientificName: Hammerschmidtiasedmani Vockeroth, Moran and Skevington; kingdom: Animalia; phylum: Arthropoda; class: Hexapoda; order: Diptera; family: Syrphidae; genus: Hammerschmidtia; specificEpithet: sedmani; taxonRank: species; scientificNameAuthorship: Vockeroth, Moran and Skevington 2019; vernacularName: Pale-bristled Logsitter; nomenclaturalStatus: new species; **Location:** country: Canada; stateProvince: British Columbia; locality: Barkerville; decimalLatitude: 53.083426; decimalLongitude: -121.510871; **Identification:** identifiedBy: J.R. Vockeroth; **Event:** eventDate: 1948-07-17; year: 1948; month: 7; day: 17; verbatimEventDate: 17.vii.1948; **Record Level:** institutionCode: CNC; basisOfRecord: PreservedSpecimen
- **Type status:** Paratype. **Occurrence:** catalogNumber: CNC_Diptera49265; recordedBy: C.H. Curran; individualCount: 1; sex: female; lifeStage: adult; **Taxon:** scientificName: Hammerschmidtiasedmani Vockeroth, Moran and Skevington; kingdom: Animalia; phylum: Arthropoda; class: Hexapoda; order: Diptera; family: Syrphidae; genus: Hammerschmidtia; specificEpithet: sedmani; taxonRank: species; scientificNameAuthorship: Vockeroth, Moran and Skevington 2019; vernacularName: Pale-bristled Logsitter; nomenclaturalStatus: new species; **Location:** country: Canada; stateProvince: Quebec; locality: Mégantic; decimalLatitude: 45.583333; decimalLongitude: -70.883333; **Identification:** identifiedBy: J.R. Vockeroth; **Event:** eventDate: 1923-06-26; year: 1923; month: 6; day: 26; verbatimEventDate: 26.vi.1923; **Record Level:** institutionCode: CNC; basisOfRecord: PreservedSpecimen
- **Type status:** Paratype. **Occurrence:** catalogNumber: CNC_Diptera49266; recordedBy: C.H. Curran; individualCount: 1; sex: female; lifeStage: adult; **Taxon:** scientificName: Hammerschmidtiasedmani Vockeroth, Moran and Skevington; kingdom: Animalia; phylum: Arthropoda; class: Hexapoda; order: Diptera; family: Syrphidae; genus: Hammerschmidtia; specificEpithet: sedmani; taxonRank: species; scientificNameAuthorship: VockerVockeroth, Moran and Skevington 2019; vernacularName: Pale-bristled Logsitter; nomenclaturalStatus: new species; **Location:** country: Canada; stateProvince: Ontario; locality: Ottawa; decimalLatitude: 45.423; decimalLongitude: -75.698; **Identification:** identifiedBy: J.R. Vockeroth; **Event:** eventDate: 1927-06-02; year: 1927; month: 6; day: 2; verbatimEventDate: 2.vi.1927; **Record Level:** institutionCode: CNC; basisOfRecord: PreservedSpecimen
- **Type status:** Paratype. **Occurrence:** catalogNumber: CNC_Diptera49267; recordedBy: J.F. McAlpine; individualCount: 1; sex: female; lifeStage: adult; **Taxon:** scientificName: Hammerschmidtiasedmani Vockeroth, Moran and Skevington; kingdom: Animalia; phylum: Arthropoda; class: Hexapoda; order: Diptera; family: Syrphidae; genus: Hammerschmidtia; specificEpithet: sedmani; taxonRank: species; scientificNameAuthorship: Vockeroth, Moran and Skevington 2019; vernacularName: Pale-bristled Logsitter; nomenclaturalStatus: new species; **Location:** country: Canada; stateProvince: Quebec; locality: Duncan Lake, near Rupert; decimalLatitude: 45.681389; decimalLongitude: -76.050278; **Identification:** identifiedBy: J.R. Vockeroth; **Event:** eventDate: 1971-06-17; year: 1971; month: 6; day: 17; verbatimEventDate: 17.vi.1971; **Record Level:** institutionCode: CNC; basisOfRecord: PreservedSpecimen
- **Type status:** Paratype. **Occurrence:** catalogNumber: CNC_Diptera49268; recordedBy: J.R. Vockeroth; individualCount: 1; sex: female; lifeStage: adult; **Taxon:** scientificName: Hammerschmidtiasedmani Vockeroth, Moran and Skevington; kingdom: Animalia; phylum: Arthropoda; class: Hexapoda; order: Diptera; family: Syrphidae; genus: Hammerschmidtia; specificEpithet: sedmani; taxonRank: species; scientificNameAuthorship: Vockeroth, Moran and Skevington 2019; vernacularName: Pale-bristled Logsitter; nomenclaturalStatus: new species; **Location:** country: Canada; stateProvince: Ontario; locality: Orleans, Chapel Hill; decimalLatitude: 45.466667; decimalLongitude: -75.516667; **Identification:** identifiedBy: J.R. Vockeroth; **Event:** samplingProtocol: sweeping; eventDate: 1997-06-26; year: 1997; month: 6; day: 26; verbatimEventDate: 26.vi.1997; **Record Level:** institutionCode: CNC; basisOfRecord: PreservedSpecimen
- **Type status:** Paratype. **Occurrence:** catalogNumber: CNC_Diptera49269; recordedBy: W.R.M. Mason; individualCount: 1; sex: female; lifeStage: adult; **Taxon:** scientificName: Hammerschmidtiasedmani Vockeroth, Moran and Skevington; kingdom: Animalia; phylum: Arthropoda; class: Hexapoda; order: Diptera; family: Syrphidae; genus: Hammerschmidtia; specificEpithet: sedmani; taxonRank: species; scientificNameAuthorship: Vockeroth, Moran and Skevington 2019; vernacularName: Pale-bristled Logsitter; nomenclaturalStatus: new species; **Location:** country: Canada; stateProvince: Alberta; locality: Waterton Lakes National Park; minimumElevationInMeters: 1300; decimalLatitude: 49.083313; decimalLongitude: -113.916732; **Identification:** identifiedBy: J.R. Vockeroth; **Event:** eventDate: 1980-06-21; year: 1980; month: 6; day: 21; verbatimEventDate: 21.vi.1980; **Record Level:** institutionCode: CNC; basisOfRecord: PreservedSpecimen
- **Type status:** Paratype. **Occurrence:** catalogNumber: CNC_Diptera49270; recordedBy: J.R. Vockeroth; individualCount: 1; sex: male; lifeStage: adult; **Taxon:** scientificName: Hammerschmidtiasedmani Vockeroth, Moran and Skevington; kingdom: Animalia; phylum: Arthropoda; class: Hexapoda; order: Diptera; family: Syrphidae; genus: Hammerschmidtia; specificEpithet: sedmani; taxonRank: species; scientificNameAuthorship: Vockeroth, Moran and Skevington 2019; vernacularName: Pale-bristled Logsitter; nomenclaturalStatus: new species; **Location:** country: Canada; stateProvince: Ontario; locality: Orleans, Chapel Hill; decimalLatitude: 45.466667; decimalLongitude: -75.516667; **Identification:** identifiedBy: J.R. Vockeroth; **Event:** samplingProtocol: sweeping; eventDate: 1997-06-23; year: 1997; month: 6; day: 23; verbatimEventDate: 23.vi.1997; **Record Level:** institutionCode: CNC; basisOfRecord: PreservedSpecimen
- **Type status:** Paratype. **Occurrence:** catalogNumber: CNC_Diptera556; recordedBy: E.R. Buckell; individualCount: 1; sex: female; lifeStage: adult; **Taxon:** scientificName: Hammerschmidtiasedmani Vockeroth, Moran and Skevington; kingdom: Animalia; phylum: Arthropoda; class: Hexapoda; order: Diptera; family: Syrphidae; genus: Hammerschmidtia; specificEpithet: sedmani; taxonRank: species; scientificNameAuthorship: Vockeroth, Moran and Skevington 2019; vernacularName: Pale-bristled Logsitter; nomenclaturalStatus: new species; **Location:** country: Canada; stateProvince: British Columbia; locality: Barkerville; decimalLatitude: 53.066667; decimalLongitude: -121.516667; **Identification:** identifiedBy: J.R. Vockeroth; **Event:** eventDate: 1948-07-17; year: 1948; month: 7; day: 17; verbatimEventDate: 17.vii.1948; **Record Level:** institutionCode: CNC; basisOfRecord: PreservedSpecimen
- **Type status:** Paratype. **Occurrence:** catalogNumber: CNC_Diptera574; recordedBy: Kelton & Whitney; individualCount: 1; sex: female; lifeStage: adult; **Taxon:** scientificName: Hammerschmidtiasedmani Vockeroth, Moran and Skevington; kingdom: Animalia; phylum: Arthropoda; class: Hexapoda; order: Diptera; family: Syrphidae; genus: Hammerschmidtia; specificEpithet: sedmani; taxonRank: species; scientificNameAuthorship: Vockeroth, Moran and Skevington 2019; vernacularName: Pale-bristled Logsitter; nomenclaturalStatus: new species; **Location:** country: Canada; stateProvince: Ontario; locality: One Sided Lake; decimalLatitude: 49.05; decimalLongitude: -93.883333; **Identification:** identifiedBy: J.R. Vockeroth; **Event:** eventDate: 1960-06-14; year: 1960; month: 6; day: 14; verbatimEventDate: 14.vi.1960; **Record Level:** institutionCode: CNC; basisOfRecord: PreservedSpecimen
- **Type status:** Paratype. **Occurrence:** catalogNumber: CNC_Diptera60262; recordedBy: S. & J. Peck; individualCount: 1; sex: female; lifeStage: adult; **Taxon:** scientificName: Hammerschmidtiasedmani Vockeroth, Moran and Skevington; kingdom: Animalia; phylum: Arthropoda; class: Hexapoda; order: Diptera; family: Syrphidae; genus: Hammerschmidtia; specificEpithet: sedmani; taxonRank: species; scientificNameAuthorship: Vockeroth, Moran and Skevington 2019; vernacularName: Pale-bristled Logsitter; nomenclaturalStatus: new species; **Location:** country: Canada; stateProvince: Alberta; locality: Demmitt, 40 km North West of Beaverlodge; decimalLatitude: 55.450035; decimalLongitude: -119.89992; **Identification:** identifiedBy: J.R. Vockeroth; **Event:** samplingProtocol: malaise trap; eventDate: 1984-06/09-12/06; eventTime: 164; startDayOfYear: 250; year: 1984; month: 6; day: 12; verbatimEventDate: 12.vi.-6.xi.1984; habitat: Forest Pupulus; **Record Level:** institutionCode: CNC; basisOfRecord: PreservedSpecimen
- **Type status:** Paratype. **Occurrence:** catalogNumber: CNC_Diptera60560; recordedBy: S. & J. Peck; individualCount: 1; sex: female; lifeStage: adult; **Taxon:** scientificName: Hammerschmidtiasedmani Vockeroth, Moran and Skevington; kingdom: Animalia; phylum: Arthropoda; class: Hexapoda; order: Diptera; family: Syrphidae; genus: Hammerschmidtia; specificEpithet: sedmani; taxonRank: species; scientificNameAuthorship: Vockeroth, Moran and Skevington 2019; vernacularName: Pale-bristled Logsitter; nomenclaturalStatus: new species; **Location:** country: Canada; stateProvince: British Columbia; locality: 14 km East of Coal River; decimalLatitude: 59.649875; decimalLongitude: -126.683892; **Identification:** identifiedBy: J.R. Vockeroth; **Event:** eventDate: 1984-06/09-14/03; eventTime: 166; startDayOfYear: 247; year: 1984; month: 6; day: 14; verbatimEventDate: 14.vi.-3.ix.1984; habitat: Picea-Alnus forest; **Record Level:** institutionCode: CNC; basisOfRecord: PreservedSpecimen
- **Type status:** Paratype. **Occurrence:** catalogNumber: CNC_Diptera94610; recordedBy: S. & J. Peck; individualCount: 1; sex: female; lifeStage: adult; **Taxon:** scientificName: Hammerschmidtiasedmani Vockeroth, Moran and Skevington; kingdom: Animalia; phylum: Arthropoda; class: Hexapoda; order: Diptera; family: Syrphidae; genus: Hammerschmidtia; specificEpithet: sedmani; taxonRank: species; scientificNameAuthorship: Vockeroth, Moran and Skevington 2019; vernacularName: Pale-bristled Logsitter; nomenclaturalStatus: new species; **Location:** country: Canada; stateProvince: Alberta; locality: Demmitt, 40 km North West of Beaverlodge; decimalLatitude: 55.450036; decimalLongitude: -119.899922; **Identification:** identifiedBy: J.R. Vockeroth; **Event:** eventDate: 1984-06/09-12/06; eventTime: 164; startDayOfYear: 250; year: 1984; month: 6; day: 12; verbatimEventDate: 12.vi.-6.xi.1984; habitat: Forest; **Record Level:** institutionCode: CNC; basisOfRecord: PreservedSpecimen
- **Type status:** Paratype. **Occurrence:** catalogNumber: CNC_Diptera96475; recordedBy: M. Sanborne; individualCount: 1; sex: female; lifeStage: adult; **Taxon:** scientificName: Hammerschmidtiasedmani Vockeroth, Moran and Skevington; kingdom: Animalia; phylum: Arthropoda; class: Hexapoda; order: Diptera; family: Syrphidae; genus: Hammerschmidtia; specificEpithet: sedmani; taxonRank: species; scientificNameAuthorship: Vockeroth, Moran and Skevington 2019; vernacularName: Pale-bristled Logsitter; nomenclaturalStatus: new species; **Location:** country: Canada; stateProvince: Ontario; locality: Stittsville, Carleton County; decimalLatitude: 45.263479; decimalLongitude: -75.925163; **Identification:** identifiedBy: J.R. Vockeroth; **Event:** eventDate: 1977-05-17/20; eventTime: 137; startDayOfYear: 140; year: 1977; month: 5; day: 17; verbatimEventDate: 17-20.v.1977; **Record Level:** institutionCode: CNC; basisOfRecord: PreservedSpecimen
- **Type status:** Paratype. **Occurrence:** catalogNumber: CNC_Diptera96476; recordedBy: M. Sanborne; individualCount: 1; sex: male; lifeStage: adult; **Taxon:** scientificName: Hammerschmidtiasedmani Vockeroth, Moran and Skevington; kingdom: Animalia; phylum: Arthropoda; class: Hexapoda; order: Diptera; family: Syrphidae; genus: Hammerschmidtia; specificEpithet: sedmani; taxonRank: species; scientificNameAuthorship: Vockeroth, Moran and Skevington 2019; vernacularName: Pale-bristled Logsitter; nomenclaturalStatus: new species; **Location:** country: Canada; stateProvince: Ontario; locality: Stittsville, Carleton County; decimalLatitude: 45.263479; decimalLongitude: -75.925163; **Identification:** identifiedBy: J.R. Vockeroth; **Event:** eventDate: 1977-05-20/22; eventTime: 140; startDayOfYear: 142; year: 1977; month: 5; day: 20; verbatimEventDate: 20-22.v.1977; **Record Level:** institutionCode: CNC; basisOfRecord: PreservedSpecimen
- **Type status:** Paratype. **Occurrence:** catalogNumber: CNC_Diptera96844; recordedBy: J.R. Vockeroth; individualCount: 1; sex: female; lifeStage: adult; **Taxon:** scientificName: Hammerschmidtiasedmani Vockeroth, Moran and Skevington; kingdom: Animalia; phylum: Arthropoda; class: Hexapoda; order: Diptera; family: Syrphidae; genus: Hammerschmidtia; specificEpithet: sedmani; taxonRank: species; scientificNameAuthorship: Vockeroth, Moran and Skevington 2019; vernacularName: Pale-bristled Logsitter; nomenclaturalStatus: new species; **Location:** country: Canada; stateProvince: Ontario; locality: Orleans, Chapel Hill; decimalLatitude: 45.466667; decimalLongitude: -75.516667; **Identification:** identifiedBy: J.R. Vockeroth; **Event:** samplingProtocol: sweeping; eventDate: 1995-06-21; year: 1995; month: 6; day: 21; verbatimEventDate: 21.vi.1995; habitat: meadow area; **Record Level:** institutionCode: CNC; basisOfRecord: PreservedSpecimen
- **Type status:** Paratype. **Occurrence:** catalogNumber: CNC_Diptera96845; recordedBy: J.R. Vockeroth; individualCount: 1; sex: female; lifeStage: adult; **Taxon:** scientificName: Hammerschmidtiasedmani Vockeroth, Moran and Skevington; kingdom: Animalia; phylum: Arthropoda; class: Hexapoda; order: Diptera; family: Syrphidae; genus: Hammerschmidtia; specificEpithet: sedmani; taxonRank: species; scientificNameAuthorship: Vockeroth, Moran and Skevington 2019; vernacularName: Pale-bristled Logsitter; nomenclaturalStatus: new species; **Location:** country: Canada; stateProvince: Ontario; locality: Orleans, Chapel Hill; decimalLatitude: 45.466667; decimalLongitude: -75.516667; **Identification:** identifiedBy: J.R. Vockeroth; **Event:** samplingProtocol: sweeping; eventDate: 1995-06-21; year: 1995; month: 6; day: 21; verbatimEventDate: 21.vi.1995; habitat: meadow area; **Record Level:** institutionCode: CNC; basisOfRecord: PreservedSpecimen
- **Type status:** Paratype. **Occurrence:** catalogNumber: CNC_Diptera96846; recordedBy: J.R. Vockeroth; individualCount: 1; sex: female; lifeStage: adult; **Taxon:** scientificName: Hammerschmidtiasedmani Vockeroth, Moran and Skevington; kingdom: Animalia; phylum: Arthropoda; class: Hexapoda; order: Diptera; family: Syrphidae; genus: Hammerschmidtia; specificEpithet: sedmani; taxonRank: species; scientificNameAuthorship: Vockeroth, Moran and Skevington 2019; vernacularName: Pale-bristled Logsitter; nomenclaturalStatus: new species; **Location:** country: Canada; stateProvince: Ontario; locality: Orleans, Chapel Hill; decimalLatitude: 45.466667; decimalLongitude: -75.516667; **Identification:** identifiedBy: J.R. Vockeroth; **Event:** samplingProtocol: sweeping; eventDate: 1995-06-21; year: 1995; month: 6; day: 21; verbatimEventDate: 21.vi.1995; habitat: meadow area; **Record Level:** institutionCode: CNC; basisOfRecord: PreservedSpecimen
- **Type status:** Paratype. **Occurrence:** catalogNumber: CNC_Diptera96847; recordedBy: J.R. Vockeroth; individualCount: 1; sex: male; lifeStage: adult; **Taxon:** scientificName: Hammerschmidtiasedmani Vockeroth, Moran and Skevington; kingdom: Animalia; phylum: Arthropoda; class: Hexapoda; order: Diptera; family: Syrphidae; genus: Hammerschmidtia; specificEpithet: sedmani; taxonRank: species; scientificNameAuthorship: Vockeroth, Moran and Skevington 2019; vernacularName: Pale-bristled Logsitter; nomenclaturalStatus: new species; **Location:** country: Canada; stateProvince: Ontario; locality: Orleans, Chapel Hill; decimalLatitude: 45.466667; decimalLongitude: -75.516667; **Identification:** identifiedBy: J.R. Vockeroth; **Event:** samplingProtocol: sweeping; eventDate: 1995-06-24; year: 1995; month: 6; day: 24; verbatimEventDate: 24.vi.1995; habitat: meadow area; **Record Level:** institutionCode: CNC; basisOfRecord: PreservedSpecimen
- **Type status:** Paratype. **Occurrence:** catalogNumber: JK10466; recordedBy: J. Klymko; individualCount: 1; sex: male; lifeStage: adult; **Taxon:** scientificName: Hammerschmidtiasedmani Vockeroth, Moran and Skevington; kingdom: Animalia; phylum: Arthropoda; class: Hexapoda; order: Diptera; family: Syrphidae; genus: Hammerschmidtia; specificEpithet: sedmani; taxonRank: species; scientificNameAuthorship: Vockeroth, Moran and Skevington 2019; vernacularName: Pale-bristled Logsitter; nomenclaturalStatus: new species; **Location:** country: Canada; stateProvince: New Brunswick; locality: Northumberland County, Nepisiguit Protected Natural Area, 1.0km northwest of Popple Depot; decimalLatitude: 47.4024; decimalLongitude: -66.522066; **Identification:** identifiedBy: J. Klymko; **Event:** eventDate: 2017-06-22; year: 2017; month: 6; day: 22; verbatimEventDate: 22.vi.2017; habitat: open Picea forest; **Record Level:** institutionCode: NBMB; basisOfRecord: PreservedSpecimen
- **Type status:** Paratype. **Occurrence:** catalogNumber: JK10468; recordedBy: J. Klymko; individualCount: 1; sex: female; lifeStage: adult; **Taxon:** scientificName: Hammerschmidtiasedmani Vockeroth, Moran and Skevington; kingdom: Animalia; phylum: Arthropoda; class: Hexapoda; order: Diptera; family: Syrphidae; genus: Hammerschmidtia; specificEpithet: sedmani; taxonRank: species; scientificNameAuthorship: Vockeroth, Moran and Skevington 2019; vernacularName: Pale-bristled Logsitter; nomenclaturalStatus: new species; **Location:** country: Canada; stateProvince: New Brunswick; locality: Northumberland County, Nepisiguit Protected Natural Area, 1.0km northwest of Popple Depot; decimalLatitude: 47.4024; decimalLongitude: -66.522066; **Identification:** identifiedBy: J. Klymko; **Event:** eventDate: 2017-06-22; year: 2017; month: 6; day: 22; verbatimEventDate: 22.vi.2017; habitat: open Picea forest; **Record Level:** institutionCode: NBMB; basisOfRecord: PreservedSpecimen
- **Type status:** Paratype. **Occurrence:** catalogNumber: JK10470; recordedBy: J. Klymko; individualCount: 1; sex: male; lifeStage: adult; **Taxon:** scientificName: Hammerschmidtiasedmani Vockeroth, Moran and Skevington; kingdom: Animalia; phylum: Arthropoda; class: Hexapoda; order: Diptera; family: Syrphidae; genus: Hammerschmidtia; specificEpithet: sedmani; taxonRank: species; scientificNameAuthorship: Vockeroth, Moran and Skevington 2019; vernacularName: Pale-bristled Logsitter; nomenclaturalStatus: new species; **Location:** country: Canada; stateProvince: New Brunswick; locality: Northumberland County, Nepisiguit Protected Natural Area, 1.0km northwest of Popple Depot; decimalLatitude: 47.4024; decimalLongitude: -66.522066; **Identification:** identifiedBy: J. Klymko; **Event:** eventDate: 2017-06-22; year: 2017; month: 6; day: 22; verbatimEventDate: 22.vi.2017; habitat: open Picea forest; **Record Level:** institutionCode: NBMB; basisOfRecord: PreservedSpecimen
- **Type status:** Paratype. **Occurrence:** catalogNumber: JK10474; recordedBy: J. Klymko; individualCount: 1; sex: male; lifeStage: adult; **Taxon:** scientificName: Hammerschmidtiasedmani Vockeroth, Moran and Skevington; kingdom: Animalia; phylum: Arthropoda; class: Hexapoda; order: Diptera; family: Syrphidae; genus: Hammerschmidtia; specificEpithet: sedmani; taxonRank: species; scientificNameAuthorship: Vockeroth, Moran and Skevington 2019; vernacularName: Pale-bristled Logsitter; nomenclaturalStatus: new species; **Location:** country: Canada; stateProvince: New Brunswick; locality: Northumberland County, Nepisiguit Protected Natural Area, 1.0km northwest of Popple Depot; decimalLatitude: 47.4024; decimalLongitude: -66.522066; **Identification:** identifiedBy: J. Klymko; **Event:** eventDate: 2017-06-22; year: 2017; month: 6; day: 22; verbatimEventDate: 22.vi.2017; habitat: open Picea forest; **Record Level:** institutionCode: NBMB; basisOfRecord: PreservedSpecimen
- **Type status:** Paratype. **Occurrence:** catalogNumber: JK736; recordedBy: J.Klymko,S.L.Robinson; individualCount: 1; sex: male; lifeStage: adult; **Taxon:** scientificName: Hammerschmidtiasedmani Vockeroth, Moran and Skevington; kingdom: Animalia; phylum: Arthropoda; class: Hexapoda; order: Diptera; family: Syrphidae; genus: Hammerschmidtia; specificEpithet: sedmani; taxonRank: species; scientificNameAuthorship: Vockeroth, Moran and Skevington 2019; vernacularName: Pale-bristled Logsitter; nomenclaturalStatus: new species; **Location:** country: Canada; stateProvince: New Brunswick; locality: Restigouche Co., Jacquet R Gg PNA, woods rd in mixed woods along Jacquet R.; decimalLatitude: 47.7733; decimalLongitude: -66.1253; **Identification:** identifiedBy: J. Klymko; **Event:** eventDate: 2010-06-25; year: 2010; month: 6; day: 25; verbatimEventDate: 25.vi.2010; habitat: Mixed Woods; **Record Level:** institutionCode: NBMB; basisOfRecord: PreservedSpecimen
- **Type status:** Paratype. **Occurrence:** catalogNumber: USNMENT22534; recordedBy: B.J. & F.C. Thompson; individualCount: 1; sex: female; lifeStage: adult; **Taxon:** scientificName: Hammerschmidtiasedmani Vockeroth, Moran and Skevington; kingdom: Animalia; phylum: Arthropoda; class: Hexapoda; order: Diptera; family: Syrphidae; genus: Hammerschmidtia; specificEpithet: sedmani; taxonRank: species; scientificNameAuthorship: Vockeroth, Moran and Skevington 2019; vernacularName: Pale-bristled Logsitter; nomenclaturalStatus: new species; **Location:** country: U.S.A.; stateProvince: New Hampshire; locality: Pittsburg, Rt. 3, Connecticut Lakes; decimalLatitude: 45.104264; decimalLongitude: -71.259207; **Identification:** identifiedBy: F.C. Thompson; **Event:** eventDate: 1974-06-22/24; eventTime: 142; startDayOfYear: 144; year: 1974; month: 6; day: 22; verbatimEventDate: 22-24.vi.1974; **Record Level:** institutionCode: USNM; basisOfRecord: PreservedSpecimen
- **Type status:** Paratype. **Occurrence:** catalogNumber: USNMENT22535; recordedBy: B.J. & F.C. Thompson; individualCount: 1; sex: female; lifeStage: adult; **Taxon:** scientificName: Hammerschmidtiasedmani Vockeroth, Moran and Skevington; kingdom: Animalia; phylum: Arthropoda; class: Hexapoda; order: Diptera; family: Syrphidae; genus: Hammerschmidtia; specificEpithet: sedmani; taxonRank: species; scientificNameAuthorship: Vockeroth, Moran and Skevington 2019; vernacularName: Pale-bristled Logsitter; nomenclaturalStatus: new species; **Location:** country: U.S.A.; stateProvince: New Hampshire; locality: Pittsburg, Rt. 3, Connecticut Lakes; decimalLatitude: 45.104264; decimalLongitude: -71.259207; **Identification:** identifiedBy: F.C. Thompson; **Event:** eventDate: 1974-06-22/24; eventTime: 142; startDayOfYear: 144; year: 1974; month: 6; day: 22; verbatimEventDate: 22-24.vi.1974; **Record Level:** institutionCode: USNM; basisOfRecord: PreservedSpecimen
- **Type status:** Paratype. **Occurrence:** catalogNumber: USNMENT22536; recordedBy: B.J. & F.C. Thompson; individualCount: 1; sex: female; lifeStage: adult; **Taxon:** scientificName: Hammerschmidtiasedmani Vockeroth, Moran and Skevington; kingdom: Animalia; phylum: Arthropoda; class: Hexapoda; order: Diptera; family: Syrphidae; genus: Hammerschmidtia; specificEpithet: sedmani; taxonRank: species; scientificNameAuthorship: Vockeroth, Moran and Skevington 2019; vernacularName: Pale-bristled Logsitter; nomenclaturalStatus: new species; **Location:** country: U.S.A.; stateProvince: New Hampshire; locality: Pittsburg, Rt. 3, Connecticut Lakes; decimalLatitude: 45.104264; decimalLongitude: -71.259207; **Identification:** identifiedBy: F.C. Thompson; **Event:** eventDate: 1974-06-22/24; eventTime: 142; startDayOfYear: 144; year: 1974; month: 6; day: 22; verbatimEventDate: 22-24.vi.1974; **Record Level:** institutionCode: USNM; basisOfRecord: PreservedSpecimen
- **Type status:** Paratype. **Occurrence:** catalogNumber: USNMENT249891; recordedBy: A.L. Melander; individualCount: 1; sex: female; lifeStage: adult; **Taxon:** scientificName: Hammerschmidtiasedmani Vockeroth, Moran and Skevington; kingdom: Animalia; phylum: Arthropoda; class: Hexapoda; order: Diptera; family: Syrphidae; genus: Hammerschmidtia; specificEpithet: sedmani; taxonRank: species; scientificNameAuthorship: Vockeroth, Moran and Skevington 2019; vernacularName: Pale-bristled Logsitter; nomenclaturalStatus: new species; **Location:** country: U.S.A.; stateProvince: New Hampshire; locality: White Mountains, Dolly Copp; decimalLatitude: 44.271; decimalLongitude: -71.305; **Identification:** identifiedBy: F.C. Thompson; **Event:** eventDate: 1931-07-11; year: 1931; month: 7; day: 11; verbatimEventDate: 11.vii.1931; **Record Level:** institutionCode: USNM; basisOfRecord: PreservedSpecimen
- **Type status:** Paratype. **Occurrence:** catalogNumber: USNMENT249892; recordedBy: R.P. Currie; individualCount: 1; sex: male; lifeStage: adult; **Taxon:** scientificName: Hammerschmidtiasedmani Vockeroth, Moran and Skevington; kingdom: Animalia; phylum: Arthropoda; class: Hexapoda; order: Diptera; family: Syrphidae; genus: Hammerschmidtia; specificEpithet: sedmani; taxonRank: species; scientificNameAuthorship: Vockeroth, Moran and Skevington 2019; vernacularName: Pale-bristled Logsitter; nomenclaturalStatus: new species; **Location:** country: Canada; stateProvince: British Columbia; locality: Kaslo; decimalLatitude: 49.907907; decimalLongitude: -116.910358; **Identification:** identifiedBy: F.C. Thompson; **Event:** eventDate: 06-15; month: 6; day: 15; verbatimEventDate: 15.vi; **Record Level:** institutionCode: USNM; basisOfRecord: PreservedSpecimen
- **Type status:** Paratype. **Occurrence:** catalogNumber: USNMENT249893; recordedBy: O. Bryant; individualCount: 1; sex: male; lifeStage: adult; **Taxon:** scientificName: Hammerschmidtiasedmani Vockeroth, Moran and Skevington; kingdom: Animalia; phylum: Arthropoda; class: Hexapoda; order: Diptera; family: Syrphidae; genus: Hammerschmidtia; specificEpithet: sedmani; taxonRank: species; scientificNameAuthorship: Vockeroth, Moran and Skevington 2019; vernacularName: Pale-bristled Logsitter; nomenclaturalStatus: new species; **Location:** country: Canada; stateProvince: Alberta; locality: Bilby; decimalLatitude: 53.683333; decimalLongitude: -114.1; **Identification:** identifiedBy: F.C. Thompson; **Event:** eventDate: 1924-06-11; year: 1924; month: 6; day: 11; verbatimEventDate: 11.vi.1924; **Record Level:** institutionCode: USNM; basisOfRecord: PreservedSpecimen
- **Type status:** Paratype. **Occurrence:** catalogNumber: USNMENT249894; recordedBy: D.D. Wilder; individualCount: 1; sex: male; lifeStage: adult; **Taxon:** scientificName: Hammerschmidtiasedmani Vockeroth, Moran and Skevington; kingdom: Animalia; phylum: Arthropoda; class: Hexapoda; order: Diptera; family: Syrphidae; genus: Hammerschmidtia; specificEpithet: sedmani; taxonRank: species; scientificNameAuthorship: Vockeroth, Moran and Skevington 2019; vernacularName: Pale-bristled Logsitter; nomenclaturalStatus: new species; **Location:** country: U.S.A.; stateProvince: Michigan; locality: Macinac Co. Cedarville; decimalLatitude: 45.998; decimalLongitude: -84.3656; **Identification:** identifiedBy: F.C. Thompson; **Event:** eventDate: 1975-06-21; year: 1975; month: 6; day: 21; verbatimEventDate: 21.vi.1975; **Record Level:** institutionCode: CAS; basisOfRecord: PreservedSpecimen
- **Type status:** Paratype. **Occurrence:** catalogNumber: CNC1185349; recordedBy: C. Kassebeer; individualCount: 1; sex: female; lifeStage: adult; **Taxon:** scientificName: Hammerschmidtiasedmani Vockeroth, Moran and Skevington; kingdom: Animalia; phylum: Arthropoda; class: Hexapoda; order: Diptera; family: Syrphidae; genus: Hammerschmidtia; specificEpithet: sedmani; taxonRank: species; scientificNameAuthorship: Vockeroth, Moran and Skevington; vernacularName: Pale-bristled Logsitter; nomenclaturalStatus: new species; **Location:** country: Canada; stateProvince: Quebec; locality: Duncan Lake, near Rupert; decimalLatitude: 45.681389; decimalLongitude: -76.050278; **Identification:** identifiedBy: F.C. Thompson; **Event:** eventDate: 1996-06-29/30; eventTime: 181; startDayOfYear: 182; year: 1996; month: 6; day: 29; verbatimEventDate: 29-30.vi.1996; **Record Level:** institutionCode: CNC; basisOfRecord: PreservedSpecimen
- **Type status:** Paratype. **Occurrence:** catalogNumber: CNC1185350; recordedBy: C. Kassebeer; individualCount: 1; sex: male; lifeStage: adult; **Taxon:** scientificName: Hammerschmidtiasedmani Vockeroth, Moran and Skevington; kingdom: Animalia; phylum: Arthropoda; class: Hexapoda; order: Diptera; family: Syrphidae; genus: Hammerschmidtia; specificEpithet: sedmani; taxonRank: species; scientificNameAuthorship: Vockeroth, Moran and Skevington 2019; vernacularName: Pale-bristled Logsitter; nomenclaturalStatus: new species; **Location:** country: Canada; stateProvince: Quebec; locality: Duncan Lake, near Rupert; decimalLatitude: 45.681389; decimalLongitude: -76.050278; **Identification:** identifiedBy: F.C. Thompson; **Event:** eventDate: 1996-06-29/30; eventTime: 181; startDayOfYear: 182; year: 1996; month: 6; day: 29; verbatimEventDate: 29-30.vi.1996; **Record Level:** institutionCode: CNC; basisOfRecord: PreservedSpecimen
- **Type status:** Paratype. **Occurrence:** catalogNumber: CNC1185351; recordedBy: B.J. & F.C. Thompson; individualCount: 1; sex: female; lifeStage: adult; **Taxon:** scientificName: Hammerschmidtiasedmani Vockeroth, Moran and Skevington; kingdom: Animalia; phylum: Arthropoda; class: Hexapoda; order: Diptera; family: Syrphidae; genus: Hammerschmidtia; specificEpithet: sedmani; taxonRank: species; scientificNameAuthorship: Vockeroth, Moran and Skevington 2019; vernacularName: Pale-bristled Logsitter; nomenclaturalStatus: new species; **Location:** country: U.S.A.; stateProvince: New Hampshire; locality: Grafton Co., Bethlehem, Rt. 3, Rt. 3; decimalLatitude: 44.281166; decimalLongitude: -71.687319; **Identification:** identifiedBy: F.C. Thompson; **Event:** eventDate: 1973-06-18; year: 1973; month: 6; day: 18; verbatimEventDate: 18.vi.1973; **Record Level:** institutionCode: USNM; basisOfRecord: PreservedSpecimen
- **Type status:** Paratype. **Occurrence:** catalogNumber: CNC1185352; recordedBy: C.W. Johnson; individualCount: 1; sex: female; lifeStage: adult; **Taxon:** scientificName: Hammerschmidtiasedmani Vockeroth, Moran and Skevington; kingdom: Animalia; phylum: Arthropoda; class: Hexapoda; order: Diptera; family: Syrphidae; genus: Hammerschmidtia; specificEpithet: sedmani; taxonRank: species; scientificNameAuthorship: Vockeroth, Moran and Skevington 2019; vernacularName: Pale-bristled Logsitter; nomenclaturalStatus: new species; **Location:** country: U.S.A.; stateProvince: New Hampshire; locality: Jaffrey; decimalLatitude: 42.814629; decimalLongitude: -72.023437; **Identification:** identifiedBy: F.C. Thompson; **Event:** eventDate: 06-28; month: 6; day: 28; verbatimEventDate: 28.vi; **Record Level:** institutionCode: MCZ; basisOfRecord: PreservedSpecimen
- **Type status:** Paratype. **Occurrence:** catalogNumber: CNC1185353; recordedBy: H. Notman; individualCount: 1; sex: female; lifeStage: adult; **Taxon:** scientificName: Hammerschmidtiasedmani Vockeroth, Moran and Skevington; kingdom: Animalia; phylum: Arthropoda; class: Hexapoda; order: Diptera; family: Syrphidae; genus: Hammerschmidtia; specificEpithet: sedmani; taxonRank: species; scientificNameAuthorship: Vockeroth, Moran and Skevington 2019; vernacularName: Pale-bristled Logsitter; nomenclaturalStatus: new species; **Location:** country: U.S.A.; stateProvince: New York; locality: Essex Co., Keene Valley; decimalLatitude: 44.190122; decimalLongitude: -73.786586; **Identification:** identifiedBy: F.C. Thompson; **Event:** eventDate: 1920-06-12; year: 1920; month: 6; day: 12; verbatimEventDate: 12.vi.1920; **Record Level:** institutionCode: MCZ; basisOfRecord: PreservedSpecimen
- **Type status:** Paratype. **Occurrence:** catalogNumber: CNC1185354; recordedBy: F.D. Fee; individualCount: 1; sex: female; lifeStage: adult; **Taxon:** scientificName: Hammerschmidtiasedmani Vockeroth, Moran and Skevington; kingdom: Animalia; phylum: Arthropoda; class: Hexapoda; order: Diptera; family: Syrphidae; genus: Hammerschmidtia; specificEpithet: sedmani; taxonRank: species; scientificNameAuthorship: Vockeroth, Moran and Skevington 2019; vernacularName: Pale-bristled Logsitter; nomenclaturalStatus: new species; **Location:** country: U.S.A.; stateProvince: Pennsylvania; locality: Centre Co., Black Moshannon State Park; decimalLatitude: 40.914568; decimalLongitude: -78.061262; **Identification:** identifiedBy: F.C. Thompson; **Event:** eventDate: 1977-06-30; year: 1977; month: 6; day: 30; verbatimEventDate: 30.vi.1977; **Record Level:** institutionCode: ANSP; basisOfRecord: PreservedSpecimen

#### Description

**Size**: Body length 7.5 to 9.5mm; wing length 6.0 to 7.7 mm

**Male: Head**: Orange; face with low tubercle, shiny medially, light (not obscuring orange ground colour) white pollinose laterally; gena shiny anteriorly, white pollinose posteriorly, white pilose; frontal triangle shiny, bare except for short white pile narrowly along eye margin; front bare on ventral ⅓, yellow pilose dorsally; vertical triangle short yellow pilose; occiput light white pollinose, white pilose with some short black setae on dorsal ¼; antenna yellow pilose; postpedicel oval, only slightly (1.2) longer than broad; aristal pile long, with longest pili as long as pedicel width; eyes very narrowly dichoptic, separated by width of an ommatidium; area of approximation short, about 5-6 ommatidia long.

**Thorax**: Dark brownish-orange; postpronotum yellow pilose except for a few black pili medially; mesonotum shiny except light grey pollinose submedial vitta and notopleuron, short black pilose except yellow pilose anteriorly and on notopleuron, with 3 supra-alar and 1-2 postalar black bristles; scutellum shiny, black pilose, with broadly separated apical black bristles and 1-3 subapical black bristles; pleuron sparsely grey pollinose, white pilose except intermixed black pili on posterior anepisternum; anepistemum with 2-3 yellow posterodorsal bristles; katepistemum bare dorsally or with at most a few short pili. Legs: pro- and mesocoxae orange, sparsely white pollinose, white pilose; metacoxa dark brownish-orange, sparsely grey pollinose, yellow pilose, with 1-2 black apicolateral bristles; trochanters orange, pale pilose; profemur orange, shiny, white pilose except black pilose on posterior apical ½; mesofemur orange, black pilose except white pilose on basoventral ½; metafemur brownish-orange, black pilose except narrowly white pilose basally, with black setae on apico-ventral ⅓; pro- and mesotibiae orange, black pilose; metatibia brownish-orange, black pilose, with a few black seta on anterior side; tarsi orange except apical 2 tarsomeres brown, black pilose. Wing: halter orange; calypter white; wing microtrichose, hyaline except apical veins diffusely margined with brown.

**Abdomen**: Brownish-orange, shiny, extensively white pilose, black pilose apicolaterally on 2nd tergum and on apicomedial ½ of 3rd and 4th terga.

**Genitalia**: Epandrium triangular, narrowing posteriorly; cercus with laterally compressed, oval sclerotised outer portion covered in long pile and membranous inner portion; surstylus long, fingerlike with rounded apex and with ventral tooth at about half the length, densely setose on outer surface and loosely setose on inner surface; subepandrial sclerite divided with one arm off of each surstylus, broad proximally and joined only where it articulates with hypandrium; ejaculatory apodeme weakly fan-shaped; phallapodeme long and narrow; postgonite small, quadrate; basiphallus claw-like, broad; distiphallus simple tube.

**Female**: Face concave instead of tuberculate, eyes more broadly dichoptic.

See Fig. 7.

#### Diagnosis

Always orange with pale anepisternal bristles and a sparsely pilose katepisternum.

#### Etymology

This species is named for Yale Sedman in recognition of his early work on the male genitalia and classification of brachyopine flower flies.

#### Distribution

This species is known from New Brunswick to British Columbia, south to Pennsylvania.

#### Ecology

This uncommon species is known to fly from mid-May to mid-July. They have been found visiting *Cornuscanadensis*, *Heracleum*, *Physocarpus* and *Prunusvirginiana* and are known from bogs and mixed woods. Several specimens were collected on a recently fallen aspen log while they were searching for oviposition sites. Larvae of all known species of *Hammerschmidtia* live in recently fallen aspens.

#### Taxon discussion

DNA sequences of this species are available from BOLD and GenBank (Table 2). This new species is similar to *H.rufa*, but can be distinguished by the black anepisternal bristles and katepisternum that is densely pilose.

#### Common Name

The common name given to the species by Skevington et al. (2019) is Pale-bristled Logsitter.

### Microdon (Microdon) scauros

Skevington and Locke sp. n.

88774C9BCB375E65B31A7CB0DBE4D163

urn:lsid:zoobank.org:act:0DDD7DE3-C6F7-490D-A0B3-AA870F1C4815

#### Materials

- **Type status:** Holotype. **Occurrence:** catalogNumber: Jeff_Skevington_Specimen44169; recordedBy: F.D. Fee; individualCount: 1; sex: male; lifeStage: adult; **Taxon:** scientificName: Microdonscauros Skevington and Locke; kingdom: Animalia; phylum: Arthropoda; class: Hexapoda; order: Diptera; family: Syrphidae; genus: Microdon; subgenus: Microdon; specificEpithet: scauros; taxonRank: species; scientificNameAuthorship: Skevington and Locke 2019; vernacularName: Big-footed Ant Fly; nomenclaturalStatus: new species; **Location:** country: United States of America; stateProvince: Pennsylvania; locality: Huntingdon County, Whipple Dam State Park; decimalLatitude: 40.69385; decimalLongitude: -77.857447; **Identification:** identifiedBy: Jeffrey H. Skevington; dateIdentified: 2017; **Event:** samplingProtocol: hand collected; eventDate: 1986-09-03; year: 1986; month: 9; day: 3; habitat: open grassy old field; **Record Level:** language: en; institutionCode: ANSP; basisOfRecord: PreservedSpecimen
- **Type status:** Paratype. **Occurrence:** catalogNumber: Jeff_Skevington_Specimen44200; recordedBy: F.D. Fee; individualCount: 1; sex: female; lifeStage: adult; **Taxon:** scientificName: Microdonscauros Skevington and Locke; kingdom: Animalia; phylum: Arthropoda; class: Hexapoda; order: Diptera; family: Syrphidae; genus: Microdon; subgenus: Microdon; specificEpithet: scauros; taxonRank: species; scientificNameAuthorship: Skevington and Locke 2019; vernacularName: Big-footed Ant Fly; nomenclaturalStatus: new species; **Location:** country: United States of America; stateProvince: Maryland; locality: Allegany County, Fifteen-Mile Creek, Green Ridge State Forest; decimalLatitude: 39.617; decimalLongitude: -78.383; **Identification:** identifiedBy: Jeffrey H. Skevington; dateIdentified: 2017; **Event:** samplingProtocol: hand collected; eventDate: 1978-07-06; year: 1978; month: 7; day: 6; **Record Level:** language: en; institutionCode: ANSP; basisOfRecord: PreservedSpecimen
- **Type status:** Paratype. **Occurrence:** catalogNumber: Jeff_Skevington_Specimen44176; recordedBy: F.D. Fee; individualCount: 1; sex: male; lifeStage: adult; **Taxon:** scientificName: Microdonscauros Skevington and Locke; kingdom: Animalia; phylum: Arthropoda; class: Hexapoda; order: Diptera; family: Syrphidae; genus: Microdon; subgenus: Microdon; specificEpithet: scauros; taxonRank: species; scientificNameAuthorship: Skevington and Locke 2019; vernacularName: Big-footed Ant Fly; nomenclaturalStatus: new species; **Location:** country: United States of America; stateProvince: Pennsylvania; locality: Huntingdon County, Ridge & Nursery Roads, Barree Township; decimalLatitude: 40.642501; decimalLongitude: -77.908807; **Identification:** identifiedBy: Jeffrey H. Skevington; dateIdentified: 2017; **Event:** samplingProtocol: hand collected; eventDate: 1987-08-30; year: 1987; month: 8; day: 30; habitat: perch/veg., dry weedy meadow; **Record Level:** language: en; institutionCode: ANSP; basisOfRecord: PreservedSpecimen
- **Type status:** Paratype. **Occurrence:** catalogNumber: Jeff_Skevington_Specimen44179; recordedBy: F.D. Fee; individualCount: 1; sex: male; lifeStage: adult; **Taxon:** scientificName: Microdonscauros Skevington and Locke; kingdom: Animalia; phylum: Arthropoda; class: Hexapoda; order: Diptera; family: Syrphidae; genus: Microdon; subgenus: Microdon; specificEpithet: scauros; taxonRank: species; scientificNameAuthorship: Skevington and Locke 2019; vernacularName: Big-footed Ant Fly; nomenclaturalStatus: new species; **Location:** country: United States of America; stateProvince: Pennsylvania; locality: Huntingdon County, Ridge & Nursery Roads, Barree Township; decimalLatitude: 40.642501; decimalLongitude: -77.908807; **Identification:** identifiedBy: Jeffrey H. Skevington; dateIdentified: 2017; **Event:** samplingProtocol: hand collected; eventDate: 1987-09-15; year: 1987; month: 9; day: 15; habitat: low veg. adjacent to marsh; **Record Level:** language: en; institutionCode: ANSP; basisOfRecord: PreservedSpecimen
- **Type status:** Paratype. **Occurrence:** catalogNumber: Jeff_Skevington_Specimen44180; recordedBy: F.D. Fee; individualCount: 1; sex: male; lifeStage: adult; **Taxon:** scientificName: Microdonscauros Skevington and Locke; kingdom: Animalia; phylum: Arthropoda; class: Hexapoda; order: Diptera; family: Syrphidae; genus: Microdon; subgenus: Microdon; specificEpithet: scauros; taxonRank: species; scientificNameAuthorship: Skevington and Locke 2019; vernacularName: Big-footed Ant Fly; nomenclaturalStatus: new species; **Location:** country: United States of America; stateProvince: Pennsylvania; locality: Huntingdon County, Ridge & Nursery Roads, Barree Township; decimalLatitude: 40.642501; decimalLongitude: -77.908807; **Identification:** identifiedBy: Jeffrey H. Skevington; dateIdentified: 2017; **Event:** samplingProtocol: hand collected; eventDate: 1987-09-15; year: 1987; month: 9; day: 15; habitat: low veg. adjacent to marsh; **Record Level:** language: en; institutionCode: ANSP; basisOfRecord: PreservedSpecimen
- **Type status:** Paratype. **Occurrence:** catalogNumber: Jeff_Skevington_Specimen44184; recordedBy: F.D. Fee; individualCount: 1; sex: male; lifeStage: adult; **Taxon:** scientificName: Microdonscauros Skevington and Locke; kingdom: Animalia; phylum: Arthropoda; class: Hexapoda; order: Diptera; family: Syrphidae; genus: Microdon; subgenus: Microdon; specificEpithet: scauros; taxonRank: species; scientificNameAuthorship: Skevington and Locke 2019; vernacularName: Big-footed Ant Fly; nomenclaturalStatus: new species; **Location:** country: United States of America; stateProvince: Pennsylvania; locality: Huntingdon County, Ridge & Nursery Roads, Barree Township; decimalLatitude: 40.642501; decimalLongitude: -77.908807; **Identification:** identifiedBy: Jeffrey H. Skevington; dateIdentified: 2017; **Event:** samplingProtocol: hand collected; eventDate: 1978-09-02; year: 1978; month: 9; day: 2; **Record Level:** language: en; institutionCode: ANSP; basisOfRecord: PreservedSpecimen
- **Type status:** Paratype. **Occurrence:** catalogNumber: Jeff_Skevington_Specimen44185; recordedBy: F.D. Fee; individualCount: 1; sex: male; lifeStage: adult; **Taxon:** scientificName: Microdonscauros Skevington and Locke; kingdom: Animalia; phylum: Arthropoda; class: Hexapoda; order: Diptera; family: Syrphidae; genus: Microdon; subgenus: Microdon; specificEpithet: scauros; taxonRank: species; scientificNameAuthorship: Skevington and Locke 2019; vernacularName: Big-footed Ant Fly; nomenclaturalStatus: new species; **Location:** country: United States of America; stateProvince: Pennsylvania; locality: Huntingdon County, Ridge & Nursery Roads, Barree Township; decimalLatitude: 40.642501; decimalLongitude: -77.908807; **Identification:** identifiedBy: Jeffrey H. Skevington; dateIdentified: 2017; **Event:** samplingProtocol: hand collected; eventDate: 1978-09-02; year: 1978; month: 9; day: 2; **Record Level:** language: en; institutionCode: ANSP; basisOfRecord: PreservedSpecimen
- **Type status:** Paratype. **Occurrence:** catalogNumber: Jeff_Skevington_Specimen44186; recordedBy: F.D. Fee; individualCount: 1; sex: male; lifeStage: adult; **Taxon:** scientificName: Microdonscauros Skevington and Locke; kingdom: Animalia; phylum: Arthropoda; class: Hexapoda; order: Diptera; family: Syrphidae; genus: Microdon; subgenus: Microdon; specificEpithet: scauros; taxonRank: species; scientificNameAuthorship: Skevington and Locke 2019; vernacularName: Big-footed Ant Fly; nomenclaturalStatus: new species; **Location:** country: United States of America; stateProvince: Pennsylvania; locality: Huntingdon County, Ridge & Nursery Roads, Barree Township; decimalLatitude: 40.642501; decimalLongitude: -77.908807; **Identification:** identifiedBy: Jeffrey H. Skevington; dateIdentified: 2017; **Event:** samplingProtocol: hand collected; eventDate: 1978-09-02; year: 1978; month: 9; day: 2; **Record Level:** language: en; institutionCode: ANSP; basisOfRecord: PreservedSpecimen
- **Type status:** Paratype. **Occurrence:** catalogNumber: Jeff_Skevington_Specimen44187; recordedBy: F.D. Fee; individualCount: 1; sex: male; lifeStage: adult; **Taxon:** scientificName: Microdonscauros Skevington and Locke; kingdom: Animalia; phylum: Arthropoda; class: Hexapoda; order: Diptera; family: Syrphidae; genus: Microdon; subgenus: Microdon; specificEpithet: scauros; taxonRank: species; scientificNameAuthorship: Skevington and Locke 2019; vernacularName: Big-footed Ant Fly; nomenclaturalStatus: new species; **Location:** country: United States of America; stateProvince: Pennsylvania; locality: Huntingdon County, Ridge & Nursery Roads, Barree Township; decimalLatitude: 40.642501; decimalLongitude: -77.908807; **Identification:** identifiedBy: Jeffrey H. Skevington; dateIdentified: 2017; **Event:** samplingProtocol: hand collected; eventDate: 1978-09-02; year: 1978; month: 9; day: 2; **Record Level:** language: en; institutionCode: ANSP; basisOfRecord: PreservedSpecimen
- **Type status:** Paratype. **Occurrence:** catalogNumber: Jeff_Skevington_Specimen44188; recordedBy: F.D. Fee; individualCount: 1; sex: male; lifeStage: adult; **Taxon:** scientificName: Microdonscauros Skevington and Locke; kingdom: Animalia; phylum: Arthropoda; class: Hexapoda; order: Diptera; family: Syrphidae; genus: Microdon; subgenus: Microdon; specificEpithet: scauros; taxonRank: species; scientificNameAuthorship: Skevington and Locke 2019; vernacularName: Big-footed Ant Fly; nomenclaturalStatus: new species; **Location:** country: United States of America; stateProvince: Pennsylvania; locality: Huntingdon County, Ridge & Nursery Roads, Barree Township; decimalLatitude: 40.642501; decimalLongitude: -77.908807; **Identification:** identifiedBy: Jeffrey H. Skevington; dateIdentified: 2017; **Event:** samplingProtocol: hand collected; eventDate: 1978-09-02; year: 1978; month: 9; day: 2; **Record Level:** language: en; institutionCode: CNC; basisOfRecord: PreservedSpecimen
- **Type status:** Paratype. **Occurrence:** catalogNumber: Jeff_Skevington_Specimen44199; recordedBy: F.D. Fee; individualCount: 1; sex: female; lifeStage: adult; **Taxon:** scientificName: Microdonscauros Skevington and Locke; kingdom: Animalia; phylum: Arthropoda; class: Hexapoda; order: Diptera; family: Syrphidae; genus: Microdon; subgenus: Microdon; specificEpithet: scauros; taxonRank: species; scientificNameAuthorship: Skevington and Locke 2019; vernacularName: Big-footed Ant Fly; nomenclaturalStatus: new species; **Location:** country: United States of America; stateProvince: Pennsylvania; locality: Huntingdon County, Ridge & Nursery Roads, Barree Township; decimalLatitude: 40.642501; decimalLongitude: -77.908807; **Identification:** identifiedBy: Jeffrey H. Skevington; dateIdentified: 2017; **Event:** samplingProtocol: hand collected; eventDate: 1978-09-02; year: 1978; month: 9; day: 2; habitat: veg., shrubby meadow; **Record Level:** language: en; institutionCode: CNC; basisOfRecord: PreservedSpecimen
- **Type status:** Paratype. **Occurrence:** catalogNumber: Jeff_Skevington_Specimen45193; recordedBy: F.D. Fee; individualCount: 1; sex: female; lifeStage: adult; otherCatalogNumbers: CCDB-26861; **Taxon:** scientificName: Microdonscauros Skevington and Locke; kingdom: Animalia; phylum: Arthropoda; class: Hexapoda; order: Diptera; family: Syrphidae; genus: Microdon; subgenus: Microdon; specificEpithet: scauros; taxonRank: species; scientificNameAuthorship: Skevington and Locke 2019; vernacularName: Big-footed Ant Fly; nomenclaturalStatus: new species; **Location:** country: United States of America; stateProvince: Pennsylvania; locality: Huntingdon County, Ridge & Nursery Roads, Barree Township; decimalLatitude: 40.642501; decimalLongitude: -77.908807; **Identification:** identifiedBy: Jeffrey H. Skevington; dateIdentified: 2017; **Event:** samplingProtocol: hand collected; eventDate: 1987-08-31; year: 1987; month: 8; day: 31; habitat: ex. low veg., dry weedy field; **Record Level:** language: en; institutionCode: ANSP; basisOfRecord: PreservedSpecimen
- **Type status:** Paratype. **Occurrence:** catalogNumber: Jeff_Skevington_Specimen44181; recordedBy: F.D. Fee; individualCount: 1; sex: male; lifeStage: adult; **Taxon:** scientificName: Microdonscauros Skevington and Locke; kingdom: Animalia; phylum: Arthropoda; class: Hexapoda; order: Diptera; family: Syrphidae; genus: Microdon; subgenus: Microdon; specificEpithet: scauros; taxonRank: species; scientificNameAuthorship: Skevington and Locke 2019; vernacularName: Big-footed Ant Fly; nomenclaturalStatus: new species; **Location:** country: United States of America; stateProvince: Pennsylvania; locality: Huntingdon County, Route 26, 1.1 km west of Whipple Dam State Park; decimalLatitude: 40.69385; decimalLongitude: -77.857447; **Identification:** identifiedBy: Jeffrey H. Skevington; dateIdentified: 2017; **Event:** samplingProtocol: hand collected; eventDate: 1988-08-27; year: 1988; month: 8; day: 27; habitat: veg., sedge meadow; **Record Level:** language: en; institutionCode: ANSP; basisOfRecord: PreservedSpecimen
- **Type status:** Paratype. **Occurrence:** catalogNumber: Jeff_Skevington_Specimen44182; recordedBy: F.D. Fee; individualCount: 1; sex: male; lifeStage: adult; **Taxon:** scientificName: Microdonscauros Skevington and Locke; kingdom: Animalia; phylum: Arthropoda; class: Hexapoda; order: Diptera; family: Syrphidae; genus: Microdon; subgenus: Microdon; specificEpithet: scauros; taxonRank: species; scientificNameAuthorship: Skevington and Locke 2019; vernacularName: Big-footed Ant Fly; nomenclaturalStatus: new species; **Location:** country: United States of America; stateProvince: Pennsylvania; locality: Huntingdon County, Route 26, 1.1 km west of Whipple Dam State Park; decimalLatitude: 40.69385; decimalLongitude: -77.857447; **Identification:** identifiedBy: Jeffrey H. Skevington; dateIdentified: 2017; **Event:** samplingProtocol: hand collected; eventDate: 1988-08-27; year: 1988; month: 8; day: 27; habitat: veg., sedge meadow; **Record Level:** language: en; institutionCode: ANSP; basisOfRecord: PreservedSpecimen
- **Type status:** Paratype. **Occurrence:** catalogNumber: Jeff_Skevington_Specimen44177; recordedBy: F.D. Fee; individualCount: 1; sex: male; lifeStage: adult; **Taxon:** scientificName: Microdonscauros Skevington and Locke; kingdom: Animalia; phylum: Arthropoda; class: Hexapoda; order: Diptera; family: Syrphidae; genus: Microdon; subgenus: Microdon; specificEpithet: scauros; taxonRank: species; scientificNameAuthorship: Skevington and Locke 2019; vernacularName: Big-footed Ant Fly; nomenclaturalStatus: new species; **Location:** country: United States of America; stateProvince: Pennsylvania; locality: Huntingdon County, Stone Valley Recreational Area; decimalLatitude: 40.659991; decimalLongitude: -77.919963; **Identification:** identifiedBy: Jeffrey H. Skevington; dateIdentified: 2017; **Event:** samplingProtocol: hand collected; eventDate: 1987-08-30; year: 1987; month: 8; day: 30; habitat: veg., shrubby meadow; **Record Level:** language: en; institutionCode: ANSP; basisOfRecord: PreservedSpecimen
- **Type status:** Paratype. **Occurrence:** catalogNumber: Jeff_Skevington_Specimen44178; recordedBy: F.D. Fee; individualCount: 1; sex: male; lifeStage: adult; **Taxon:** scientificName: Microdonscauros Skevington and Locke; kingdom: Animalia; phylum: Arthropoda; class: Hexapoda; order: Diptera; family: Syrphidae; genus: Microdon; subgenus: Microdon; specificEpithet: scauros; taxonRank: species; scientificNameAuthorship: Skevington and Locke 2019; vernacularName: Big-footed Ant Fly; nomenclaturalStatus: new species; **Location:** country: United States of America; stateProvince: Pennsylvania; locality: Huntingdon County, Stone Valley Recreational Area; decimalLatitude: 40.659991; decimalLongitude: -77.919963; **Identification:** identifiedBy: Jeffrey H. Skevington; dateIdentified: 2017; **Event:** samplingProtocol: hand collected; eventDate: 1987-08-30; year: 1987; month: 8; day: 30; habitat: veg., shrubby meadow; **Record Level:** language: en; institutionCode: ANSP; basisOfRecord: PreservedSpecimen
- **Type status:** Paratype. **Occurrence:** catalogNumber: Jeff_Skevington_Specimen44197; recordedBy: F.D. Fee; individualCount: 1; sex: female; lifeStage: adult; **Taxon:** scientificName: Microdonscauros Skevington and Locke; kingdom: Animalia; phylum: Arthropoda; class: Hexapoda; order: Diptera; family: Syrphidae; genus: Microdon; subgenus: Microdon; specificEpithet: scauros; taxonRank: species; scientificNameAuthorship: Skevington and Locke 2019; vernacularName: Big-footed Ant Fly; nomenclaturalStatus: new species; **Location:** country: United States of America; stateProvince: Pennsylvania; locality: Huntingdon County, Stone Valley Recreational Area; decimalLatitude: 40.659991; decimalLongitude: -77.919963; **Identification:** identifiedBy: Jeffrey H. Skevington; dateIdentified: 2017; **Event:** samplingProtocol: hand collected; eventDate: 1987-08-31; year: 1987; month: 8; day: 31; habitat: veg., shrubby meadow; **Record Level:** language: en; institutionCode: ANSP; basisOfRecord: PreservedSpecimen
- **Type status:** Paratype. **Occurrence:** catalogNumber: Jeff_Skevington_Specimen44198; recordedBy: F.D. Fee; individualCount: 1; sex: female; lifeStage: adult; **Taxon:** scientificName: Microdonscauros Skevington and Locke; kingdom: Animalia; phylum: Arthropoda; class: Hexapoda; order: Diptera; family: Syrphidae; genus: Microdon; subgenus: Microdon; specificEpithet: scauros; taxonRank: species; scientificNameAuthorship: Skevington and Locke 2019; vernacularName: Big-footed Ant Fly; nomenclaturalStatus: new species; **Location:** country: United States of America; stateProvince: Pennsylvania; locality: Huntingdon County, Stone Valley Recreational Area; decimalLatitude: 40.659991; decimalLongitude: -77.919963; **Identification:** identifiedBy: Jeffrey H. Skevington; dateIdentified: 2017; **Event:** samplingProtocol: hand collected; eventDate: 1987-08-31; year: 1987; month: 8; day: 31; habitat: veg., shrubby meadow; **Record Level:** language: en; institutionCode: ANSP; basisOfRecord: PreservedSpecimen
- **Type status:** Paratype. **Occurrence:** catalogNumber: Jeff_Skevington_Specimen29871; recordedBy: F.D. Fee; individualCount: 1; sex: male; lifeStage: adult; otherCatalogNumbers: CCDB-26861; **Taxon:** scientificName: Microdonscauros Skevington and Locke; kingdom: Animalia; phylum: Arthropoda; class: Hexapoda; order: Diptera; family: Syrphidae; genus: Microdon; subgenus: Microdon; specificEpithet: scauros; taxonRank: species; scientificNameAuthorship: Skevington and Locke 2019; vernacularName: Big-footed Ant Fly; nomenclaturalStatus: new species; **Location:** country: United States of America; stateProvince: Pennsylvania; locality: Huntingdon County, Whipple Dam State Park; decimalLatitude: 40.69385; decimalLongitude: -77.857447; **Identification:** identifiedBy: Jeffrey H. Skevington; dateIdentified: 2017; **Event:** samplingProtocol: hand collected; eventDate: 1986-08-31; year: 1986; month: 8; day: 31; habitat: ex: clearing among white pines/old field; **Record Level:** language: en; institutionCode: CNC; basisOfRecord: PreservedSpecimen
- **Type status:** Paratype. **Occurrence:** catalogNumber: Jeff_Skevington_Specimen29872; recordedBy: F.D. Fee; individualCount: 1; sex: male; lifeStage: adult; **Taxon:** scientificName: Microdonscauros Skevington and Locke; kingdom: Animalia; phylum: Arthropoda; class: Hexapoda; order: Diptera; family: Syrphidae; genus: Microdon; subgenus: Microdon; specificEpithet: scauros; taxonRank: species; scientificNameAuthorship: Skevington and Locke 2019; vernacularName: Big-footed Ant Fly; nomenclaturalStatus: new species; **Location:** country: United States of America; stateProvince: Pennsylvania; locality: Huntingdon County, Whipple Dam State Park; decimalLatitude: 40.69385; decimalLongitude: -77.857447; **Identification:** identifiedBy: Jeffrey H. Skevington; dateIdentified: 2017; **Event:** samplingProtocol: hand collected; eventDate: 1986-08-31; year: 1986; month: 8; day: 31; habitat: ex: clearing among white pines/old field; **Record Level:** language: en; institutionCode: ANSP; basisOfRecord: PreservedSpecimen
- **Type status:** Paratype. **Occurrence:** catalogNumber: Jeff_Skevington_Specimen44167; recordedBy: F.D. Fee; individualCount: 1; sex: male; lifeStage: adult; **Taxon:** scientificName: Microdonscauros Skevington and Locke; kingdom: Animalia; phylum: Arthropoda; class: Hexapoda; order: Diptera; family: Syrphidae; genus: Microdon; subgenus: Microdon; specificEpithet: scauros; taxonRank: species; scientificNameAuthorship: Skevington and Locke 2019; vernacularName: Big-footed Ant Fly; nomenclaturalStatus: new species; **Location:** country: United States of America; stateProvince: Pennsylvania; locality: Huntingdon County, Whipple Dam State Park; decimalLatitude: 40.69385; decimalLongitude: -77.857447; **Identification:** identifiedBy: Jeffrey H. Skevington; dateIdentified: 2017; **Event:** samplingProtocol: hand collected; eventDate: 1986-08-31; year: 1986; month: 8; day: 31; habitat: old field clearing/white pines; **Record Level:** language: en; institutionCode: ANSP; basisOfRecord: PreservedSpecimen
- **Type status:** Paratype. **Occurrence:** catalogNumber: Jeff_Skevington_Specimen44168; recordedBy: F.D. Fee; individualCount: 1; sex: male; lifeStage: adult; **Taxon:** scientificName: Microdonscauros Skevington and Locke; kingdom: Animalia; phylum: Arthropoda; class: Hexapoda; order: Diptera; family: Syrphidae; genus: Microdon; subgenus: Microdon; specificEpithet: scauros; taxonRank: species; scientificNameAuthorship: Skevington and Locke 2019; vernacularName: Big-footed Ant Fly; nomenclaturalStatus: new species; **Location:** country: United States of America; stateProvince: Pennsylvania; locality: Huntingdon County, Whipple Dam State Park; decimalLatitude: 40.69385; decimalLongitude: -77.857447; **Identification:** identifiedBy: Jeffrey H. Skevington; dateIdentified: 2017; **Event:** samplingProtocol: hand collected; eventDate: 1986-08-31; year: 1986; month: 8; day: 31; habitat: old field clearing/white pines; **Record Level:** language: en; institutionCode: ANSP; basisOfRecord: PreservedSpecimen
- **Type status:** Paratype. **Occurrence:** catalogNumber: Jeff_Skevington_Specimen44170; recordedBy: F.D. Fee; individualCount: 1; sex: male; lifeStage: adult; **Taxon:** scientificName: Microdonscauros Skevington and Locke; kingdom: Animalia; phylum: Arthropoda; class: Hexapoda; order: Diptera; family: Syrphidae; genus: Microdon; subgenus: Microdon; specificEpithet: scauros; taxonRank: species; scientificNameAuthorship: Skevington and Locke 2019; vernacularName: Big-footed Ant Fly; nomenclaturalStatus: new species; **Location:** country: United States of America; stateProvince: Pennsylvania; locality: Huntingdon County, Whipple Dam State Park; decimalLatitude: 40.69385; decimalLongitude: -77.857447; **Identification:** identifiedBy: Jeffrey H. Skevington; dateIdentified: 2017; **Event:** samplingProtocol: hand collected; eventDate: 1986-09-03; year: 1986; month: 9; day: 3; habitat: old field, clearing, white pines; **Record Level:** language: en; institutionCode: ANSP; basisOfRecord: PreservedSpecimen
- **Type status:** Paratype. **Occurrence:** catalogNumber: Jeff_Skevington_Specimen44171; recordedBy: F.D. Fee; individualCount: 1; sex: male; lifeStage: adult; **Taxon:** scientificName: Microdonscauros Skevington and Locke; kingdom: Animalia; phylum: Arthropoda; class: Hexapoda; order: Diptera; family: Syrphidae; genus: Microdon; subgenus: Microdon; specificEpithet: scauros; taxonRank: species; scientificNameAuthorship: Skevington and Locke 2019; vernacularName: Big-footed Ant Fly; nomenclaturalStatus: new species; **Location:** country: United States of America; stateProvince: Pennsylvania; locality: Huntingdon County, Whipple Dam State Park; decimalLatitude: 40.69385; decimalLongitude: -77.857447; **Identification:** identifiedBy: Jeffrey H. Skevington; dateIdentified: 2017; **Event:** samplingProtocol: hand collected; eventDate: 1986-09-06; year: 1986; month: 9; day: 6; habitat: low veg., old field; **Record Level:** language: en; institutionCode: ANSP; basisOfRecord: PreservedSpecimen
- **Type status:** Paratype. **Occurrence:** catalogNumber: Jeff_Skevington_Specimen44172; recordedBy: F.D. Fee; individualCount: 1; sex: male; lifeStage: adult; **Taxon:** scientificName: Microdonscauros Skevington and Locke; kingdom: Animalia; phylum: Arthropoda; class: Hexapoda; order: Diptera; family: Syrphidae; genus: Microdon; subgenus: Microdon; specificEpithet: scauros; taxonRank: species; scientificNameAuthorship: Skevington and Locke 2019; vernacularName: Big-footed Ant Fly; nomenclaturalStatus: new species; **Location:** country: United States of America; stateProvince: Pennsylvania; locality: Huntingdon County, Whipple Dam State Park; decimalLatitude: 40.69385; decimalLongitude: -77.857447; **Identification:** identifiedBy: Jeffrey H. Skevington; dateIdentified: 2017; **Event:** samplingProtocol: hand collected; eventDate: 1986-09-06; year: 1986; month: 9; day: 6; habitat: low veg., old field; **Record Level:** language: en; institutionCode: ANSP; basisOfRecord: PreservedSpecimen
- **Type status:** Paratype. **Occurrence:** catalogNumber: Jeff_Skevington_Specimen44173; recordedBy: F.D. Fee; individualCount: 1; sex: male; lifeStage: adult; **Taxon:** scientificName: Microdonscauros Skevington and Locke; kingdom: Animalia; phylum: Arthropoda; class: Hexapoda; order: Diptera; family: Syrphidae; genus: Microdon; subgenus: Microdon; specificEpithet: scauros; taxonRank: species; scientificNameAuthorship: Skevington and Locke 2019; vernacularName: Big-footed Ant Fly; nomenclaturalStatus: new species; **Location:** country: United States of America; stateProvince: Pennsylvania; locality: Huntingdon County, Whipple Dam State Park; decimalLatitude: 40.69385; decimalLongitude: -77.857447; **Identification:** identifiedBy: Jeffrey H. Skevington; dateIdentified: 2017; **Event:** samplingProtocol: hand collected; eventDate: 1986-09-06; year: 1986; month: 9; day: 6; habitat: clearing-white pines-old field; **Record Level:** language: en; institutionCode: ANSP; basisOfRecord: PreservedSpecimen
- **Type status:** Paratype. **Occurrence:** catalogNumber: Jeff_Skevington_Specimen44174; recordedBy: F.D. Fee; individualCount: 1; sex: male; lifeStage: adult; **Taxon:** scientificName: Microdonscauros Skevington and Locke; kingdom: Animalia; phylum: Arthropoda; class: Hexapoda; order: Diptera; family: Syrphidae; genus: Microdon; subgenus: Microdon; specificEpithet: scauros; taxonRank: species; scientificNameAuthorship: Skevington and Locke 2019; vernacularName: Big-footed Ant Fly; nomenclaturalStatus: new species; **Location:** country: United States of America; stateProvince: Pennsylvania; locality: Huntingdon County, Whipple Dam State Park; decimalLatitude: 40.69385; decimalLongitude: -77.857447; **Identification:** identifiedBy: Jeffrey H. Skevington; dateIdentified: 2017; **Event:** samplingProtocol: hand collected; eventDate: 1986-09-11; year: 1986; month: 9; day: 11; habitat: Perching-grass-old field; **Record Level:** language: en; institutionCode: ANSP; basisOfRecord: PreservedSpecimen
- **Type status:** Paratype. **Occurrence:** catalogNumber: Jeff_Skevington_Specimen44175; recordedBy: F.D. Fee; individualCount: 1; sex: male; lifeStage: adult; **Taxon:** scientificName: Microdonscauros Skevington and Locke; kingdom: Animalia; phylum: Arthropoda; class: Hexapoda; order: Diptera; family: Syrphidae; genus: Microdon; subgenus: Microdon; specificEpithet: scauros; taxonRank: species; scientificNameAuthorship: Skevington and Locke 2019; vernacularName: Big-footed Ant Fly; nomenclaturalStatus: new species; **Location:** country: United States of America; stateProvince: Pennsylvania; locality: Huntingdon County, Whipple Dam State Park; decimalLatitude: 40.69385; decimalLongitude: -77.857447; **Identification:** identifiedBy: Jeffrey H. Skevington; dateIdentified: 2017; **Event:** samplingProtocol: hand collected; eventDate: 1986-09-13; year: 1986; month: 9; day: 13; habitat: clearing, old field; **Record Level:** language: en; institutionCode: USNM; basisOfRecord: PreservedSpecimen
- **Type status:** Paratype. **Occurrence:** catalogNumber: Jeff_Skevington_Specimen44183; recordedBy: F.D. Fee; individualCount: 1; sex: male; lifeStage: adult; **Taxon:** scientificName: Microdonscauros Skevington and Locke; kingdom: Animalia; phylum: Arthropoda; class: Hexapoda; order: Diptera; family: Syrphidae; genus: Microdon; subgenus: Microdon; specificEpithet: scauros; taxonRank: species; scientificNameAuthorship: Skevington and Locke 2019; vernacularName: Big-footed Ant Fly; nomenclaturalStatus: new species; **Location:** country: United States of America; stateProvince: Pennsylvania; locality: Huntingdon County, Whipple Dam State Park; decimalLatitude: 40.69385; decimalLongitude: -77.857447; **Identification:** identifiedBy: Jeffrey H. Skevington; dateIdentified: 2017; **Event:** samplingProtocol: hand collected; eventDate: 1989-07-29; year: 1989; month: 7; day: 29; **Record Level:** language: en; institutionCode: CNC; basisOfRecord: PreservedSpecimen
- **Type status:** Paratype. **Occurrence:** catalogNumber: Jeff_Skevington_Specimen44189; recordedBy: F.D. Fee; individualCount: 1; sex: female; lifeStage: adult; **Taxon:** scientificName: Microdonscauros Skevington and Locke; kingdom: Animalia; phylum: Arthropoda; class: Hexapoda; order: Diptera; family: Syrphidae; genus: Microdon; subgenus: Microdon; specificEpithet: scauros; taxonRank: species; scientificNameAuthorship: Skevington and Locke 2019; vernacularName: Big-footed Ant Fly; nomenclaturalStatus: new species; **Location:** country: United States of America; stateProvince: Pennsylvania; locality: Huntingdon County, Whipple Dam State Park; decimalLatitude: 40.69385; decimalLongitude: -77.857447; **Identification:** identifiedBy: Jeffrey H. Skevington; dateIdentified: 2017; **Event:** samplingProtocol: hand collected; eventDate: 1986-08-31; year: 1986; month: 8; day: 31; habitat: clearing, white pine/old field; **Record Level:** language: en; institutionCode: CNC; basisOfRecord: PreservedSpecimen
- **Type status:** Paratype. **Occurrence:** catalogNumber: Jeff_Skevington_Specimen44190; recordedBy: F.D. Fee; individualCount: 1; sex: female; lifeStage: adult; **Taxon:** scientificName: Microdonscauros Skevington and Locke; kingdom: Animalia; phylum: Arthropoda; class: Hexapoda; order: Diptera; family: Syrphidae; genus: Microdon; subgenus: Microdon; specificEpithet: scauros; taxonRank: species; scientificNameAuthorship: Skevington and Locke 2019; vernacularName: Big-footed Ant Fly; nomenclaturalStatus: new species; **Location:** country: United States of America; stateProvince: Pennsylvania; locality: Huntingdon County, Whipple Dam State Park; decimalLatitude: 40.69385; decimalLongitude: -77.857447; **Identification:** identifiedBy: Jeffrey H. Skevington; dateIdentified: 2017; **Event:** samplingProtocol: hand collected; eventDate: 1986-09-04; year: 1986; month: 9; day: 4; habitat: open grassy old field; **Record Level:** language: en; institutionCode: USNM; basisOfRecord: PreservedSpecimen
- **Type status:** Paratype. **Occurrence:** catalogNumber: Jeff_Skevington_Specimen44191; recordedBy: F.D. Fee; individualCount: 1; sex: female; lifeStage: adult; **Taxon:** scientificName: Microdonscauros Skevington and Locke; kingdom: Animalia; phylum: Arthropoda; class: Hexapoda; order: Diptera; family: Syrphidae; genus: Microdon; subgenus: Microdon; specificEpithet: scauros; taxonRank: species; scientificNameAuthorship: Skevington and Locke 2019; vernacularName: Big-footed Ant Fly; nomenclaturalStatus: new species; **Location:** country: United States of America; stateProvince: Pennsylvania; locality: Huntingdon County, Whipple Dam State Park; decimalLatitude: 40.69385; decimalLongitude: -77.857447; **Identification:** identifiedBy: Jeffrey H. Skevington; dateIdentified: 2017; **Event:** samplingProtocol: hand collected; eventDate: 1986-09-06; year: 1986; month: 9; day: 6; habitat: low veg., old field; **Record Level:** language: en; institutionCode: ANSP; basisOfRecord: PreservedSpecimen
- **Type status:** Paratype. **Occurrence:** catalogNumber: Jeff_Skevington_Specimen44192; recordedBy: F.D. Fee; individualCount: 1; sex: female; lifeStage: adult; **Taxon:** scientificName: Microdonscauros Skevington and Locke; kingdom: Animalia; phylum: Arthropoda; class: Hexapoda; order: Diptera; family: Syrphidae; genus: Microdon; subgenus: Microdon; specificEpithet: scauros; taxonRank: species; scientificNameAuthorship: Skevington and Locke 2019; vernacularName: Big-footed Ant Fly; nomenclaturalStatus: new species; **Location:** country: United States of America; stateProvince: Pennsylvania; locality: Huntingdon County, Whipple Dam State Park; decimalLatitude: 40.69385; decimalLongitude: -77.857447; **Identification:** identifiedBy: Jeffrey H. Skevington; dateIdentified: 2017; **Event:** samplingProtocol: hand collected; eventDate: 1986-09-06; year: 1986; month: 9; day: 6; habitat: low veg., old field; **Record Level:** language: en; institutionCode: ANSP; basisOfRecord: PreservedSpecimen
- **Type status:** Paratype. **Occurrence:** catalogNumber: Jeff_Skevington_Specimen44193; recordedBy: F.D. Fee; individualCount: 1; sex: female; lifeStage: adult; **Taxon:** scientificName: Microdonscauros Skevington and Locke; kingdom: Animalia; phylum: Arthropoda; class: Hexapoda; order: Diptera; family: Syrphidae; genus: Microdon; subgenus: Microdon; specificEpithet: scauros; taxonRank: species; scientificNameAuthorship: Skevington and Locke 2019; vernacularName: Big-footed Ant Fly; nomenclaturalStatus: new species; **Location:** country: United States of America; stateProvince: Pennsylvania; locality: Huntingdon County, Whipple Dam State Park; decimalLatitude: 40.69385; decimalLongitude: -77.857447; **Identification:** identifiedBy: Jeffrey H. Skevington; dateIdentified: 2017; **Event:** samplingProtocol: hand collected; eventDate: 1986-09-06; year: 1986; month: 9; day: 6; habitat: open grassy mowed area; **Record Level:** language: en; institutionCode: ANSP; basisOfRecord: PreservedSpecimen
- **Type status:** Paratype. **Occurrence:** catalogNumber: Jeff_Skevington_Specimen44194; recordedBy: F.D. Fee; individualCount: 1; sex: female; lifeStage: adult; **Taxon:** scientificName: Microdonscauros Skevington and Locke; kingdom: Animalia; phylum: Arthropoda; class: Hexapoda; order: Diptera; family: Syrphidae; genus: Microdon; subgenus: Microdon; specificEpithet: scauros; taxonRank: species; scientificNameAuthorship: Skevington and Locke 2019; vernacularName: Big-footed Ant Fly; nomenclaturalStatus: new species; **Location:** country: United States of America; stateProvince: Pennsylvania; locality: Huntingdon County, Whipple Dam State Park; decimalLatitude: 40.69385; decimalLongitude: -77.857447; **Identification:** identifiedBy: Jeffrey H. Skevington; dateIdentified: 2017; **Event:** samplingProtocol: hand collected; eventDate: 1986-09-06; year: 1986; month: 9; day: 6; habitat: low veg., old field; **Record Level:** language: en; institutionCode: ANSP; basisOfRecord: PreservedSpecimen
- **Type status:** Paratype. **Occurrence:** catalogNumber: Jeff_Skevington_Specimen44195; recordedBy: F.D. Fee; individualCount: 1; sex: female; lifeStage: adult; **Taxon:** scientificName: Microdonscauros Skevington and Locke; kingdom: Animalia; phylum: Arthropoda; class: Hexapoda; order: Diptera; family: Syrphidae; genus: Microdon; subgenus: Microdon; specificEpithet: scauros; taxonRank: species; scientificNameAuthorship: Skevington and Locke 2019; vernacularName: Big-footed Ant Fly; nomenclaturalStatus: new species; **Location:** country: United States of America; stateProvince: Pennsylvania; locality: Huntingdon County, Whipple Dam State Park; decimalLatitude: 40.69385; decimalLongitude: -77.857447; **Identification:** identifiedBy: Jeffrey H. Skevington; dateIdentified: 2017; **Event:** samplingProtocol: hand collected; eventDate: 1986-09-11; year: 1986; month: 9; day: 11; habitat: Perching-grasses-old field; **Record Level:** language: en; institutionCode: ANSP; basisOfRecord: PreservedSpecimen
- **Type status:** Paratype. **Occurrence:** catalogNumber: Jeff_Skevington_Specimen44196; recordedBy: F.D. Fee; individualCount: 1; sex: female; lifeStage: adult; **Taxon:** scientificName: Microdonscauros Skevington and Locke; kingdom: Animalia; phylum: Arthropoda; class: Hexapoda; order: Diptera; family: Syrphidae; genus: Microdon; subgenus: Microdon; specificEpithet: scauros; taxonRank: species; scientificNameAuthorship: Skevington and Locke 2019; vernacularName: Big-footed Ant Fly; nomenclaturalStatus: new species; **Location:** country: United States of America; stateProvince: Pennsylvania; locality: Huntingdon County, Whipple Dam State Park; decimalLatitude: 40.69385; decimalLongitude: -77.857447; **Identification:** identifiedBy: Jeffrey H. Skevington; dateIdentified: 2017; **Event:** samplingProtocol: hand collected; eventDate: 1986-09-13; year: 1986; month: 9; day: 13; habitat: clearing, old field; **Record Level:** language: en; institutionCode: ANSP; basisOfRecord: PreservedSpecimen
- **Type status:** Paratype. **Occurrence:** catalogNumber: Jeff_Skevington_Specimen18258; recordedBy: F.D. Fee; individualCount: 1; sex: female; lifeStage: adult; **Taxon:** scientificName: Microdonscauros Skevington and Locke; kingdom: Animalia; phylum: Arthropoda; class: Hexapoda; order: Diptera; family: Syrphidae; genus: Microdon; subgenus: Microdon; specificEpithet: scauros; taxonRank: species; scientificNameAuthorship: Skevington and Locke 2019; vernacularName: Big-footed Ant Fly; nomenclaturalStatus: new species; **Location:** country: United States of America; stateProvince: Pennsylvania; locality: Huntington County, Route 26, 1.1 km west of Whipple Dam State Park; decimalLatitude: 40.684219; decimalLongitude: -77.863903; **Identification:** identifiedBy: Jeffrey H. Skevington; dateIdentified: 2017; **Event:** samplingProtocol: hand collected; eventDate: 1988-09-01; year: 1988; month: 9; day: 1; habitat: ex. vegetation, dry weedy hillside; **Record Level:** language: en; institutionCode: ANSP; basisOfRecord: PreservedSpecimen

#### Description

**Size**: Body length 7.5 to 11.9 mm; wing length 4.7 to 7.2 mm

**Male: Head**: Brown, shining, entirely white pilose; occiput weakly grey pollinose, white pilose; scape pale brown, pedicel and postpedicel contrasting dark brown; postpedicel with a short sensory groove (about 0.1x length of postpedicel) below base of arista; antennal ratio (scape:pedicel:postpedicel) 4:1:7; eye bare; dichoptic, with eyes separated by 0.6-0.7x length of scape.

**Thorax**: Brown, shining; scutum and scutellum entirely white pilose; scutellum with prominent pair of apical, inward-pointing calcars; postpronotum white pilose; anterior anepisternum mostly bare with white pile only adjacent to posterior anepisternum, posterior anepisternum entirely white pilose, bare ventrally; katepisternum mostly shining brown with white pile only on posterodorsal corner and in a small ventral patch between fore and mid coxae; katepisternum bare and shining ventrally, pilose dorsally; meron and katepimeron bare, shining brown; anepimeron and anatergum sparsely pilose, dull; halter pale yellow; calypter pale yellow with white fringe; femora, coxae and trochanters dark brown, white pilose; tibiae pale yellowish-brown with dark brown medial band, white pilose; pro- and mesotarsi pale yellowish-brown with white pile dorsally and shorter golden pile ventrally and laterally; metabasitarsis dark brown, swollen and cylindrical; other metatarsi similar to those of pro- and mesolegs; wing light brownish, microtrichose except for narrow bare area along leading edges of bm and br.

**Abdomen**: Entirely dark brown dorsally and white pilose; sternites and terminalia paler brown, white pilose.

**Genitalia**: Epandrium compact, similar in length to surstylus; cercus subquadrate, with apex rounded and slightly wider at base; surstylus broad base, finger-shaped distally, with broad short spines ventroapically; phallus broad basally, ending in 2 stout tubules; apical part of hypandrium narrow, leaf-like; ejaculatory apodeme flat, rounded.

**Female**: Similar to male, differing as follows: Antennal ratio 3-4:1:5-6; terminalia dark brown except cerci paler.

See Fig. 8 & Fig. 9.

#### Diagnosis

Dark with prominent inward-pointing calcars on the scutellum, swollen metabasitarsus particularly evident on the male, postpedicel longer than scape, wings shorter than *M.trisits* and completely microtrichose (including cell bm).

#### Etymology

Based on the Greek word *skauros*, for having large and swollen ankles, with reference to the swollen metabasitarsus of this species.

#### Distribution

Specimens are known from five sites in a small area of Pennsylvania and Maryland and were all collected by Frank Fee.

#### Ecology

This species appears to be active late in the season and is known to fly from late August to mid-September. One specimen was found flying earlier, in late July. This species is known from a small area of the north-eastern United States and is apparently rare. The known specimens were found in old fields and sedge meadows with adjacent pine-dominated forests or marshland.

#### Taxon discussion

Most closely resembles *Microdontristis* Loew, 1864. *Microdontristis* can be distinguished by prominent, outward-pointing calcars on the scutellum, metabasitarsus only slightly expanded (not prominently swollen), orange tibiae contrast with dark femora, postpedicel as long or longer than scape, wing with cell bm bare posterobasally. DNA was obtained from a single specimen (Table 2). Although morphologically most similar to *M.tristis* , it is most similar genetically to *M.globosus* (Fabricius 1805). *Microdonscauros* and *M.globosus*, while genetically similar, are quite different morphologically and clearly distinguished by Skevington et al. (2019).

#### Common Name

The common name given to the species by Skevington et al. (2019) is Big-footed Ant Fly.

### Mixogaster fattigi

Locke, Skevington and Greene sp. n.

D38635B4921957D798C0EB43A26D4E6A

urn:lsid:zoobank.org:act:42F8F983-1C7B-49BD-B015-8C50A2CB6884

#### Materials

- **Type status:** Holotype. **Occurrence:** catalogNumber: Jeff_Skevington_Specimen17086; recordedBy: P.W. Fattig; individualCount: 1; sex: male; lifeStage: adult; **Taxon:** scientificName: Mixogasterfattigi Locke, Skevington and Greene; kingdom: Animalia; phylum: Arthropoda; class: Hexapoda; order: Diptera; family: Syrphidae; genus: Mixogaster; specificEpithet: fattigi; taxonRank: species; scientificNameAuthorship: Locke, Skevington and Greene 2019; vernacularName: Fattig's Ant Fly; nomenclaturalStatus: new species; **Location:** country: U.S.A.; stateProvince: Georgia; locality: Atlanta; decimalLatitude: 33.75; decimalLongitude: -84.383333; **Identification:** identifiedBy: J.H. Skevington; **Event:** eventDate: 1941-07-08; year: 1941; month: 7; day: 8; verbatimEventDate: 8.vii.1941; **Record Level:** language: en; institutionCode: USNM; basisOfRecord: PreservedSpecimen
- **Type status:** Paratype. **Occurrence:** catalogNumber: CNC1129651; recordedBy: G. Steck and B. Sutton; individualCount: 1; sex: male; lifeStage: adult; **Taxon:** scientificName: Mixogasterfattigi Locke, Skevington and Greene; kingdom: Animalia; phylum: Arthropoda; class: Hexapoda; order: Diptera; family: Syrphidae; genus: Mixogaster; specificEpithet: fattigi; taxonRank: species; scientificNameAuthorship: Locke, Skevington and Greene 2019; vernacularName: Fattig's Ant Fly; nomenclaturalStatus: new species; **Location:** country: U.S.A.; stateProvince: Tennessee; locality: Great Smoky Mountains National Park, Cades Cove, Parson's Branch Road; decimalLatitude: 35.6; decimalLongitude: -83.85; **Identification:** identifiedBy: J.H. Skevington; **Event:** samplingProtocol: Malaise trap; eventDate: 1999-07-10; year: 1999; month: 7; day: 10; verbatimEventDate: 10-12.vii.1999; **Record Level:** language: en; institutionCode: CNC; basisOfRecord: PreservedSpecimen
- **Type status:** Paratype. **Occurrence:** catalogNumber: CNC1129652; recordedBy: G. Steck and B. Sutton; individualCount: 1; sex: male; lifeStage: adult; **Taxon:** scientificName: Mixogasterfattigi Locke, Skevington and Greene; kingdom: Animalia; phylum: Arthropoda; class: Hexapoda; order: Diptera; family: Syrphidae; genus: Mixogaster; specificEpithet: fattigi; taxonRank: species; scientificNameAuthorship: Locke, Skevington and Greene 2019; vernacularName: Fattig's Ant Fly; nomenclaturalStatus: new species; **Location:** country: U.S.A.; stateProvince: Tennessee; locality: Great Smoky Mountains National Park, Cades Cove, Parson\'s Branch Road; decimalLatitude: 35.6; decimalLongitude: -83.85; **Identification:** identifiedBy: J.H. Skevington; **Event:** samplingProtocol: Malaise trap; eventDate: 1999-07-10/12; year: 1999; month: 7; day: 10; verbatimEventDate: 10-12.vii.1999; **Record Level:** language: en; institutionCode: CNC; basisOfRecord: PreservedSpecimen
- **Type status:** Paratype. **Occurrence:** catalogNumber: CNC1129653; recordedBy: G. Steck and B. Sutton; individualCount: 1; sex: male; lifeStage: adult; **Taxon:** scientificName: Mixogasterfattigi Locke, Skevington and Greene; kingdom: Animalia; phylum: Arthropoda; class: Hexapoda; order: Diptera; family: Syrphidae; genus: Mixogaster; specificEpithet: fattigi; taxonRank: species; scientificNameAuthorship: Locke, Skevington and Greene 2019; vernacularName: Fattig's Ant Fly; nomenclaturalStatus: new species; **Location:** country: U.S.A.; stateProvince: Tennessee; locality: Great Smoky Mountains National Park, Cades Cove, Parson\'s Branch Road; decimalLatitude: 35.6; decimalLongitude: -83.85; **Identification:** identifiedBy: J.H. Skevington; **Event:** samplingProtocol: Malaise trap; eventDate: 1999-07-10/12; year: 1999; month: 7; day: 10; verbatimEventDate: 10-12.vii.1999; **Record Level:** language: en; institutionCode: CNC; basisOfRecord: PreservedSpecimen
- **Type status:** Paratype. **Occurrence:** catalogNumber: CNC1129654; recordedBy: G. Steck and B. Sutton; individualCount: 1; sex: male; lifeStage: adult; **Taxon:** scientificName: Mixogasterfattigi Locke, Skevington and Greene; kingdom: Animalia; phylum: Arthropoda; class: Hexapoda; order: Diptera; family: Syrphidae; genus: Mixogaster; specificEpithet: fattigi; taxonRank: species; scientificNameAuthorship: Locke, Skevington and Greene 2019; vernacularName: Fattig's Ant Fly; nomenclaturalStatus: new species; **Location:** country: U.S.A.; stateProvince: Tennessee; locality: Great Smoky Mountains National Park, Cades Cove, Parson\'s Branch Road; decimalLatitude: 35.6; decimalLongitude: -83.85; **Identification:** identifiedBy: J.H. Skevington; **Event:** samplingProtocol: Malaise trap; eventDate: 1999-07-10/12; year: 1999; month: 7; day: 10; verbatimEventDate: 10-12.vii.1999; **Record Level:** language: en; institutionCode: CNC; basisOfRecord: PreservedSpecimen
- **Type status:** Paratype. **Occurrence:** catalogNumber: CNC_Diptera102833; recordedBy: D.Shanek; individualCount: 1; sex: male; lifeStage: adult; **Taxon:** scientificName: Mixogasterfattigi Locke, Skevington and Greene; kingdom: Animalia; phylum: Arthropoda; class: Hexapoda; order: Diptera; family: Syrphidae; genus: Mixogaster; specificEpithet: fattigi; taxonRank: species; scientificNameAuthorship: Locke, Skevington and Greene 2019; vernacularName: Fattig's Ant Fly; nomenclaturalStatus: new species; **Location:** country: U.S.A.; stateProvince: Louisiana; locality: Bayou Chicot, Evangeline Parish; decimalLatitude: 30.815558; decimalLongitude: -92.350645; **Identification:** identifiedBy: J.H. Skevington; **Event:** eventDate: 1971-09-17; year: 1971; month: 8; day: 17; verbatimEventDate: 17.viii.1971; **Record Level:** language: en; institutionCode: CNC; basisOfRecord: PreservedSpecimen
- **Type status:** Paratype. **Occurrence:** catalogNumber: CNC_Diptera102834; recordedBy: D.Shanek; individualCount: 1; sex: male; lifeStage: adult; **Taxon:** scientificName: Mixogasterfattigi Locke, Skevington and Greene; kingdom: Animalia; phylum: Arthropoda; class: Hexapoda; order: Diptera; family: Syrphidae; genus: Mixogaster; specificEpithet: fattigi; taxonRank: species; scientificNameAuthorship: Locke, Skevington and Greene 2019; vernacularName: Fattig's Ant Fly; nomenclaturalStatus: new species; **Location:** country: U.S.A.; stateProvince: Louisiana; locality: Bayou Chicot, Evangeline Parish; decimalLatitude: 30.815558; decimalLongitude: -92.350645; **Identification:** identifiedBy: J.H. Skevington; **Event:** eventDate: 1971-09-17; year: 1971; month: 8; day: 17; verbatimEventDate: 17.viii.1971; **Record Level:** language: en; institutionCode: CNC; basisOfRecord: PreservedSpecimen
- **Type status:** Paratype. **Occurrence:** catalogNumber: CNC_Diptera92828; recordedBy: P.W. Fattig; individualCount: 1; sex: male; lifeStage: adult; **Taxon:** scientificName: Mixogasterfattigi Locke, Skevington and Greene; kingdom: Animalia; phylum: Arthropoda; class: Hexapoda; order: Diptera; family: Syrphidae; genus: Mixogaster; specificEpithet: fattigi; taxonRank: species; scientificNameAuthorship: Locke, Skevington and Greene; vernacularName: Fattig's Ant Fly; nomenclaturalStatus: new species; **Location:** country: U.S.A.; stateProvince: Georgia; locality: Atlanta; decimalLatitude: 33.75; decimalLongitude: -84.383333; **Identification:** identifiedBy: J.H. Skevington; **Event:** eventDate: 1941-06-26; year: 1941; month: 6; day: 26; verbatimEventDate: 26.vi.1941; **Record Level:** language: en; institutionCode: CNC; basisOfRecord: PreservedSpecimen
- **Type status:** Paratype. **Occurrence:** catalogNumber: CNC_Diptera92829; recordedBy: P.W. Fattig; individualCount: 1; sex: male; lifeStage: adult; **Taxon:** scientificName: Mixogasterfattigi Locke, Skevington and Greene; kingdom: Animalia; phylum: Arthropoda; class: Hexapoda; order: Diptera; family: Syrphidae; genus: Mixogaster; specificEpithet: fattigi; taxonRank: species; scientificNameAuthorship: Locke, Skevington and Greene 2019; vernacularName: Fattig's Ant Fly; nomenclaturalStatus: new species; **Location:** country: U.S.A.; stateProvince: Georgia; locality: Atlanta; decimalLatitude: 33.75; decimalLongitude: -84.383333; **Identification:** identifiedBy: J.H. Skevington; **Event:** eventDate: 1942-06-30; year: 1942; month: 6; day: 30; verbatimEventDate: 30.vi.1942; **Record Level:** language: en; institutionCode: CNC; basisOfRecord: PreservedSpecimen
- **Type status:** Paratype. **Occurrence:** catalogNumber: CNC_Diptera92831; recordedBy: P.W. Fattig; individualCount: 1; sex: male; lifeStage: adult; **Taxon:** scientificName: Mixogasterfattigi Locke, Skevington and Greene; kingdom: Animalia; phylum: Arthropoda; class: Hexapoda; order: Diptera; family: Syrphidae; genus: Mixogaster; specificEpithet: fattigi; taxonRank: species; scientificNameAuthorship: Locke, Skevington and Greene 2019; vernacularName: Fattig's Ant Fly; nomenclaturalStatus: new species; **Location:** country: U.S.A.; stateProvince: Georgia; locality: Atlanta; decimalLatitude: 33.75; decimalLongitude: -84.383333; **Identification:** identifiedBy: J.H. Skevington; **Event:** eventDate: 1942-07-15; year: 1942; month: 7; day: 15; verbatimEventDate: 15.vii.1942; **Record Level:** language: en; institutionCode: CNC; basisOfRecord: PreservedSpecimen
- **Type status:** Paratype. **Occurrence:** catalogNumber: Jeff_Skevington_Specimen25513; recordedBy: W. Godwin; individualCount: 1; sex: male; lifeStage: adult; otherCatalogNumbers: accession#SFASU lot 53; **Taxon:** scientificName: Mixogasterfattigi Locke, Skevington and Greene; kingdom: Animalia; phylum: Arthropoda; class: Hexapoda; order: Diptera; family: Syrphidae; genus: Mixogaster; specificEpithet: fattigi; taxonRank: species; scientificNameAuthorship: Locke, Skevington and Greene 2019; vernacularName: Fattig's Ant Fly; nomenclaturalStatus: new species; **Location:** country: U.S.A.; stateProvince: Texas; locality: Lamar Co., Camp Maxey; decimalLatitude: 33.667222; decimalLongitude: -95.566389; **Identification:** identifiedBy: J.H. Skevington; **Event:** samplingProtocol: Malaise trap; eventDate: 2003-07-01; year: 2003; month: 7; day: 1; verbatimEventDate: 1-15.vii.2003; habitat: in pine forest; **Record Level:** language: en; institutionCode: CNC; basisOfRecord: PreservedSpecimen
- **Type status:** Paratype. **Occurrence:** catalogNumber: Jeff_Skevington_Specimen45174; recordedBy: N. Bedwell; individualCount: 1; sex: male; lifeStage: adult; **Taxon:** scientificName: Mixogasterfattigi Locke, Skevington and Greene; kingdom: Animalia; phylum: Arthropoda; class: Hexapoda; order: Diptera; family: Syrphidae; genus: Mixogaster; specificEpithet: fattigi; taxonRank: species; scientificNameAuthorship: Locke, Skevington and Greene 2019; vernacularName: Fattig's Ant Fly; nomenclaturalStatus: new species; **Location:** country: U.S.A.; stateProvince: Mississippi; locality: Winston Co., 13 mi. S. Starkville; decimalLatitude: 33.286; decimalLongitude: -88.91; **Identification:** identifiedBy: J.H. Skevington; **Event:** samplingProtocol: Malaise trap; eventDate: 1982-06-28; year: 1982; month: 6; day: 28; verbatimEventDate: 28.vi.1982; habitat: pine forest; **Record Level:** language: en; institutionCode: ANSP; basisOfRecord: PreservedSpecimen
- **Type status:** Paratype. **Occurrence:** catalogNumber: Jeff_Skevington_Specimen45175; recordedBy: H. V. Weems, Jr. & G. B. Fairchild; individualCount: 1; sex: male; lifeStage: adult; **Taxon:** scientificName: Mixogasterfattigi Locke, Skevington and Greene; kingdom: Animalia; phylum: Arthropoda; class: Hexapoda; order: Diptera; family: Syrphidae; genus: Mixogaster; specificEpithet: fattigi; taxonRank: species; scientificNameAuthorship: Locke, Skevington and Greene 2019; vernacularName: Fattig's Ant Fly; nomenclaturalStatus: new species; **Location:** country: U.S.A.; stateProvince: Florida; locality: Alachua Co., San Felasco Hammock; decimalLatitude: 29.73; decimalLongitude: -82.43; **Identification:** identifiedBy: J.H. Skevington; **Event:** samplingProtocol: carbon dioxide baited flight interception trap; eventDate: 1977-06-02; year: 1977; month: 6; day: 2; verbatimEventDate: 2.vi.1977; **Record Level:** language: en; institutionCode: ANSP; basisOfRecord: PreservedSpecimen
- **Type status:** Paratype. **Occurrence:** catalogNumber: MEM92633; recordedBy: R.L. Brown; individualCount: 1; sex: male; lifeStage: adult; **Taxon:** scientificName: Mixogasterfattigi Locke, Skevington and Greene; kingdom: Animalia; phylum: Arthropoda; class: Hexapoda; order: Diptera; family: Syrphidae; genus: Mixogaster; specificEpithet: fattigi; taxonRank: species; scientificNameAuthorship: Locke, Skevington and Greene 2019; vernacularName: Fattig's Ant Fly; nomenclaturalStatus: new species; **Location:** country: U.S.A.; stateProvince: Mississippi; locality: Oktibbeha Co., Dorman Lk.; decimalLatitude: 33.3394; decimalLongitude: -88.8728; **Identification:** identifiedBy: J.H. Skevington; **Event:** samplingProtocol: Malaise trap; eventDate: 1981-06-20/27; year: 1981; month: 6; day: 20; verbatimEventDate: 20-27.vi.1981; habitat: hardwood forest; **Record Level:** language: en; institutionCode: MEMU; basisOfRecord: PreservedSpecimen
- **Type status:** Paratype. **Occurrence:** catalogNumber: MEM92634; recordedBy: R.L. & B.B. Brown; individualCount: 1; sex: male; lifeStage: adult; **Taxon:** scientificName: Mixogasterfattigi Locke, Skevington and Greene; kingdom: Animalia; phylum: Arthropoda; class: Hexapoda; order: Diptera; family: Syrphidae; genus: Mixogaster; specificEpithet: fattigi; taxonRank: species; scientificNameAuthorship: Locke, Skevington and Greene 2019; vernacularName: Fattig's Ant Fly; nomenclaturalStatus: new species; **Location:** country: U.S.A.; stateProvince: Mississippi; locality: Oktibbeha Co., 6 mi. SW of Starkville; decimalLatitude: 33.3646; decimalLongitude: -88.873; **Identification:** identifiedBy: J.H. Skevington; **Event:** samplingProtocol: Malaise trap; eventDate: 1984-07-18/22; year: 1984; month: 7; day: 18; verbatimEventDate: 18-22.vii.1984; habitat: mixed pine/hardwood forest; **Record Level:** language: en; institutionCode: MEMU; basisOfRecord: PreservedSpecimen
- **Type status:** Paratype. **Occurrence:** catalogNumber: MEM92716; recordedBy: R.L. Brown; individualCount: 1; sex: male; lifeStage: adult; **Taxon:** scientificName: Mixogasterfattigi Locke, Skevington and Greene; kingdom: Animalia; phylum: Arthropoda; class: Hexapoda; order: Diptera; family: Syrphidae; genus: Mixogaster; specificEpithet: fattigi; taxonRank: species; scientificNameAuthorship: Locke, Skevington and Greene 2019; vernacularName: Fattig's Ant Fly; nomenclaturalStatus: new species; **Location:** country: U.S.A.; stateProvince: Mississippi; locality: Oktibbeha Co., 5 mi. S of Starkville; decimalLatitude: 33.379722; decimalLongitude: -88.828889; **Identification:** identifiedBy: J.H. Skevington; **Event:** samplingProtocol: Malaise trap; eventDate: 2000-06-20/26; year: 2000; month: 6; day: 20; verbatimEventDate: 20-26.vi.2000; habitat: mixed pine/oak forest; **Record Level:** language: en; institutionCode: MEMU; basisOfRecord: PreservedSpecimen
- **Type status:** Paratype. **Occurrence:** catalogNumber: MEM92717; recordedBy: R.L. Brown; individualCount: 2; sex: male; lifeStage: adult; **Taxon:** scientificName: Mixogasterfattigi Locke, Skevington and Greene; kingdom: Animalia; phylum: Arthropoda; class: Hexapoda; order: Diptera; family: Syrphidae; genus: Mixogaster; specificEpithet: fattigi; taxonRank: species; scientificNameAuthorship: Locke, Skevington and Greene 2019; vernacularName: Fattig's Ant Fly; nomenclaturalStatus: new species; **Location:** country: U.S.A.; stateProvince: Mississippi; locality: Oktibbeha Co., 5 mi. S of Starkville; decimalLatitude: 33.379722; decimalLongitude: -88.828889; **Identification:** identifiedBy: J.H. Skevington; **Event:** samplingProtocol: Malaise trap; eventDate: 2000-06-13/19; year: 2000; month: 6; day: 13; verbatimEventDate: 13-19.vi.2000; habitat: mixed pine/oak forest; **Record Level:** language: en; institutionCode: MEMU; basisOfRecord: PreservedSpecimen
- **Type status:** Paratype. **Occurrence:** catalogNumber: MEM92718; recordedBy: R.L. & B.B. Brown; individualCount: 1; sex: male; lifeStage: adult; **Taxon:** scientificName: Mixogasterfattigi Locke, Skevington and Greene; kingdom: Animalia; phylum: Arthropoda; class: Hexapoda; order: Diptera; family: Syrphidae; genus: Mixogaster; specificEpithet: fattigi; taxonRank: species; scientificNameAuthorship: Locke, Skevington and Greene 2019; vernacularName: Fattig's Ant Fly; nomenclaturalStatus: new species; **Location:** country: U.S.A.; stateProvince: Mississippi; locality: Oktibbeha Co., T18N R14E Sec. 23; decimalLatitude: 33.43; decimalLongitude: -88.88; **Identification:** identifiedBy: J.H. Skevington; **Event:** eventDate: 1990-06-26; year: 1990; month: 6; day: 26; verbatimEventDate: 26.vi.1990; habitat: mixed pine/oak forest; **Record Level:** language: en; institutionCode: MEMU; basisOfRecord: PreservedSpecimen
- **Type status:** Paratype. **Occurrence:** catalogNumber: MEM92719; recordedBy: R.L. & B.B. Brown; individualCount: 1; sex: male; lifeStage: adult; **Taxon:** scientificName: Mixogasterfattigi Locke, Skevington and Greene; kingdom: Animalia; phylum: Arthropoda; class: Hexapoda; order: Diptera; family: Syrphidae; genus: Mixogaster; specificEpithet: fattigi; taxonRank: species; scientificNameAuthorship: Locke, Skevington and Greene; vernacularName: Fattig's Ant Fly; nomenclaturalStatus: new species; **Location:** country: U.S.A.; stateProvince: Mississippi; locality: Oktibbeha Co., 6 mi SW Starkville; decimalLatitude: 33.3646; decimalLongitude: -88.873; **Identification:** identifiedBy: J.H. Skevington; **Event:** samplingProtocol: Malaise trap; eventDate: 1990-07-11; year: 1990; month: 7; day: 11; verbatimEventDate: 11.vii.1990; **Record Level:** language: en; institutionCode: MEMU; basisOfRecord: PreservedSpecimen
- **Type status:** Paratype. **Occurrence:** catalogNumber: MEM92720; recordedBy: R.L. & B.B. Brown; individualCount: 2; sex: male; lifeStage: adult; **Taxon:** scientificName: Mixogasterfattigi Locke, Skevington and Greene; kingdom: Animalia; phylum: Arthropoda; class: Hexapoda; order: Diptera; family: Syrphidae; genus: Mixogaster; specificEpithet: fattigi; taxonRank: species; scientificNameAuthorship: Locke, Skevington and Greene 2019; vernacularName: Fattig's Ant Fly; nomenclaturalStatus: new species; **Location:** country: U.S.A.; stateProvince: Mississippi; locality: Oktibbeha Co., 6 mi SW Starkville; decimalLatitude: 33.3646; decimalLongitude: -88.873; **Identification:** identifiedBy: J.H. Skevington; **Event:** samplingProtocol: Malaise trap; eventDate: 1984-07-06; year: 1984; month: 7; day: 6; verbatimEventDate: 6-7.vii.1984; **Record Level:** language: en; institutionCode: MEMU; basisOfRecord: PreservedSpecimen
- **Type status:** Paratype. **Occurrence:** catalogNumber: MEM92721; recordedBy: R.L. & B.B. Brown; individualCount: 3; sex: male; lifeStage: adult; **Taxon:** scientificName: Mixogasterfattigi Locke, Skevington and Greene; kingdom: Animalia; phylum: Arthropoda; class: Hexapoda; order: Diptera; family: Syrphidae; genus: Mixogaster; specificEpithet: fattigi; taxonRank: species; scientificNameAuthorship: Locke, Skevington and Greene 2019; vernacularName: Fattig's Ant Fly; nomenclaturalStatus: new species; **Location:** country: U.S.A.; stateProvince: Mississippi; locality: Oktibbeha Co., 6 mi SW Starkville; decimalLatitude: 33.3646; decimalLongitude: -88.873; **Identification:** identifiedBy: J.H. Skevington; **Event:** samplingProtocol: Malaise trap; eventDate: 1984-07-18/22; year: 1984; month: 7; day: 18; verbatimEventDate: 18-22.vii.1984; habitat: mixed pine/hardwood forest; **Record Level:** language: en; institutionCode: MEMU; basisOfRecord: PreservedSpecimen
- **Type status:** Paratype. **Occurrence:** catalogNumber: USNMENT19965; recordedBy: W.W. Wirth; individualCount: 1; sex: female; lifeStage: adult; **Taxon:** scientificName: Mixogasterfattigi Locke, Skevington and Greene; kingdom: Animalia; phylum: Arthropoda; class: Hexapoda; order: Diptera; family: Syrphidae; genus: Mixogaster; specificEpithet: fattigi; taxonRank: species; scientificNameAuthorship: Locke, Skevington and Greene 2019; vernacularName: Fattig's Ant Fly; nomenclaturalStatus: new species; **Location:** country: U.S.A.; stateProvince: Virginia; locality: Falls Church; decimalLatitude: 38.883333; decimalLongitude: -77.166667; **Identification:** identifiedBy: J.H. Skevington; **Event:** eventDate: 1951-07-22; year: 1951; month: 7; day: 22; verbatimEventDate: 22.vii.1951; habitat: Stream margin; **Record Level:** language: en; institutionCode: USNM; basisOfRecord: PreservedSpecimen
- **Type status:** Paratype. **Occurrence:** catalogNumber: USNMENT247135; recordedBy: P.W. Fattig; individualCount: 1; sex: male; lifeStage: adult; **Taxon:** scientificName: Mixogasterfattigi Locke, Skevington and Greene; kingdom: Animalia; phylum: Arthropoda; class: Hexapoda; order: Diptera; family: Syrphidae; genus: Mixogaster; specificEpithet: fattigi; taxonRank: species; scientificNameAuthorship: Locke, Skevington and Greene 2019; vernacularName: Fattig's Ant Fly; nomenclaturalStatus: new species; **Location:** country: U.S.A.; stateProvince: Georgia; locality: Atlanta; decimalLatitude: 33.75; decimalLongitude: -84.383333; **Identification:** identifiedBy: J.H. Skevington; **Event:** eventDate: 1942-06-09; year: 1942; month: 7; day: 9; verbatimEventDate: 9.vii.1942; **Record Level:** language: en; institutionCode: USNM; basisOfRecord: PreservedSpecimen
- **Type status:** Paratype. **Occurrence:** catalogNumber: USNMENT247136; recordedBy: H.D. Pratt; individualCount: 1; sex: male; lifeStage: adult; **Taxon:** scientificName: Mixogasterfattigi Locke, Skevington and Greene; kingdom: Animalia; phylum: Arthropoda; class: Hexapoda; order: Diptera; family: Syrphidae; genus: Mixogaster; specificEpithet: fattigi; taxonRank: species; scientificNameAuthorship: Locke, Skevington and Greene 2019; vernacularName: Fattig's Ant Fly; nomenclaturalStatus: new species; **Location:** country: U.S.A.; stateProvince: Georgia; locality: Atlanta; decimalLatitude: 33.75; decimalLongitude: -84.383333; **Identification:** identifiedBy: J.H. Skevington; **Event:** eventDate: 1973-07-07; year: 1973; month: 7; day: 7; verbatimEventDate: 7.vii.1973; **Record Level:** language: en; institutionCode: USNM; basisOfRecord: PreservedSpecimen
- **Type status:** Paratype. **Occurrence:** catalogNumber: USNMENT247137; recordedBy: R.W. Matthews; individualCount: 1; sex: male; lifeStage: adult; **Taxon:** scientificName: Mixogasterfattigi Locke, Skevington and Greene; kingdom: Animalia; phylum: Arthropoda; class: Hexapoda; order: Diptera; family: Syrphidae; genus: Mixogaster; specificEpithet: fattigi; taxonRank: species; scientificNameAuthorship: Locke, Skevington and Greene 2019; vernacularName: Fattig's Ant Fly; nomenclaturalStatus: new species; **Location:** country: U.S.A.; stateProvince: Georgia; locality: Clarke County, Athens; decimalLatitude: 33.95; decimalLongitude: -83.366667; **Identification:** identifiedBy: J.H. Skevington; **Event:** samplingProtocol: Malaise trap; eventDate: 1976-10/12; year: 1976; month: 10; verbatimEventDate: x.-xii.1976; **Record Level:** language: en; institutionCode: USNM; basisOfRecord: PreservedSpecimen
- **Type status:** Paratype. **Occurrence:** catalogNumber: USNMENT247138; recordedBy: R.W. Matthews; individualCount: 1; sex: male; lifeStage: adult; **Taxon:** scientificName: Mixogasterfattigi Locke, Skevington and Greene; kingdom: Animalia; phylum: Arthropoda; class: Hexapoda; order: Diptera; family: Syrphidae; genus: Mixogaster; specificEpithet: fattigi; taxonRank: species; scientificNameAuthorship: Locke, Skevington and Greene 2019; vernacularName: Fattig's Ant Fly; nomenclaturalStatus: new species; **Location:** country: U.S.A.; stateProvince: Georgia; locality: Clarke County, Athens; decimalLatitude: 33.95; decimalLongitude: -83.366667; **Identification:** identifiedBy: J.H. Skevington; **Event:** samplingProtocol: Malaise trap; eventDate: 1976-10/12; year: 1976; month: 10; verbatimEventDate: x.-xii.1976; **Record Level:** language: en; institutionCode: USNM; basisOfRecord: PreservedSpecimen
- **Type status:** Paratype. **Occurrence:** catalogNumber: USNMENT247139; recordedBy: R. Duffield; individualCount: 1; sex: male; lifeStage: adult; **Taxon:** scientificName: Mixogasterfattigi Locke, Skevington and Greene; kingdom: Animalia; phylum: Arthropoda; class: Hexapoda; order: Diptera; family: Syrphidae; genus: Mixogaster; specificEpithet: fattigi; taxonRank: species; scientificNameAuthorship: Locke, Skevington and Greene 2019; vernacularName: Fattig's Ant Fly; nomenclaturalStatus: new species; **Location:** country: U.S.A.; stateProvince: Georgia; locality: Athens; decimalLatitude: 33.95; decimalLongitude: -83.366667; **Identification:** identifiedBy: J.H. Skevington; **Event:** samplingProtocol: Malaise trap; **Record Level:** language: en; institutionCode: USNM; basisOfRecord: PreservedSpecimen
- **Type status:** Paratype. **Occurrence:** catalogNumber: USNMENT247140; recordedBy: R.T. Franklin; individualCount: 1; sex: male; lifeStage: adult; **Taxon:** scientificName: Mixogasterfattigi Locke, Skevington and Greene; kingdom: Animalia; phylum: Arthropoda; class: Hexapoda; order: Diptera; family: Syrphidae; genus: Mixogaster; specificEpithet: fattigi; taxonRank: species; scientificNameAuthorship: Locke, Skevington and Greene 2019; vernacularName: Fattig's Ant Fly; nomenclaturalStatus: new species; **Location:** country: U.S.A.; stateProvince: Georgia; locality: Clarke County; decimalLatitude: 33.95; decimalLongitude: -83.366667; **Identification:** identifiedBy: J.H. Skevington; **Event:** samplingProtocol: Malaise trap; eventDate: 1971-07-04/10; year: 1971; month: 7; day: 4; verbatimEventDate: 4-10.vii.1971; **Record Level:** language: en; institutionCode: USNM; basisOfRecord: PreservedSpecimen
- **Type status:** Paratype. **Occurrence:** catalogNumber: USNMENT247141; recordedBy: R. Franklin; individualCount: 1; sex: male; lifeStage: adult; **Taxon:** scientificName: Mixogasterfattigi Locke, Skevington and Greene; kingdom: Animalia; phylum: Arthropoda; class: Hexapoda; order: Diptera; family: Syrphidae; genus: Mixogaster; specificEpithet: fattigi; taxonRank: species; scientificNameAuthorship: Locke, Skevington and Greene 2019; vernacularName: Fattig's Ant Fly; nomenclaturalStatus: new species; **Location:** country: U.S.A.; stateProvince: Georgia; locality: Athens; decimalLatitude: 33.95; decimalLongitude: -83.366667; **Identification:** identifiedBy: J.H. Skevington; **Event:** samplingProtocol: Malaise trap; eventDate: 1971-06-30; year: 1971; month: 6; day: 30; verbatimEventDate: 30.vi.1971; **Record Level:** language: en; institutionCode: USNM; basisOfRecord: PreservedSpecimen
- **Type status:** Paratype. **Occurrence:** catalogNumber: USNMENT247142; individualCount: 1; sex: male; lifeStage: adult; **Taxon:** scientificName: Mixogasterfattigi Locke, Skevington and Greene; kingdom: Animalia; phylum: Arthropoda; class: Hexapoda; order: Diptera; family: Syrphidae; genus: Mixogaster; specificEpithet: fattigi; taxonRank: species; scientificNameAuthorship: Locke, Skevington and Greene 2019; vernacularName: Fattig's Ant Fly; nomenclaturalStatus: new species; **Location:** country: U.S.A.; stateProvince: Georgia; locality: Clarke County, Athens; decimalLatitude: 33.95; decimalLongitude: -83.366667; **Identification:** identifiedBy: J.H. Skevington; **Event:** eventDate: 1976-07; year: 1976; month: 7; verbatimEventDate: vii.1976; **Record Level:** language: en; institutionCode: USNM; basisOfRecord: PreservedSpecimen
- **Type status:** Paratype. **Occurrence:** catalogNumber: USNMENT247143; recordedBy: R. Duffield; individualCount: 1; sex: female; lifeStage: adult; **Taxon:** scientificName: Mixogasterfattigi Locke, Skevington and Greene; kingdom: Animalia; phylum: Arthropoda; class: Hexapoda; order: Diptera; family: Syrphidae; genus: Mixogaster; specificEpithet: fattigi; taxonRank: species; scientificNameAuthorship: Locke, Skevington and Greene 2019; vernacularName: Fattig's Ant Fly; nomenclaturalStatus: new species; **Location:** country: U.S.A.; stateProvince: Georgia; locality: Athens; decimalLatitude: 33.95; decimalLongitude: -83.366667; **Identification:** identifiedBy: J.H. Skevington; **Event:** samplingProtocol: Malaise trap; **Record Level:** language: en; institutionCode: USNM; basisOfRecord: PreservedSpecimen
- **Type status:** Paratype. **Occurrence:** catalogNumber: USNMENT247144; recordedBy: A. Lavallee; individualCount: 1; sex: male; lifeStage: adult; **Taxon:** scientificName: Mixogasterfattigi Locke, Skevington and Greene; kingdom: Animalia; phylum: Arthropoda; class: Hexapoda; order: Diptera; family: Syrphidae; genus: Mixogaster; specificEpithet: fattigi; taxonRank: species; scientificNameAuthorship: Locke, Skevington and Greene 2019; vernacularName: Fattig's Ant Fly; nomenclaturalStatus: new species; **Location:** country: U.S.A.; stateProvince: Georgia; locality: Bogart; decimalLatitude: 33.949258; decimalLongitude: -83.5346; **Identification:** identifiedBy: J.H. Skevington; **Event:** eventDate: 1974-07-07; year: 1974; month: 7; day: 7; verbatimEventDate: 7.vii.1974; **Record Level:** language: en; institutionCode: USNM; basisOfRecord: PreservedSpecimen
- **Type status:** Paratype. **Occurrence:** catalogNumber: USNMENT247145; recordedBy: R.W. Matthews; individualCount: 1; sex: male; lifeStage: adult; **Taxon:** scientificName: Mixogasterfattigi Locke, Skevington and Greene; kingdom: Animalia; phylum: Arthropoda; class: Hexapoda; order: Diptera; family: Syrphidae; genus: Mixogaster; specificEpithet: fattigi; taxonRank: species; scientificNameAuthorship: Locke, Skevington and Greene 2019; vernacularName: Fattig's Ant Fly; nomenclaturalStatus: new species; **Location:** country: U.S.A.; stateProvince: Georgia; locality: Clarke County, Athens; decimalLatitude: 33.95; decimalLongitude: -83.366667; **Identification:** identifiedBy: J.H. Skevington; **Event:** samplingProtocol: Malaise trap; eventDate: 1976-10/12; year: 1976; month: 10; verbatimEventDate: x.-xii.1976; **Record Level:** language: en; institutionCode: USNM; basisOfRecord: PreservedSpecimen
- **Type status:** Paratype. **Occurrence:** catalogNumber: USNMENT247146; recordedBy: A. Lavallee; individualCount: 1; sex: male; lifeStage: adult; **Taxon:** scientificName: Mixogasterfattigi Locke, Skevington and Greene; kingdom: Animalia; phylum: Arthropoda; class: Hexapoda; order: Diptera; family: Syrphidae; genus: Mixogaster; specificEpithet: fattigi; taxonRank: species; scientificNameAuthorship: Locke, Skevington and Greene 2019; vernacularName: Fattig's Ant Fly; nomenclaturalStatus: new species; **Location:** country: U.S.A.; stateProvince: Georgia; locality: Bogart; decimalLatitude: 33.949258; decimalLongitude: -83.5346; **Identification:** identifiedBy: J.H. Skevington; **Event:** eventDate: 1972-07-07; year: 1972; month: 7; day: 7; verbatimEventDate: 7.vii.1972; **Record Level:** language: en; institutionCode: USNM; basisOfRecord: PreservedSpecimen
- **Type status:** Paratype. **Occurrence:** catalogNumber: USNMENT247147; recordedBy: R. Duffield; individualCount: 1; sex: male; lifeStage: adult; **Taxon:** scientificName: Mixogasterfattigi Locke, Skevington and Greene; kingdom: Animalia; phylum: Arthropoda; class: Hexapoda; order: Diptera; family: Syrphidae; genus: Mixogaster; specificEpithet: fattigi; taxonRank: species; scientificNameAuthorship: Locke, Skevington and Greene 2019; vernacularName: Fattig's Ant Fly; nomenclaturalStatus: new species; **Location:** country: U.S.A.; stateProvince: Georgia; locality: Athens; decimalLatitude: 33.95; decimalLongitude: -83.366667; **Identification:** identifiedBy: J.H. Skevington; **Event:** samplingProtocol: Malaise trap; **Record Level:** language: en; institutionCode: USNM; basisOfRecord: PreservedSpecimen
- **Type status:** Paratype. **Occurrence:** catalogNumber: USNMENT247148; recordedBy: R. Duffield; individualCount: 1; sex: male; lifeStage: adult; **Taxon:** scientificName: Mixogasterfattigi Locke, Skevington and Greene; kingdom: Animalia; phylum: Arthropoda; class: Hexapoda; order: Diptera; family: Syrphidae; genus: Mixogaster; specificEpithet: fattigi; taxonRank: species; scientificNameAuthorship: Locke, Skevington and Greene 2019; vernacularName: Fattig's Ant Fly; nomenclaturalStatus: new species; **Location:** country: U.S.A.; stateProvince: Georgia; locality: Athens; decimalLatitude: 33.95; decimalLongitude: -83.366667; **Identification:** identifiedBy: J.H. Skevington; **Event:** samplingProtocol: Malaise trap; **Record Level:** language: en; institutionCode: CNC; basisOfRecord: PreservedSpecimen

#### Description

**Size**: Body length: 10.3-14.6 mm; wing length 7.4-9.1 mm

**Male: Head**: Face slightly convex, parallel-sided, projecting downwards slightly below the eye, yellow, shining with yellow pile, sometimes with darkened area below antennae rarely extending to black, narrow medial stripe not reaching the oral margin; gena yellow, reduced, yellow pilose, separated from face by black stripe extending from eye to oral margin; frons with broad black transverse band along lower edge, black pilose laterally, some admixed yellow pile, shining medially; vertex yellow, black pilose; ocellar triangle black with black extending posteriorly to a point; antenna elongate, dark, scape and pedicel yellow to orange, scape elongate, postpedicel brown to black, >2 times as long as scape, arista yellow; eye bare.

**Thorax**: Scutum mostly black, mostly yellow pilose, yellow laterally from postpronotum to postalar callus, sometimes narrowly brown anterior to postalar callus; postpronotum yellow; postalar callus yellow; scutellum yellow, yellow pilose; pleuron yellow, shining, bare, brown to black on anterior margin of anepisternum, ventral ½ katepisternum, posterior anepimeron and meron; legs mostly yellow, coxae brown, femora sometimes slightly darker, orange to brown, on basal ⅔, tarsi often slightly darker, orange to brown. Wing infuscated anteriorly in cells bc, c, sc, r_1_, r_2+3_ and br anterior to spurious vein, wing densely microtrichose apically, bare basally, cells br and bm extensively bare, microtrichose apically, cua bare on basal half, cup bare basally, alula bare; calypter brown; halter yellow-orange.

**Abdomen**: Abdomen constricted anteriorly; tergites 1+2 fused, about as wide as scutellum at base, constricted medially, longer than tergite 4, yellow, small brown spot on medioanterior edge, large brown marking on medial ½-⅔; tergites 3 and 4 brown, posterior edge with broad yellow band, pile on tergites mostly yellow on yellow cuticle and black on black cuticle except some yellow pile on black cuticle posteriorly on tergite 4; sternite 1 shining, bare, brown anteriorly, yellow posteriorly; sternite 2 shining, bare, yellow, often brown medially, usually restricted to lateral edges; sternite 3 yellow with broad brown to black medial band, mostly black pilose, sometimes yellow pile medially; sternite 4 broadly brown to black with yellow posterior edge, anterior edge sometimes narrowly yellow, mostly yellow pile on black cuticle and black pile on yellow cuticle.

**Genitalia**: Epandrium longer than tall, mostly covered in short pile; cercus connected to epandrium posteroventrally, with dorsal lobe, outer ventral corner projecting slightly, covered in long setae; surstylus pointed, curving inwards, covered in long setae; basal hypandrium rounded ventrally, apical hypandrium bifurcate, on either side of phallus, subquadrate with apicodorsal corner projecting further than apicoventral corner, covered in short pile; phallus laterally compressed, slightly smaller than basal hypandrium and not projecting much beyond, phallus ends in small point directed dorsally.

**Female**: Differs from male in the following ways: face with yellow pile sometimes admixed with few black pili, medial facial stripe extending ventrally to oral margin; frons entirely black pilose; scutellum black or yellow pilose; tergites 1+2 constricted medially but less so than in male, not as long as in male, yellow anteriorly with brown anteromedial spot, brown medially, yellow along posterior margin; tergite 5 similar to tergite 4 of male; sternite 1 almost entirely brown, narrowly yellow posteriorly; sternite 2 yellow with brown medial band and lateral margins brown medially, sparse pile posteriorly; sternites 3-5 brown with yellow posterior margin, black pilose with some yellow pili admixed.

See Fig. 10.

#### Diagnosis

Yellow vertex and black ocellar triangle; face yellow, occasionally with darkened area below antennae extending to black, narrow medial stripe not reaching the oral margin; tergites 1+2 fused, longer than tergite 4.

#### Etymology

Named after the collector of the first known specimens, P.W. Fattig. Fattig was the Curator of the Museum in Emory University from 1926 to 1953 and noted by Weems (1953) to have encouraged his first interest in entomology.

#### Distribution

This species is known from the south-eastern United States including Florida, Georgia, Louisiana, Mississippi, Tennessee, Texas and Virginia.

#### Ecology

This fly is rarely collected. It has been found from early June to late August, with three specimens having been collected in a Malaise trap sometime from October to December in Georgia. This species is known from pine, pine-oak, pine-hardwood or hardwood forests.

#### Taxon discussion

*Mixogasterfattigi* differs from *M.breviventris* Kahl 1897 and *M.johnsoni* Hull 1941 by the entirely yellow vertex, entirely yellow metafemur (not dark on apicomedial ⅓) and yellow lateral margin of 2nd tergite (not dark on posterior 2/3); from *M.cubensis* Curran 1932 by the uniformly dark postpedicel which is not strongly constricted medially.

This species has sometimes been attributed to Howard Weems as he provided a description of it in his PhD thesis (Weems 1953). However, as Weems himself did not consider his thesis a publication in the meaning of the Code (International Commission on Zoological Nomenclature 1999), the name has remained a nomen nudum. In the files of the Systematic Entomology Laboratory at the Smithsonian, Chris Thompson found a complete manuscript by his predecessor, Charles T. Greene, written long before the Weems' thesis. Despite having not seeing Greene's work, we have honoured his contribution by including him as an author of the species.

A single specimen was sequenced (Table 2).

#### Common Name

The common name given to the species by Skevington et al. (2019) is Fattig's Ant Fly.

### Neoascia guttata

Skevington and Moran sp. n.

840E6300D88C5E7D94D41A1369D353A7

urn:lsid:zoobank.org:act:A1BD8821-B02E-492C-B2C7-E24209387F7D

#### Materials

- **Type status:** Holotype. **Occurrence:** catalogNumber: CNC_Diptera170046; recordedBy: D.M. Wood; individualCount: 1; sex: male; lifeStage: adult; **Taxon:** scientificName: Neoasciaguttata Skevington and Moran; kingdom: Animalia; phylum: Arthropoda; class: Hexapoda; order: Diptera; family: Syrphidae; genus: Neoascia; subgenus: Neoasciella; specificEpithet: guttata; taxonRank: species; scientificNameAuthorship: Skevington and Moran 2019; vernacularName: Spotted Fen Fly; nomenclaturalStatus: new species; **Location:** country: Canada; stateProvince: Nova Scotia; locality: Lawrencetown, Halifax County; decimalLatitude: 44.644175; decimalLongitude: -63.344633; **Identification:** identifiedBy: J.H. Skevington; **Event:** samplingProtocol: hand collected; eventDate: 1967-07-19/20; year: 1967; month: 7; day: 19; verbatimEventDate: 19-20.vii.1967; **Record Level:** language: en; institutionCode: CNC; basisOfRecord: PreservedSpecimen
- **Type status:** Paratype. **Occurrence:** catalogNumber: CNC_Diptera169737; recordedBy: W.R.M. Mason; individualCount: 1; sex: female; lifeStage: adult; **Taxon:** scientificName: Neoasciaguttata Skevington and Moran; kingdom: Animalia; phylum: Arthropoda; class: Hexapoda; order: Diptera; family: Syrphidae; genus: Neoascia; subgenus: Neoasciella; specificEpithet: guttata; taxonRank: species; scientificNameAuthorship: Skevington and Moran 2019; vernacularName: Spotted Fen Fly; nomenclaturalStatus: new species; **Location:** country: Canada; stateProvince: Alberta; locality: Jumping Pound Creek, 20 miles West of Calgary; decimalLatitude: 51.040727; decimalLongitude: -114.753223; **Identification:** identifiedBy: K. Moran; **Event:** samplingProtocol: hand collected; eventDate: 1962-06-23; year: 1962; month: 6; day: 23; verbatimEventDate: 23.vi.1962; **Record Level:** language: en; institutionCode: CNC; basisOfRecord: PreservedSpecimen
- **Type status:** Paratype. **Occurrence:** catalogNumber: CNC_Diptera169738; recordedBy: C.B.D. Garrett; individualCount: 1; sex: female; lifeStage: adult; **Taxon:** scientificName: Neoasciaguttata Skevington and Moran; kingdom: Animalia; phylum: Arthropoda; class: Hexapoda; order: Diptera; family: Syrphidae; genus: Neoascia; subgenus: Neoasciella; specificEpithet: guttata; taxonRank: species; scientificNameAuthorship: Skevington and Moran 2019; vernacularName: Spotted Fen Fly; nomenclaturalStatus: new species; **Location:** country: Canada; stateProvince: Alberta; locality: Banff; decimalLatitude: 51.18; decimalLongitude: -115.57; **Identification:** identifiedBy: K. Moran; **Event:** samplingProtocol: hand collected; eventDate: 1922-06-2; year: 1922; month: 6; day: 2; verbatimEventDate: 2.vi.1922; **Record Level:** language: en; institutionCode: CNC; basisOfRecord: PreservedSpecimen

#### Description

**Size**: Body length 4.7 to 5.0 mm; wing length 3.8 to 4.4 mm

**Male: Head**: Black with metallic sheen; face yellowish pollinose and white pilose; gena sparsely pollinose and pilose posteriorly; frons shiny, rugose, white pilose on ventral ⅔, black pilose dorsally; vertex shiny, smooth, pale pilose; occiput sparsely grey pollinose and black pilose dorsally, becoming densely silvery white pollinose and white pilose ventrally; antenna dark brown except orange basoventral ⅓ of postpedicel; postpedicel short, about as long as wide; arista slightly longer than postpedicel.

**Thorax**: Black with metallic sheen; postpronotum shiny, yellowish-white pilose; mesonotum shiny, yellowish-white pilose; scutellum shiny, yellowish-white pilose; pleuron greyish-white pollinose except shiny on most of katepisternum, meron and katatergite; katepisternum with a few white pili dorsally; meron bare; katatergite white pilose; postmetacoxal bridge absent; coxae dark brown, greyish-white pollinose, white and black pilose; trochanters orange; pro- and mesofemora narrowly orange basally and apically, black elsewhere, white pilose; pro- and mesotibiae and tarsi similar with a bit more orange basally; metafemur orange on basal ⅕ and narrowly on apex, black elsewhere, black pilose; metatibia orange on basal ⅓ and on apical tip, black medially, white pilose; tarsi bicoloured, pale pilose; basitarsomere brown except orange apically; 2nd and 3rd tarsomeres orange; apical 2 tarsomeres brown; calypter white; halter white; wing lightly infuscate, completely microtrichose.

**Abdomen**: Black except with paired orange spots proximally on 3rd tergite, with metallic sheen, white pilose.

**Genitalia**: Male terminalia black pilose; epandrium compact, about same length as surstylus; cercus elongate, protruding; surstylus broad and elongate, with a few large bristles on the dorsal proximal corner; distiphallus simple, bag-like; hypandrium elongate; postgonite simple; phallapodeme straight; ejaculatory apodeme tiny, parasol-shaped.

**Female**: Similar to male except for normal sexual dimorphism and the following: abdomen completely black (spots absent); metatarsi entirely black.

See Fig. 11.

#### Diagnosis

Postmetacoxal bridge absent. Face long and straight, different from shorter, concave face found in similar species. Paired spots on tergite 4 of male. Male cerci protruding; surstyli broad and elongate.

#### Etymology

This name is from the Latin *guttata*, meaning dappled, speckled, spotted.

#### Distribution

This species is known from a single male collected on 19-20 July 1967 in Lawrencetown, Nova Scotia by Monty Wood and two females from Alberta.

#### Ecology

This rare fly has been collected from early June to mid-July.

#### Taxon discussion

The unique face shape (long and straight) suggests that these females are correctly associated with the males. DNA evidence (Table 2) corroborates this. As the abdominal pattern varies in *Neoascia*, it is possible that further dissections will turn up more specimens of this species that are masquerading as other species. DNA barcodes suggest that this species is closely related to the North African species *N.clausseni* Hauser and Kassebeer 1998. The shape of the face is identical in these two species, but the abdominal pattern and male genitalia are different.

#### Common Name

The common name given to the species by Skevington et al. (2019) is Spotted Fen Fly .

### Orthonevra feei

Moran and Skevington sp. n.

A38680BE9C115F5695596DF90B08050A

urn:lsid:zoobank.org:act:66A24081-4FF8-421E-AD79-89E139B6428F

#### Materials

- **Type status:** Holotype. **Occurrence:** catalogNumber: Jeff_Skevington_Specimen44217; recordedBy: F.D. Fee; individualCount: 1; sex: male; lifeStage: adult; **Taxon:** scientificName: Orthonevrafeei Moran and Skevington; kingdom: Animalia; phylum: Arthropoda; class: Hexapoda; order: Diptera; family: Syrphidae; genus: Orthonevra; specificEpithet: feei; taxonRank: species; scientificNameAuthorship: Moran and Skevington 2019; vernacularName: Fee's Mucksucker; nomenclaturalStatus: new species; **Location:** country: U.S.A.; stateProvince: New Hampshire; locality: Coos County, Scott Bog, Connecticut Lakes; decimalLatitude: 45.217; decimalLongitude: -71.174; **Identification:** identifiedBy: K. Moran; **Event:** eventDate: 1987-06-05; year: 1987; month: 6; day: 5; verbatimEventDate: 5.vi.1987; habitat: Cornus stolonifera; **Record Level:** language: en; institutionCode: ANSP; basisOfRecord: PreservedSpecimen
- **Type status:** Paratype. **Occurrence:** catalogNumber: Jeff_Skevington_Specimen44216; recordedBy: F.D. Fee; individualCount: 1; sex: male; lifeStage: adult; **Taxon:** scientificName: Orthonevrafeei Moran and Skevington; kingdom: Animalia; phylum: Arthropoda; class: Hexapoda; order: Diptera; family: Syrphidae; genus: Orthonevra; specificEpithet: feei; taxonRank: species; scientificNameAuthorship: Moran and Skevington 2019; vernacularName: Fee's Mucksucker; nomenclaturalStatus: new species; **Location:** country: U.S.A.; stateProvince: New Hampshire; locality: Coos County, Scott Bog, Connecticut Lakes; decimalLatitude: 45.217; decimalLongitude: -71.174; **Identification:** identifiedBy: K. Moran; **Event:** eventDate: 1987-06-05; year: 1987; month: 6; day: 5; verbatimEventDate: 5.vi.1987; habitat: Cornus stolonifera; **Record Level:** language: en; institutionCode: ANSP; basisOfRecord: PreservedSpecimen
- **Type status:** Paratype. **Occurrence:** catalogNumber: Jeff_Skevington_Specimen44218; recordedBy: F.D. Fee; individualCount: 1; sex: male; lifeStage: adult; **Taxon:** scientificName: Orthonevrafeei Moran and Skevington; kingdom: Animalia; phylum: Arthropoda; class: Hexapoda; order: Diptera; family: Syrphidae; genus: Orthonevra; specificEpithet: feei; taxonRank: species; scientificNameAuthorship: Moran and Skevington 2019; vernacularName: Fee's Mucksucker; nomenclaturalStatus: new species; **Location:** country: U.S.A.; stateProvince: New Hampshire; locality: Coos County, Scott Bog, Connecticut Lakes; decimalLatitude: 45.217; decimalLongitude: -71.174; **Identification:** identifiedBy: K. Moran; **Event:** eventDate: 1987-06-05; year: 1987; month: 6; day: 5; verbatimEventDate: 5.vi.1987; habitat: Fragaria; **Record Level:** language: en; institutionCode: ANSP; basisOfRecord: PreservedSpecimen
- **Type status:** Paratype. **Occurrence:** catalogNumber: Jeff_Skevington_Specimen44219; recordedBy: F.D. Fee; individualCount: 1; sex: male; lifeStage: adult; **Taxon:** scientificName: Orthonevrafeei Moran and Skevington; kingdom: Animalia; phylum: Arthropoda; class: Hexapoda; order: Diptera; family: Syrphidae; genus: Orthonevra; specificEpithet: feei; taxonRank: species; scientificNameAuthorship: Moran and Skevington 2019; vernacularName: Fee's Mucksucker; nomenclaturalStatus: new species; **Location:** country: U.S.A.; stateProvince: New Hampshire; locality: Coos County, Scott Bog, Connecticut Lakes; decimalLatitude: 45.217; decimalLongitude: -71.174; **Identification:** identifiedBy: K. Moran; **Event:** eventDate: 1987-06-07; year: 1987; month: 6; day: 7; verbatimEventDate: 7.vi.1987; habitat: Fragaria; **Record Level:** language: en; institutionCode: CNC; basisOfRecord: PreservedSpecimen
- **Type status:** Paratype. **Occurrence:** catalogNumber: Jeff_Skevington_Specimen44220; recordedBy: F.D. Fee; individualCount: 1; sex: male; lifeStage: adult; **Taxon:** scientificName: Orthonevrafeei Moran and Skevington; kingdom: Animalia; phylum: Arthropoda; class: Hexapoda; order: Diptera; family: Syrphidae; genus: Orthonevra; specificEpithet: feei; taxonRank: species; scientificNameAuthorship: Moran and Skevington 2019; vernacularName: Fee's Mucksucker; nomenclaturalStatus: new species; **Location:** country: U.S.A.; stateProvince: New Hampshire; locality: Coos County, Scott Bog, Connecticut Lakes; decimalLatitude: 45.217; decimalLongitude: -71.174; **Identification:** identifiedBy: K. Moran; **Event:** eventDate: 1990-06-13; year: 1990; month: 6; day: 13; verbatimEventDate: 13.vi.1990; habitat: Fragaria; **Record Level:** language: en; institutionCode: CNC; basisOfRecord: PreservedSpecimen
- **Type status:** Paratype. **Occurrence:** catalogNumber: Jeff_Skevington_Specimen44221; recordedBy: F.D. Fee; individualCount: 1; sex: male; lifeStage: adult; **Taxon:** scientificName: Orthonevrafeei Moran and Skevington; kingdom: Animalia; phylum: Arthropoda; class: Hexapoda; order: Diptera; family: Syrphidae; genus: Orthonevra; specificEpithet: feei; taxonRank: species; scientificNameAuthorship: Moran and Skevington 2019; vernacularName: Fee's Mucksucker; nomenclaturalStatus: new species; **Location:** country: U.S.A.; stateProvince: New Hampshire; locality: Coos County, Scott Bog, Connecticut Lakes; decimalLatitude: 45.217; decimalLongitude: -71.174; **Identification:** identifiedBy: K. Moran; **Event:** eventDate: 1990-06-13; year: 1990; month: 6; day: 13; verbatimEventDate: 13.vi.1990; habitat: Fragaria; **Record Level:** language: en; institutionCode: ANSP; basisOfRecord: PreservedSpecimen
- **Type status:** Paratype. **Occurrence:** catalogNumber: Jeff_Skevington_Specimen44222; recordedBy: F.D. Fee; individualCount: 1; sex: male; lifeStage: adult; **Taxon:** scientificName: Orthonevrafeei Moran and Skevington; kingdom: Animalia; phylum: Arthropoda; class: Hexapoda; order: Diptera; family: Syrphidae; genus: Orthonevra; specificEpithet: feei; taxonRank: species; scientificNameAuthorship: Moran and Skevington 2019; vernacularName: Fee's Mucksucker; nomenclaturalStatus: new species; **Location:** country: U.S.A.; stateProvince: New Hampshire; locality: Coos County, Scott Bog, Connecticut Lakes; decimalLatitude: 45.217; decimalLongitude: -71.174; **Identification:** identifiedBy: K. Moran; **Event:** eventDate: 1990-06-13; year: 1990; month: 6; day: 13; verbatimEventDate: 13.vi.1990; habitat: Taraxacum; **Record Level:** language: en; institutionCode: ANSP; basisOfRecord: PreservedSpecimen
- **Type status:** Paratype. **Occurrence:** catalogNumber: Jeff_Skevington_Specimen44223; recordedBy: F.D. Fee; individualCount: 1; sex: male; lifeStage: adult; **Taxon:** scientificName: Orthonevrafeei Moran and Skevington; kingdom: Animalia; phylum: Arthropoda; class: Hexapoda; order: Diptera; family: Syrphidae; genus: Orthonevra; specificEpithet: feei; taxonRank: species; scientificNameAuthorship: Moran and Skevington 2019; vernacularName: Fee's Mucksucker; nomenclaturalStatus: new species; **Location:** country: U.S.A.; stateProvince: New Hampshire; locality: Coos County, Scott Bog, Connecticut Lakes; decimalLatitude: 45.217; decimalLongitude: -71.174; **Identification:** identifiedBy: K. Moran; **Event:** eventDate: 1990-06-13; year: 1990; month: 6; day: 13; verbatimEventDate: 13.vi.1990; habitat: Fragaria; **Record Level:** language: en; institutionCode: USNM; basisOfRecord: PreservedSpecimen
- **Type status:** Paratype. **Occurrence:** catalogNumber: Jeff_Skevington_Specimen44224; recordedBy: F.D. Fee; individualCount: 1; sex: male; lifeStage: adult; **Taxon:** scientificName: Orthonevrafeei Moran and Skevington; kingdom: Animalia; phylum: Arthropoda; class: Hexapoda; order: Diptera; family: Syrphidae; genus: Orthonevra; specificEpithet: feei; taxonRank: species; scientificNameAuthorship: Moran and Skevington 2019; vernacularName: Fee's Mucksucker; nomenclaturalStatus: new species; **Location:** country: U.S.A.; stateProvince: New Hampshire; locality: Coos County, Scott Bog, Connecticut Lakes; decimalLatitude: 45.217; decimalLongitude: -71.174; **Identification:** identifiedBy: K. Moran; **Event:** eventDate: 1990-06-13; year: 1990; month: 6; day: 13; verbatimEventDate: 13.vi.1990; habitat: Fragaria; **Record Level:** language: en; institutionCode: ANSP; basisOfRecord: PreservedSpecimen
- **Type status:** Paratype. **Occurrence:** catalogNumber: Jeff_Skevington_Specimen44225; recordedBy: F.D. Fee; individualCount: 1; sex: male; lifeStage: adult; **Taxon:** scientificName: Orthonevrafeei Moran and Skevington; kingdom: Animalia; phylum: Arthropoda; class: Hexapoda; order: Diptera; family: Syrphidae; genus: Orthonevra; specificEpithet: feei; taxonRank: species; scientificNameAuthorship: Moran and Skevington 2019; vernacularName: Fee's Mucksucker; nomenclaturalStatus: new species; **Location:** country: U.S.A.; stateProvince: New Hampshire; locality: Coos County, Scott Bog, Connecticut Lakes; decimalLatitude: 45.217; decimalLongitude: -71.174; **Identification:** identifiedBy: K. Moran; **Event:** eventDate: 1990-06-13; year: 1990; month: 6; day: 13; verbatimEventDate: 13.vi.1990; habitat: Fragaria; **Record Level:** language: en; institutionCode: ANSP; basisOfRecord: PreservedSpecimen
- **Type status:** Paratype. **Occurrence:** catalogNumber: Jeff_Skevington_Specimen44226; recordedBy: F.D. Fee; individualCount: 1; sex: male; lifeStage: adult; **Taxon:** scientificName: Orthonevrafeei Moran and Skevington; kingdom: Animalia; phylum: Arthropoda; class: Hexapoda; order: Diptera; family: Syrphidae; genus: Orthonevra; specificEpithet: feei; taxonRank: species; scientificNameAuthorship: Moran and Skevington 2019; vernacularName: Fee's Mucksucker; nomenclaturalStatus: new species; **Location:** country: U.S.A.; stateProvince: New Hampshire; locality: Coos County, Scott Bog, Connecticut Lakes; decimalLatitude: 45.217; decimalLongitude: -71.174; **Identification:** identifiedBy: K. Moran; **Event:** eventDate: 1990-06-14; year: 1990; month: 6; day: 14; verbatimEventDate: 14.vi.1990; habitat: Stellaria; **Record Level:** language: en; institutionCode: ANSP; basisOfRecord: PreservedSpecimen
- **Type status:** Paratype. **Occurrence:** catalogNumber: Jeff_Skevington_Specimen44227; recordedBy: F.D. Fee; individualCount: 1; sex: male; lifeStage: adult; **Taxon:** scientificName: Orthonevrafeei Moran and Skevington; kingdom: Animalia; phylum: Arthropoda; class: Hexapoda; order: Diptera; family: Syrphidae; genus: Orthonevra; specificEpithet: feei; taxonRank: species; scientificNameAuthorship: Moran and Skevington 2019; vernacularName: Fee's Mucksucker; nomenclaturalStatus: new species; **Location:** country: U.S.A.; stateProvince: New Hampshire; locality: Coos County, Scott Bog, Connecticut Lakes; decimalLatitude: 45.217; decimalLongitude: -71.174; **Identification:** identifiedBy: K. Moran; **Event:** eventDate: 1990-06-14; year: 1990; month: 6; day: 14; verbatimEventDate: 14.vi.1990; habitat: Fragaria; **Record Level:** language: en; institutionCode: USNM; basisOfRecord: PreservedSpecimen
- **Type status:** Paratype. **Occurrence:** catalogNumber: Moran and Skevington 2019; recordedBy: F.D. Fee; individualCount: 1; sex: male; lifeStage: adult; **Taxon:** scientificName: Orthonevrafeei Moran and Skevington; kingdom: Animalia; phylum: Arthropoda; class: Hexapoda; order: Diptera; family: Syrphidae; genus: Orthonevra; specificEpithet: feei; taxonRank: species; scientificNameAuthorship: Moran and Skevington; vernacularName: Fee's Mucksucker; nomenclaturalStatus: new species; **Location:** country: U.S.A.; stateProvince: New Hampshire; locality: Coos County, Scott Bog, Connecticut Lakes; decimalLatitude: 45.217; decimalLongitude: -71.174; **Identification:** identifiedBy: K. Moran; **Event:** eventDate: 1990-06-14; year: 1990; month: 6; day: 14; verbatimEventDate: 14.vi.1990; habitat: Fragaria; **Record Level:** language: en; institutionCode: ANSP; basisOfRecord: PreservedSpecimen
- **Type status:** Paratype. **Occurrence:** catalogNumber: Jeff_Skevington_Specimen44229; recordedBy: F.D. Fee; individualCount: 2; sex: male+female; lifeStage: adult; **Taxon:** scientificName: Orthonevrafeei Moran and Skevington; kingdom: Animalia; phylum: Arthropoda; class: Hexapoda; order: Diptera; family: Syrphidae; genus: Orthonevra; specificEpithet: feei; taxonRank: species; scientificNameAuthorship: Moran and Skevington 2019; vernacularName: Fee's Mucksucker; nomenclaturalStatus: new species; **Location:** country: U.S.A.; stateProvince: New Hampshire; locality: Coos County, Scott Bog, Connecticut Lakes; decimalLatitude: 45.217; decimalLongitude: -71.174; **Identification:** identifiedBy: K. Moran; **Event:** eventDate: 1990-06-14; year: 1990; month: 6; day: 14; verbatimEventDate: 14.vi.1990; habitat: in cop on flower Fragaria, female feeding on flower; **Record Level:** language: en; institutionCode: ANSP; basisOfRecord: PreservedSpecimen
- **Type status:** Paratype. **Occurrence:** catalogNumber: Jeff_Skevington_Specimen44230; recordedBy: F.D. Fee; individualCount: 1; sex: female; lifeStage: adult; **Taxon:** scientificName: Orthonevrafeei Moran and Skevington; kingdom: Animalia; phylum: Arthropoda; class: Hexapoda; order: Diptera; family: Syrphidae; genus: Orthonevra; specificEpithet: feei; taxonRank: species; scientificNameAuthorship: Moran and Skevington 2019; vernacularName: Fee's Mucksucker; nomenclaturalStatus: new species; **Location:** country: U.S.A.; stateProvince: New Hampshire; locality: Coos County, Scott Bog, Connecticut Lakes; decimalLatitude: 45.217; decimalLongitude: -71.174; **Identification:** identifiedBy: K. Moran; **Event:** eventDate: 1987-06-05; year: 1987; month: 6; day: 5; verbatimEventDate: 5.vi.1987; habitat: Cornus stolonifera; **Record Level:** language: en; institutionCode: ANSP; basisOfRecord: PreservedSpecimen
- **Type status:** Paratype. **Occurrence:** catalogNumber: Jeff_Skevington_Specimen44231; recordedBy: F.D. Fee; individualCount: 1; sex: female; lifeStage: adult; **Taxon:** scientificName: Orthonevrafeei Moran and Skevington; kingdom: Animalia; phylum: Arthropoda; class: Hexapoda; order: Diptera; family: Syrphidae; genus: Orthonevra; specificEpithet: feei; taxonRank: species; scientificNameAuthorship: Moran and Skevington 2019; vernacularName: Fee's Mucksucker; nomenclaturalStatus: new species; **Location:** country: U.S.A.; stateProvince: New Hampshire; locality: Coos County, Scott Bog, Connecticut Lakes; decimalLatitude: 45.217; decimalLongitude: -71.174; **Identification:** identifiedBy: K. Moran; **Event:** eventDate: 1987-06-06; year: 1987; month: 6; day: 6; verbatimEventDate: 6.vi.1987; habitat: Cornus stolonifera; **Record Level:** language: en; institutionCode: ANSP; basisOfRecord: PreservedSpecimen
- **Type status:** Paratype. **Occurrence:** catalogNumber: Jeff_Skevington_Specimen44232; recordedBy: F.D. Fee; individualCount: 1; sex: female; lifeStage: adult; **Taxon:** scientificName: Orthonevrafeei Moran and Skevington; kingdom: Animalia; phylum: Arthropoda; class: Hexapoda; order: Diptera; family: Syrphidae; genus: Orthonevra; specificEpithet: feei; taxonRank: species; scientificNameAuthorship: Moran and Skevington 2019; vernacularName: Fee's Mucksucker; nomenclaturalStatus: new species; **Location:** country: U.S.A.; stateProvince: New Hampshire; locality: Coos County, Scott Bog, Connecticut Lakes; decimalLatitude: 45.217; decimalLongitude: -71.174; **Identification:** identifiedBy: K. Moran; **Event:** eventDate: 1990-06-13; year: 1990; month: 6; day: 13; verbatimEventDate: 13.vi.1990; habitat: Fragaria; **Record Level:** language: en; institutionCode: ANSP; basisOfRecord: PreservedSpecimen
- **Type status:** Paratype. **Occurrence:** catalogNumber: Jeff_Skevington_Specimen44233; recordedBy: F.D. Fee; individualCount: 1; sex: female; lifeStage: adult; **Taxon:** scientificName: Orthonevrafeei Moran and Skevington; kingdom: Animalia; phylum: Arthropoda; class: Hexapoda; order: Diptera; family: Syrphidae; genus: Orthonevra; specificEpithet: feei; taxonRank: species; scientificNameAuthorship: Moran and Skevington 2019; vernacularName: Fee's Mucksucker; nomenclaturalStatus: new species; **Location:** country: U.S.A.; stateProvince: New Hampshire; locality: Coos County, Scott Bog, Connecticut Lakes; decimalLatitude: 45.217; decimalLongitude: -71.174; **Identification:** identifiedBy: K. Moran; **Event:** eventDate: 1990-06-14; year: 1990; month: 6; day: 14; verbatimEventDate: 14.vi.1990; habitat: Fragaria; **Record Level:** language: en; institutionCode: ANSP; basisOfRecord: PreservedSpecimen
- **Type status:** Paratype. **Occurrence:** catalogNumber: Jeff_Skevington_Specimen44234; recordedBy: F.D. Fee; individualCount: 1; sex: female; lifeStage: adult; **Taxon:** scientificName: Orthonevrafeei Moran and Skevington; kingdom: Animalia; phylum: Arthropoda; class: Hexapoda; order: Diptera; family: Syrphidae; genus: Orthonevra; specificEpithet: feei; taxonRank: species; scientificNameAuthorship: Moran and Skevington 2019; vernacularName: Fee's Mucksucker; nomenclaturalStatus: new species; **Location:** country: U.S.A.; stateProvince: New Hampshire; locality: Coos County, Scott Bog, Connecticut Lakes; decimalLatitude: 45.217; decimalLongitude: -71.174; **Identification:** identifiedBy: K. Moran; **Event:** eventDate: 1990-06-14; year: 1990; month: 6; day: 14; verbatimEventDate: 14.vi.1990; habitat: Fragaria; **Record Level:** language: en; institutionCode: USNM; basisOfRecord: PreservedSpecimen
- **Type status:** Paratype. **Occurrence:** catalogNumber: Jeff_Skevington_Specimen44235; recordedBy: F.D. Fee; individualCount: 1; sex: female; lifeStage: adult; **Taxon:** scientificName: Orthonevrafeei Moran and Skevington; kingdom: Animalia; phylum: Arthropoda; class: Hexapoda; order: Diptera; family: Syrphidae; genus: Orthonevra; specificEpithet: feei; taxonRank: species; scientificNameAuthorship: Moran and Skevington 2019; vernacularName: Fee's Mucksucker; nomenclaturalStatus: new species; **Location:** country: U.S.A.; stateProvince: New Hampshire; locality: Coos County, Scott Bog, Connecticut Lakes; decimalLatitude: 45.217; decimalLongitude: -71.174; **Identification:** identifiedBy: K. Moran; **Event:** eventDate: 1990-06-14; year: 1990; month: 6; day: 14; verbatimEventDate: 14.vi.1990; habitat: Fragaria; **Record Level:** language: en; institutionCode: ANSP; basisOfRecord: PreservedSpecimen
- **Type status:** Paratype. **Occurrence:** catalogNumber: Jeff_Skevington_Specimen44236; recordedBy: F.D. Fee; individualCount: 1; sex: female; lifeStage: adult; **Taxon:** scientificName: Orthonevrafeei Moran and Skevington; kingdom: Animalia; phylum: Arthropoda; class: Hexapoda; order: Diptera; family: Syrphidae; genus: Orthonevra; specificEpithet: feei; taxonRank: species; scientificNameAuthorship: Moran and Skevington 2019; vernacularName: Fee's Mucksucker; nomenclaturalStatus: new species; **Location:** country: U.S.A.; stateProvince: New Hampshire; locality: Coos County, Scott Bog, Connecticut Lakes; decimalLatitude: 45.217; decimalLongitude: -71.174; **Identification:** identifiedBy: K. Moran; **Event:** eventDate: 1990-06-14; year: 1990; month: 6; day: 14; verbatimEventDate: 14.vi.1990; habitat: Fragaria; **Record Level:** language: en; institutionCode: CNC; basisOfRecord: PreservedSpecimen
- **Type status:** Paratype. **Occurrence:** catalogNumber: Jeff_Skevington_Specimen44237; recordedBy: F.D. Fee; individualCount: 1; sex: female; lifeStage: adult; **Taxon:** scientificName: Orthonevrafeei Moran and Skevington; kingdom: Animalia; phylum: Arthropoda; class: Hexapoda; order: Diptera; family: Syrphidae; genus: Orthonevra; specificEpithet: feei; taxonRank: species; scientificNameAuthorship: Moran and Skevington 2019; vernacularName: Fee's Mucksucker; nomenclaturalStatus: new species; **Location:** country: U.S.A.; stateProvince: New Hampshire; locality: Coos County, Scott Bog, Connecticut Lakes; decimalLatitude: 45.217; decimalLongitude: -71.174; **Identification:** identifiedBy: K. Moran; **Event:** eventDate: 1990-06-14; year: 1990; month: 6; day: 14; verbatimEventDate: 14.vi.1990; habitat: Fragaria; **Record Level:** language: en; institutionCode: ANSP; basisOfRecord: PreservedSpecimen

#### Description

**Size**: Body length 5.4 to 6.5 mm; wing length 4.1 to 4.9 mm

**Description: Head**: Black; face concave with lower face projecting forwards along the oral margin, pale pilose along oral margin but bare above and shiny except with triangular silver area of pollinosity between antenna and margin of eye; gena pale pilose, shiny; frontal triangle shiny, pale pilose; vertex black, bare except ocellar triangle black pilose; occiput pale pilose with some short black setae on dorsal ¼ and shiny except for some white pollinosity along lower eye margin; antenna brown or brown and orange, scape and pedicel with short, black pile, pedicel about as long as scape, postpedicel elongate, about three times as long as wide, with minute sensory pit on inner surface, arista bare; eye bare, with no coloured markings and broadly holoptic.

**Thorax**: Mesonotum dominantly metallic blue, sometimes with a mix of green, bronze and or purple colouration, with differently coloured dorsomedial stripes and pale pilose; scutellum metallic blue, pale pilose; subscutellar fringe absent; postpronotum metallic blue, pale pilose; postalar callus metallic blue, pale pilose and shiny; pleuron metallic blue and shiny; anterior anepisternum, katepimeron and meron bare; posterior anepisternum and anepimeron pale pilose; katepisternum discontinuously pale pilose; metasternum black and bare; coxa brown, shiny and pale pilose; femora brown except apex yellow, pale pilose, except metafemur with black setae ventrally and shiny; tibia brown, except yellow on anterior third and at posterior joint, pale pilose and shiny; tarsomeres brown except 1st tarsomere yellow and black pilose ventrally; wing entirely densely microtrichose; halter yellow; calypter white.

**Abdomen**: Black; tergites 1-4 dull brown medially and shining laterally, black pilose medially and pale pilose laterally; sternites shining and pale pilose; postabdomen shining and pale pilose.

**Genitalia**: Epandrium subquadrate, longer than wide, narrows slightly dorsally; cercus with laterally compressed, subtriangular sclerotised outer portion covered in long pile and membranous inner portion; surstylus long, fingerlike, but curving inwards and with rounded apex, with conspicuous long pile on outer surface and inner surface; subepandrial sclerite broad, with narrow membranous v-shaped medial section; hypandrium with patch of macrosetae posterior to postgonite, ejaculatory apodeme conical; phallapodeme short and straight; postgonite long antler-like structure projecting ventrally, terminating in three spines; distiphallus elongated apically, narrow, projecting upwards between surstyli, with sharp pointed apex and long ventral tooth curving anteriorly ; phallus simple tube within hypandrium.

**Female**: Same as male except for usual sexual dimorphism.

See Fig. 12.

#### Diagnosis

Lacks eye markings typical of most *Orthonevra* species. Legs bi-coloured and postpedicel longer than scape plus pedicel combined.

#### Etymology

This species is named after Frank Fee, who collected all known specimens of the species. Frank was a prolific syrphid collector, returning to the same sites year after year in an effort to slowly build series around rare or new species.

#### Distribution

All known specimens were collected from Scott Bog in New Hampshire.

#### Ecology

All specimens of this rare species were collected in early to mid-June at a single location. Specimens were collected on *Cornussericea* , *Fragaria* and *Taraxacum*.

#### Taxon discussion

Attempts to sequence this species failed. It is clearly closely related to the other *Orthonevra* species that lack eye markings. *Orthonevrarobust*a also lacks eye markings but has entirely black legs and postpedicel shorter than scape plus pedicel combined.

#### Common Name

The common name given to the species by Skevington et al. (2019) is Fee's Mucksucker.

### Psilota klymkoi

Locke, Young and Skevington sp. n.

2344F09CFFC55A20B97BFB2563809DAD

urn:lsid:zoobank.org:act:EFDCD11A-7CEF-4364-A960-BFE625547300

#### Materials

- **Type status:** Holotype. **Occurrence:** catalogNumber: Jeff_Skevington_Specimen30434; recordedBy: J. Klymko, S.L. Robinson; individualCount: 1; sex: male; lifeStage: adult; otherCatalogNumbers: JK053333; **Taxon:** scientificName: Psilotaklymkoi Locke, Young and Skevington; kingdom: Animalia; phylum: Arthropoda; class: Hexapoda; order: Diptera; family: Syrphidae; genus: Psilota; specificEpithet: klymkoi; taxonRank: species; scientificNameAuthorship: Locke, Young and Skevington 2019; vernacularName: Black Haireye; nomenclaturalStatus: new species; **Location:** country: Canada; stateProvince: New Brunswick; locality: Albert Co., Caledonia Grg PNA, Tingley Road; decimalLatitude: 45.8304; decimalLongitude: -64.7784; **Identification:** identifiedBy: M.M. Locke; **Event:** samplingProtocol: hand collected; eventDate: 2013-06-10; year: 2013; month: 6; day: 10; verbatimEventDate: 10.vi.2013; habitat: mixed woods at edge of clearcut; **Record Level:** language: en; institutionCode: CNC; basisOfRecord: PreservedSpecimen
- **Type status:** Paratype. **Occurrence:** catalogNumber: CNC_Diptera106482; recordedBy: D.M.Wood; individualCount: 1; sex: female; lifeStage: adult; **Taxon:** scientificName: Psilotaklymkoi Locke, Young and Skevington; kingdom: Animalia; phylum: Arthropoda; class: Hexapoda; order: Diptera; family: Syrphidae; genus: Psilota; specificEpithet: klymkoi; taxonRank: species; scientificNameAuthorship: Locke, Young and Skevington 2019; vernacularName: Black Haireye; nomenclaturalStatus: new species; **Location:** country: Canada; stateProvince: Quebec; locality: Vaudrieul Co., Summit of Mount Rigaud; minimumElevationInMeters: 220; decimalLatitude: 45.466389; decimalLongitude: -74.326389; **Identification:** identifiedBy: M.M. Locke; **Event:** eventDate: 1995-05-31; year: 1995; month: 5; day: 31; verbatimEventDate: 31.v.1995; **Record Level:** language: en; institutionCode: CNC; basisOfRecord: PreservedSpecimen
- **Type status:** Paratype. **Occurrence:** catalogNumber: Jeff_Skevington_Specimen45312; recordedBy: F.D. Fee; individualCount: 1; sex: female; lifeStage: adult; **Taxon:** scientificName: Psilotaklymkoi Locke, Young and Skevington; kingdom: Animalia; phylum: Arthropoda; class: Hexapoda; order: Diptera; family: Syrphidae; genus: Psilota; specificEpithet: klymkoi; taxonRank: species; scientificNameAuthorship: Locke, Young and Skevington 2019; vernacularName: Black Haireye; nomenclaturalStatus: new species; **Location:** country: U.S.A.; stateProvince: Pennsylvania; locality: Huntingdon County, Ridge Road, Herod Run; decimalLatitude: 40.636043; decimalLongitude: -77.86749; **Identification:** identifiedBy: M.M. Locke; **Event:** eventDate: 1997-06-20; year: 1997; month: 6; day: 20; verbatimEventDate: 20.vi.1997; habitat: Pastinaca; **Record Level:** language: en; institutionCode: ANSP; basisOfRecord: PreservedSpecimen
- **Type status:** Paratype. **Occurrence:** catalogNumber: Jeff_Skevington_Specimen45313; recordedBy: F.D. Fee; individualCount: 1; sex: female; lifeStage: adult; **Taxon:** scientificName: Psilotaklymkoi Locke, Young and Skevington; kingdom: Animalia; phylum: Arthropoda; class: Hexapoda; order: Diptera; family: Syrphidae; genus: Psilota; specificEpithet: klymkoi; taxonRank: species; scientificNameAuthorship: Locke, Young and Skevington 2019; vernacularName: Black Haireye; nomenclaturalStatus: new species; **Location:** country: U.S.A.; stateProvince: Pennsylvania; locality: Huntingdon County, Ridge Road, Herod Run; decimalLatitude: 40.636043; decimalLongitude: -77.86749; **Identification:** identifiedBy: M.M. Locke; **Event:** eventDate: 1997-06-20; year: 1997; month: 6; day: 20; verbatimEventDate: 20.vi.1997; habitat: Pastinaca; **Record Level:** language: en; institutionCode: ANSP; basisOfRecord: PreservedSpecimen
- **Type status:** Paratype. **Occurrence:** catalogNumber: Jeff_Skevington_Specimen45315; recordedBy: F.D. Fee; individualCount: 1; sex: female; lifeStage: adult; **Taxon:** scientificName: Psilotaklymkoi Locke, Young and Skevington; kingdom: Animalia; phylum: Arthropoda; class: Hexapoda; order: Diptera; family: Syrphidae; genus: Psilota; specificEpithet: klymkoi; taxonRank: species; scientificNameAuthorship: Locke, Young and Skevington 2019; vernacularName: Black Haireye; nomenclaturalStatus: new species; **Location:** country: U.S.A.; stateProvince: Pennsylvania; locality: Centre County, Black Moshannon State Park; decimalLatitude: 40.915503; decimalLongitude: -78.05934; **Identification:** identifiedBy: M.M. Locke; **Event:** eventDate: 1999-05-20; year: 1999; month: 5; day: 20; verbatimEventDate: 20.v.1999; habitat: Pine/blueberry area, on twig; **Record Level:** language: en; institutionCode: ANSP; basisOfRecord: PreservedSpecimen

#### Description

**Size**: Body length 5.7 to 7.0 mm; wing length 5.1 to 6.0 mm

**Male: Head**: Face mostly shiny black with scattered pollinosity along central midline of face and dense pollinosity adjacent to compound eye, mostly black pilose with a few white pili near the oral margin; frons and vertex black pilose; antenna almost entirely black, very slightly orangish-brown on ventral side, postpedicel 1.5 times as long as wide; eye densely yellow pilose.

**Thorax**: Scutum and scutellum shiny black, entirely black pilose; pleuron shiny black, pollen-free on anepisternum, katepisternum and anepimeron, other areas of pleuron with scattered pollinosity, pile almost entirely black, with pale pile only on ventral half of katepisternum; legs mostly black, with apices of pro- and meso femora, bases of pro- and meso tibia and ventral side of basitarsomeres dull orange; wing hyaline, with a small bare area at the base of cell c, anterobasal halves of cells bm and cua bare; alula completely microtrichose; halter orange; calypter pale brown, pile at edge of calypter brown.

**Abdomen**: Tergites black; tergite 1 yellowish-white pilose; tergite 2 with some white pile anteriorly, otherwise entirely black pilose; tergite 3 almost entirely black pilose, with a few scattered white pili; tergite 4 with mixed black and white pile, with posterior half of tergite entirely white pilose; sternites shining, white pilose.

**Genitalia**: Epandrium compact, about as long as wide; cercus subquadrate, with apex rounded and slightly wider at base; outer lobe of surstylus inserted at dorsal edge of of inner lobe, curved downwards smoothly over its entire length and narrowing slightly towards apex; inner lobe of surstylus broadening very slightly over basal ¾, expanding more abruptly to a rounded tip at apex; hypandrium narrowing smoothly towards apex; phallapodeme smooth, with ventral projection; postgonite thin, fused to hypandrium, with tip slightly expanded; phallus with a series of small, ventral spines.

**Female**: Similar to male, differing as follows: antenna more extensively orange, with only dorsal edge brown; face with pollen restricted to lateral edges, entirely free of pollen medially, pile entirely white; frons with mixed black and white pilosity; leg with orange areas more extensively orange, tarsomeres entirely orange ventrally; katepisternum with mixed black and white pilosity on dorsal half as well as ventral; calypter pale yellowish-white; tergite 2 with pile variable, ranging from entirely black throughout to white pilose anteriorly, with white pile not reaching lateral edge of tergite.

See Fig. 13.

#### Diagnosis

Entirely black, shining species with face and frons black pilose. postpedicel 1.5 times as long as wide. Scutum entirely black pilose, scutellum entirely black pilose, pleuron entirely black pilose except for some pale pile on ventral half of katepisternum. Tarsomeres dark brown. Hind coxa mostly white pilose. Tergite 2 mostly black pilose laterally. Sternite 1 shining. All sternites entirely white pilose.

#### Etymology

Named after John Klymko, who collected the holotype and suggested that it may be an undescribed species. John is a zoologist at the Atlantic Canada Conservation Data Centre, where he works on syrphids and many other animals.

#### Distribution

This species is known from six specimens from Quebec, New Brunswick and Pennsylvania.

#### Ecology

This rare species has been collected from late May to mid-June. It has been found in mixed woods at the edge of a clearing, in an area of pines and blueberries, on an open hilltop in mixed forest and nectaring on *Pastinaca* .

#### Taxon discussion

One COI barcode sequence of this species was obtained (Table 2) .

#### Common Name

The common name given to the species by Skevington et al. (2019) is Black Haireye.

### Trichopsomyia litoralis

Vockeroth and Young sp. n.

9C885CF5960B58A6BEAA387D5039A113

urn:lsid:zoobank.org:act:1C301DA0-5649-4222-B05D-99C98BEC858F

#### Materials

- **Type status:** Holotype. **Occurrence:** catalogNumber: CNC_Diptera246378; recordedBy: J. McDunnough; individualCount: 1; sex: male; lifeStage: adult; **Taxon:** scientificName: Trichopsomyialitoralis Vockeroth and Young; kingdom: Animalia; phylum: Arthropoda; class: Hexapoda; order: Diptera; family: Syrphidae; genus: Trichopsomyia; specificEpithet: litoralis; taxonRank: species; scientificNameAuthorship: Vockeroth and Young 2019; vernacularName: Coastal Psyllid-killer; nomenclaturalStatus: new species; **Location:** country: Canada; stateProvince: Prince Edward Island; locality: Brackley Beach, Can. National Park [Prince Edward Island National Park]; decimalLatitude: 46.431111; decimalLongitude: -63.216111; **Identification:** identifiedBy: J.R. Vockeroth; **Event:** eventDate: 1940-07-30; year: 1940; month: 7; day: 30; verbatimEventDate: 30.vii.1940; **Record Level:** language: en; institutionCode: CNC; basisOfRecord: PreservedSpecimen
- **Type status:** Paratype. **Occurrence:** catalogNumber: CNC_Diptera110356; recordedBy: J.R. Vockeroth; individualCount: 1; lifeStage: adult; **Taxon:** scientificName: Trichopsomyialitoralis Vockeroth and Young; kingdom: Animalia; phylum: Arthropoda; class: Hexapoda; order: Diptera; family: Syrphidae; genus: Trichopsomyia; specificEpithet: litoralis; taxonRank: species; scientificNameAuthorship: Vockeroth and Young 2019; vernacularName: Coastal Psyllid-killer; nomenclaturalStatus: new species; **Location:** country: Canada; stateProvince: Nova Scotia; locality: South Harbour Beach; decimalLatitude: 46.878289; decimalLongitude: -60.429056; **Identification:** identifiedBy: J.R. Vockeroth; **Event:** eventDate: 1983-07-03; year: 1983; month: 7; day: 3; verbatimEventDate: 3.vii.1983; habitat: sand beach, Ammophila & Lathyrus; **Record Level:** language: en; institutionCode: CNC; basisOfRecord: PreservedSpecimen
- **Type status:** Paratype. **Occurrence:** catalogNumber: CNC_Diptera246379; recordedBy: D.M. Wood; individualCount: 1; sex: male; lifeStage: adult; **Taxon:** scientificName: Trichopsomyialitoralis Vockeroth and Young; kingdom: Animalia; phylum: Arthropoda; class: Hexapoda; order: Diptera; family: Syrphidae; genus: Trichopsomyia; specificEpithet: litoralis; taxonRank: species; scientificNameAuthorship: Vockeroth and Young 2019; vernacularName: Coastal Psyllid-killer; nomenclaturalStatus: new species; **Location:** country: Canada; stateProvince: Prince Edward Island; locality: Green Gables, Cavendish Beach; decimalLatitude: 46.5018; decimalLongitude: -63.4204; **Identification:** identifiedBy: J.R. Vockeroth; **Event:** eventDate: 1967-07-22; year: 1967; month: 7; day: 22; verbatimEventDate: 22.vii.1967; **Record Level:** language: en; institutionCode: CNC; basisOfRecord: PreservedSpecimen
- **Type status:** Paratype. **Occurrence:** catalogNumber: CNC_Diptera246380; recordedBy: D.M. Wood; individualCount: 1; sex: female; lifeStage: adult; **Taxon:** scientificName: Trichopsomyialitoralis Vockeroth and Young; kingdom: Animalia; phylum: Arthropoda; class: Hexapoda; order: Diptera; family: Syrphidae; genus: Trichopsomyia; specificEpithet: litoralis; taxonRank: species; scientificNameAuthorship: Vockeroth and Young 2019; vernacularName: Coastal Psyllid-killer; nomenclaturalStatus: new species; **Location:** country: Canada; stateProvince: Prince Edward Island; locality: Green Gables, Cavendish Beach; decimalLatitude: 46.5018; decimalLongitude: -63.4204; **Identification:** identifiedBy: J.R. Vockeroth; **Event:** eventDate: 1967-07-22; year: 1967; month: 7; day: 22; verbatimEventDate: 22.vii.1967; **Record Level:** language: en; institutionCode: CNC; basisOfRecord: PreservedSpecimen
- **Type status:** Paratype. **Occurrence:** catalogNumber: CNC_Diptera246381; recordedBy: D.M. Wood; individualCount: 1; sex: female; lifeStage: adult; **Taxon:** scientificName: Trichopsomyialitoralis Vockeroth and Young; kingdom: Animalia; phylum: Arthropoda; class: Hexapoda; order: Diptera; family: Syrphidae; genus: Trichopsomyia; specificEpithet: litoralis; taxonRank: species; scientificNameAuthorship: Vockeroth and Young 2019; vernacularName: Coastal Psyllid-killer; nomenclaturalStatus: new species; **Location:** country: Canada; stateProvince: Prince Edward Island; locality: Green Gables, Cavendish Beach; decimalLatitude: 46.5018; decimalLongitude: -63.4204; **Identification:** identifiedBy: J.R. Vockeroth; **Event:** eventDate: 1967-07-22; year: 1967; month: 7; day: 22; verbatimEventDate: 22.vii.1967; **Record Level:** language: en; institutionCode: CNC; basisOfRecord: PreservedSpecimen
- **Type status:** Paratype. **Occurrence:** catalogNumber: CNC_Diptera55044; recordedBy: C.W. Johnson; individualCount: 1; sex: male; lifeStage: adult; **Taxon:** scientificName: Trichopsomyialitoralis Vockeroth and Young; kingdom: Animalia; phylum: Arthropoda; class: Hexapoda; order: Diptera; family: Syrphidae; genus: Trichopsomyia; specificEpithet: litoralis; taxonRank: species; scientificNameAuthorship: Vockeroth and Young 2019; vernacularName: Coastal Psyllid-killer; nomenclaturalStatus: new species; **Location:** country: U.S.A.; stateProvince: New Jersey; locality: Cape May; decimalLatitude: 38.935112; decimalLongitude: -74.906005; **Identification:** identifiedBy: J.R. Vockeroth; **Event:** eventDate: 06-03; month: 6; day: 3; verbatimEventDate: 3.vi; **Record Level:** language: en; institutionCode: CNC; basisOfRecord: PreservedSpecimen
- **Type status:** Paratype. **Occurrence:** catalogNumber: CNC_Diptera55045; recordedBy: G.S. Walley; individualCount: 1; sex: male; lifeStage: adult; **Taxon:** scientificName: Trichopsomyialitoralis Vockeroth and Young; kingdom: Animalia; phylum: Arthropoda; class: Hexapoda; order: Diptera; family: Syrphidae; genus: Trichopsomyia; specificEpithet: litoralis; taxonRank: species; scientificNameAuthorship: Vockeroth and Young 2019; vernacularName: Coastal Psyllid-killer; nomenclaturalStatus: new species; **Location:** country: Canada; stateProvince: Prince Edward Island; locality: Dalvay House, Can. Nat. Park; decimalLatitude: 46.414905; decimalLongitude: -63.073145; **Identification:** identifiedBy: J.R. Vockeroth; **Event:** eventDate: 1940-07-21; year: 1940; month: 7; day: 21; verbatimEventDate: 21.vii.1940; **Record Level:** language: en; institutionCode: CNC; basisOfRecord: PreservedSpecimen
- **Type status:** Paratype. **Occurrence:** catalogNumber: CNC_Diptera55046; recordedBy: G.S. Walley; individualCount: 1; sex: female; lifeStage: adult; **Taxon:** scientificName: Trichopsomyialitoralis Vockeroth and Young; kingdom: Animalia; phylum: Arthropoda; class: Hexapoda; order: Diptera; family: Syrphidae; genus: Trichopsomyia; specificEpithet: litoralis; taxonRank: species; scientificNameAuthorship: Vockeroth and Young 2019; vernacularName: Coastal Psyllid-killer; nomenclaturalStatus: new species; **Location:** country: Canada; stateProvince: Prince Edward Island; locality: Dalvay House, Can. Nat. Park; decimalLatitude: 46.414905; decimalLongitude: -63.073145; **Identification:** identifiedBy: J.R. Vockeroth; **Event:** eventDate: 1940-07-21; year: 1940; month: 7; day: 21; verbatimEventDate: 21.vii.1940; **Record Level:** language: en; institutionCode: CNC; basisOfRecord: PreservedSpecimen
- **Type status:** Paratype. **Occurrence:** catalogNumber: CNC_Diptera55047; recordedBy: J. McDunnough; individualCount: 1; sex: female; lifeStage: adult; **Taxon:** scientificName: Trichopsomyialitoralis Vockeroth and Young; kingdom: Animalia; phylum: Arthropoda; class: Hexapoda; order: Diptera; family: Syrphidae; genus: Trichopsomyia; specificEpithet: litoralis; taxonRank: species; scientificNameAuthorship: Vockeroth and Young 2019; vernacularName: Coastal Psyllid-killer; nomenclaturalStatus: new species; **Location:** country: Canada; stateProvince: Prince Edward Island; locality: Dalvay House, Can. Nat. Park; decimalLatitude: 46.414905; decimalLongitude: -63.073145; **Identification:** identifiedBy: J.R. Vockeroth; **Event:** eventDate: 1940-07-21; year: 1940; month: 7; day: 21; verbatimEventDate: 21.vii.1940; **Record Level:** language: en; institutionCode: CNC; basisOfRecord: PreservedSpecimen
- **Type status:** Paratype. **Occurrence:** catalogNumber: CNC_Diptera9769; recordedBy: R. Dow; individualCount: 1; sex: male; lifeStage: adult; **Taxon:** scientificName: Trichopsomyialitoralis Vockeroth and Young; kingdom: Animalia; phylum: Arthropoda; class: Hexapoda; order: Diptera; family: Syrphidae; genus: Trichopsomyia; specificEpithet: litoralis; taxonRank: species; scientificNameAuthorship: Vockeroth and Young 2019; vernacularName: Coastal Psyllid-killer; nomenclaturalStatus: new species; **Location:** country: U.S.A.; stateProvince: Massachusetts; locality: Gloucester; minimumElevationInMeters: 16; decimalLatitude: 42.6; decimalLongitude: -70.633333; **Identification:** identifiedBy: J.R. Vockeroth; **Event:** eventDate: 1933-06-11; year: 1933; month: 6; day: 11; verbatimEventDate: 11.vi.1933; **Record Level:** language: en; institutionCode: CNC; basisOfRecord: PreservedSpecimen
- **Type status:** Paratype. **Occurrence:** catalogNumber: USNMENT247710; recordedBy: N.E. Woodley; individualCount: 1; sex: female; lifeStage: adult; **Taxon:** scientificName: Trichopsomyialitoralis Vockeroth and Young; kingdom: Animalia; phylum: Arthropoda; class: Hexapoda; order: Diptera; family: Syrphidae; genus: Trichopsomyia; specificEpithet: litoralis; taxonRank: species; scientificNameAuthorship: Vockeroth and Young 2019; vernacularName: Coastal Psyllid-killer; nomenclaturalStatus: new species; **Location:** country: U.S.A.; stateProvince: Virginia; locality: Accomack County, South End of Assateague Island; decimalLatitude: 38.087029; decimalLongitude: -75.217788; **Identification:** identifiedBy: J.R. Vockeroth; **Event:** eventDate: 1997-09-24; year: 1997; month: 9; day: 24; verbatimEventDate: 24.ix.1997; **Record Level:** language: en; institutionCode: USNM; basisOfRecord: PreservedSpecimen
- **Type status:** Paratype. **Occurrence:** catalogNumber: USNMENT247711; recordedBy: N.E. Woodley; individualCount: 1; sex: male; lifeStage: adult; **Taxon:** scientificName: Trichopsomyialitoralis Vockeroth and Young; kingdom: Animalia; phylum: Arthropoda; class: Hexapoda; order: Diptera; family: Syrphidae; genus: Trichopsomyia; specificEpithet: litoralis; taxonRank: species; scientificNameAuthorship: Vockeroth and Young 2019; vernacularName: Coastal Psyllid-killer; nomenclaturalStatus: new species; **Location:** country: U.S.A.; stateProvince: Virginia; locality: Accomack County, South End of Assateague Island; decimalLatitude: 38.087029; decimalLongitude: -75.217788; **Identification:** identifiedBy: J.R. Vockeroth; **Event:** eventDate: 1997-09-24; year: 1997; month: 9; day: 24; verbatimEventDate: 24.ix.1997; **Record Level:** language: en; institutionCode: USNM; basisOfRecord: PreservedSpecimen

#### Description

**Size**: Body length 5.5 to 6.0 mm; wing length 4.2 to 4.4 mm

**Male: Head**: Black; face and frontal triangle white pilose, white microtrichose only on lateral area touching compound eye; gena shining and white pilose; frontal lunule brown; vertical triangle shiny, white pilose; occiput silvery white pollinose, white pilose; scape brown, pedicel brown with orange at apex, black pilose; postpedicel brown, with a ventral orange stripe, arista bare, orange at base and brown on apical ⅓, approximately as long as postpedicel. Eye densely covered in short brown pilosity, very narrowly dichoptic dorsally, separated by about half the width of an ocellus posteriorly and nearly touching for the length of one ocelli anteriorly.

**Thorax**: Black, shining, white pilose; proepimeron and anterior anepisternum thinly white pollinose; scutum shiny, long white pilose; scutellum shiny, long white pilose; subscutellar fringe short, sparse, white, absent in middle ⅓ of scutellum; calypter white; halter whitish-yellow; metathoracic spiracular fringe white. Wing: hyaline, microtrichose except bare as follows: cell h, basal ½ cell c, cell br except for scattered microtrichia, basal ¼ cell r_1_, basoventral ⅔ cell bm, base of cell r_4+5_ and anterobasal edge of cell cua; alula microtrichose. Legs: coxae black, procoxa silvery white pollinose, meso- and metacoxa shiny, all coxa white pilose; femora black except apical ⅛ of pro- and mesofemora yellow and extreme apex of metafemur yellow, white pilose; protibia yellow, with antero- and ventrolateral ¾ brown, mesotibia yellow with anterior ½ brown excluding apex, metatibia brown with basal ⅙ yellow, all tibiae white pilose except apicoventrally on metatibia, which has appressed brownish-orange pile; protarsus with basitarsomere orange and apical 4 tarsomeres brown, apical 3 tarsomeres black pilose dorsally, tarsus otherwise white pilose; mesotarsomere with basal 2 tarsomeres orange and apical 3 tarsomeres brown, tarsomere 2 with a few black pili dorsally, apical 3 tarsomeres black pilose dorsally, tarsus otherwise white pilose; metatarsus mostly brown, with only apex of basitarsomere, tarsomere 2 and tarsomere 3 orange, basitarsomere and tarsomere 2 with long white anterodorsal pile, tarsus otherwise with short white pile, with a few long dorsal black pili on tarsomere 2 and with tarsomeres 3-5 with dorsal pile black.

**Abdomen**: Black, white pilose; all tergites shining green and white pilose; sternite 1 brown anteriorly and white posteriorly, shiny, white pilose; sternites 2 and 3 brown, shiny, white pilose, sternite 4 brown, with ventral edge raised smoothly, forming a cup where the male genitalia sits partially inside, medially thinly white pilose, white pollinose; 8th segment black, shiny, white pilose.

**Genitalia**: Epandrium elongate, about as twice as long as tall at the broadest point; cercus low, triangular, approximately half as tall as broad; surstylus curved, double-lobed, with large lateral lobe narrowing towards apex, with two broad, dorsal teeth on inner surface near base and a very small, branching, comb-like ventral lobe near base; subepandrial sclerite projecting anteriorly between surstyli and broadened at apex; distiphallus simple, rounded and microtrichose; postgonite broad, approximately twice as high as long, with a series of small teeth at apex.

**Female**: Similar to male except for normal sexual dimorphism and: front shiny except for two lateral pollinose markings on medial ⅓, white pilose; cell r_4+5_ more extensively bare, approximately basal ⅕ bare; probasitarsomere brown.

See Fig. 14 & Fig. 15.

#### Diagnosis

Males with eyes separated by about half the width of the anterior ocellus, nearly in contact for only a short distance; metatarsus with white anterodorsal pile. Female with cell dm microtrichose at base, sometimes with small bare area along anterior and posterior margins; scutellar pile long. Both sexes with cell r_4+5_ bare at base.

#### Etymology

The word *litoralis* is Latin, meaning of the seashore.

#### Distribution

This species is known from Nova Scotia, Prince Edward Island, Massachusetts, New Jersey and Virginia.

#### Ecology

This rare fly has been collected from early June to late September along the east coast on beaches; one was described as a sand beach with *Ammophila* and *Lathyrus* plants present.

#### Taxon discussion

A partial COI barcode was obtained from one specimen (Table 2).

#### Common Name

The common name given to the species by Skevington et al. (2019) is Coastal Psyllid-killer.

## Supplementary Material

XML Treatment for Anasimyia diffusa

XML Treatment for Anasimyia matutina

XML Treatment for Brachyopa caesariata

XML Treatment for Brachyopa cummingi

XML Treatment for Hammerschmidtia sedmani

XML Treatment for Microdon (Microdon) scauros

XML Treatment for Mixogaster fattigi

XML Treatment for Neoascia guttata

XML Treatment for Orthonevra feei

XML Treatment for Psilota klymkoi

XML Treatment for Trichopsomyia litoralis

## Figures and Tables

**Figure 1a.**
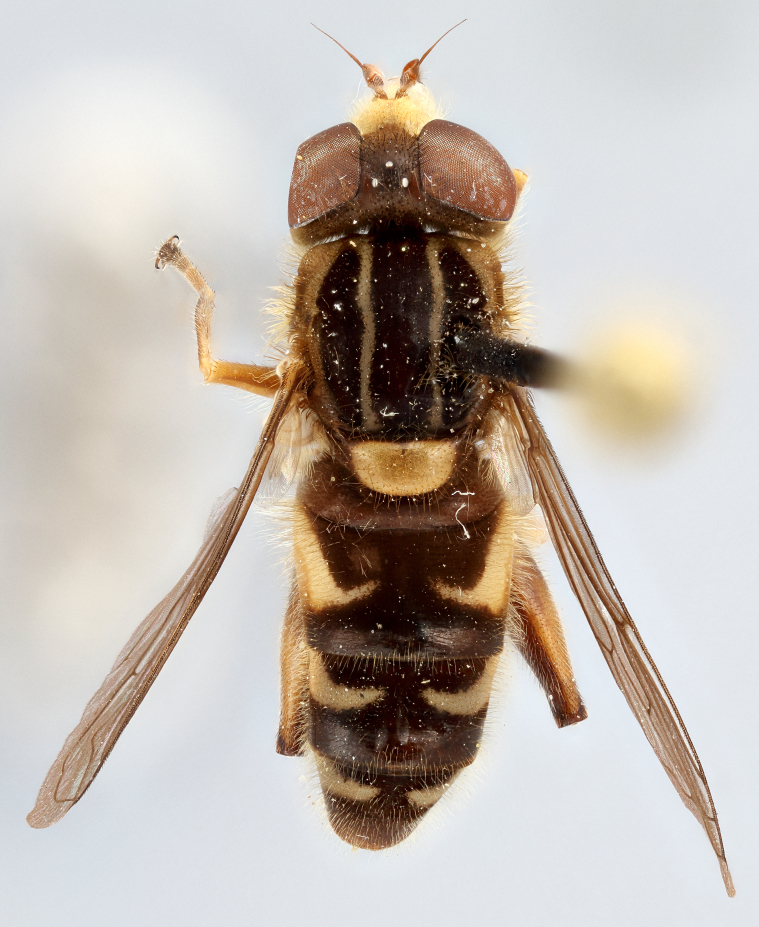
Dorsal habitus, CNC_Diptera44630

**Figure 1b.**
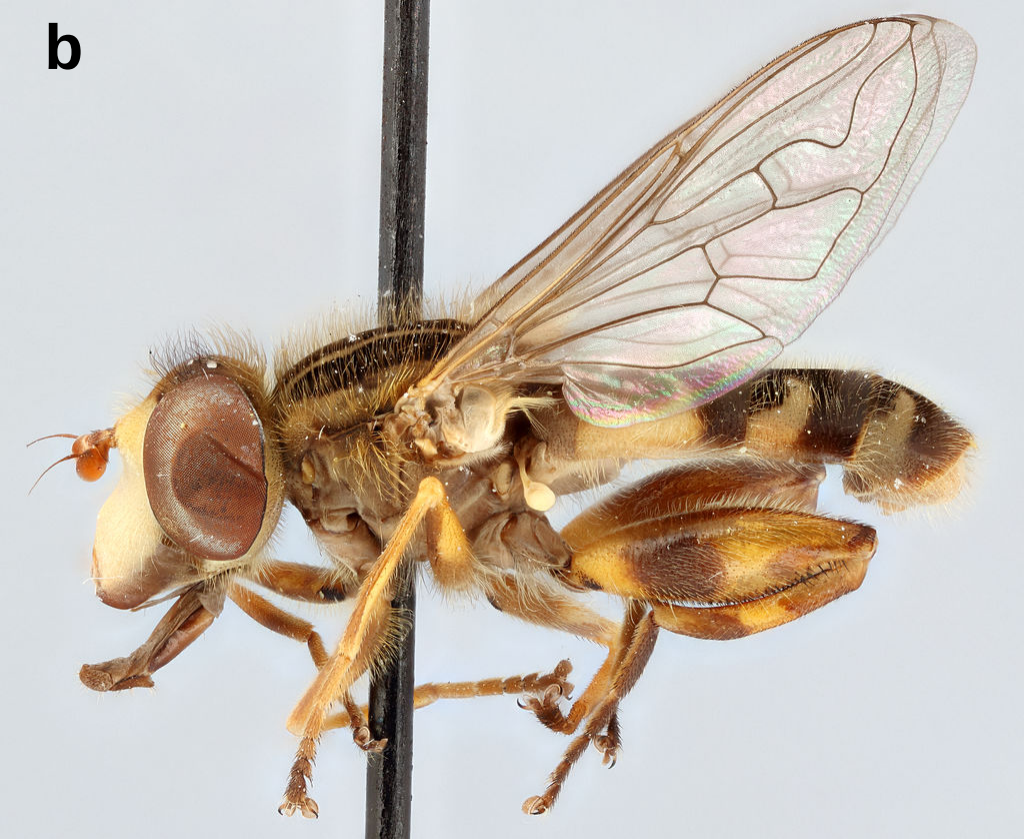
Lateral habitus, CNC_Diptera44630

**Figure 1c.**
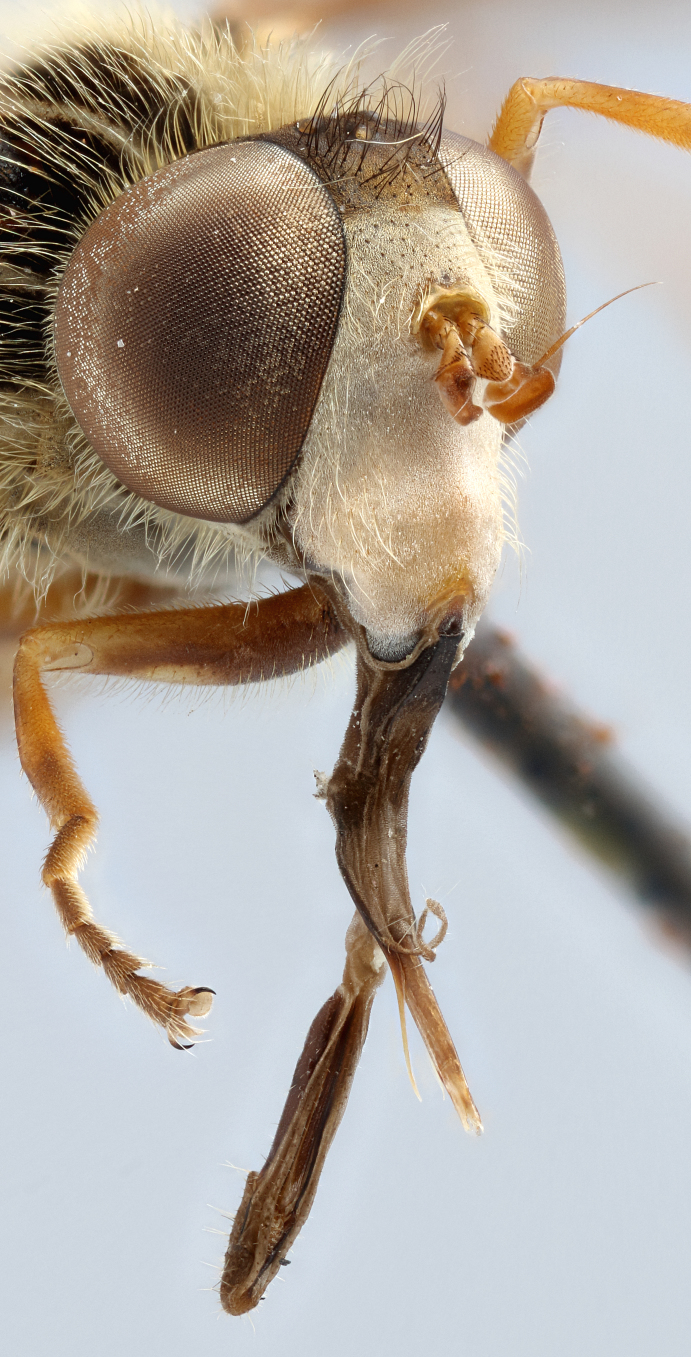
Face, oblique, Holotype, CNC_Diptera102201

**Figure 1d.**
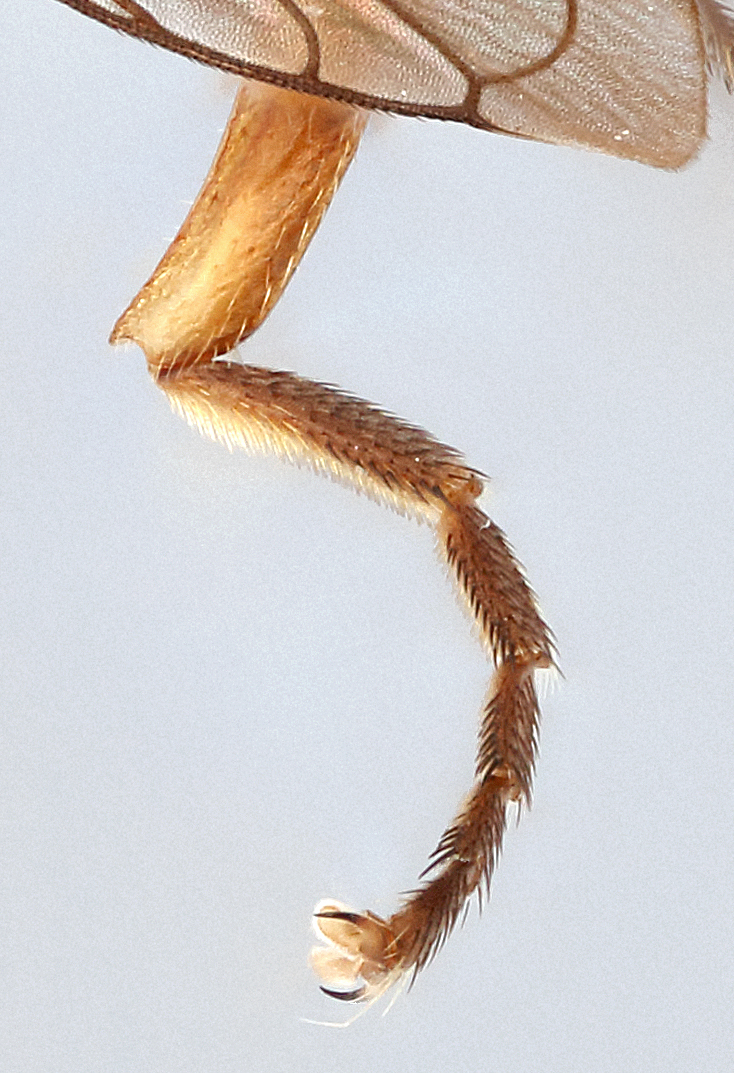
Metaleg, lateral, Holotype, CNC_Diptera102201

**Figure 1e.**
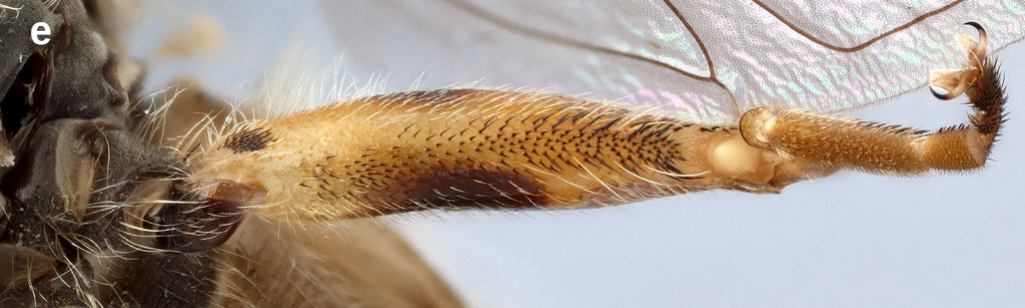
Metaleg, ventral, Holotype, CNC_Diptera102201

**Figure 2a.**
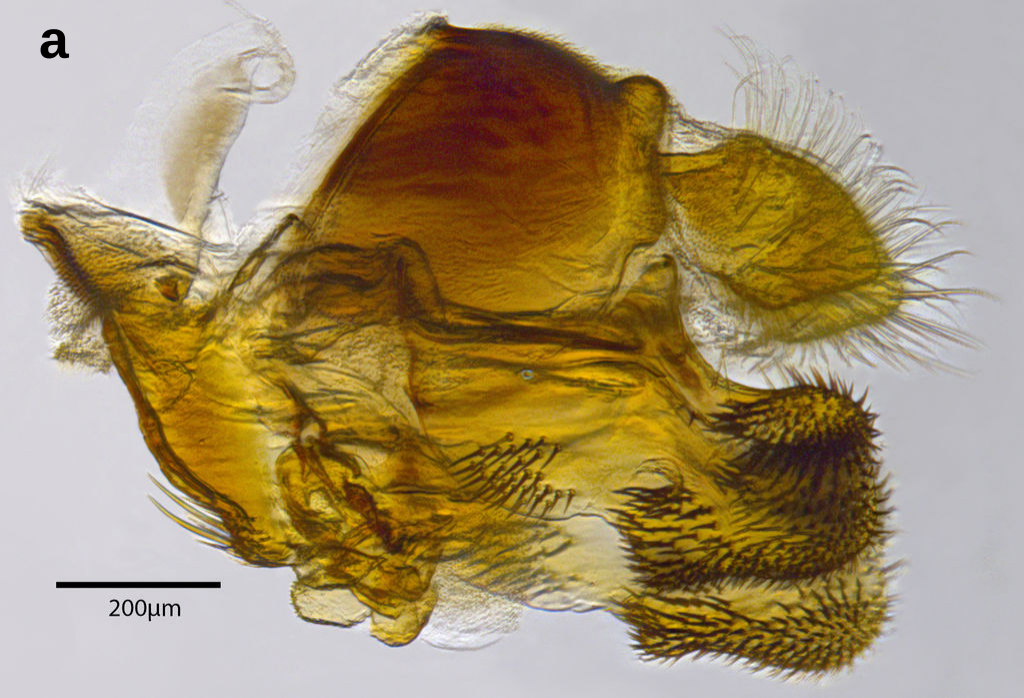
Lateral terminalia, Jeff_Skevington_Specimen17434

**Figure 2b.**
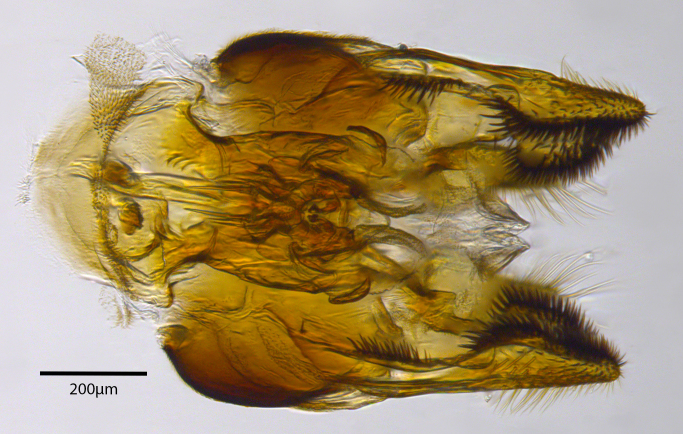
Ventral terminalia, Jeff_Skevington_Specimen17434

**Figure 2c.**
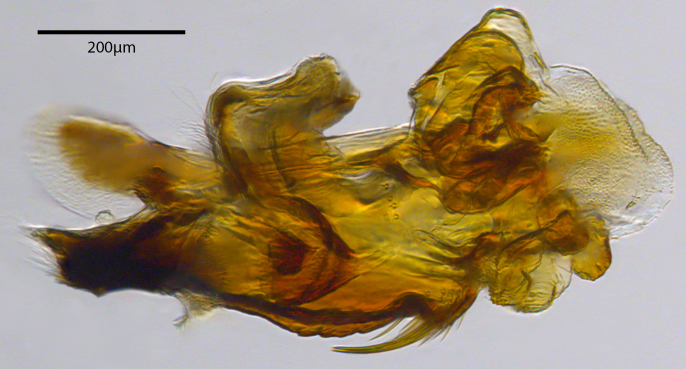
Lateral hypandrium, Jeff_Skevington_Specimen17434

**Figure 2d.**
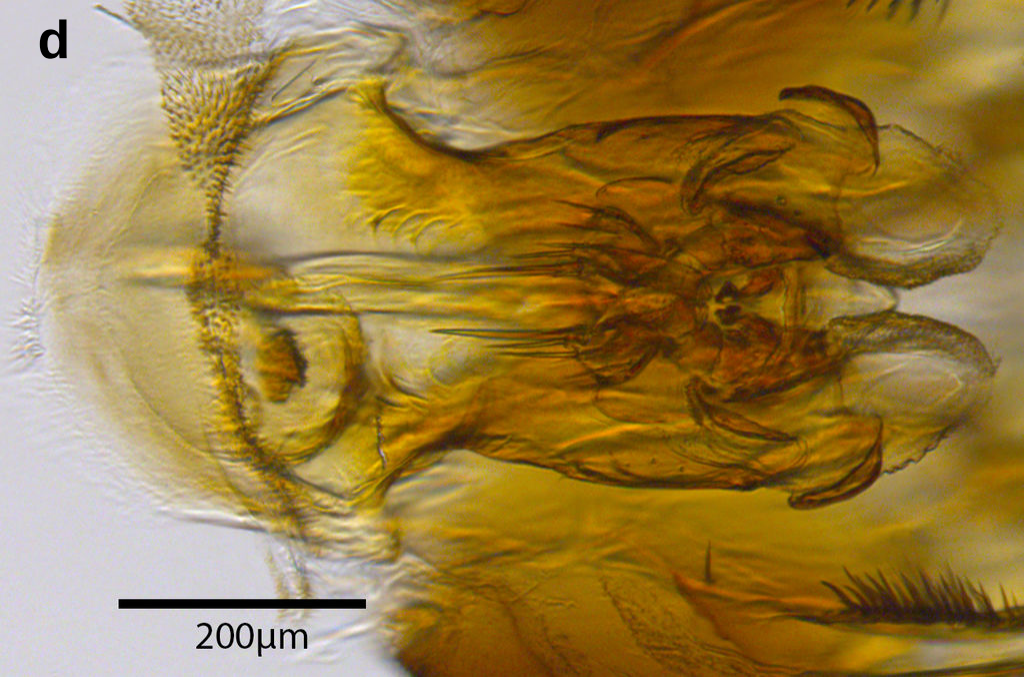
Ventral of hypandrium, Jeff_Skevington_Specimen17434

**Figure 3a.**
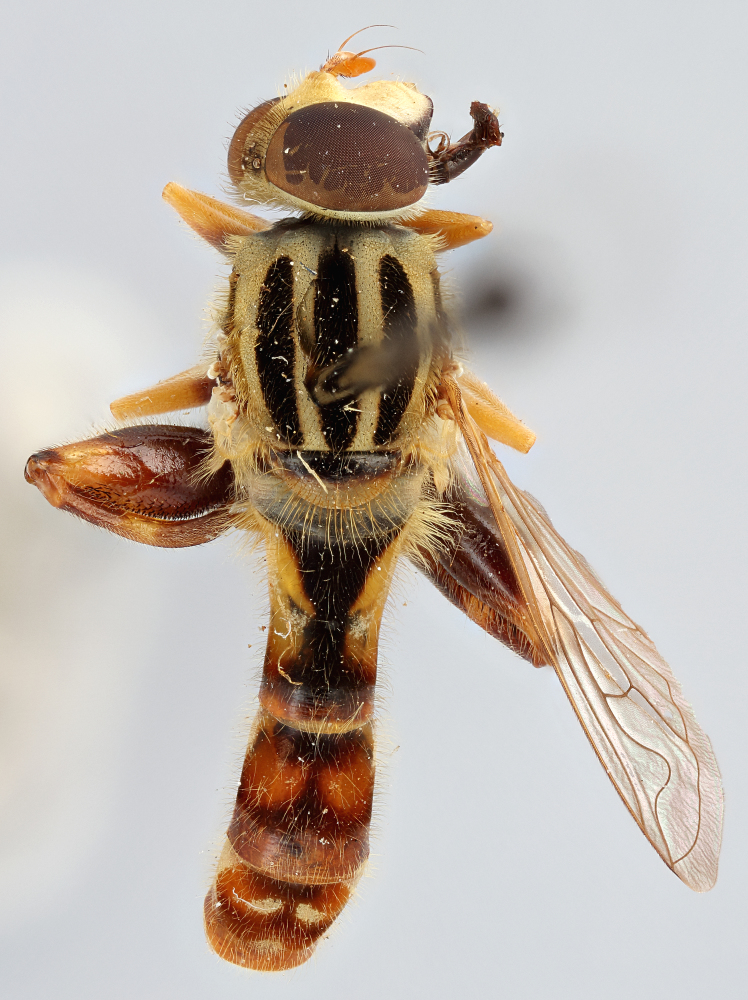
Dorsal habitus, Holotype, CNC_Diptera91232

**Figure 3b.**
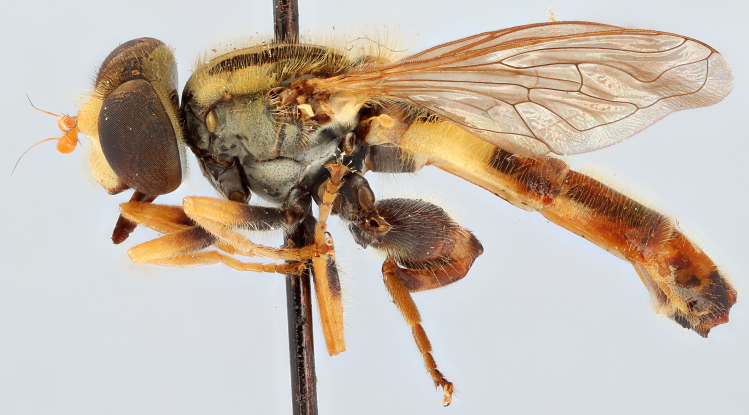
Lateral habitus, Jeff_Skevington_Specimen17432

**Figure 3c.**
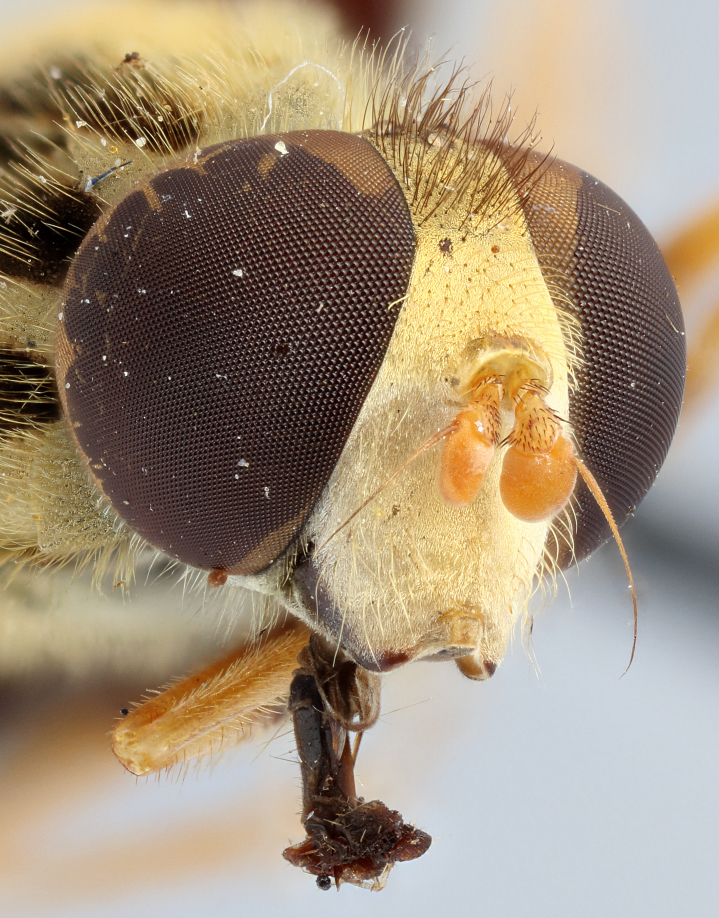
Head, oblique, Holotype, CNC_Diptera91232

**Figure 3d.**
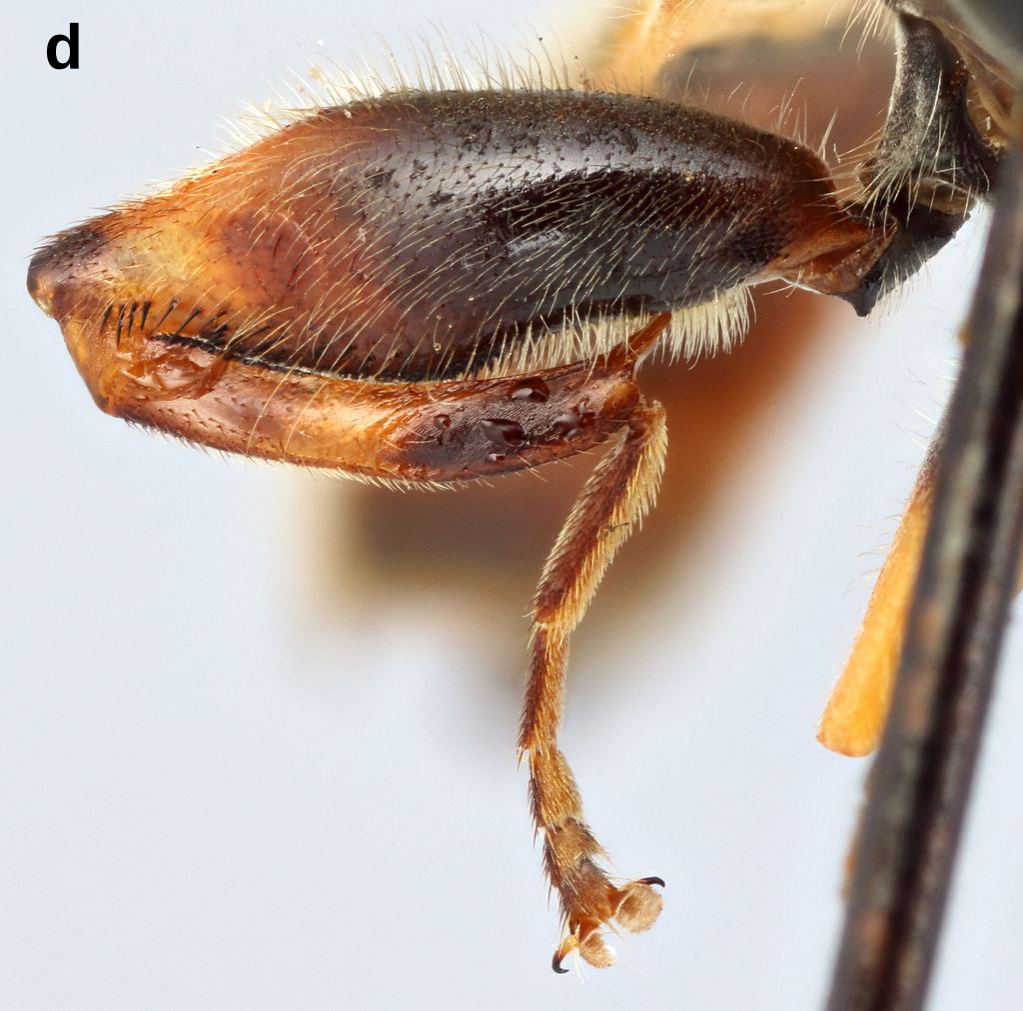
Hind leg, Jeff_Skevington_Specimen17432

**Figure 4a.**
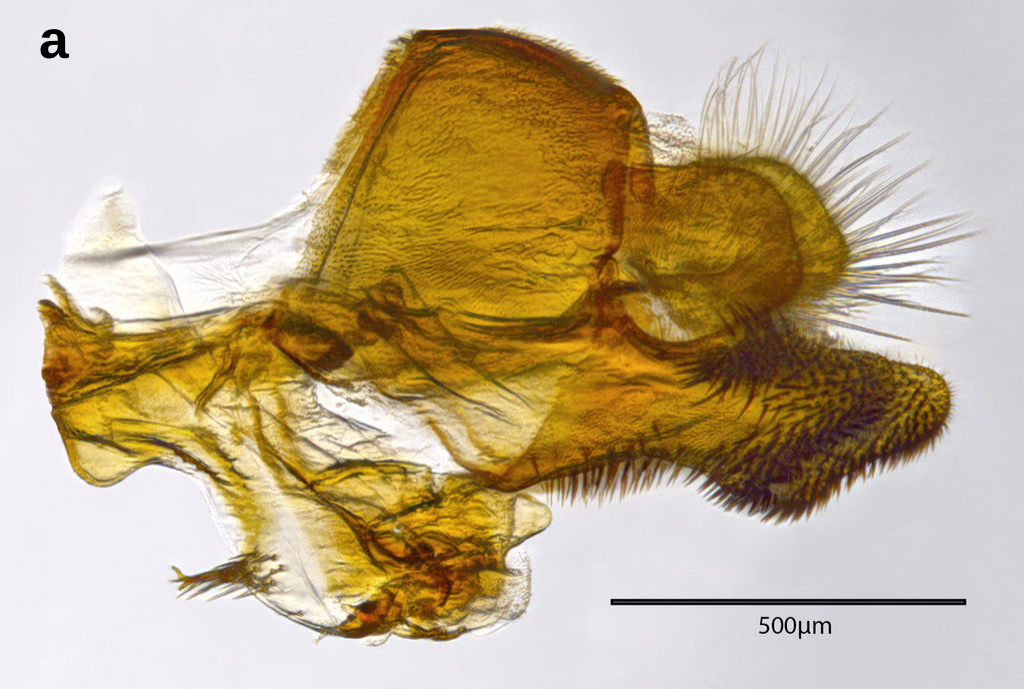
Lateral of male terminalia, Jeff_Skevington_Specimen17432; scale bar 500 µm

**Figure 4b.**
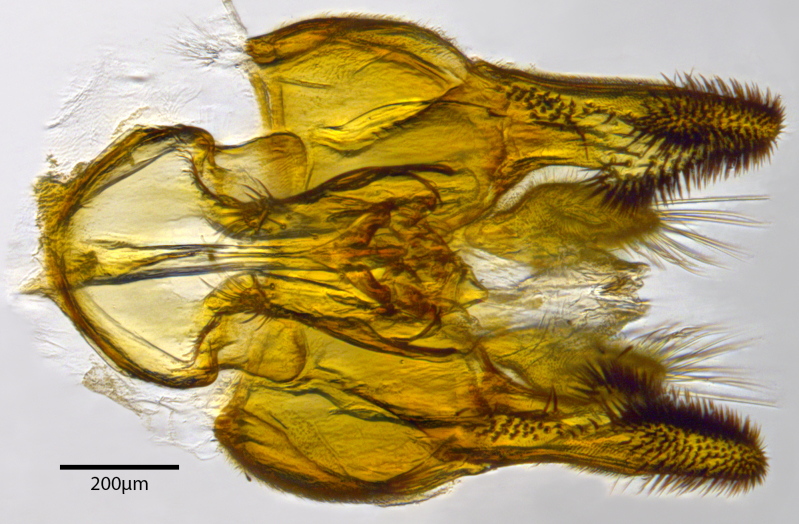
Ventral of male terminalia, Jeff_Skevington_Specimen17432; scale bar 200 µm

**Figure 4c.**
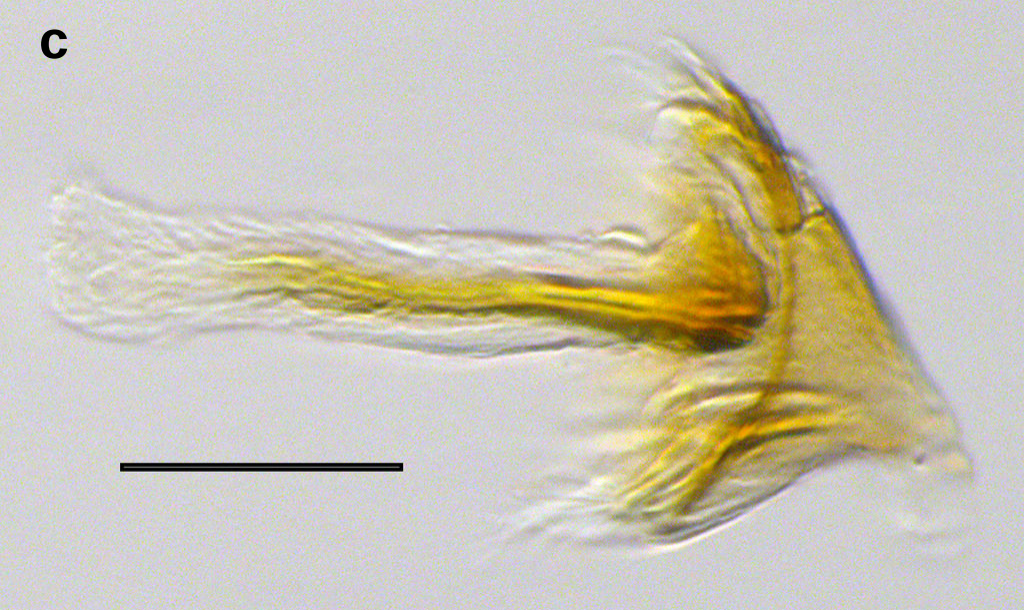
Sperm pump and ejaculatory apodeme, Jeff_Skevington_Specimen17432; scale bar 100 µm

**Figure 4d.**
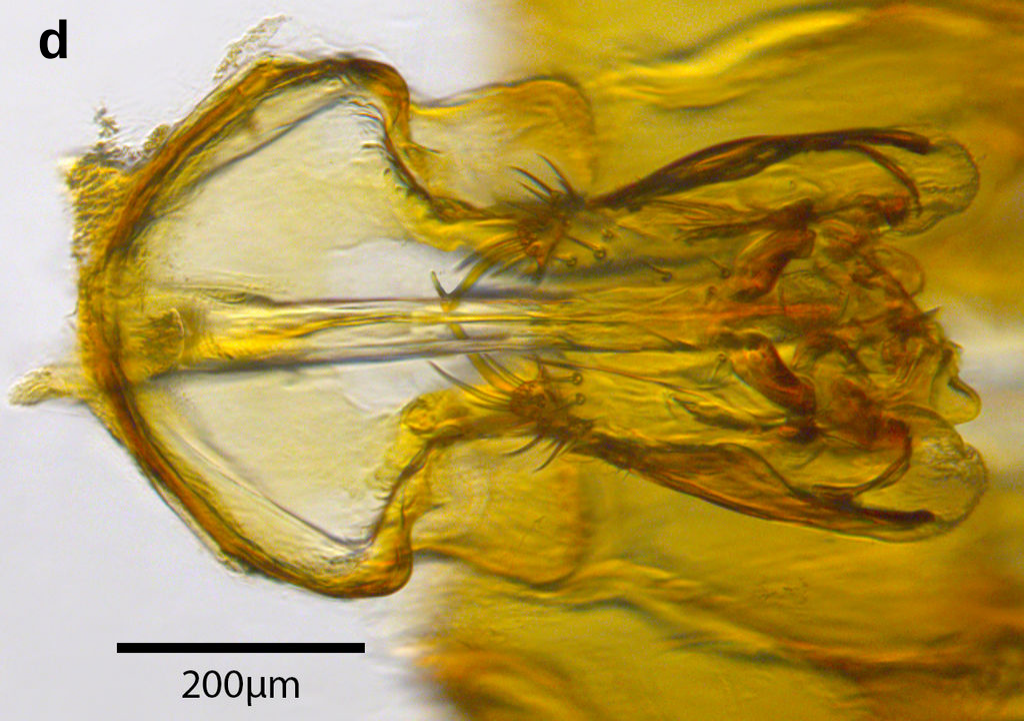
Ventral of hypandrium, Jeff_Skevington_Specimen17432; scale bar 200µm

**Figure 5a.**
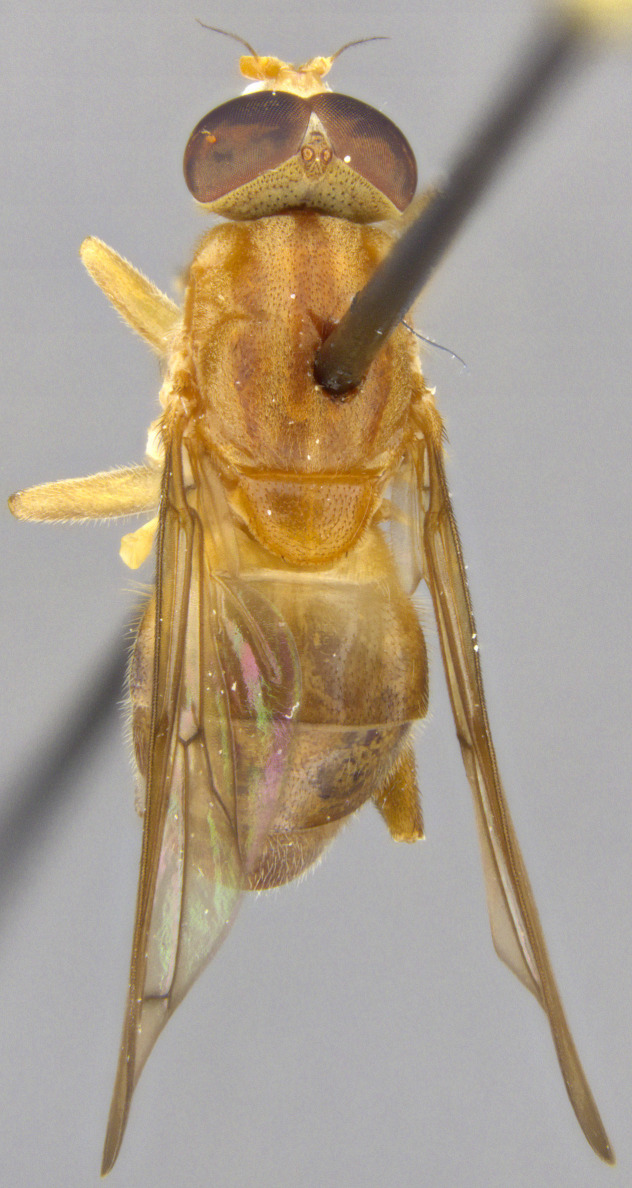
Dorsal habitus, CNC_Diptera243273

**Figure 5b.**
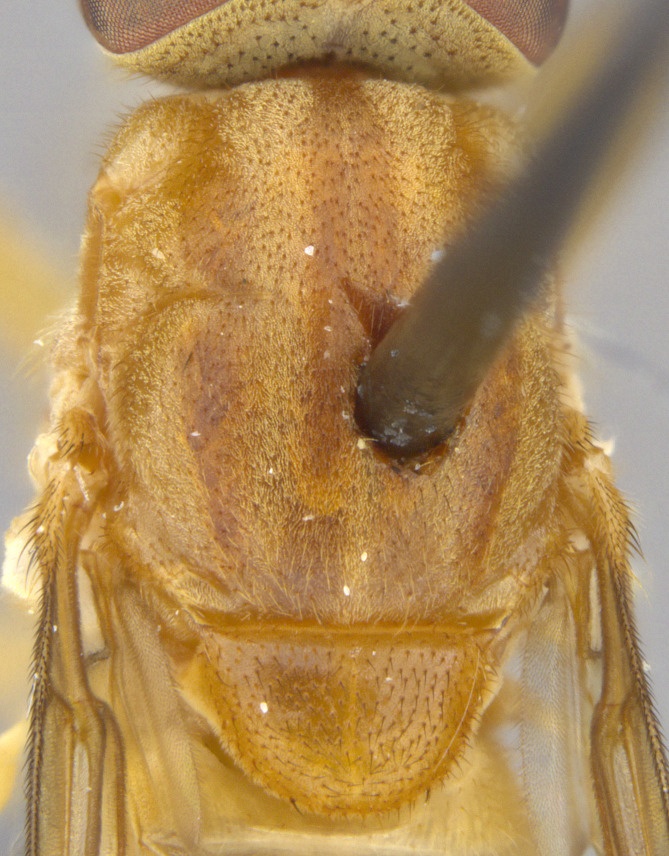
Dorsal scutellum, CNC_Diptera243273

**Figure 5c.**
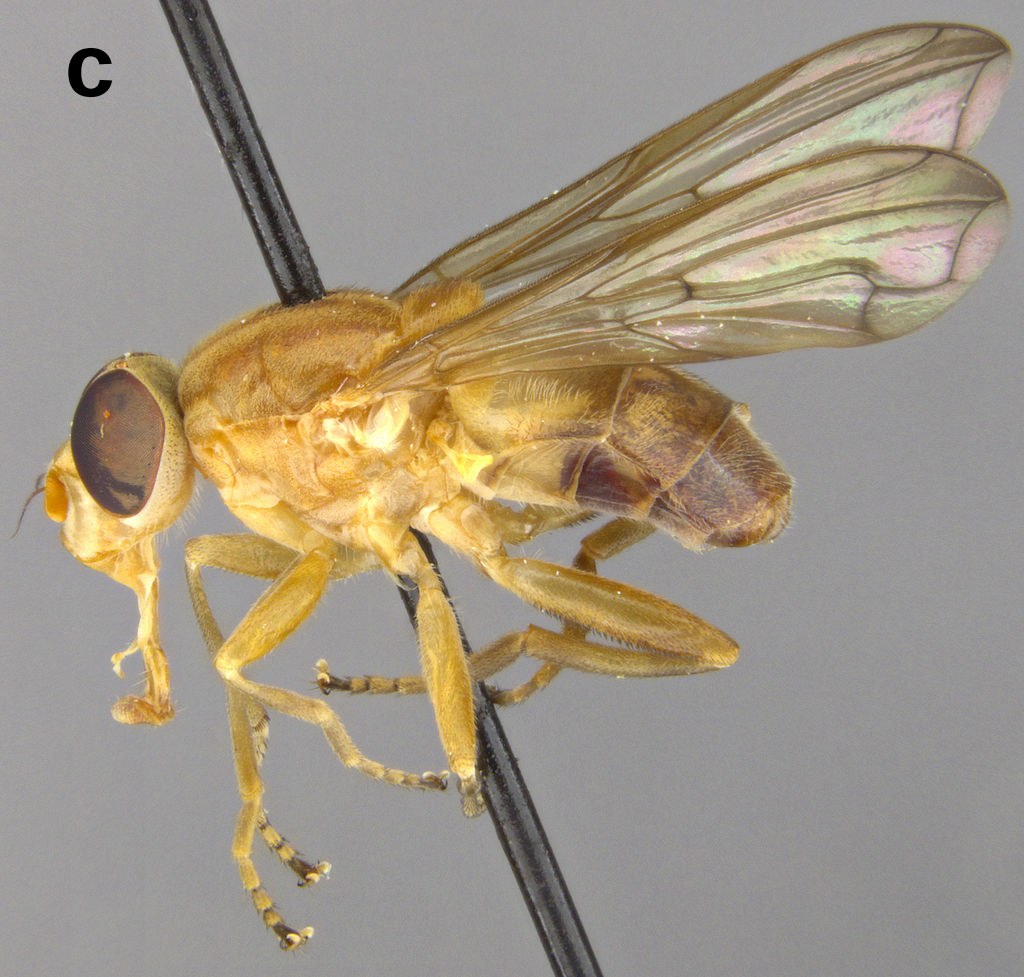
Lateral habitus, CNC_Diptera243273

**Figure 5d.**
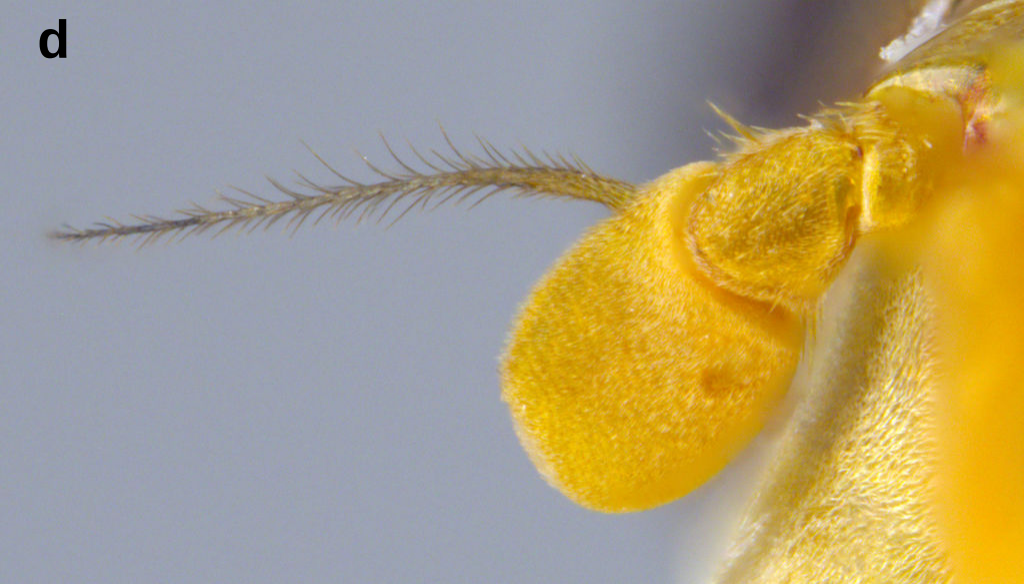
Arista, CNC_Diptera243273

**Figure 5e.**
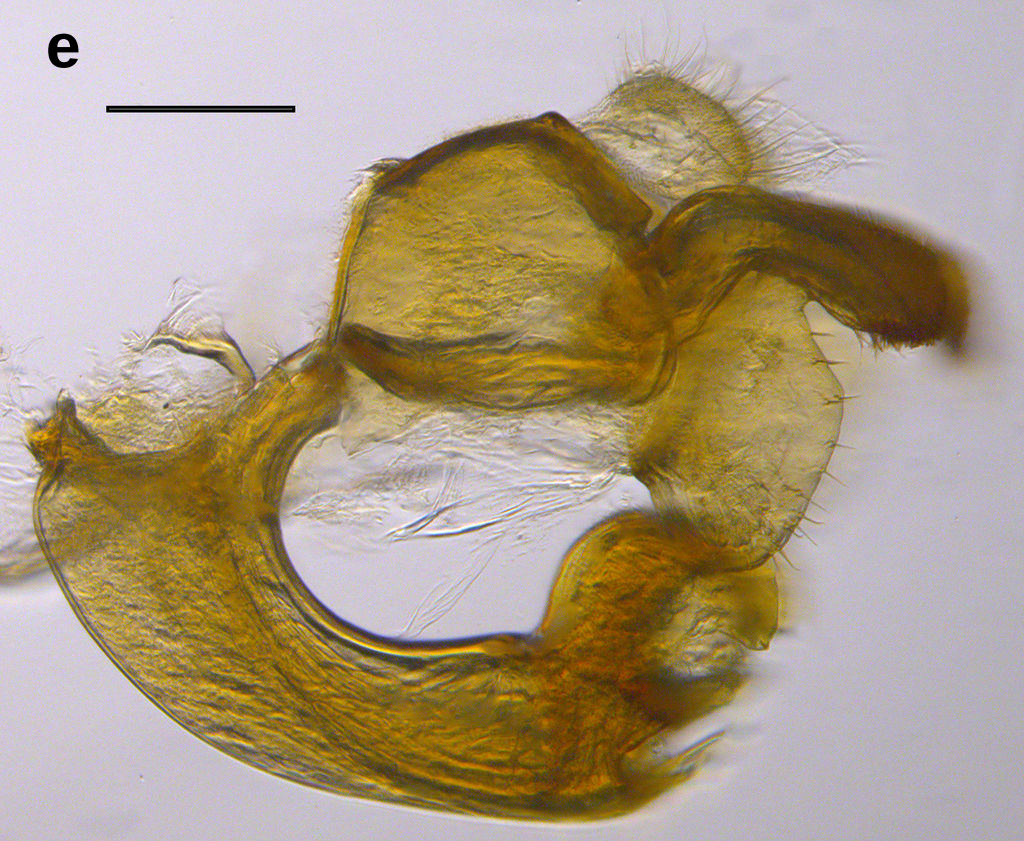
Lateral male genitalia, CNC_Diptera37549; scale bar 200 µm

**Figure 5f.**
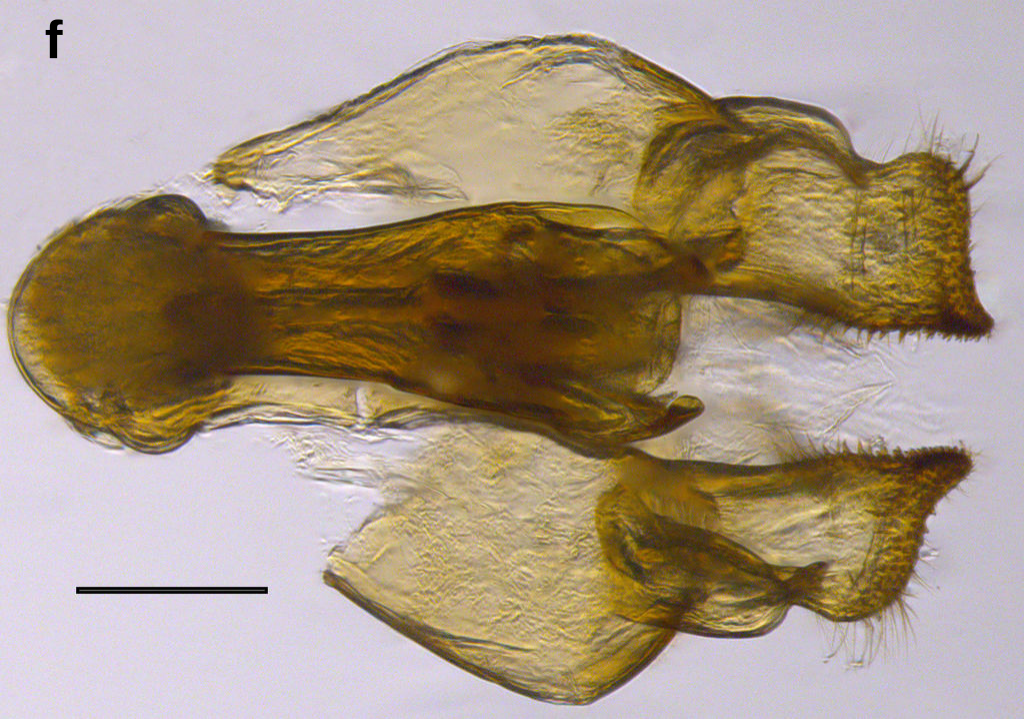
Ventral male genitalia, CNC_Diptera37549; scale bar 200 µm

**Figure 6a.**
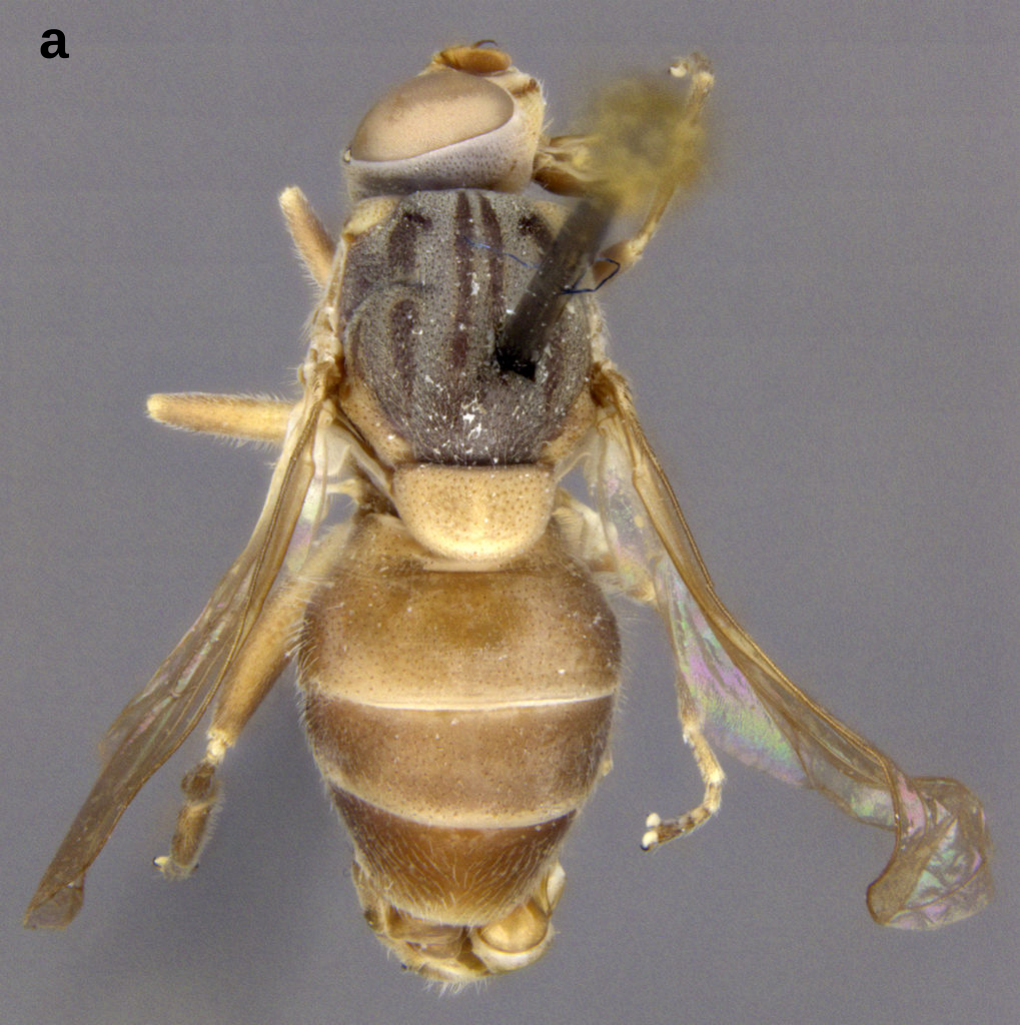
Dorsal habitus, Holotype, CNC_Diptera106864

**Figure 6b.**
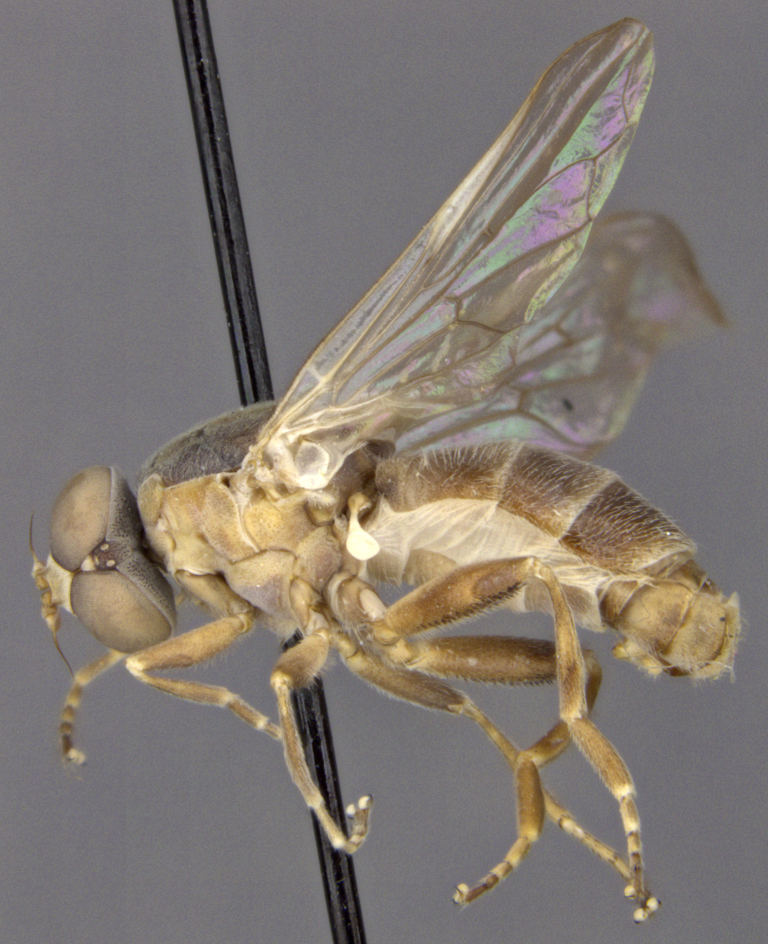
Lateral habitus, Holotype, CNC_Diptera106864

**Figure 6c.**
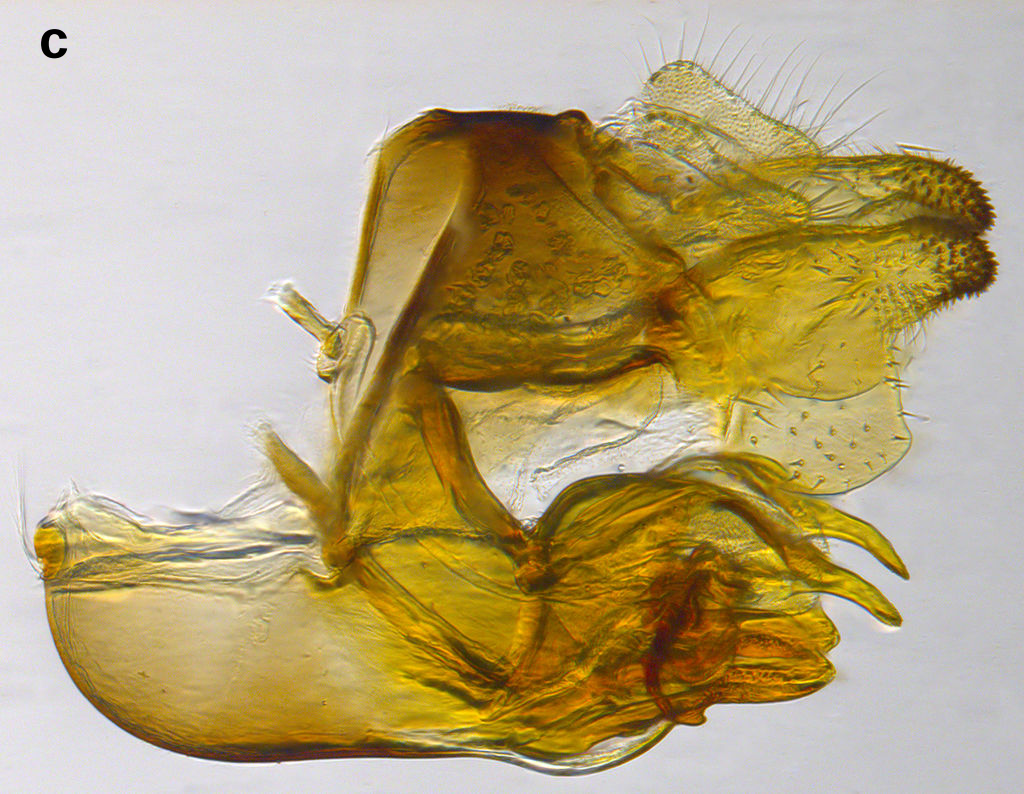
Lateral, male genitalia, Holotype, CNC_Diptera106864

**Figure 6d.**
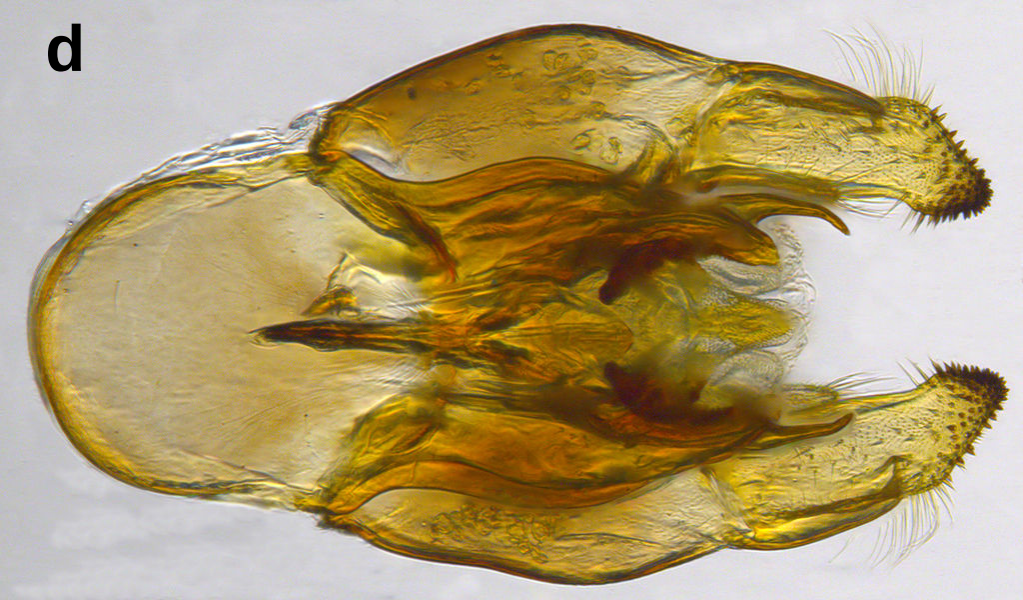
Ventral, male genitalia, Holotype, CNC_Diptera106864

**Figure 6e.**
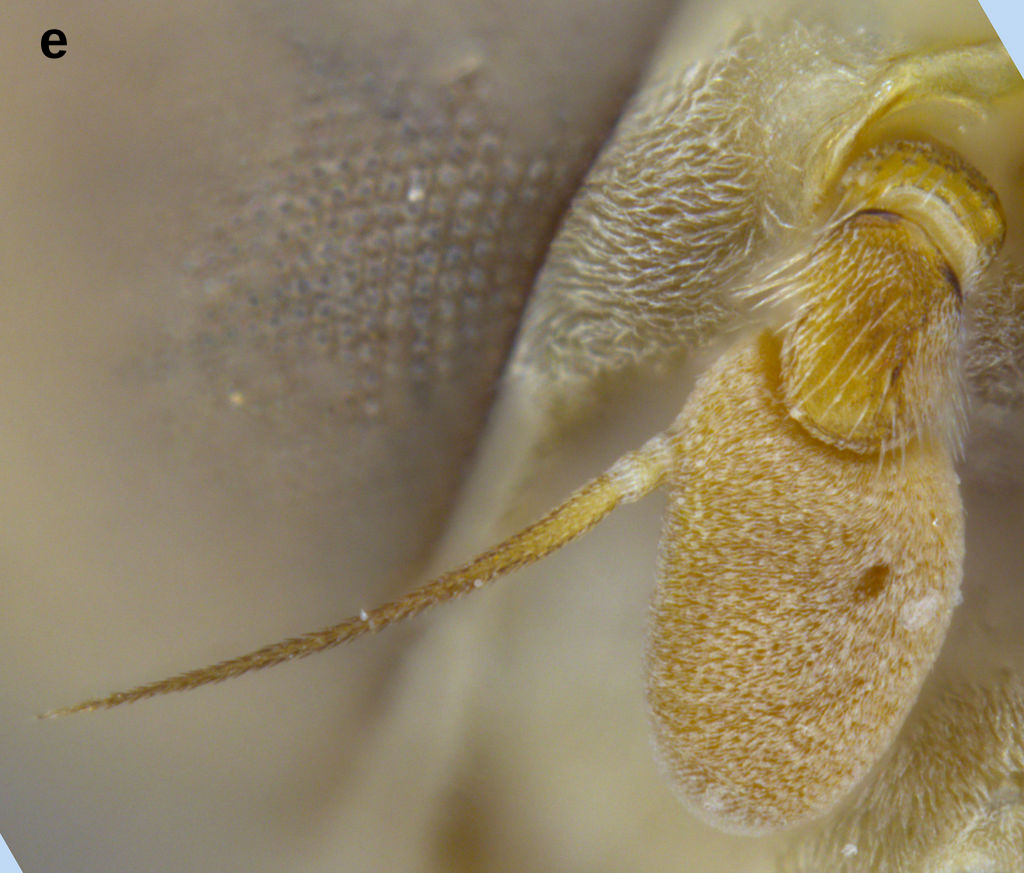
Antenna, Holotype, CNC_Diptera106864

**Figure 7a.**
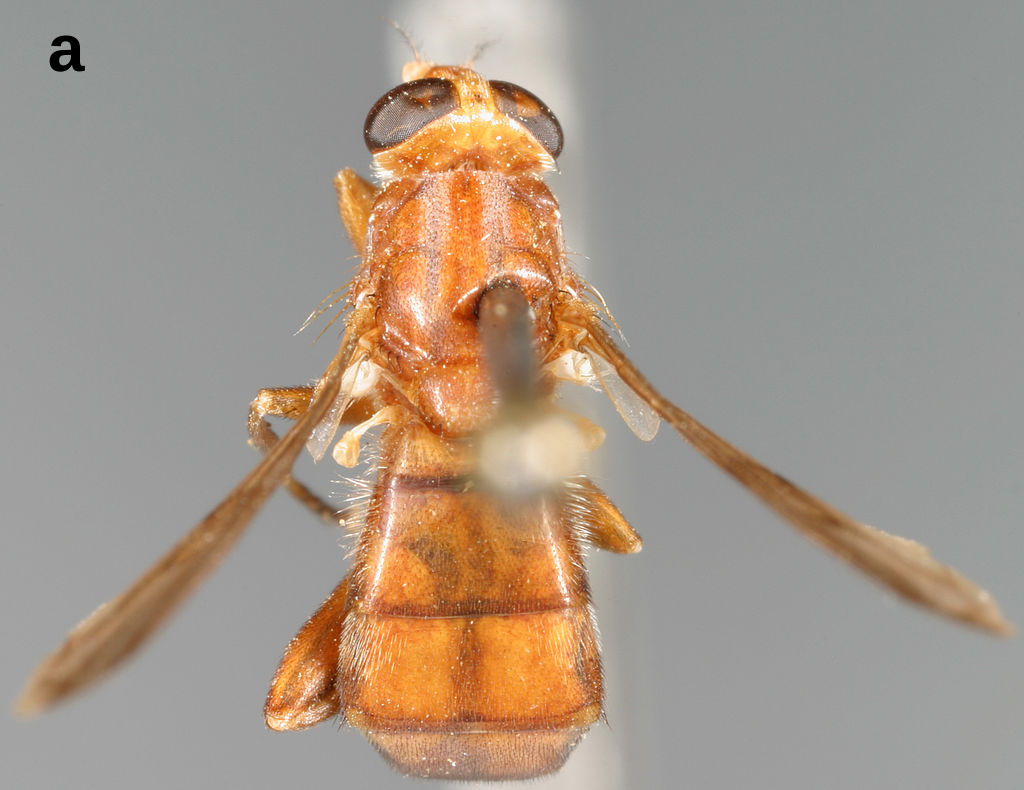
Dorsal habitus, CNC_Diptera556

**Figure 7b.**
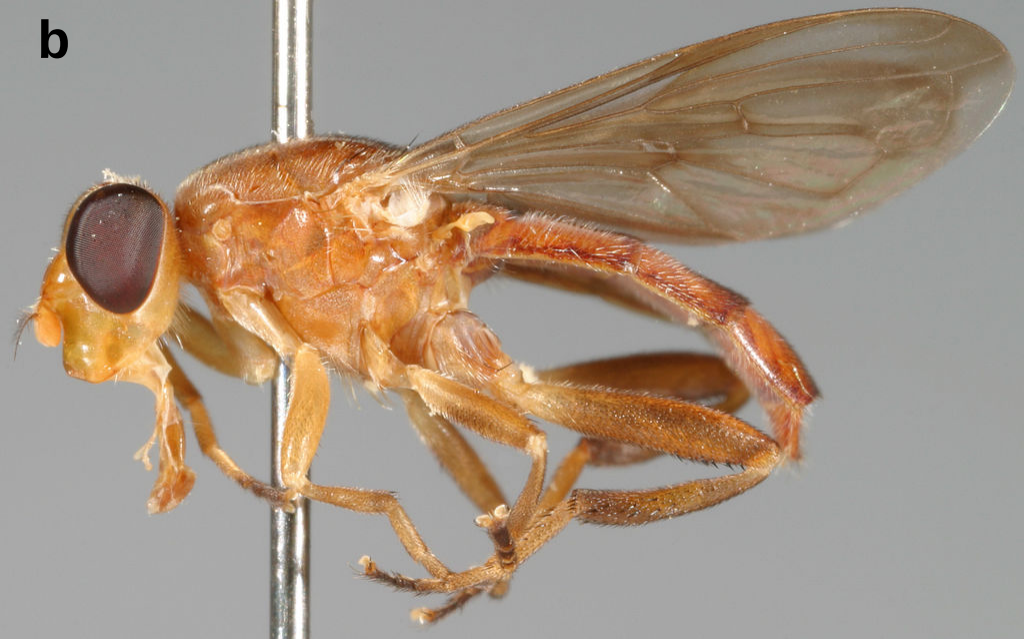
Lateral habitus, CNC_Diptera574

**Figure 7c.**
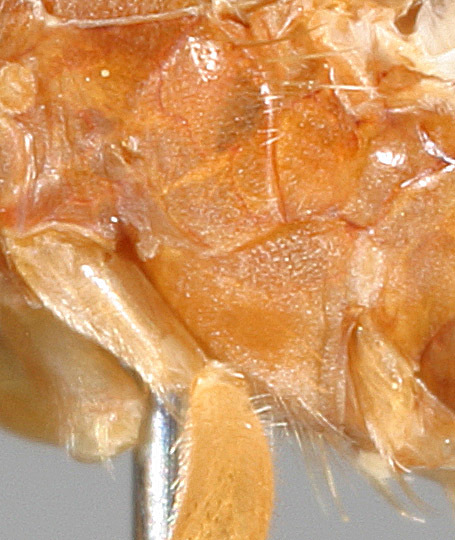
Katepisternum, CNC_Diptera574

**Figure 7d.**
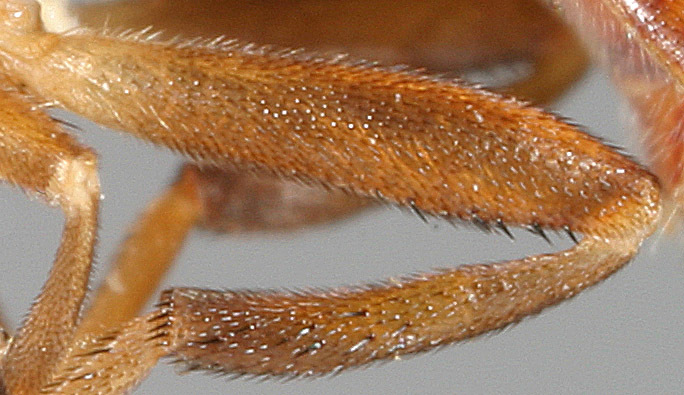
Hind leg, CNC_Diptera574

**Figure 7e.**
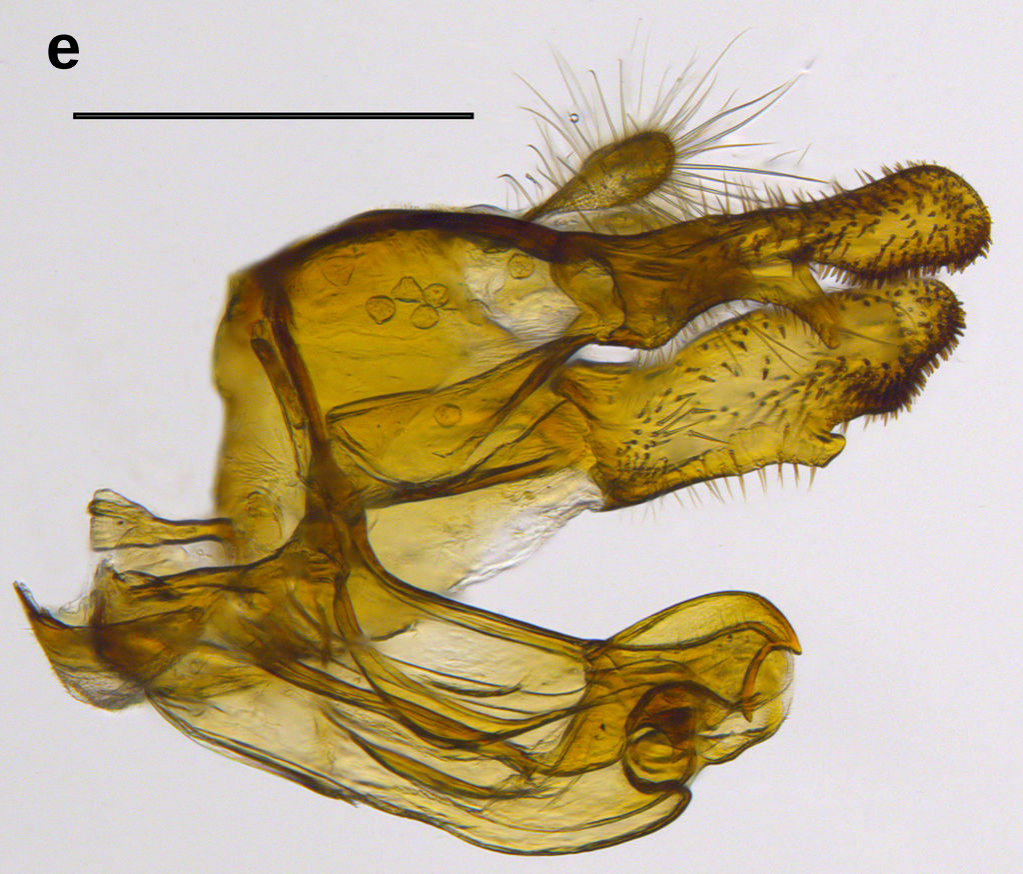
Lateral of male genitalia, CNC_Diptera49259; scale bar 500 µm

**Figure 7f.**
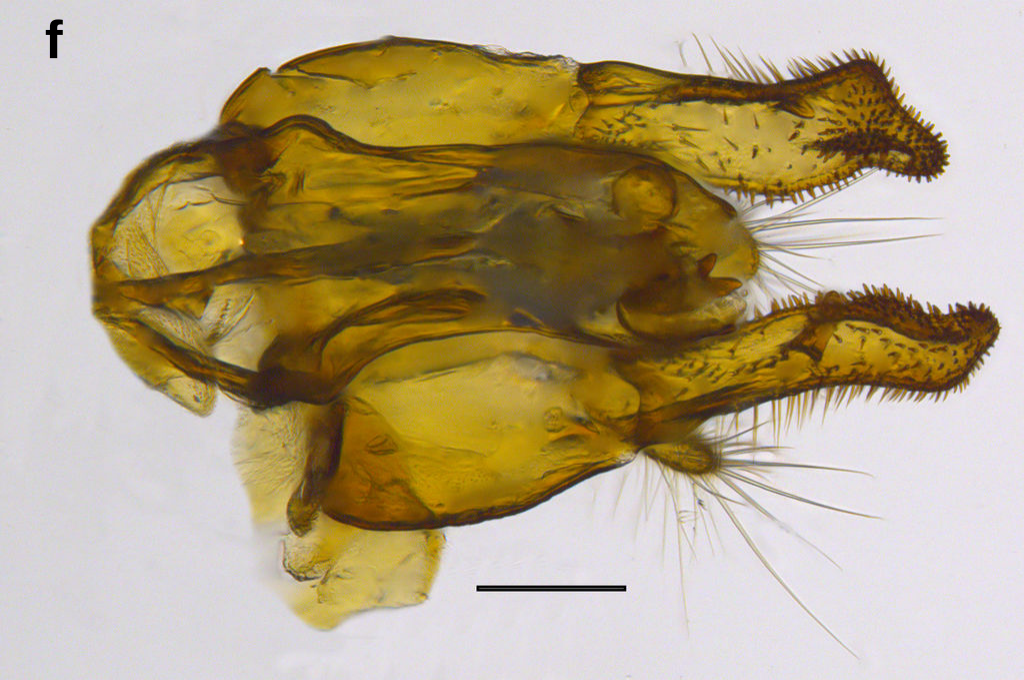
Ventral of male genitalia, CNC_Diptera49259; scale bar 200 µm

**Figure 8a.**
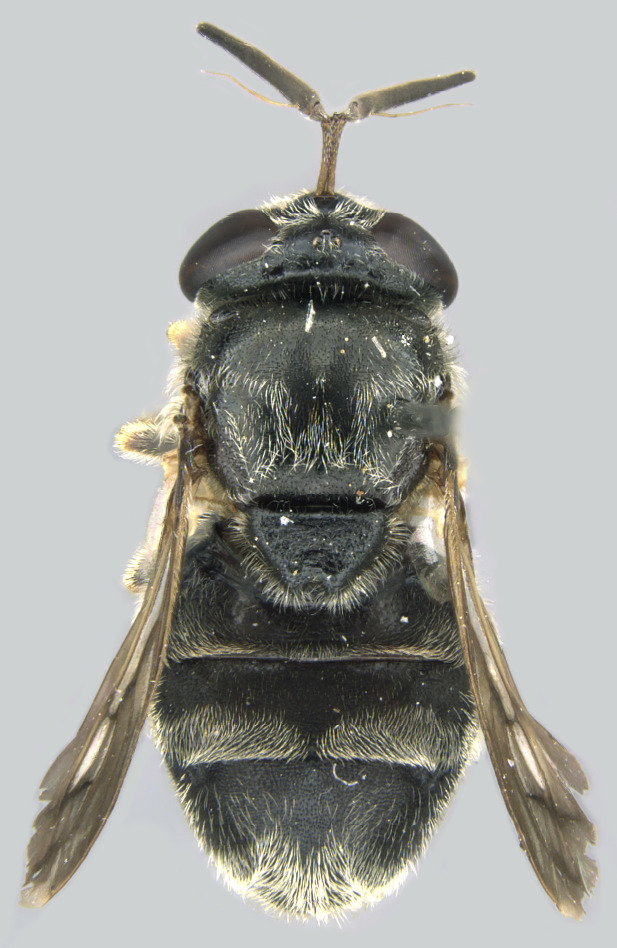
Dorsal habitus, Holotype, Jeff_Skevington_Specimen44169

**Figure 8b.**
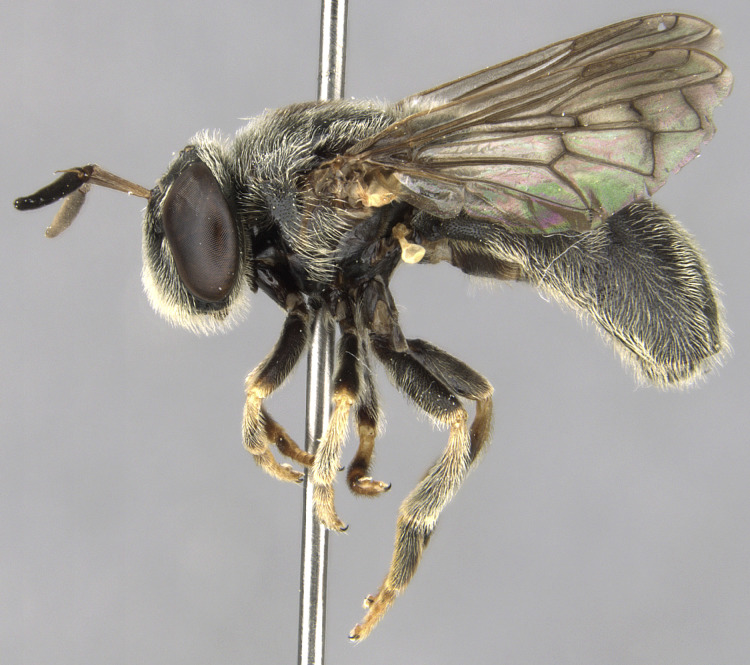
Lateral habitus, Holotype, Jeff_Skevington_Specimen44169

**Figure 8c.**
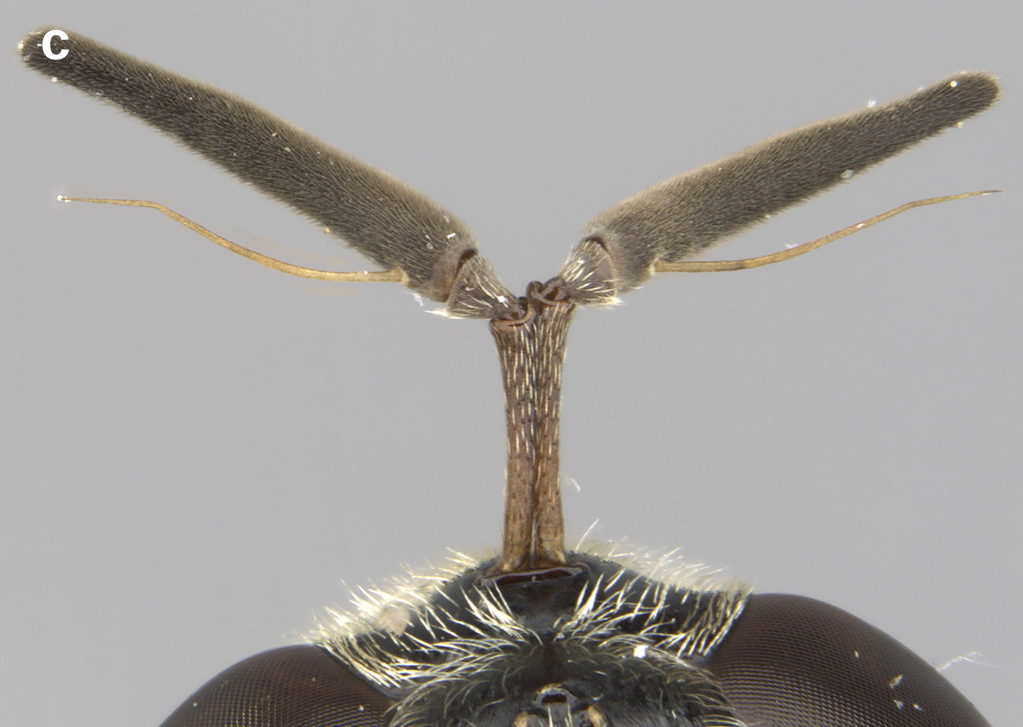
Antennae, Holotype, Jeff_Skevington_Specimen44169

**Figure 8d.**
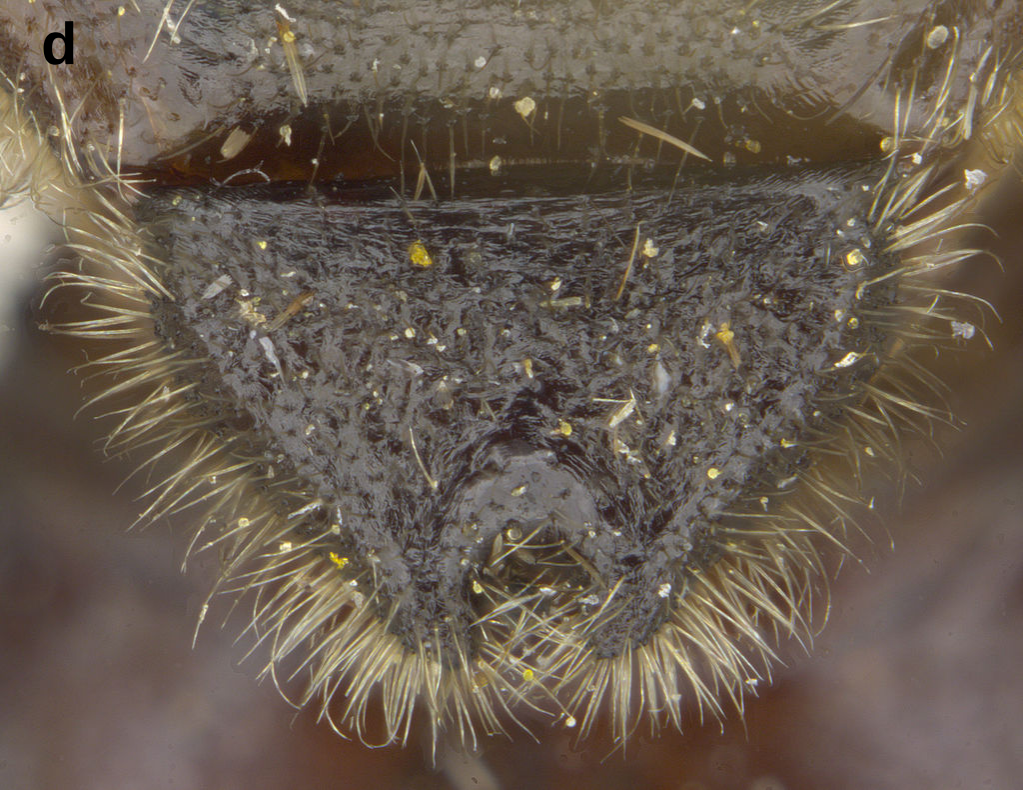
Scutellum, Jeff_Skevington_Specimen44200

**Figure 8e.**
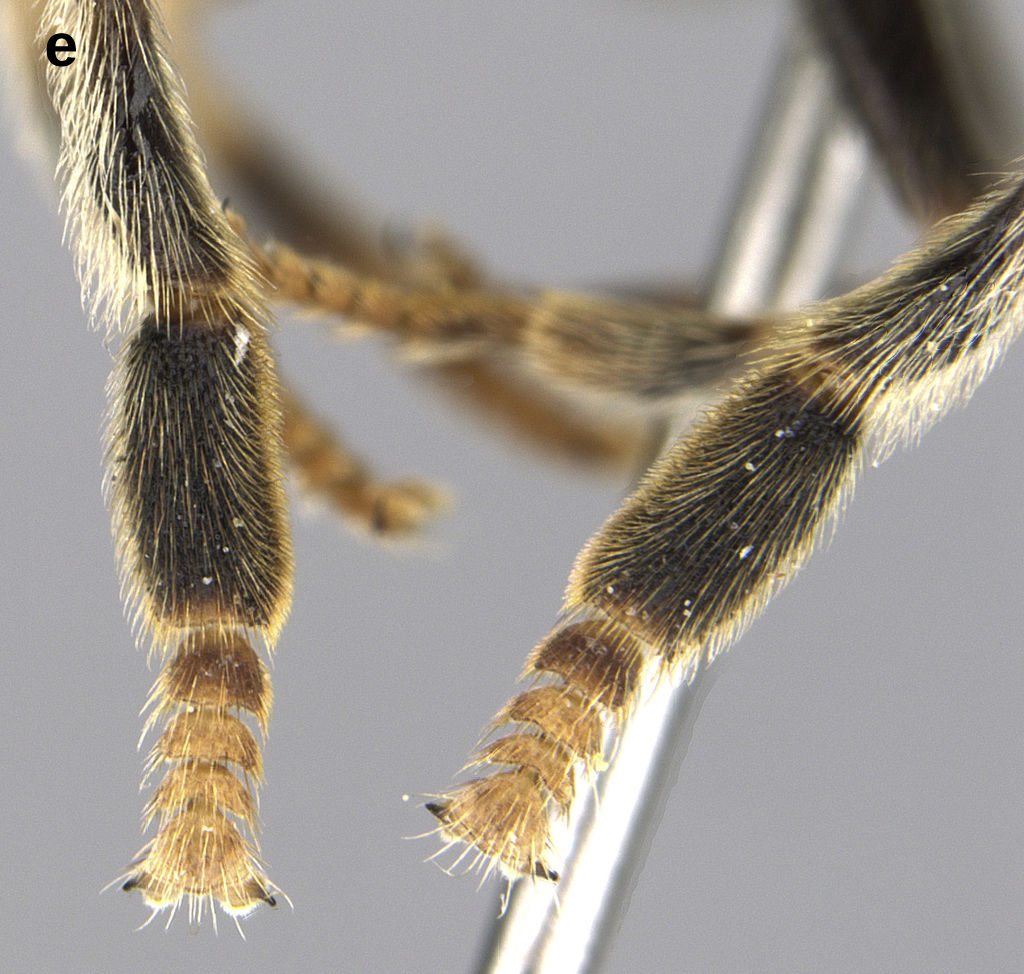
Male hind tarsi, Holotype, Jeff_Skevington_Specimen44169

**Figure 8f.**
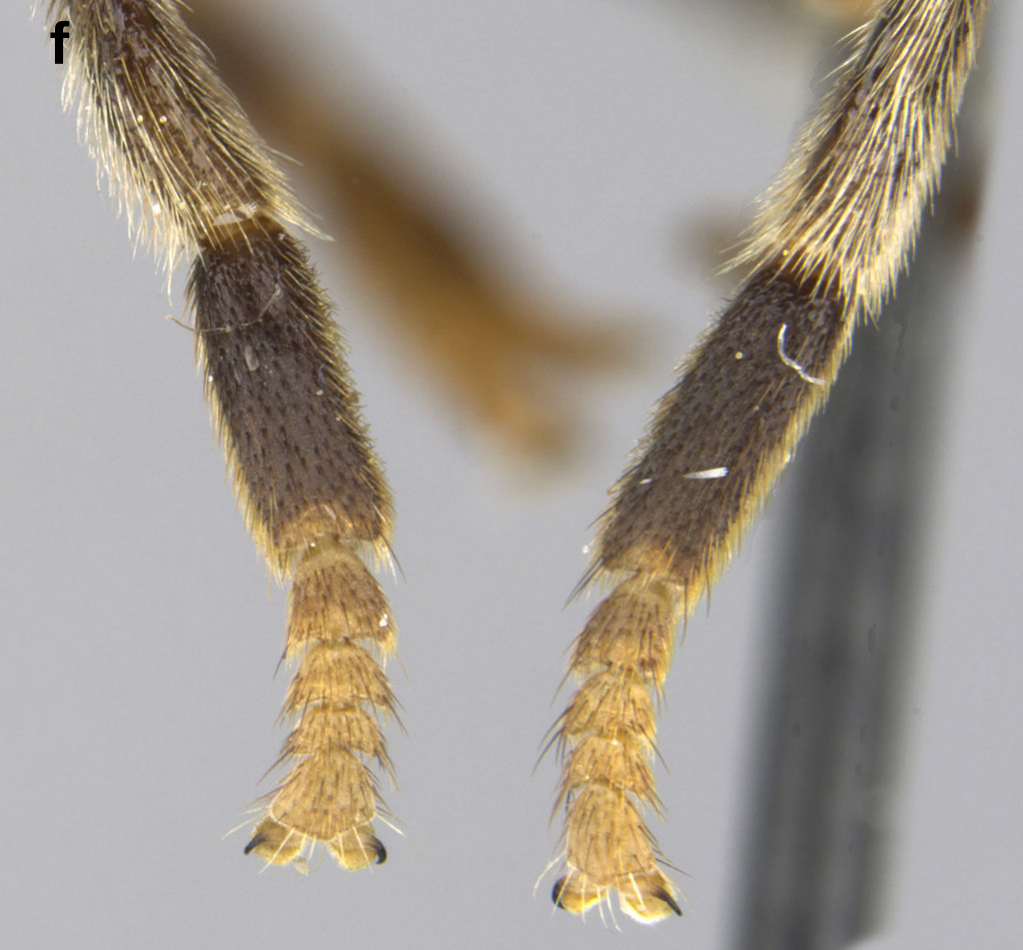
Female hind tarsi, Jeff_Skevington_Specimen44200

**Figure 9a.**
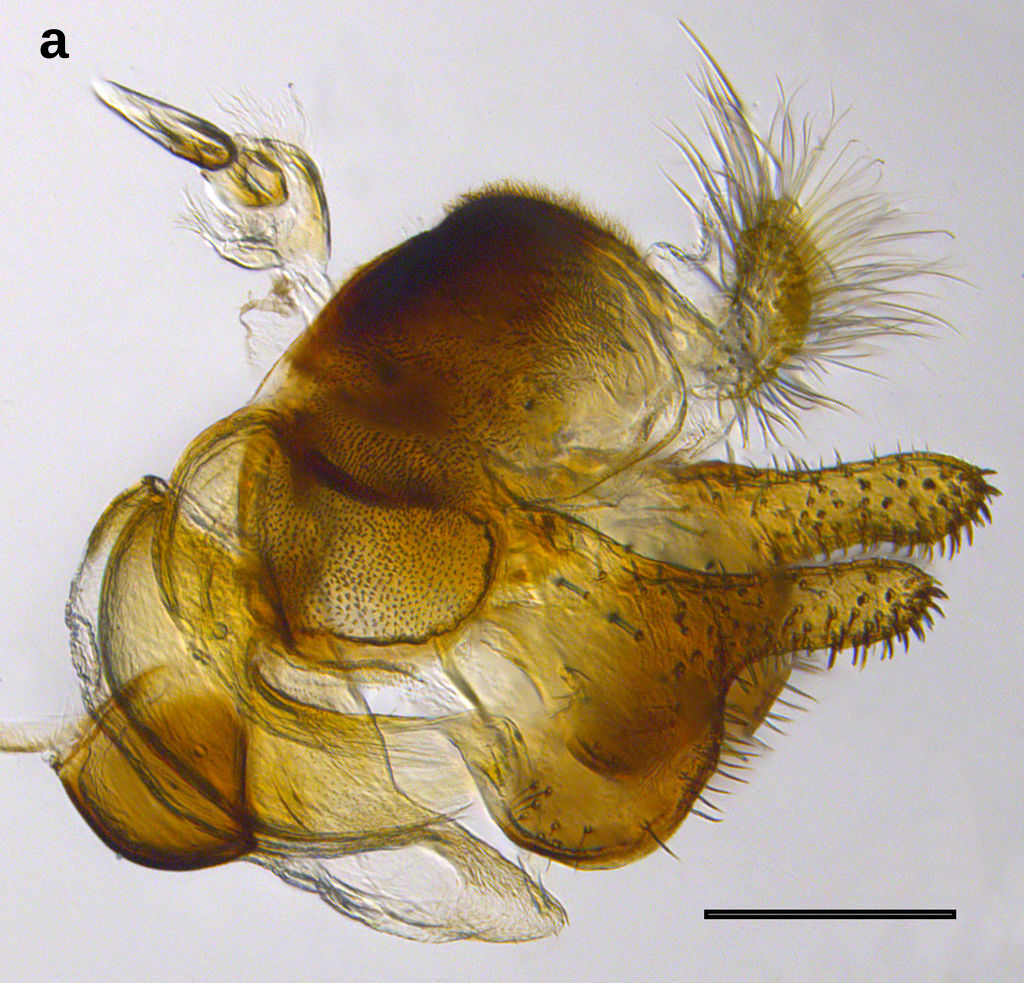
Lateral of male genitalia, Jeff_Skevington_Specimen44181; scale bar 500 µm

**Figure 9b.**
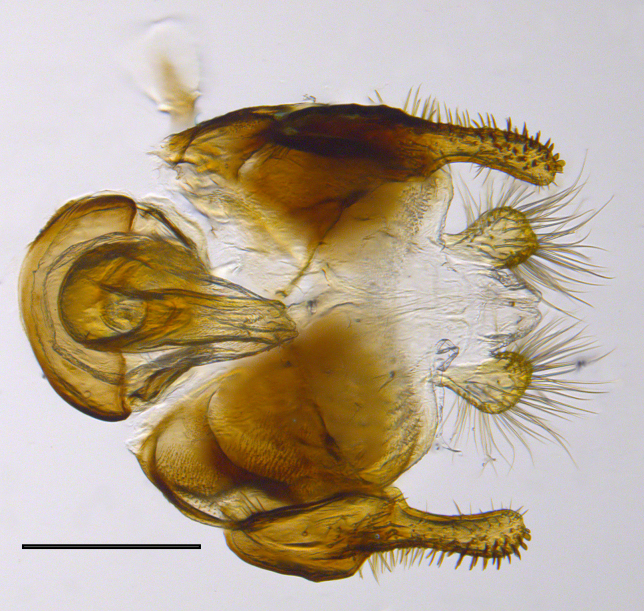
Ventral of male genitalia, Jeff_Skevington_Specimen44181; scale bar 500 µm

**Figure 9c.**
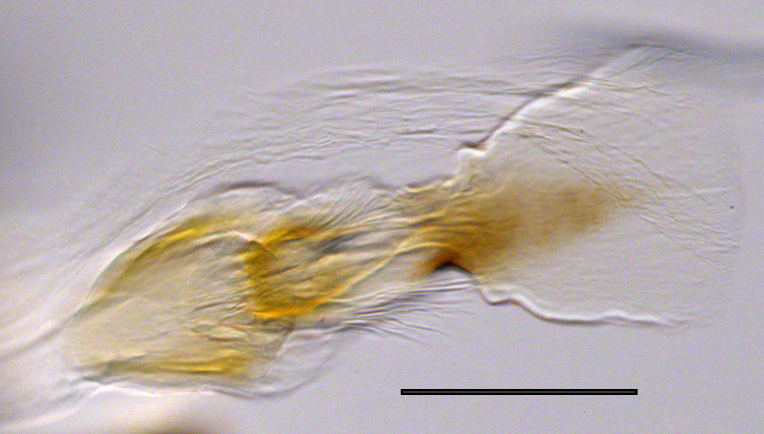
Lateral of ejaculatory apodeme and sperm pump, Jeff_Skevington_Specimen44181; scale bar 200 µm

**Figure 9d.**
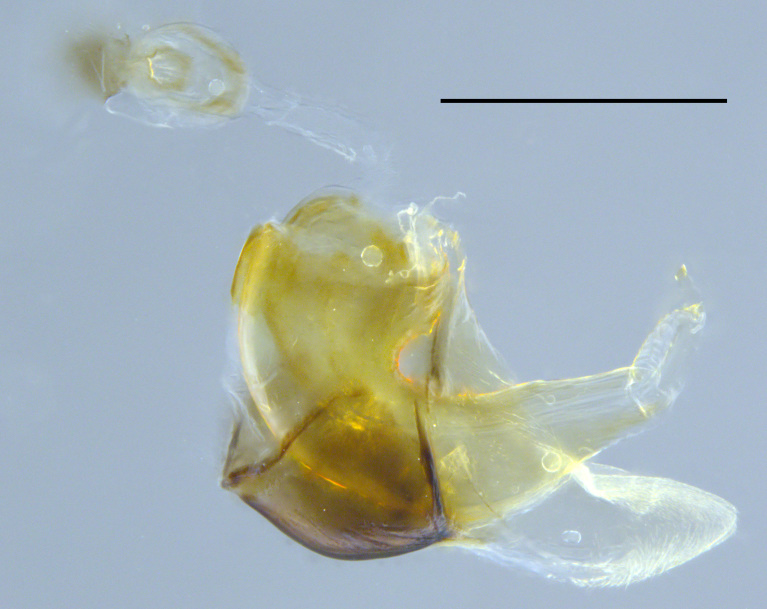
Lateral of phallus, hypandrium and related structures, Jeff_Skevington_Specimen44183, scale bar 200 µm

**Figure 10a.**
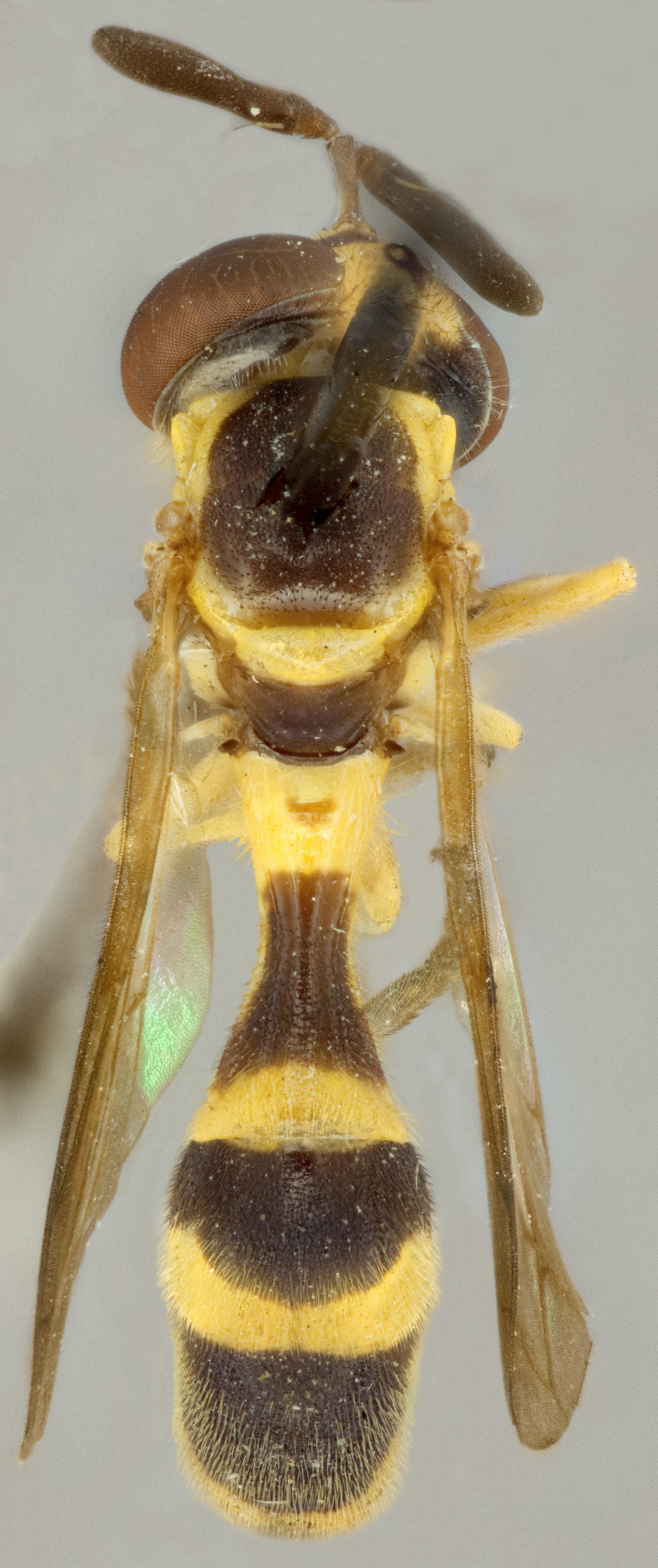
Dorsal habitus, CNC_Diptera92831

**Figure 10b.**
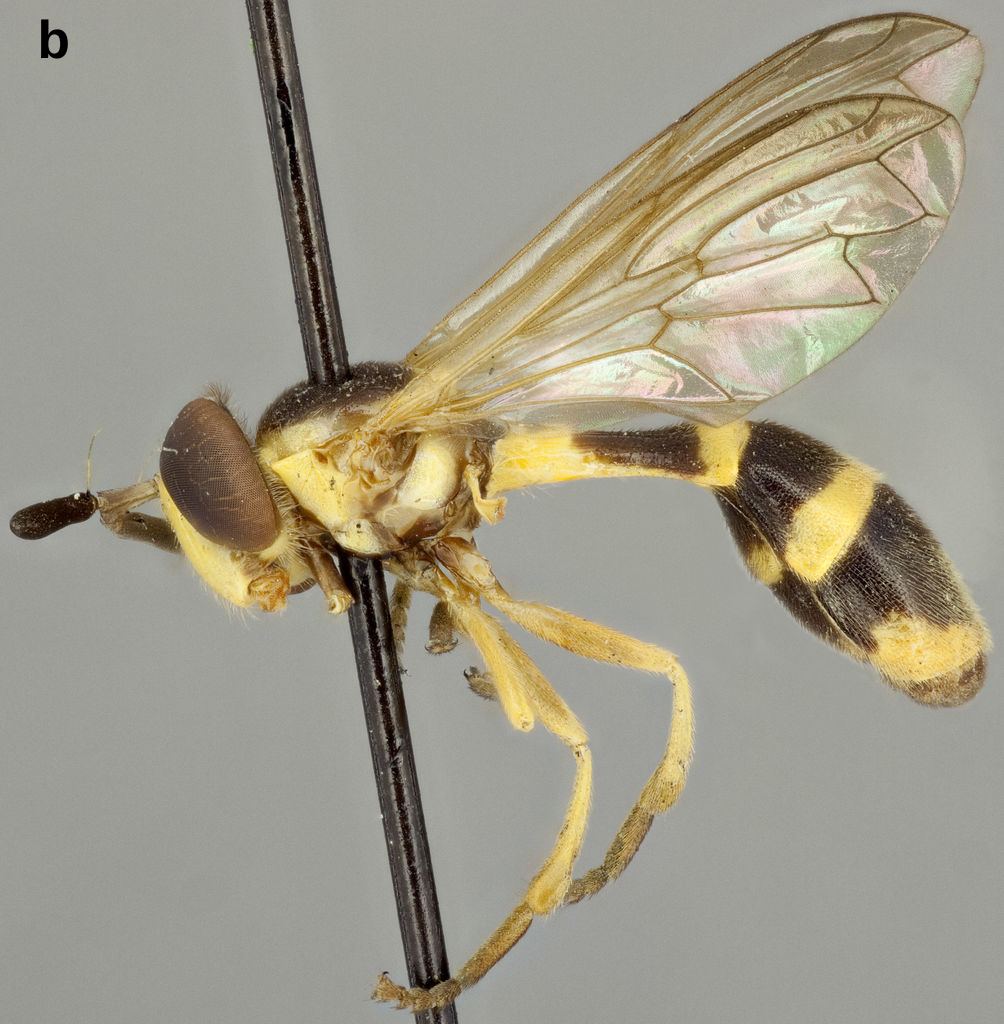
Lateral habitus, CNC_Diptera92831

**Figure 10c.**
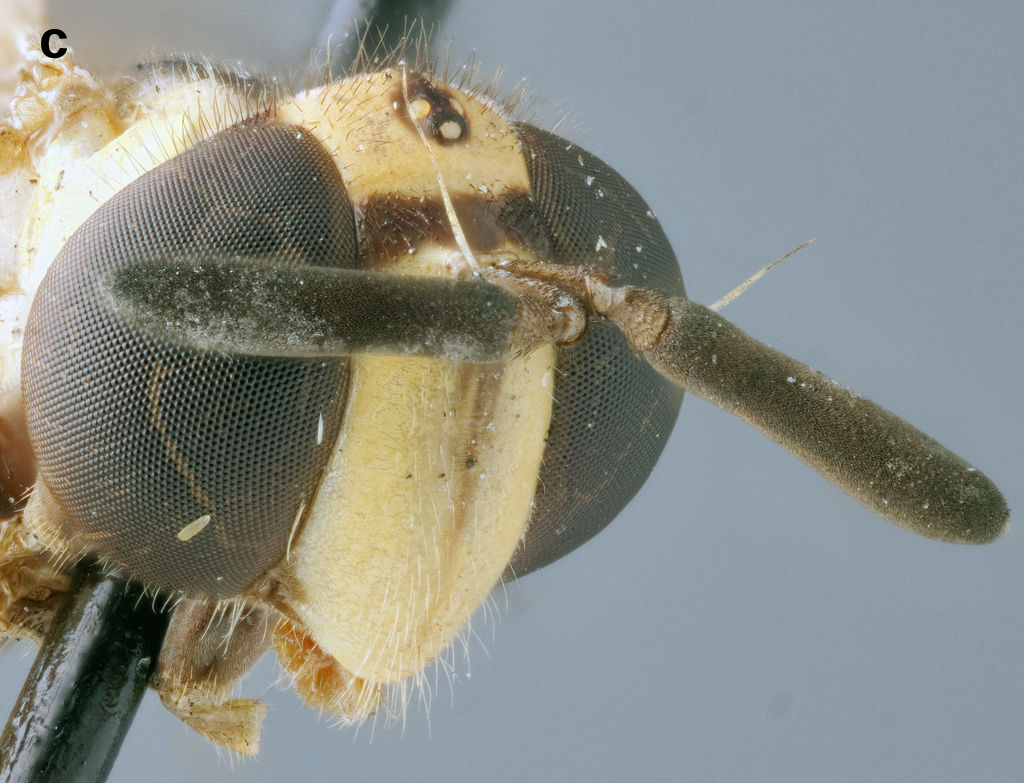
Head, oblique, CNC_Diptera92831

**Figure 10d.**
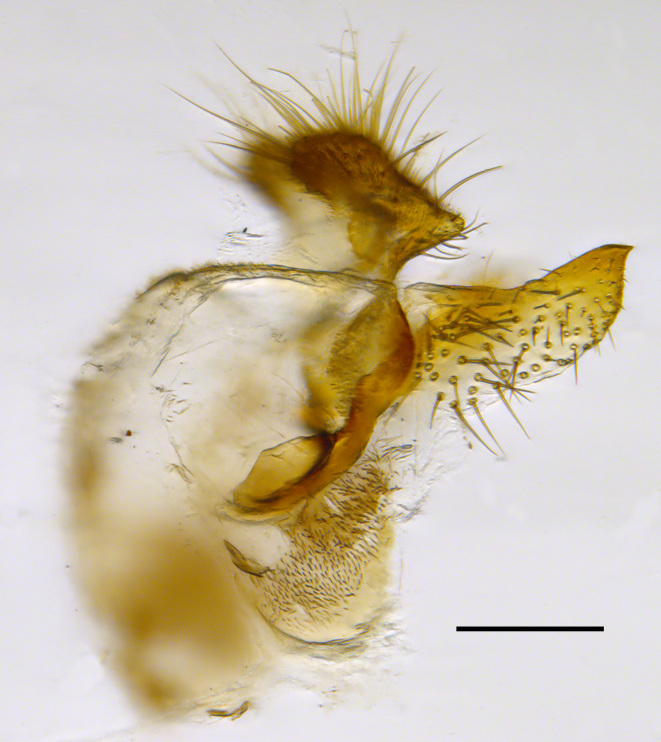
Male epandrium, cercus and surstylus, lateral, CNC1129651; scale bar 200 µm

**Figure 10e.**
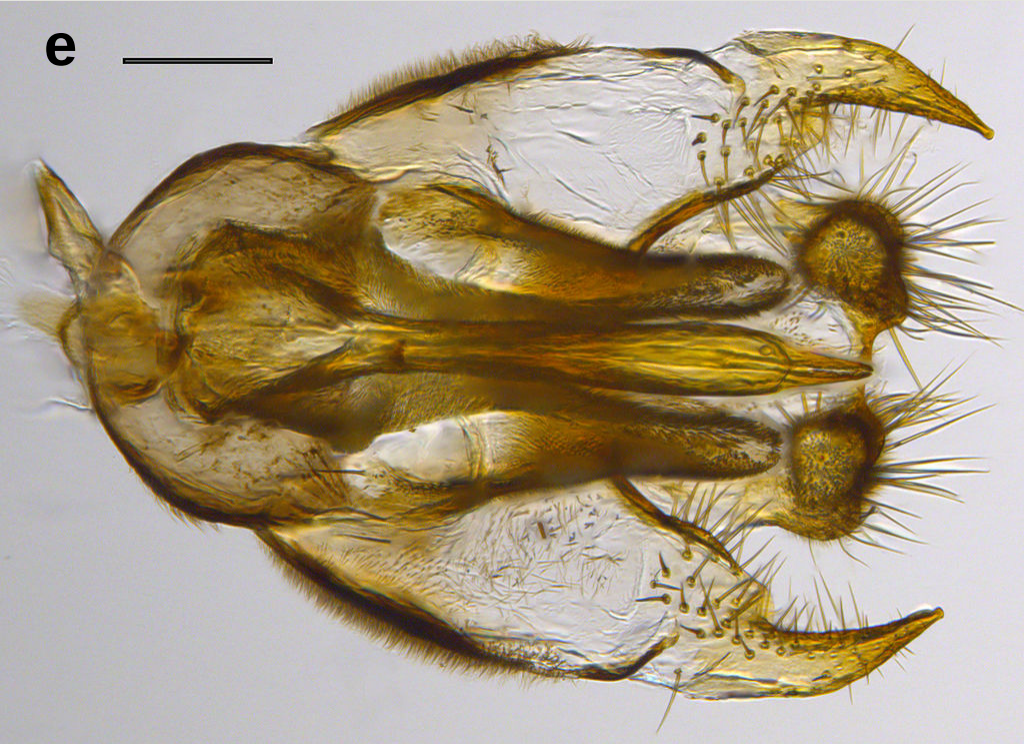
Male genitalia, ventral, CNC1129651; scale bar 200 µm

**Figure 10f.**
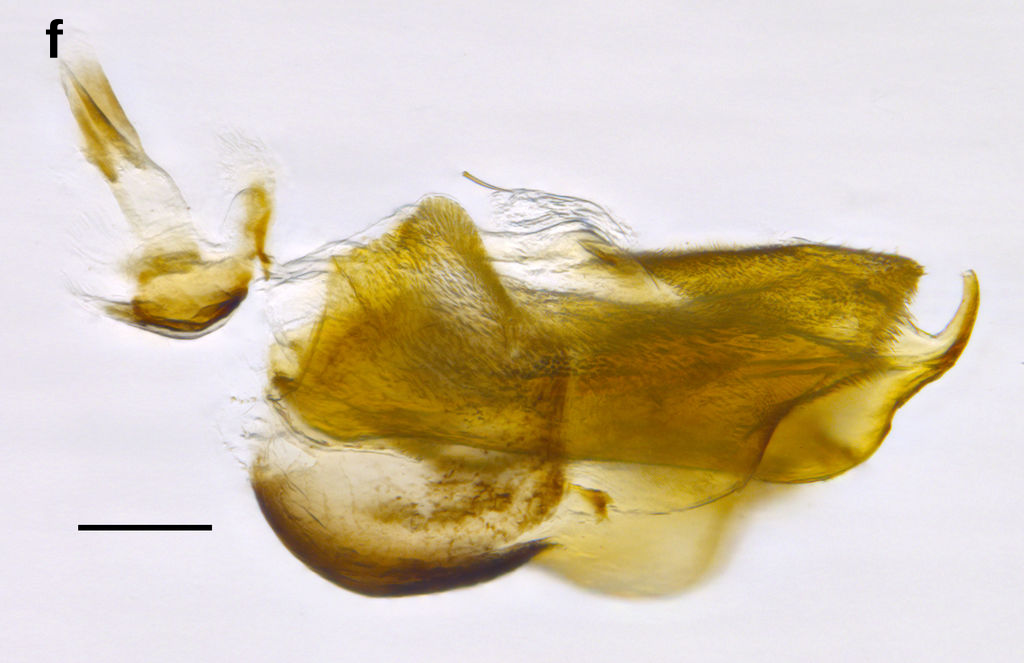
Male hypandrium and phallus, lateral, CNC1129651; scale bar 200 µm

**Figure 11a.**
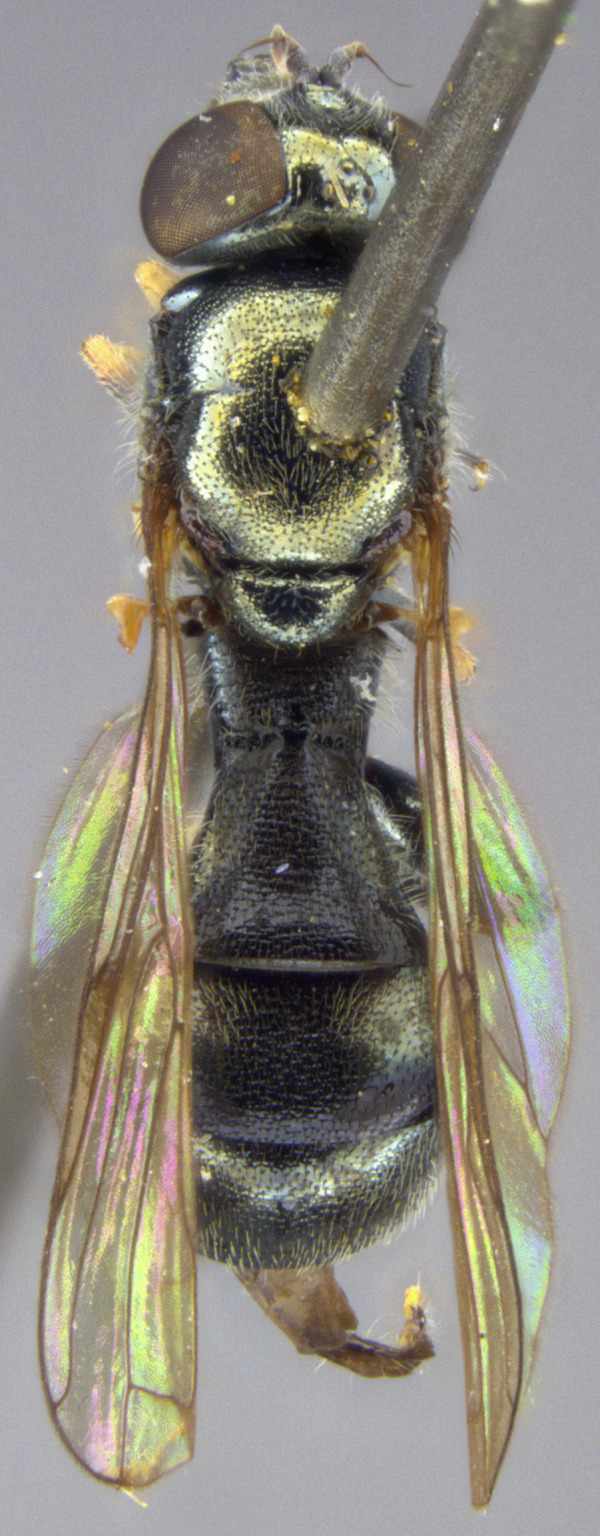
Dorsal habitus, female, CNC_Diptera169737

**Figure 11b.**
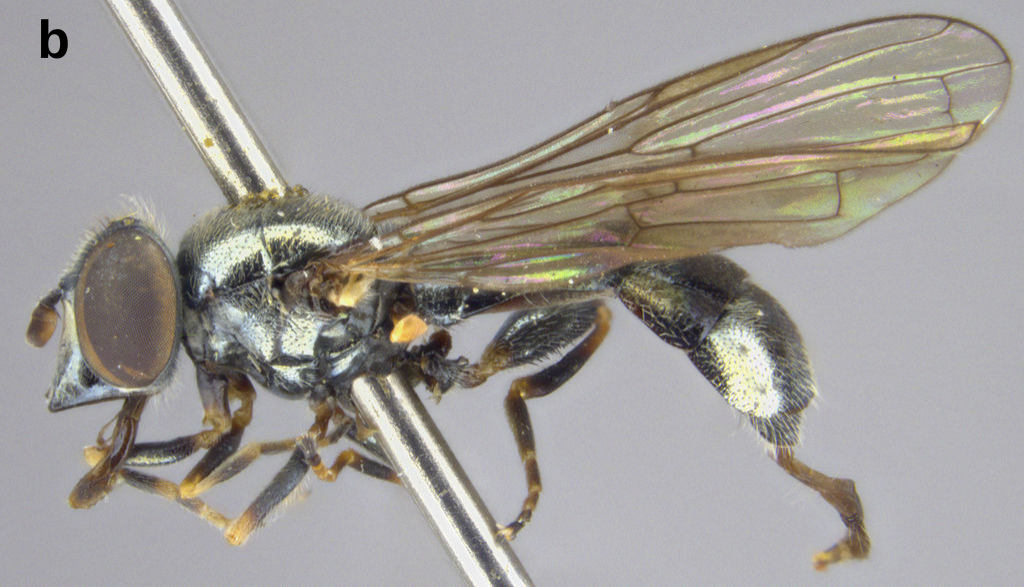
Lateral habitus, female, CNC_Diptera169737

**Figure 11c.**
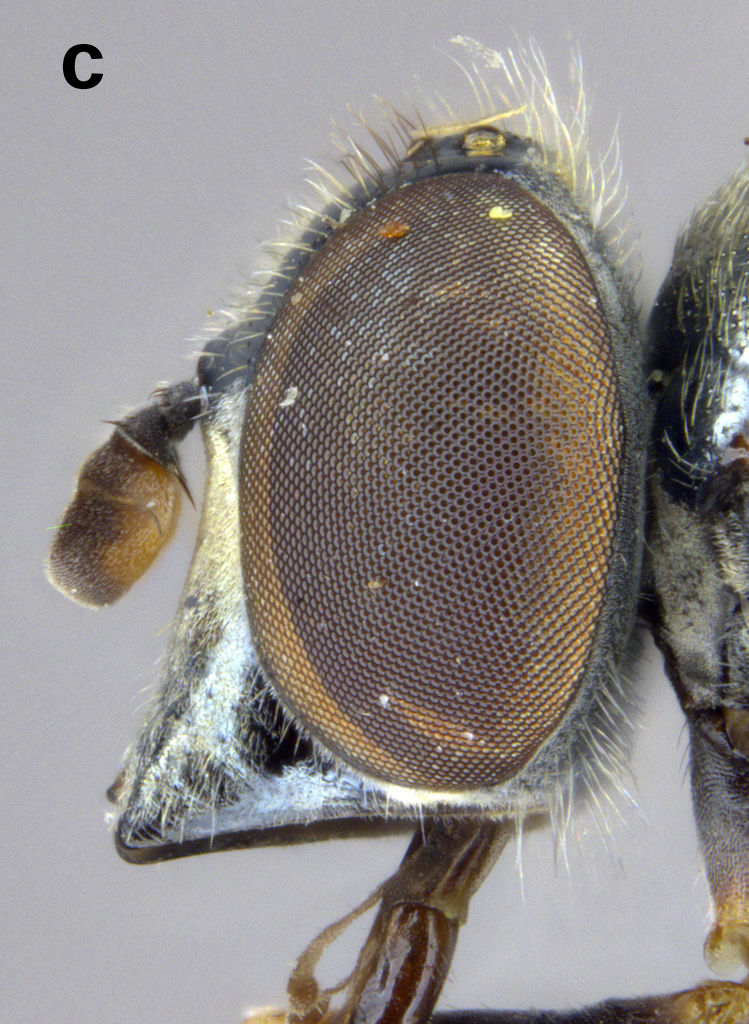
Lateral head, female, CNC_Diptera169737

**Figure 11d.**
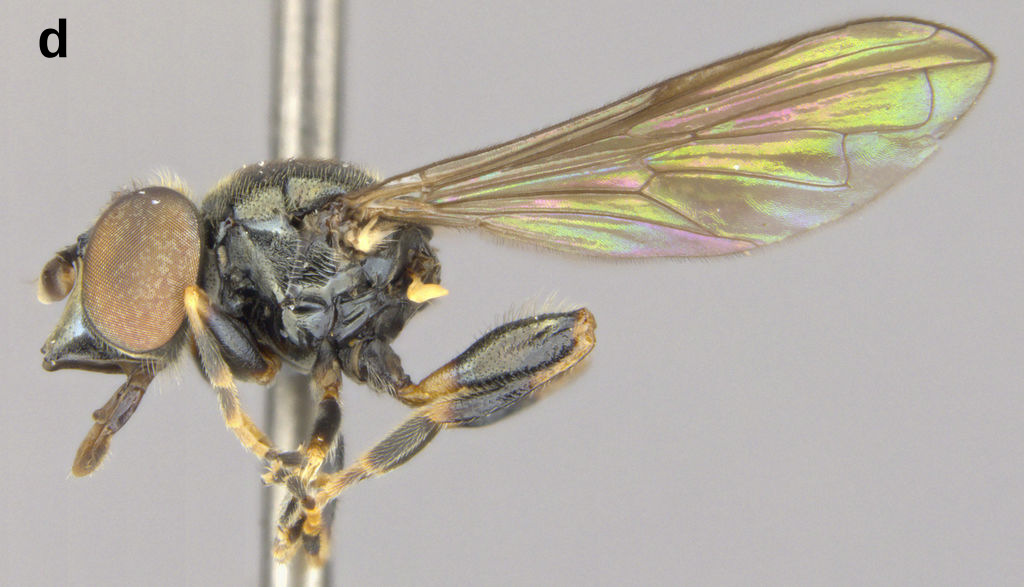
Lateral habitus, male, Holotype, CNC_Diptera170046

**Figure 11e.**
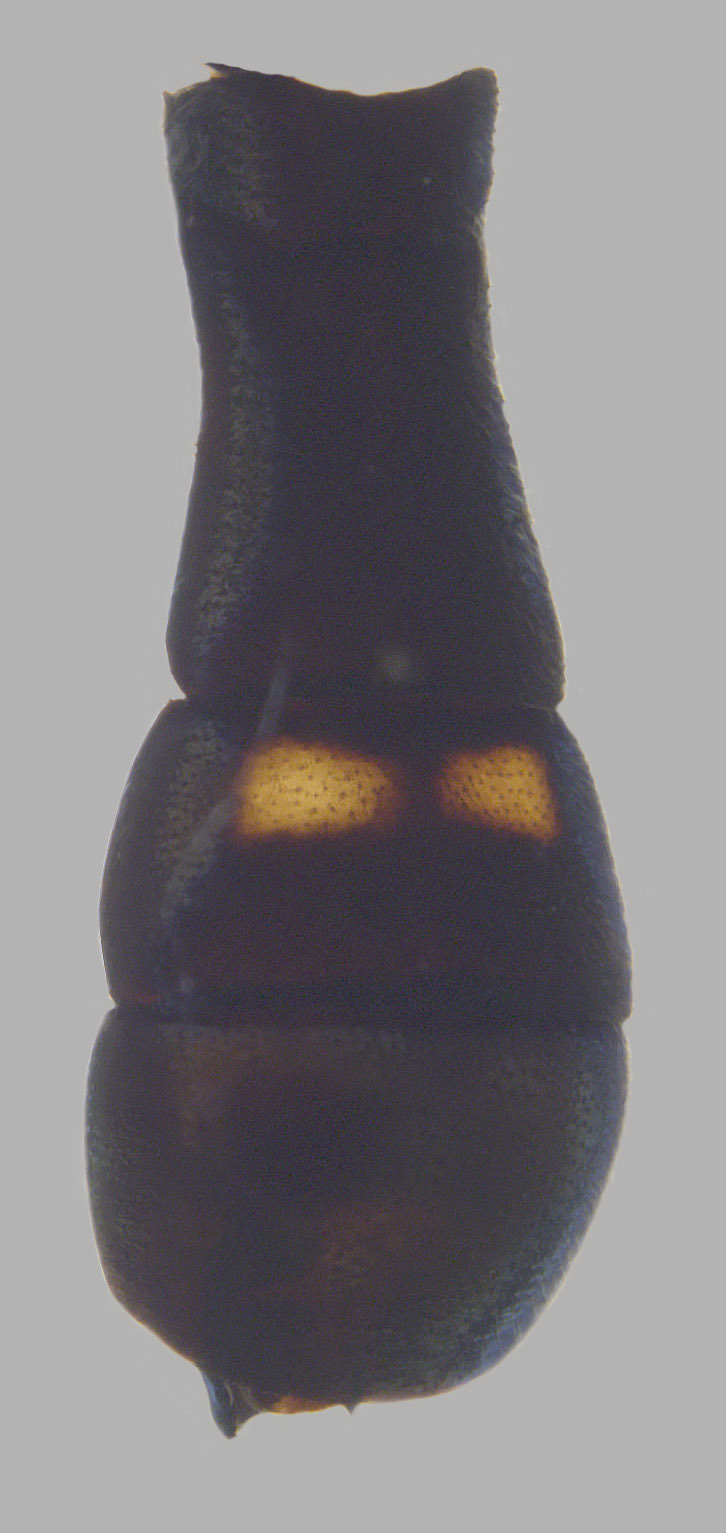
Dorsal abdomen, male, Holotype, CNC_Diptera170046

**Figure 11f.**
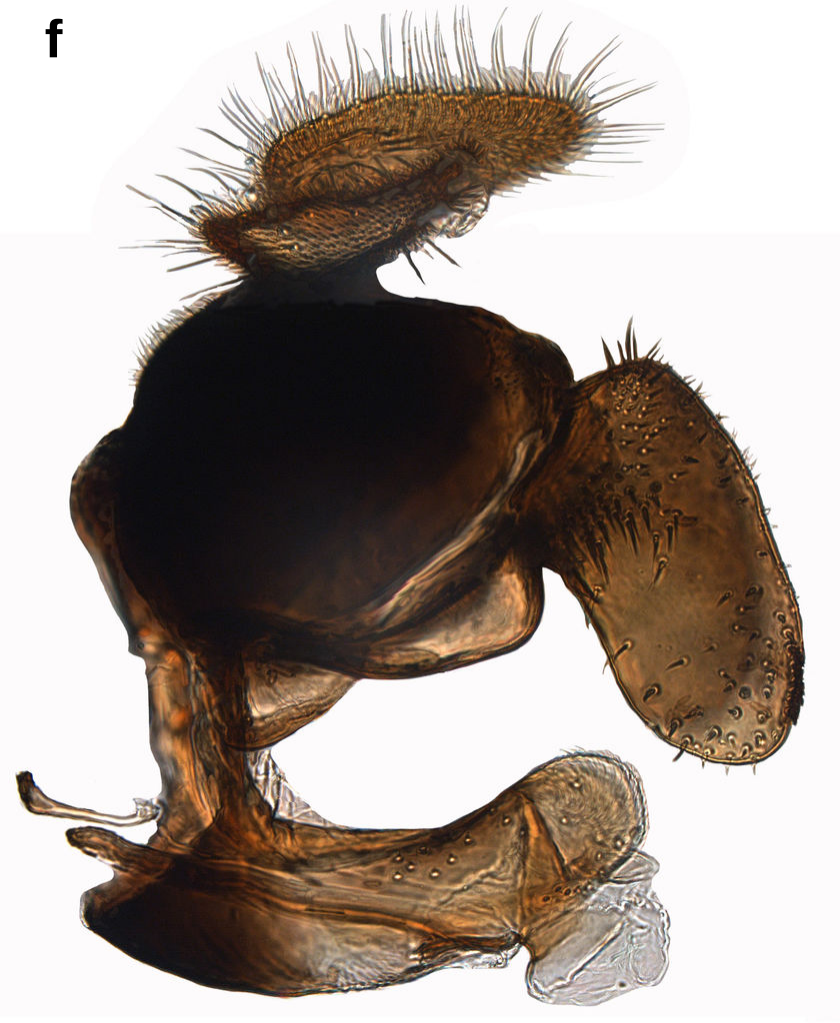
Lateral genitalia, male, Holotype, CNC_Diptera170046

**Figure 12a.**
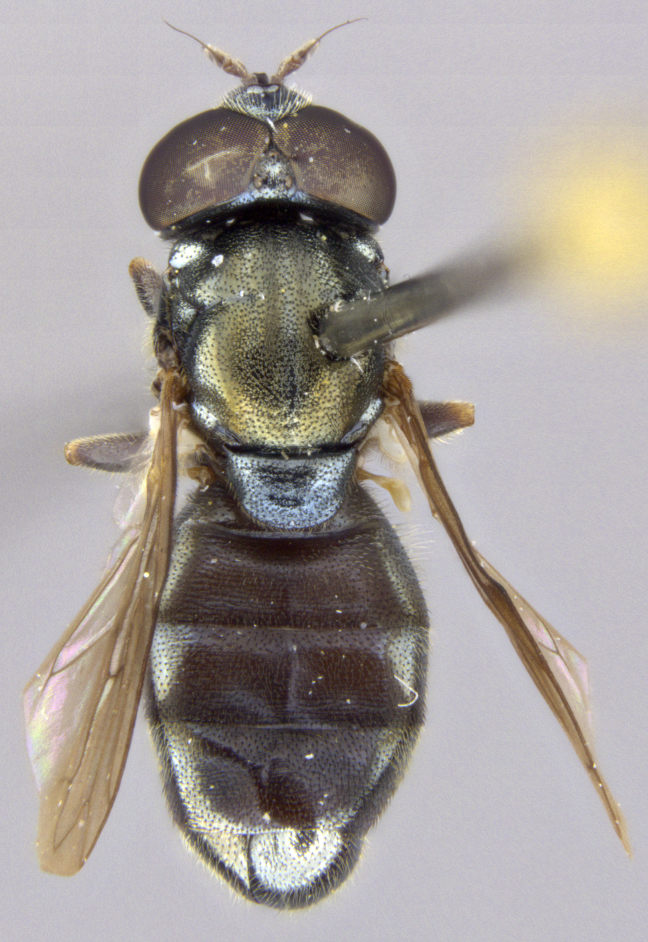
Dorsal habitus, male, Jeff_Skevington_Specimen44221

**Figure 12b.**
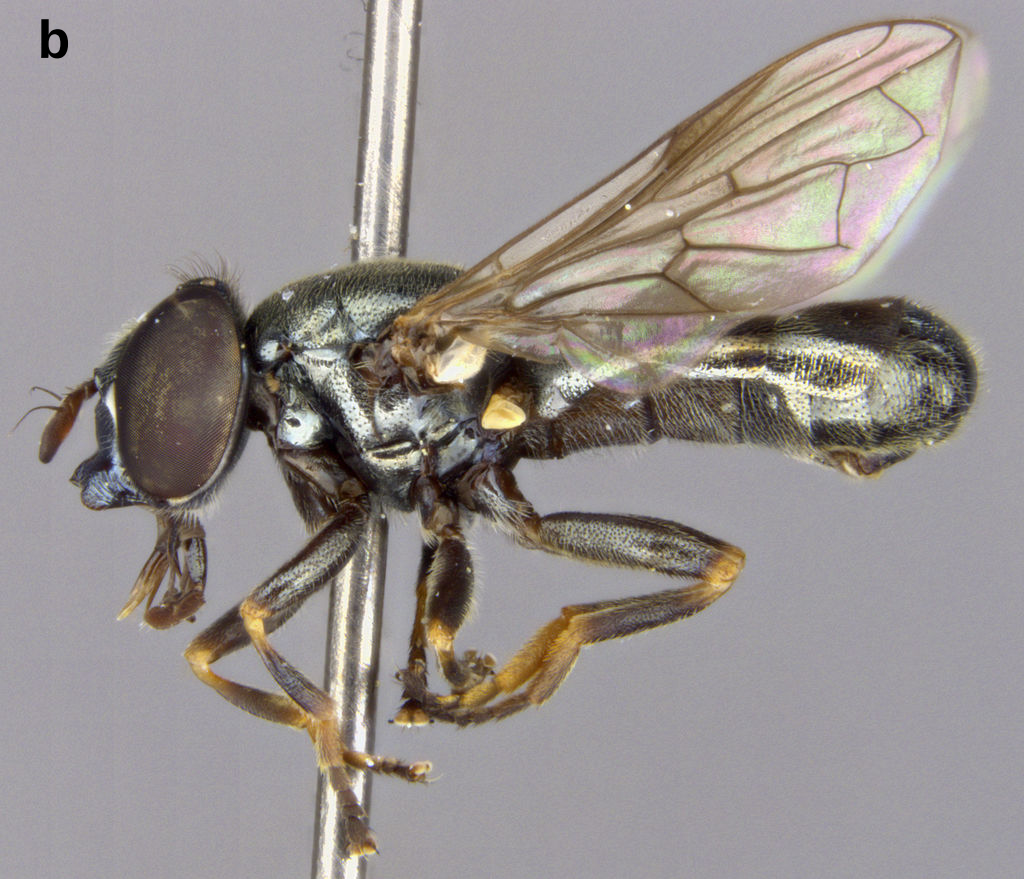
Lateral habitus, male, Jeff_Skevington_Specimen44221

**Figure 12c.**
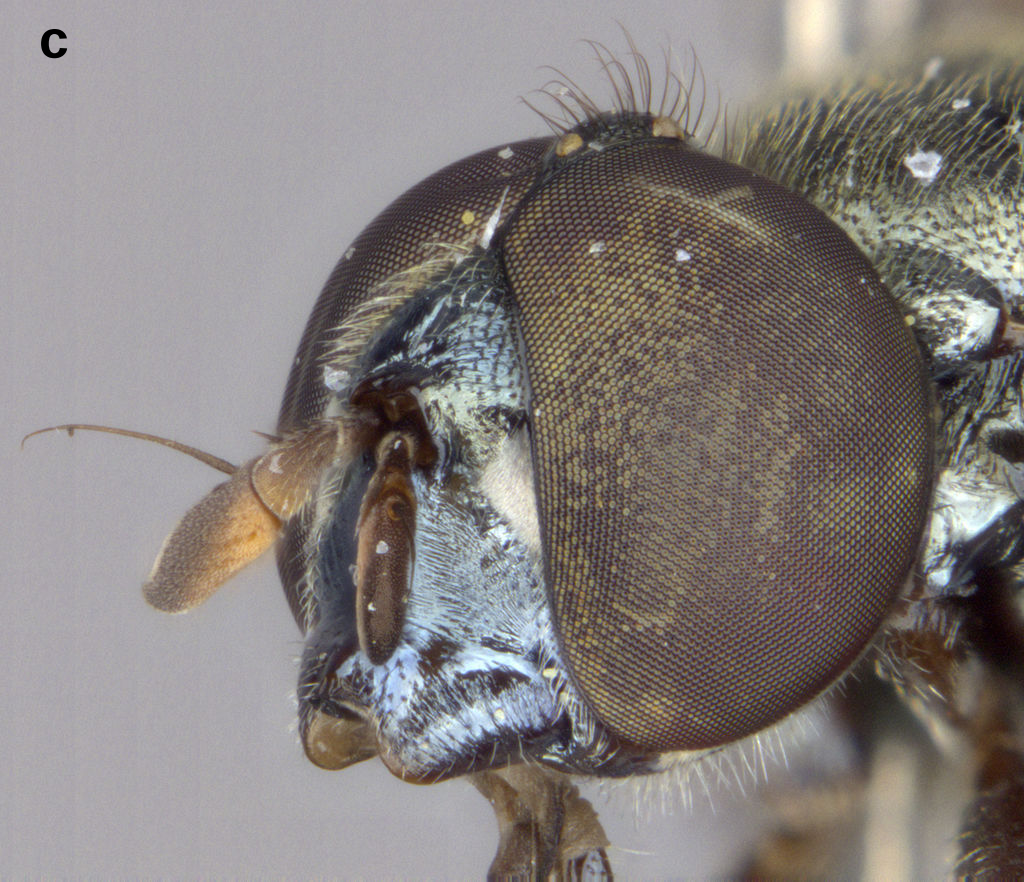
Face, oblique, male, Jeff_Skevington_Specimen44221

**Figure 12d.**
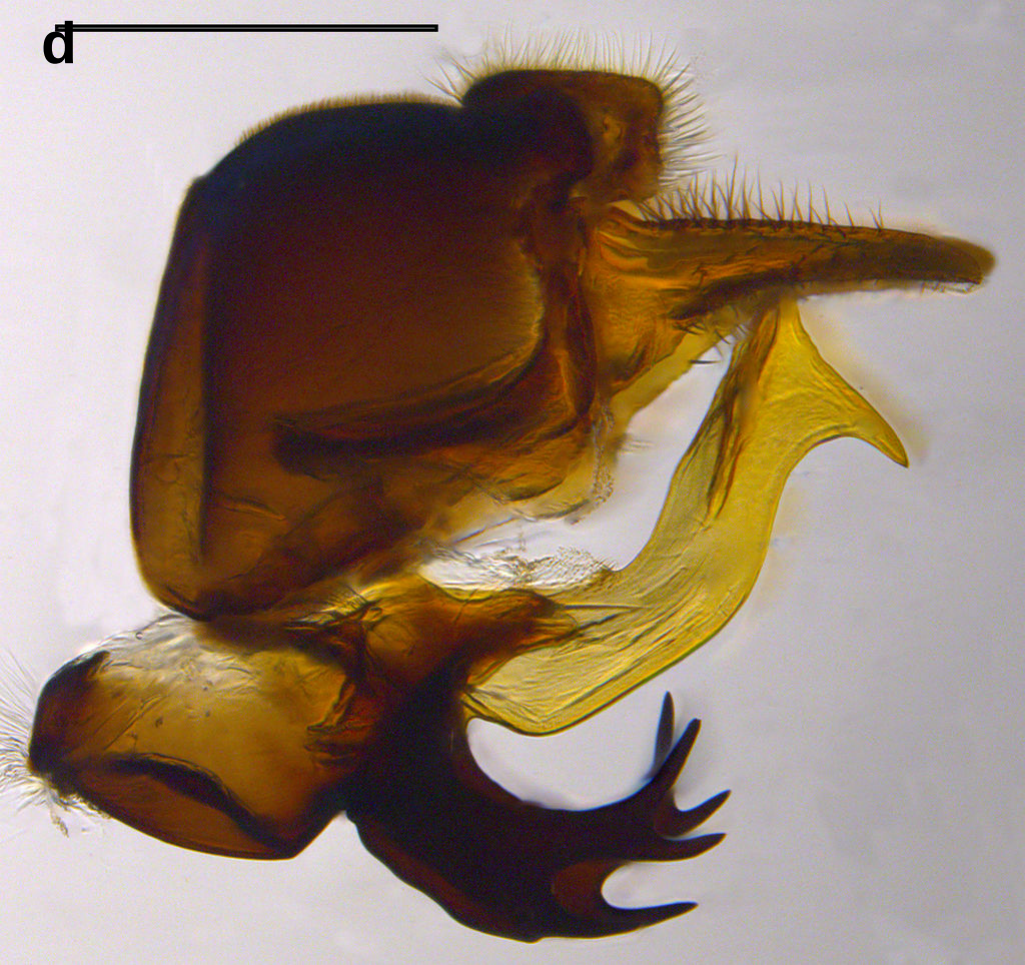
Lateral male genitalia, Jeff_Skevington_Specimen44222, Scale bar 500 µm

**Figure 12e.**
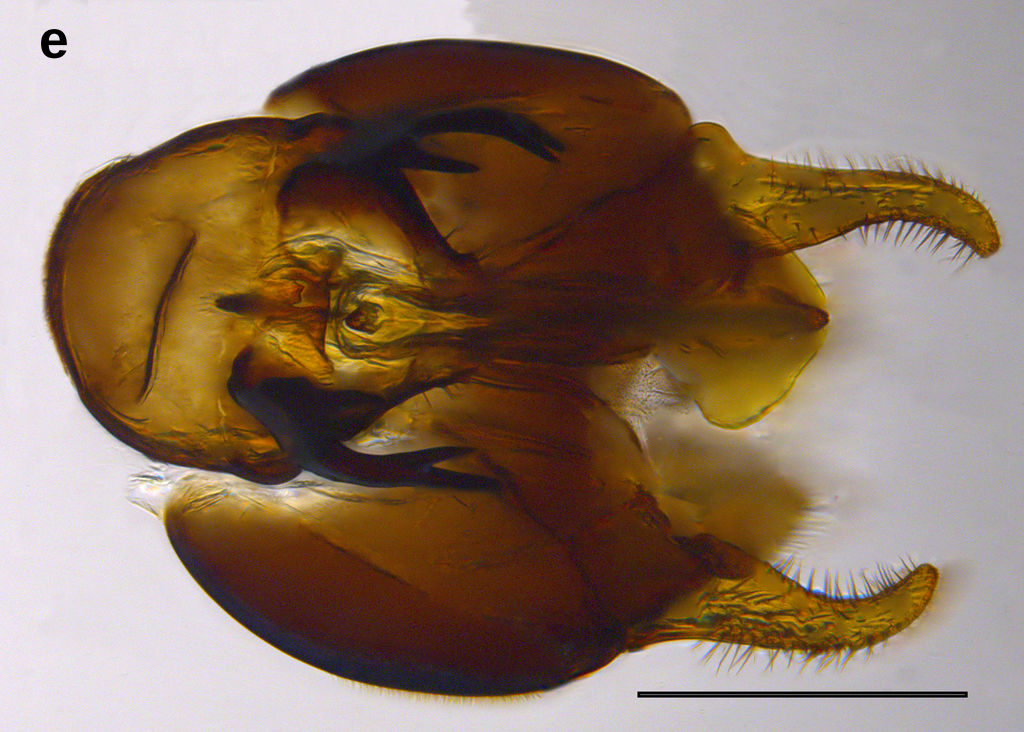
Ventral male genitalia, Jeff_Skevington_Specimen44222, Scale bar 500 µm

**Figure 13a.**
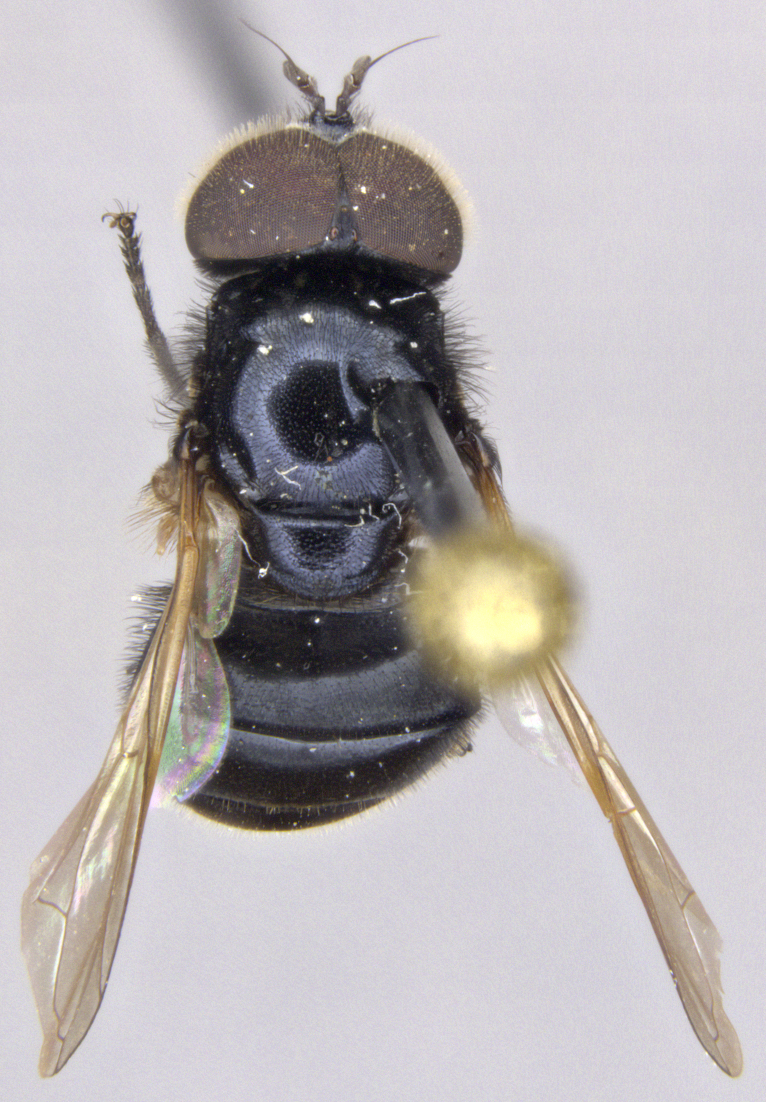
Dorsal habitus, Holotype, Jeff_Skevington_Specimen30434

**Figure 13b.**
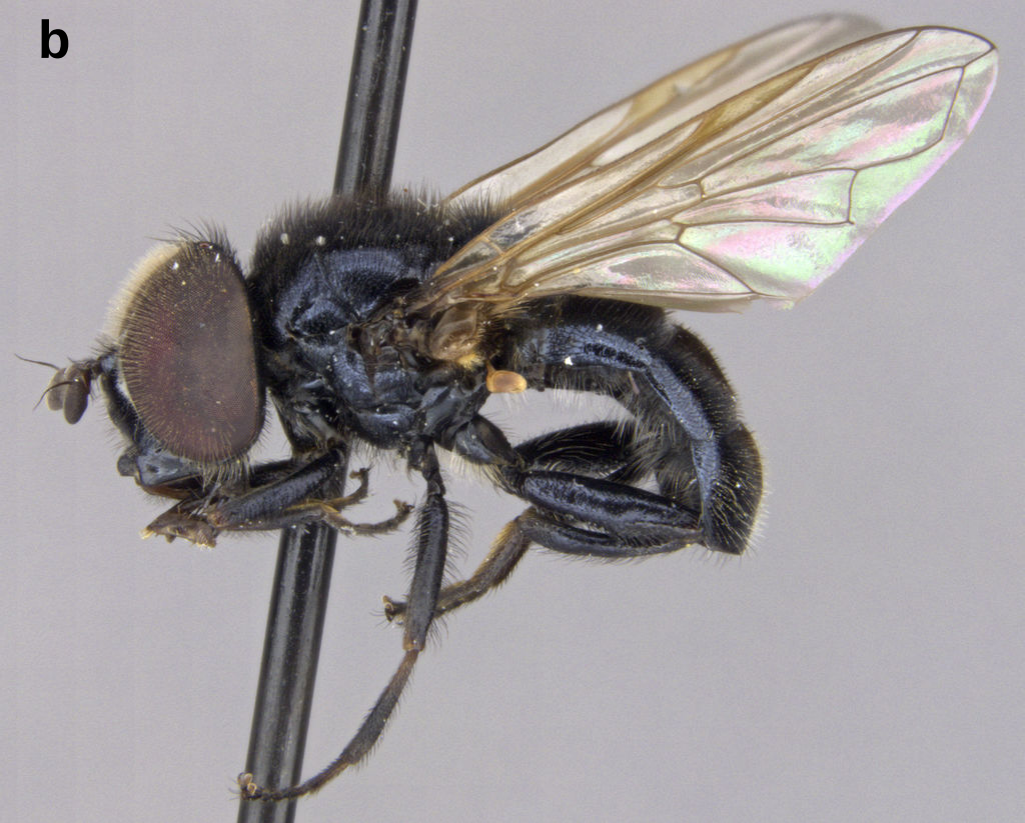
Lateral habitus, Holotype, Jeff_Skevington_Specimen30434

**Figure 13c.**
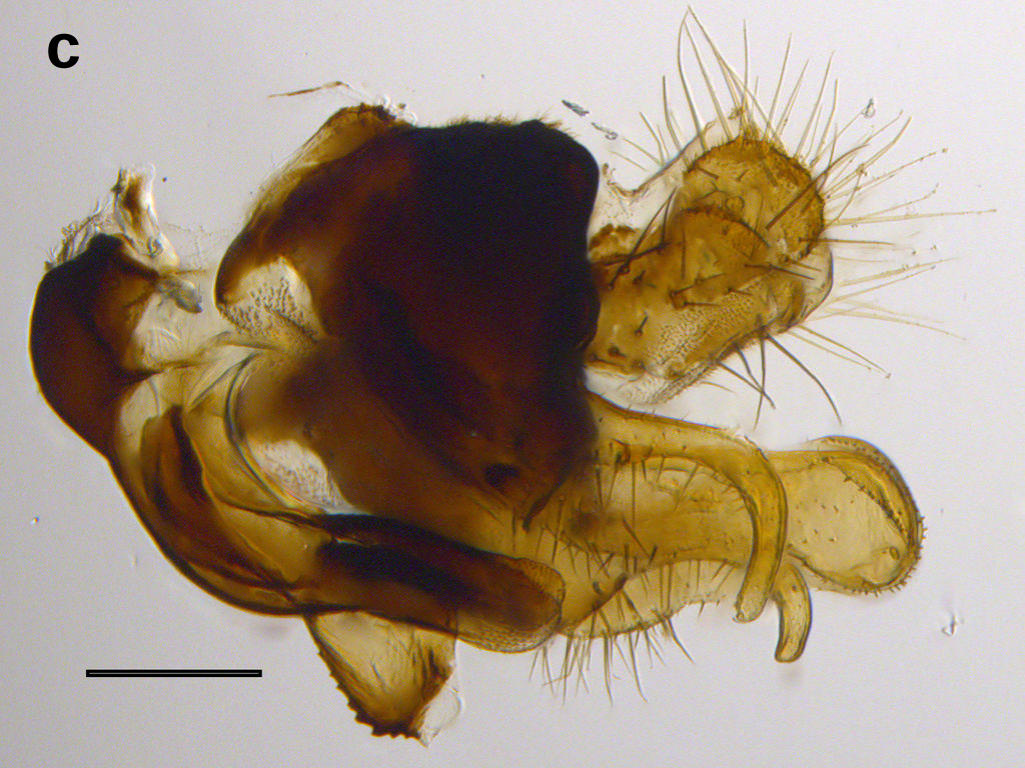
Lateral of male genitalia, Holotype, Jeff_Skevington_Specimen30434; scale bar 200µm

**Figure 13d.**
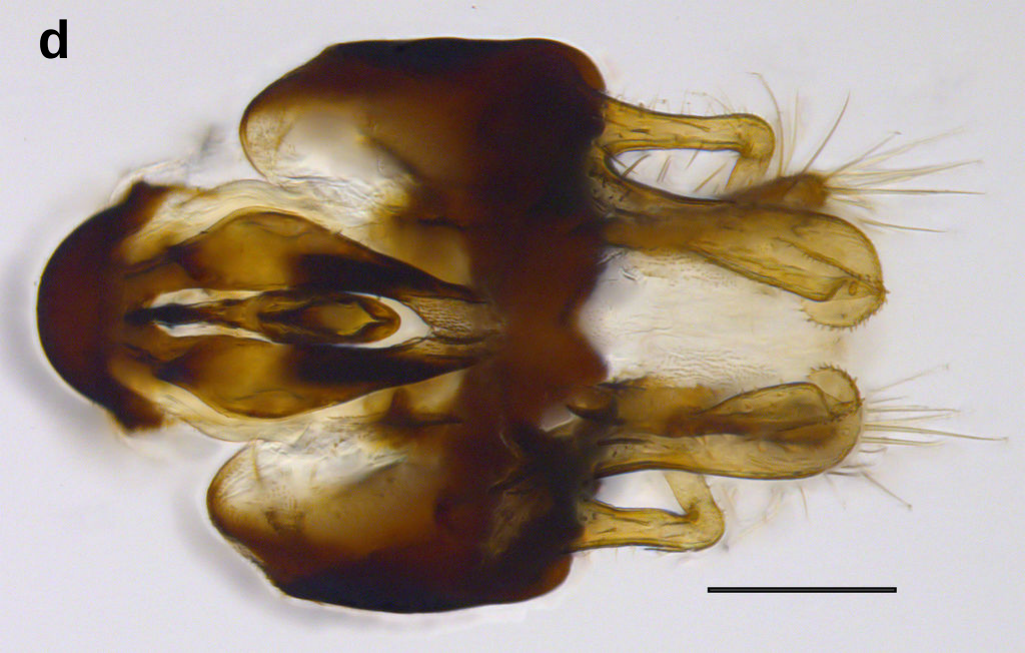
Ventral of male genitalia, Holotype, Jeff_Skevington_Specimen30434; scale bar 200µm

**Figure 14a.**
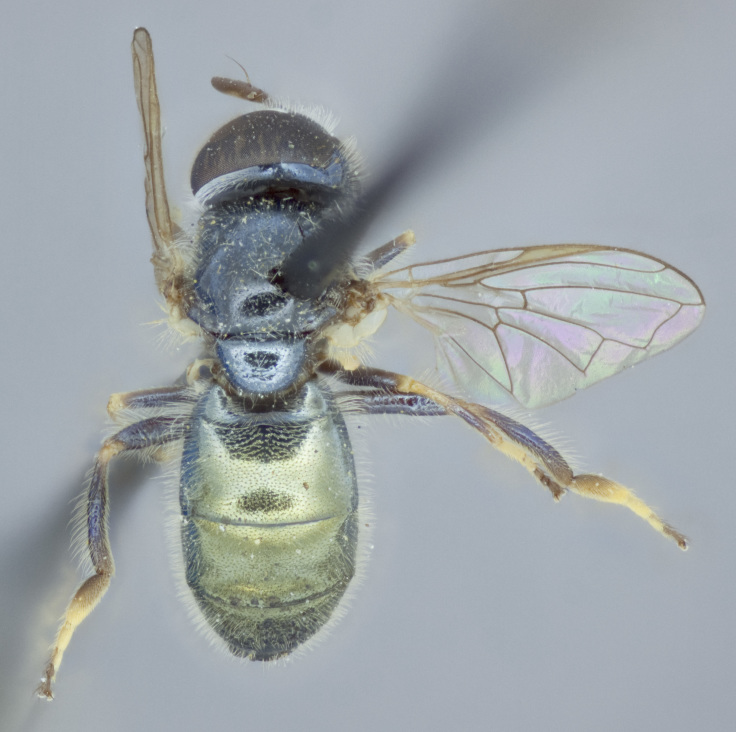
dorsal habitus, male, CNC_Diptera9769

**Figure 14b.**
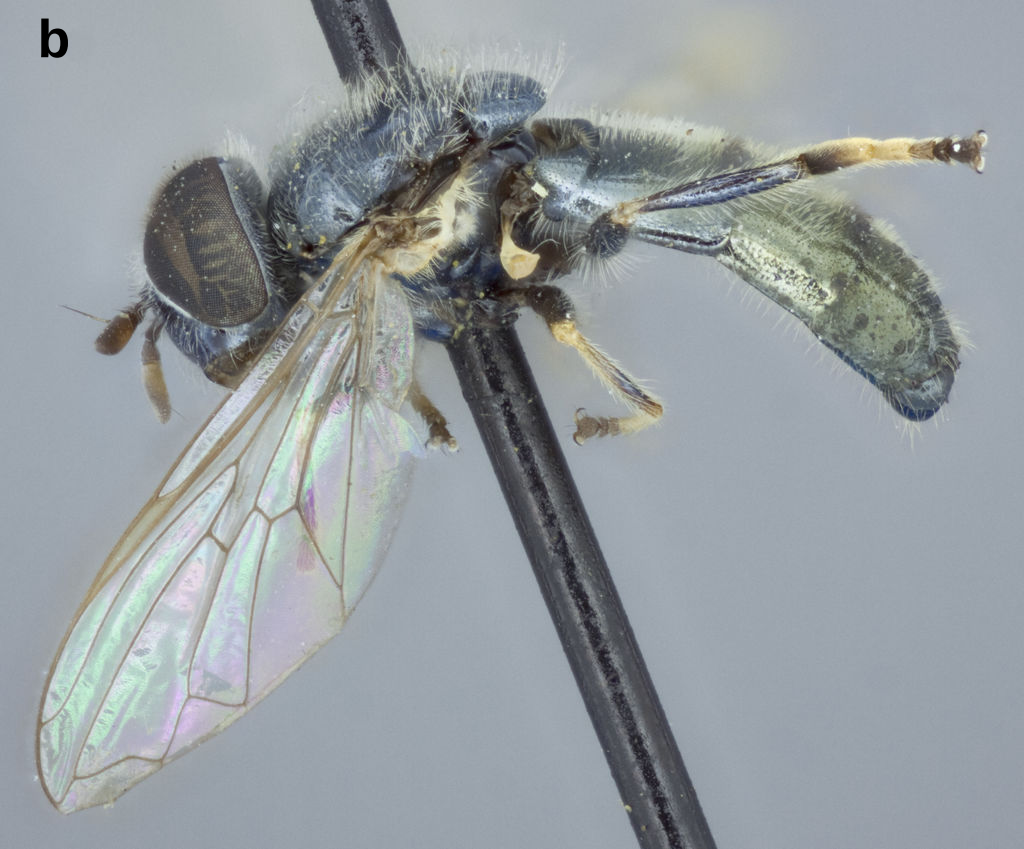
lateral habitus, male, CNC_Diptera9769

**Figure 14c.**
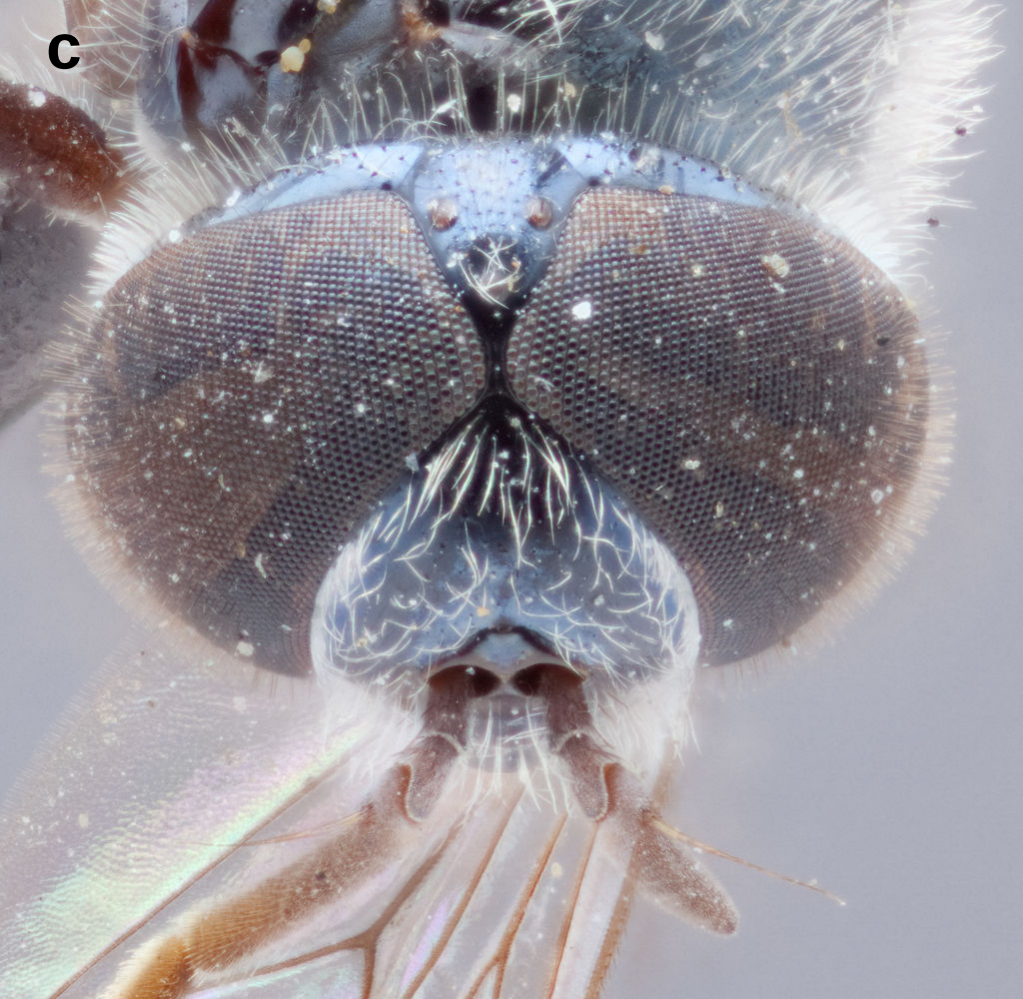
face, male, CNC_Diptera9769

**Figure 15a.**
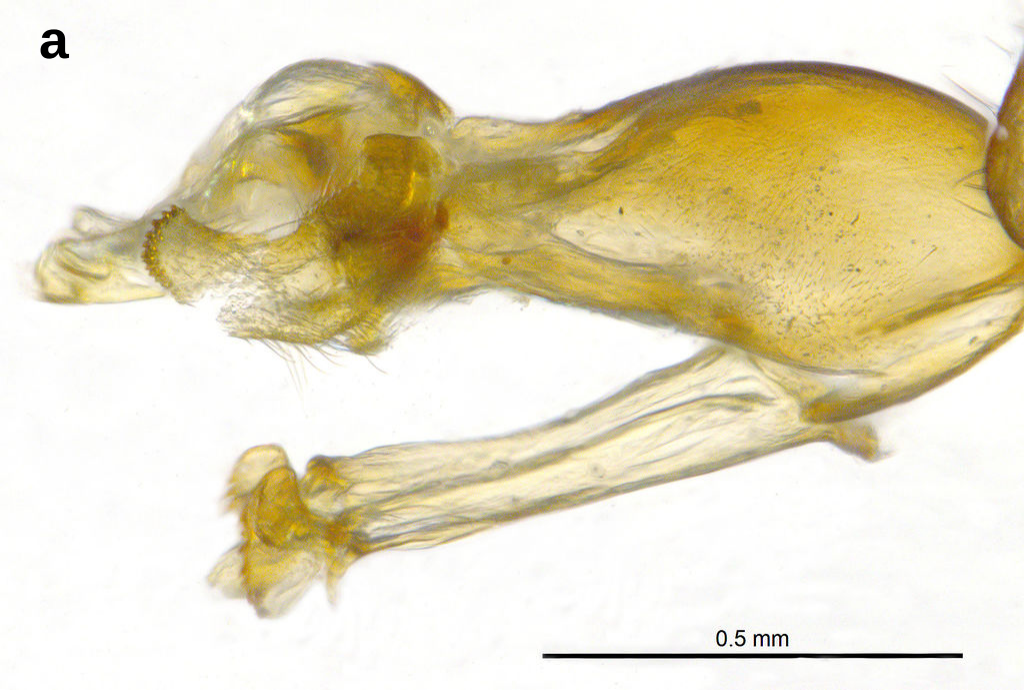
Lateral of genitalia, CNC_Diptera55045

**Figure 15b.**
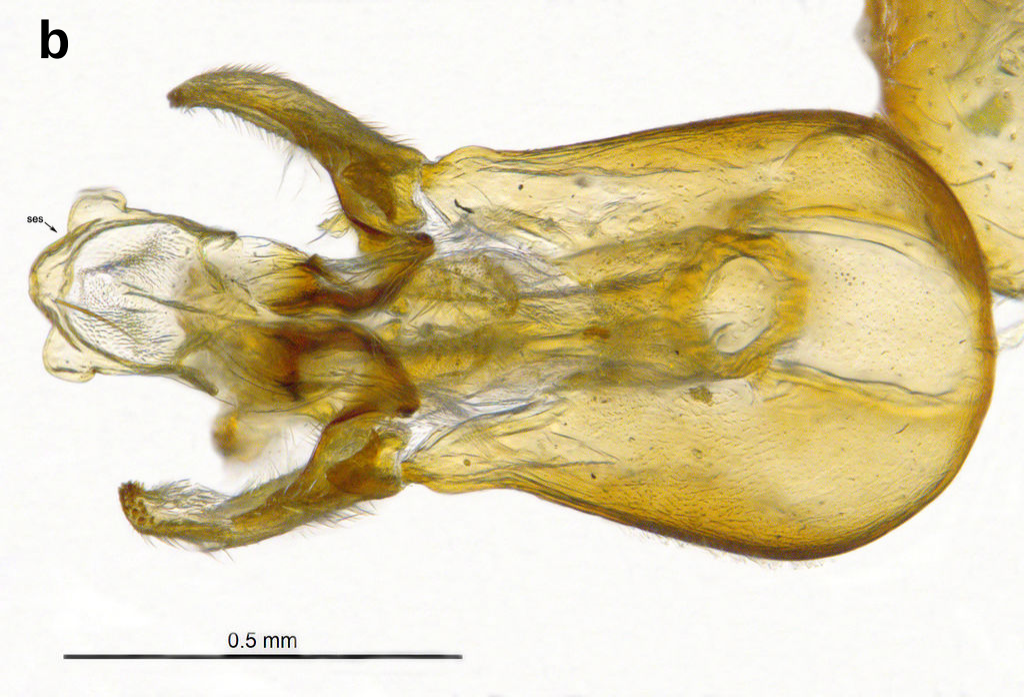
Dorsal of genitalia, ses: subepandrial sclerite, CNC_Diptera55045

**Figure 15c.**
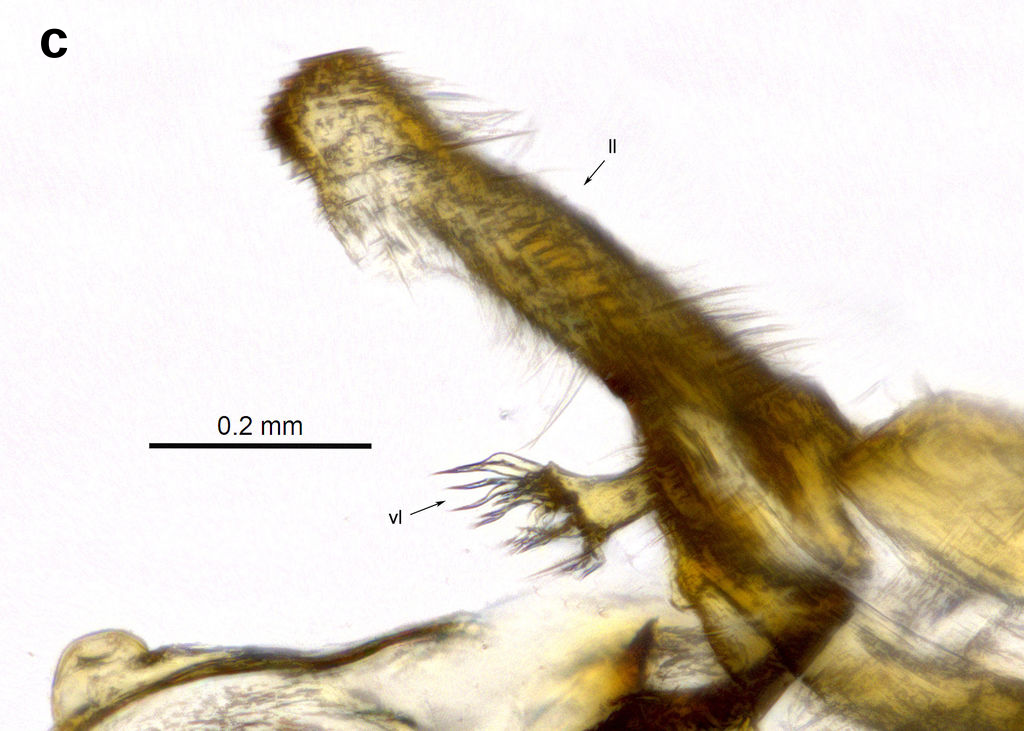
Ventral view of surstylus, ll: lateral lobe, vl: ventral lobe, CNC_Diptera55045

**Figure 15d.**
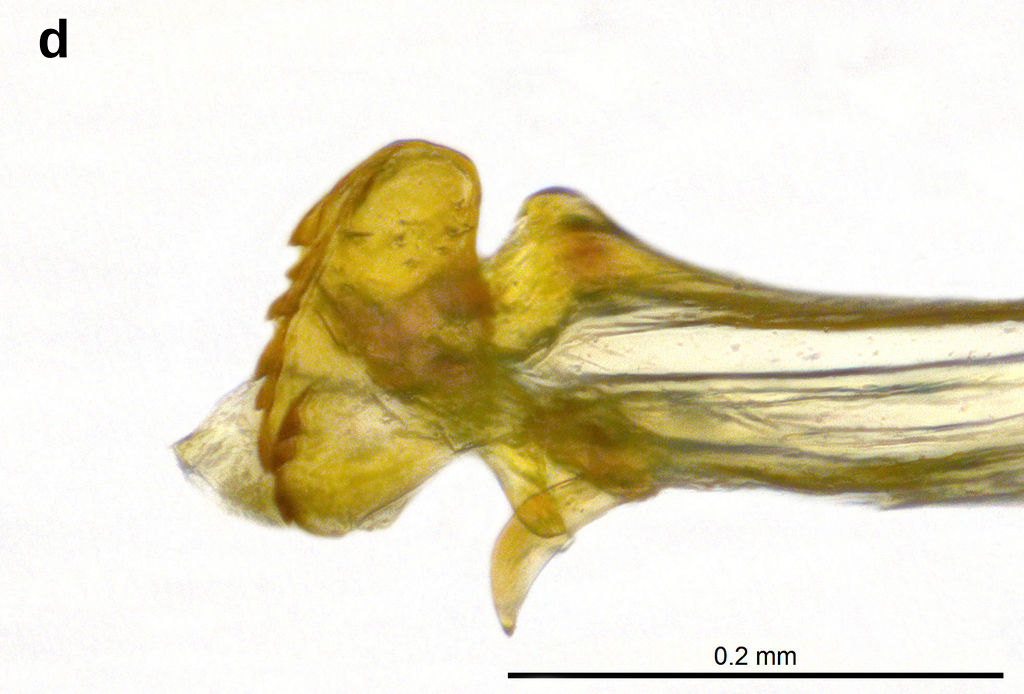
Phallus, CNC_Diptera55045

**Table 1.** Primers used to sequence COI in this study.

**Primer Name**	**Primer Design**	**Primer Sequence**
Heb-F	Folmer et al. 1994	GGT CAA CAA ATC ATA AAG ATA TTG G
COI-Fx-A-R	Young et al. (in prep)	CGD GGR AAD GCY ATR TCD GG
COI-Fx-B-F	Young et al. (in prep)	GGD KCH CCN GAY ATR GC
COI-Fx-B-R	Young et al. (in prep)	GWA ATR AAR TTW ACD GCH CC
COI-Fx-C-F	Young et al. (in prep)	GGD ATW TCH TCH ATY YTA GG
COI-780R	Gibson et al. 2011	CCA AAA AAT CAR AAT ARR TGY TG

**Table 2.** Sequenced Specimens.

**Species**	**Sample ID**	**Deposition**	**GenBank Number**	**Sequence Length**
*Anasimyiadiffusa*	10BBCDIP-0779	CBG	JF866884	633
*Anasimyiadiffusa*	10PROBE-13676	CNC	JF877353	658
*Anasimyiadiffusa*	BIOUG04123-H06	CBG	KM946129	592
*Anasimyiadiffusa*	BIOUG06854-C07	CBG	KM936389	567
*Anasimyiadiffusa*	CNC_Diptera102200	CNC	MN015555	609
*Anasimyiadiffusa*	CNC_Diptera102201	CNC	MN015565	658
*Anasimyiadiffusa*	CNC_Diptera106474	CNC	MN015574	375
*Anasimyiadiffusa*	CNC_Diptera44629	CNC	MN015577	583
*Anasimyiadiffusa*	CNC640314	CNC	MN015578	632
*Anasimyiadiffusa*	CNC640356	CNC	MN015572	633
*Anasimyiadiffusa*	CNC640369	CNC	MN015576	630
*Anasimyiadiffusa*	CNC640425	CNC	MN015557	631
*Anasimyiadiffusa*	CNC_Diptera4972	CNC	MN015558	576
*Anasimyiadiffusa*	CNC_Diptera4974	CNC	MN015559	575
*Brachyopacaesariata*	BIOUG01347-D12	CBG	KT104320	658
*Brachyopacaesariata*	BIOUG04288-F04	CBG	KR429817	658
*Brachyopacaesariata*	BIOUG04346-B01	CBG	KR433288	658
*Brachyopacaesariata*	BIOUG04570-D11	CBG	KR604654	670
*Brachyopacaesariata*	BIOUG05447-E11	CBG	KM950619	641
*Brachyopacaesariata*	BIOUG05966-A03	CBG	KM951714	617
*Brachyopacaesariata*	BIOUG20323-G12	CBG	MF837949	576
*Brachyopacaesariata*	BIOUG22325-H09	CBG	KT607903	564
*Brachyopacaesariata*	CNC_Diptera162914	CNC	MN015570	407
*Brachyopacaesariata*	CNC_Diptera37551	CNC	MN015564	658
*Hammerschmidtiasedmani*	BIOUG08107-B04	CBG	KM958957	661
*Hammerschmidtiasedmani*	BIOUG24033-B11	CBG	KT707288	506
*Hammerschmidtiasedmani*	CNC_Diptera49268	CNC	MN015556	658
*Hammerschmidtiasedmani*	CNC_Diptera49269	CNC	MN015560	658
*Hammerschmidtiasedmani*	CNC_Diptera49270	CNC	MN015571	658
*Hammerschmidtiasedmani*	CNC298243	CNC	MN015575	646
*Hammerschmidtiasedmani*	JK00736	CNC	MN015573	658
*Microdonscauros*	Jeff_Skevington_Specimen44177	ANSP	MN015566	667
*Mixogasterfattigi*	Jeff_Skevington_Specimen45174	ANSP	MN015561	660
*Neoasciaguttata*	CNC_Diptera169737	CNC	MN015562	667
*Neoasciaguttata*	CNC_Diptera170046	CNC	MN015567	434
*Psilotaklymkoi*	JK5333	CNC	MN015569	646
*Trichopsomyialitoralis*	CNC_Diptera246379	CNC	MN015568	247
